# An Integrated High Throughput Experimentation/Predictive QSAR Modeling Approach to *ansa*-Zirconocene Catalysts for Isotactic Polypropylene

**DOI:** 10.3390/polym12051005

**Published:** 2020-04-27

**Authors:** Christian Ehm, Antonio Vittoria, Georgy P. Goryunov, Vyatcheslav V. Izmer, Dmitry S. Kononovich, Oleg V. Samsonov, Rocco Di Girolamo, Peter H. M. Budzelaar, Alexander Z. Voskoboynikov, Vincenzo Busico, Dmitry V. Uborsky, Roberta Cipullo

**Affiliations:** 1 Dipartimento di Scienze Chimiche, Università di Napoli Federico II, Via Cintia, 80126 Napoli, Italy; antonio.vittoria@unina.it (A.V.); rocco.digirolamo@unina.it (R.D.G.); p.budzelaar@unina.it (P.H.M.B.); busico@unina.it (V.B.); 2 Department of Chemistry, Lomonosov Moscow State University, 1/3 Leninskie Gory, 119991 Moscow, Russia; goryunov@org.chem.msu.ru (G.P.G.); izmer_slava@mail.ru (V.V.I.); dkononovich@org.chem.msu.ru (D.S.K.); oleg.samson@mail.ru (O.V.S.); voskoboy@med.chem.msu.ru (A.Z.V.); 3 Dutch Polymer Institute (DPI), P.O. Box 902, 5600 AX Eindhoven, The Netherlands

**Keywords:** olefin polymerization, stereoselectivity, regioselectivity, molecular weight capability, molecular catalysts, QSAR, i-PP

## Abstract

Compared to heterogenous Ziegler–Natta systems (ZNS), *ansa*-metallocene catalysts for the industrial production of isotactic polypropylene feature a higher cost-to-performance balance. In particular, the *C*_2_-symmetric *bis*(indenyl) *ansa*-zirconocenes disclosed in the 1990s are complex to prepare, less stereo- and/or regioselective than ZNS, and lose performance at practical application temperatures. The golden era of these complexes, though, was before High Throughput Experimentation (HTE) could contribute significantly to their evolution. Herein, we illustrate a Quantitative Structure – Activity Relationship (QSAR) model trained on a robust and highly accurate HTE database. The clear-box QSAR model utilizes, in particular, a limited number of chemically intuitive 3D geometric descriptors that screen various regions of space in and around the catalytic pocket in a modular way thus enabling to quantify individual substituent contributions. The main focus of the paper is on the methodology, which should be of rather broad applicability in molecular organometallic catalysis. Then again, it is worth emphasizing that the specific application reported here led us to identify in a comparatively short time novel zirconocene catalysts rivaling or even outperforming all previous homologues which strongly indicates that the metallocene story is not over yet.

## 1. Introduction

Innovation in catalysis, i.e., the development of novel processes or products, selectivity or productivity enhancements for existing processes, etc., is a key driver of the chemical industry, and is typically a trial-and-error process. In recent times, the integration of High Throughput Experimentation (HTE) [1,2,3,4,5,6,7,8] with statistical modeling has been found to be a powerful accelerator. Regarding the latter, Quantitative Structure – Activity Relationship (QSAR) methods, originally introduced in pharmaceutical chemistry [9], are now spreading in several domains of chemical R&D [10,11,12,13].

The polyolefin industry pioneered this trend. In the late 1990s, companies (e.g., Dow Chemical) engaged in massive HTE-based catalyst discovery programs [2,14,15,16,17], using parallel reactor setups and workflows pioneered by Symyx [4,18] Those early applications focused on speed, that was often traded for accuracy at least in the so-called ‘primary screening’ stage. As a matter of fact, the initial identification of catalyst hits/leads was followed by structure optimization with conventional methods. More recently, in our labs we found ways to improve the precision and accuracy of the approach, by implementing highly controlled polymerization protocols on a state-of-the-art HTE reaction platform (Freeslate—former Symyx—Parallel Polymerization Reactor (PPR)), and integrating said platform with fast and thorough polymer characterization techniques, including Rapid-GPC, high-temperature cryoprobe ^13^C NMR, Differential Scanning Calorimetry (DSC), and analytical Crystallization Elution Fractionation (aCEF) [19,20,21,22,23,24]. This workflow is now exploited to rapidly generate large catalyst structure-properties databases for optimization using predictive QSAR modeling [13]. In this paper, we illustrate how the approach was successfully applied to *C*_2_-symmetric a*nsa*-zirconocene catalysts for the isotactic-selective polymerization of propene. 

Known since the 1950s [25,26], Group 4 metallocenes gained credit as practical ethene polymerization catalysts in the mid-1970s, when the serendipitous discovery of the methylalumoxane (MAO) activator boosted productivity by several orders of magnitude [27,28]. In the following years, the simple unbridged bis(cyclopentadienyl) ligand framework was elaborated into a number of stereorigid bridged variants with different symmetries, including several precatalyst families with chirotopic active sites [29,30]. This opened the era of molecular stereoselective propene polymerization catalysis. A fierce R&D competition involved practically all polyolefin companies active at that time, and several billion USD were spent globally to identify molecular catalysts able to compete with classical heterogeneous Ziegler–Natta systems (ZNS) [22,31,32,33,34,35]. The main goal was the industrial production of isotactic polypropylene (i-PP), and by the end of the 1990s a number of companies had approached that target.

However, even the best champions in the field, such as the class of *rac*-Me_2_Si(2-R^1^-4-R^2^-1-Indenyl)_2_ZrX_2_ [36,37] complexes introduced by Spaleck and co-workers at Hoechst (Spaleck type zirconocenes), their homologues with fused aromatic cycles [38], and the ultra-rigid Hf-based homologues disclosed more recently by Rieger and co-workers [39,40], could not match the exceptional cost-to-performance balance of ZNS, particularly at industrially relevant temperatures (≥70 °C).

As a matter of fact, the market of metallocene i-PP has never grown above the size of a specialty niche (currently, less than 5% by volume of total i-PP production [31,32,41]). By the end of the last millennium, the perception that an upper limit had been attained became tangible in the chemical community [42], and from that time onward the research efforts declined rapidly. Notably, this was before HTE and QSAR modeling could contribute to any significant extent. 

It may be worthy to recall at this point that, according to the definition in pharma, the QSAR is a mathematical function of the form [43]:

*P*_i_ = *k*’(*D*_1i_, *D*_2i_, …, *D*_ni_) (1)

where *P*_i_ are biological activities of a set of compounds of interest, *D*_ji_ are calculated (or, sometimes, experimentally measured) structural properties of said compounds (so-called ‘molecular descriptors’), and *k*’ is some empirically established mathematical transformation (e.g., a linear or non-linear combination) that can be applied to descriptors to calculate *P*_i_ for all compounds in the examined set. The underlying principle is that compounds with similar structures are also characterized by similar activities, and the goal is to use *k*’ in order to predict the activity of yet unknown compounds added to the set by interpolation or, possibly, by moderate extrapolation. An obvious (albeit sometimes overlooked) point is that no QSAR model can be of better quality than the experimental database on which it was trained [43]: to guarantee solid and reliable predictions, a database must be large enough to avoid over-fitting, and accurate enough to yield a meaningful correlation.

When the concept is generalized in a broad chemical context, by ‘activity’ one must intend a number of different and often highly diversified performance properties. In the specific case of propene polymerization catalysts, these typically include monomer insertion rate, stereoselectivity, regioselectivity, and polymer molar mass capability. Among the several reasons why *ansa*-metallocenes, in general, represent excellent objectives for QSAR elaboration, an important one is that the stereorigid ligand framework greatly simplifies the design and quantification of well-working descriptors. That said, the fact that these catalysts can perform outstandingly has long been demonstrated [13,36,37,44,45,46]. The last decades have brought copious amounts of performance data in the literature, however, the extreme sensitivity of these catalysts to the activation protocol and use conditions renders the compilation of a coherent database for QSAR modeling from multiple literature sources nearly impossible [29,30,47,48,49]. 

Specifically, the *ansa*-bis(1-indenyl) frame is amenable to structural amplification with innumerable substitution patterns. However, the difficult and highly idiosyncratic multi-step protocols of ligand preparation, metalation, and isomeric purification of these compounds has long represented a major drawback for HTE. It was only recently that convenient methods of parallel synthesis and rapid purification were introduced [50,51]. 

**Figure 1:**
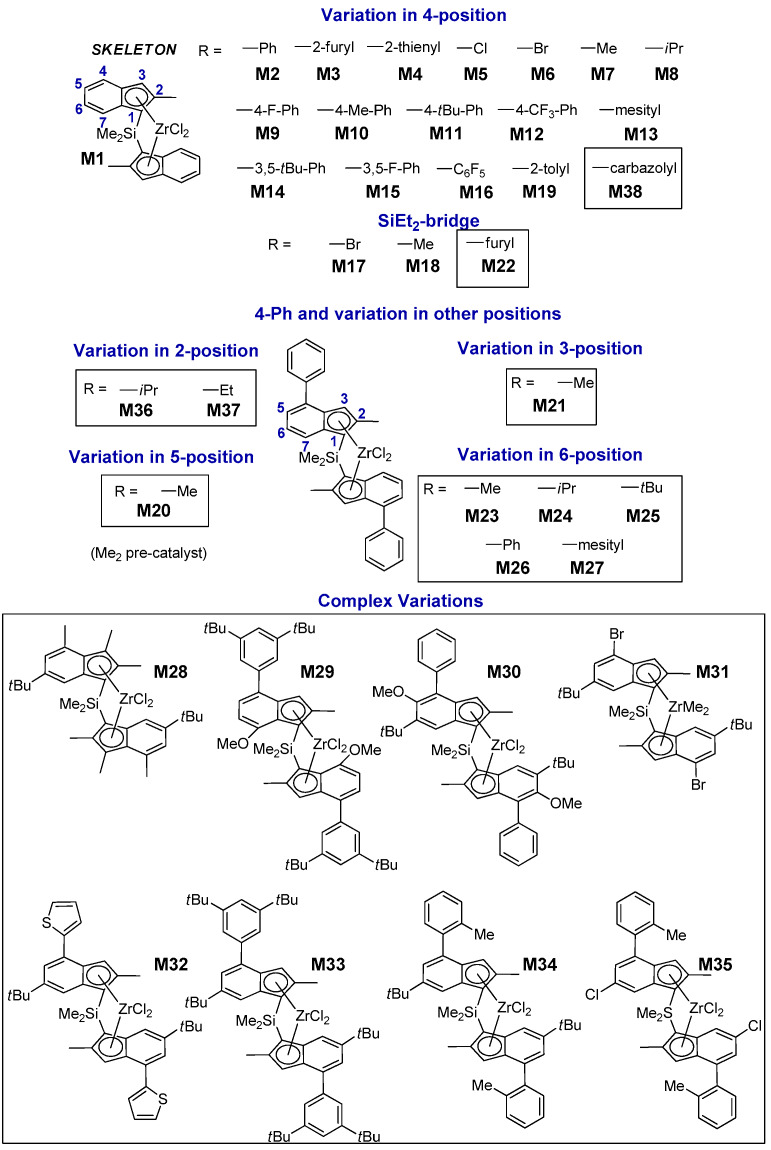
LZrCl_2_ precatalysts **M1**–**M38** synthesized and screened in propene homopolymerization at *T*_p_ = 60 °C and *p*(C_3_H_6_) = 6.6 bar in toluene. The performance of catalysts **M1**–**M19** which are part of the present Quantitative Structure – Activity Relationship (QSAR) study has been described in Reference [13].

Taking advantage of this progress, we assembled a library of 38 *C*_2_-symmetric *ansa*-zirconocene precatalysts with large structural diversity (Figure 1 and Supporting Information (SI)); of these, 22 were already known from the previous literature, whereas 16 are novel. Catalysts **M1**–**M19** feature exclusively variations in the 4-R position or in the bridge [13]. The newly added catalysts **M20**–**M38** expand the database to include variations in all other 1-indenyl positions (i.e., 2-, 3-, 5-, 6-, and 7-position), which have varying degrees of influence on polymerization performance. 

All catalysts were screened in propene homopolymerization at *T*_p_ = 60 °C and *p*(C_3_H_6_) = 6.6 bar in toluene solution using the aforementioned HTE workflow. These conditions were chosen to ensure that the side process of growing chain epimerization [29,30,52,53,54,55] was negligible [13,52,53]. The resulting database was used to train predictive QSAR models for stereoselectivity, regioselectivity and polymer molar mass capability. The predictive ability of the models was then tested on a validation set of five new catalysts (**M39**–**M43**) specifically prepared for the purpose.

## 2. Materials and Methods

Catalyst Synthesis: **M1** and **M2** were kindly donated by SABIC and used as received. **M6** and **M17** [56], **M4** [51], **M8** [36], **M7**, **M9**, **M10**, and **M13** [57] and **M11**, **M14**, and **M15** [58], **M26** [51], **M29** [39], **M30** [46], **M37** [59], **M38** [60], **M3**, **M5**, **M12**, **M16**, **M18,** and **M19** [13] were synthesized according to the literature. The synthesis of **M20**–**M28**, **M31**-**M36,** and **M39**–**M43** is detailed in the SI. 

Polymer Synthesis and Characterization: All propene polymerization experiments were performed in toluene in a Freeslate Parallel Pressure Reactor (Freeslate, San Francisco, CA, USA) setup with 48 reaction cells (PPR48), fully contained in a triple MBraun glovebox under nitrogen. The cells, with a working volume of 5.0 mL, feature an 800 rpm magnetically coupled stirring, and individual online reading/control of temperature, pressure, monomer uptake, and uptake rate. The setup and the operating protocol, described in full detail in References [19,20], are reported in the SI and have been used successfully before in various homogeneous and heterogeneous polymerization studies [13,21,24]. Polymerization conditions were as follows (and identical to those used in Reference [13]): *T* = 60 °C; *p*(C_3_H_6_) = 4.5 or 6.6 bar; activator and scavenger Al(*iso*-butyl)_3_ (TIBA)/N,N-dimethylanilinium tetrakis(perfluorophenyl)borate (AB) or MAO. Operating at two propene partial pressures was aimed to verify that growing chain epimerization did not interfere under the investigated conditions. The catalysts were not pre-activated prior to injection into the PPR cells.

The polymers were characterized by: (a) high-temperature GPC with a Freeslate Rapid-GPC setup; (b) quantitative ^13^C{^1^H} NMR with a Bruker DRX 400 spectrometer equipped with a high-temperature cryoprobe (for 5 mm OD tubes) and a pre-heated robotic sample changer; and (c) DSC with a Mettler Toledo DSC-822 calorimeter (Columbus, OH, USA). Polymer melting points (*T*_m_) were collected from the second heating scan. All results are averages on polymer samples produced in polymerization experiments performed in duplicate. More details can be found in the SI, Tables S1 and S2.

Computational Details: Geometries of LMCl_2_ complexes were fully optimized using the Gaussian16 software package [61] in combination with the OPTIMIZE routine of Baker [62,63] and the BOpt software package [64]. Following the protocol proposed in Reference [65], all pre-catalysts were optimized at the TPSSTPSS/cc-pVDZ(-PP) [66,67,68,69] level of theory, using a small core pseudo potential on Zr [70,71]. The protocol has been successfully used, in combination with M06-2X [72] single-point energies (SP), to address several polymerization related problems: i.e., absolute barrier heights for propagation [73], comonomer reactivity ratios [74,75], metal-carbon bond strengths under polymerization conditions [76,77,78], electronic and steric tuning of *M*_W_ capability [79], and QSAR modeling [13]. The density fitting approximation (Resolution of Identity, RI) [80,81,82,83] and standard Gaussian16 quality settings [Scf = Tight and Int(Grid = Fine)] were used throughout. All structures represent true minima (as indicated by the absence of imaginary frequencies). Buried volume descriptors were calculated using the SambVca 2.0 program [84]. The Natural Population Analysis (NPA) charges were determined from SP calculations at the M06-2X/cc-pVTZ(-PP) level of theory using the NBO 3.0 program [85], implemented in Gaussian16. The minimum structure of **M3**/**M22** features the furyl rings oriented towards the center of the catalysts. In this case a structure with the furyl rings frozen in the angle found in the lowest insertion TS structure was used for the QSAR models, for more details see Reference [13].

QSAR Models: QSAR models consisting of up to five descriptors were built via multiple regression analysis (LINEST function). Accepted models show *p*-values < 0.05 for all contributing descriptors and the intercept. Leave-one-out analysis was performed with *R* in *RStudio*.

## 3. Results and Discussion

When the precatalysts of Figure 1 were activated in toluene solution with methylalumoxane (MAO) or a combination of Al(*iso*-butyl)_3_ and trityl tetrakis-perfluorophenylborate (TIBA/TTB), propene polymerization was in most cases too fast (TOF > 10^3^ s^−1^ at *T* = 60 °C and *p*(C_3_H_6_) = 6.6 bar) for good reaction control in the small PPR reaction cells (5.0 mL working volume each). A protocol to modulate the reaction rate almost at will without affecting polymer properties was implemented using as the activator a combination of TIBA and N,N-dimethylanilinium tetrakis-perfluorophenylborate (AB) [86]. The PP samples for the present study were prepared purposely at moderate reaction rate (see Experimental Section and SI), and polymer properties (namely stereoregularity, regioregularity, and average molar mass) turned out to be highly reproducible (much more so than upon MAO or TIBA/TTB activation). 

Notably, with several catalysts in the set the generation of stereodefects and/or chain termini turned out to fall under the definition of ‘rare events’ (<0.1% of TOF). Measuring said microstructural features with high accuracy in HTE mode represented an extreme but also mandatory technical challenge [19,20,21], because i-PP properties depend crucially on them, and even minute differences in their fractional abundance have a strong impact on the polymer application window [87,88,89,90]. We managed to overcome the problem with the only exception of catalysts approaching ‘perfect’ stereoselectivity (>0.9998%), ranking such catalysts was not possible since the fraction of stereodefects in the polymers turned out to be lower than our ^13^C NMR evaluation uncertainty (±0.02 mol%). 

The experimental QSAR database is summarized in Table 1. Catalyst stereoselectivity (enantioselectivity, *σ*, that is the mole fraction of monomer insertions with the preferred enantioface) ranged between 0.9720 and >0.9998 (i.e., from 2.8 to <0.02 mol% stereoirregular monomeric units in the polymer); overall regioselectivity (*regio*_tot_, that is the summed mole fractions of monomer insertions with 2,1 or 3,1 enchainments) between 0.9859 and 0.9991 (i.e., from 1.4 to 0.09 mol% regioirregular monomeric units); number-average polymer molar mass (*M*_n_) between 10 and 1400 kDa. Two catalysts however produced poorly stereoregular oligomers for which neither stereo- nor regioselectivity could not be determined. The table also includes the melting temperature of the polymers measured by DSC. To the best of our knowledge, this database for the catalyst class of interest is unprecedented in terms of quality and robustness. 

### 3.1. Experimental Trends and Hints for Descriptors

In the following, we highlight some experimental structure-property trends and correlations thereof with structural features of the catalyst precursors, calculated by Density Functional Theory (DFT). This qualitative survey was used to identify chemically intuitive descriptors for a ‘clear-box’ QSAR model (as will be explained in a following Section). The ‘classical’ catalyst precursor **M2** is used as a reference for comparative purposes.

#### 3.1.1. Stereoselectivity

As is well-known, this property depends primarily on sterics and electronic effects can only have an indirect, albeit potentially devastating, impact in case they trigger the side process of growing chain epimerization [29,30,52,53,54,55]. When the popular ‘quadrant representation’ is adopted [91], it has been observed that maximizing the separation of steric bulk between ‘open’ and ‘closed’ quadrants is beneficial for stereoselectivity. This must be kept in mind when planning ligand substitution patterns (and is indeed well-captured by QSAR models, as we shall see later on). 

**Figure 2:**
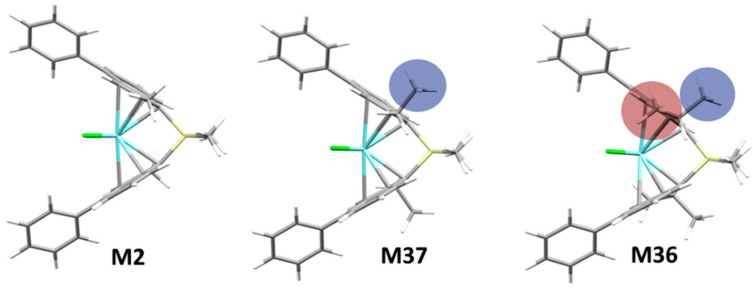
Increasing the steric demand of substituents at the 2-position negatively affects stereoselectivity if they intrude into the active pocket (see e.g., **M37** vs. **M36**). Stereoselectivity (*σ*): **M2** (2-Me) 0.9988 ≈ **M37** (2-Et) 0.9989 > **M36** (2-*i*Pr) 0.9720.

Historically, substituents at positions 2 and 4 of a *rac*-*ansa*-bis(1-Indenyl) ligand frame were demonstrated to be crucial on all properties of importance for catalyst performance [36,37,38]. From a survey of our database, we concluded that the steric demand of substituents at 2-position negatively affects stereoselectivity whenever the steric bulk points towards the active pocket (see e.g., **M36**, Figure 2). Substituting 2- *and* 3-positions together can be highly detrimental, examples are **M21** and **M28**, yielding poorly stereoregular propene oligomers. 

**Figure 3:**
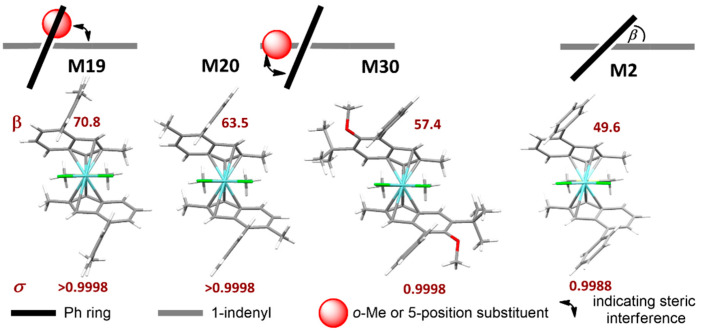
Effect of *ortho*-substituents of 4-Ph (4-*o*-Tolyl, **M19**), or substituents in the 5-position of the indenyl (**M20** and **M30**) on the dihedral angle (β) between the 4-Ph and the Indenyl plane, and consequent stereoselectivity *σ*. With increasing β the value of *σ* also increases, as separation of steric bulk between ‘open’ and ‘closed’ quadrants (see text) is perfected. **M2** data provided for comparison. Pictures generated with Mercury 4.2. Stylized sketches show upper indenyl ring (grey), phenyl substituent (black), and methyl or OMe substituent (red).

The key role of 4-position substituents is well-known from the previous literature [36,37]. When the substituent is a phenyl (Ph), in particular, our data indicate that increasing the dihedral angle (β) between the phenyl and the indenyl plane (Figure 3) leads to an increase in *σ*, as separation of steric bulk between the ‘open’ and ‘closed’ quadrants is perfected [13]. Two alternative strategies to achieve this are either substituting the 4-Ph moiety itself (e.g., in **M19**, bearing a 4-*o*-Tolyl substituent), or the 5-position of the indenyl thus introducing a steric clash with the 4-Ph (e.g., in **M20** (5-Me) and **M30** (5-OMe)). We will refer to the former as a soft conformational lock, to the latter instead as a hard conformational lock. The lock function can be on the rigid indenyl catalyst backbone (hard lock, e.g., 5-Me) or on a flexible substituent on this rigid backbone (soft lock, e.g., o-Toluyl).

Both can lead to catalysts with near-to-perfect stereoselectivity (≤0.02 mol% stereoirregular insertions), e.g., **M19** (soft lock) and **M20** (hard lock); preliminary evidence suggests that the latter imparts superior rigidity to the molecule, which is beneficial for high-temperature application [92].

Substitution of 6-position is inconsequential for stereoselectivity, unless very bulky substituents are used, e.g., mesityl in **M27**. Finally, a 7-methoxy substituent (**M29**) increases stereoselectivity when compared to the same substituent pattern without it (**M14**). Rieger et al. have argued on the basis of crystal structure analysis that: (a) the stereorigidity imparted by said methoxy substituent leads to lower values of ligand bite angle (α) [93] and Ph-Ind dihedral angle (β), and (b) this would be beneficial for the stereoselectivity (higher indeed for **M29** compared with **M2**) [94]. Gas phase DFT structures of a series of homologous precatalysts actually paint a different picture, and show that stereoselectivity primarily increases with increasing β (Table 2).

#### 3.1.2. Regioselectivity

Bulkier substituents in the 2-position like ethyl (**M37**) or *iso*propyl (**M36**) increase regioselectivity over that of **M2**, with **M36** showing the lowest amount of regioirregular monomeric units (0.09 mol%) in the entire polymer data set (Table 1 and Table 3). This is in line with earlier conclusions that metallocene regioselectivity is mainly dictated by steric effects [95,96].

We have shown earlier for catalysts **M1**–**M19** that perfecting the separation of steric bulk via tuning of the 4-position substituent enhances stereoselectivity and at the same time also benefits regioselectivity [13]. This is further confirmed by **M38** (4-(N-carbazolyl)) [60], showing high stereoselectivity and fairly high regioselectivity (0.20 mol% regioirregular monomeric units in the polymer).

Electron-donating substituents in the 5- or 6-positions are mostly detrimental to regioselectivity. As an example, 6-*tert-*butyl substitution more than doubles regioirregular monomeric units in the polymer produced. The effect, however, is small or even negligible for small substituents (see e.g., 5-OMe, by comparing **M30** and **M25**; or 6-Me, by comparing **M23** with **M2**). Electron-withdrawing 6-Cl substitution (**M35**) increases regioselectivity somewhat (0.36 mol% regioirregular units for **M35**, instead of 0.42 mol% for **M19**). The 6-position is fairly remote from the active pocket, and one might be tempted to rule out steric effects of its substitution on regioselectivity. However, the lack of a consistent trend for the various electron-donating substituents in the set (compare e.g., 6-Me to 6-*t*Bu having opposing effects) likely points to a subtle balance between steric and electronic effects even in this distant position. 7-OMe substitution slightly lowers regioselectivity (from 0.17 mol% regioirregular units in the polymer for **M14** to 0.23 mol% for **M29**). 

#### 3.1.3. Molar Mass Capability 

For the whole catalyst class of Figure 1 it has long been known that 2-Me substitution strongly increases average PP molecular weight [38]. This has been traced to a destabilization of the space-demanding 6-center TS of *β*-H transfer to the monomer [30,38], which is the dominant chain transfer pathway at least at moderate temperatures. Taking **M2** as a reference (2-Me; *M*_n_ = 320 kDa), larger alkyl groups in 2-position are detrimental in this respect, from moderately (**M37**, 2-Et; *M*_n_ 220 kDa) to very substantially (**M36**, 2-*i*Pr; *M*_n_ 19 kDa). The 3-position-substituted catalysts, in turn, yield essentially oligomers (see also SI, Figure S39).

Similar to regioselectivity, molar mass capability also increases when the distribution of steric bulk in the active pocket is perfected by 4-R substituent modulation [13]. **M38** (4-N-carbazolyl) confirms this further (*M*_n_ = 800 kDa). Introducing 5-position substituents approximately doubles molar mass capability of catalysts with a given substitution pattern. As shown before for stereoselectivity, this can be traced back to locking the 4-Ph substituent in a favorable β value. Any substituent in the 6-position increases molar mass capability, and the larger the substituent the higher the increase (Table 4). 

6-Iso-propyl and 6-*tert-*butyl show very similar effects, both orienting two methyl groups towards the active pocket. Nifant’ev has proposed that the 5-OMe and 6-*tert*-butyl substituents of **M30** lower the electrophilicity of the Zr center, thus increasing molar mass capability over that of **M2** [46]. However, looking at the data series in Table 4, it appears that—again—steric effects are dominant over electronic effects, as the charge on the ZrCl_2_-fragment in all examined pre-catalysts is similar (actually almost identical for **M23** (6-Me), **M24** (6-*i*Pr), and **M25** (6-*t*Bu)). 

Table 5 shows that introducing a 6-*tert*-butyl substituent into a given ligand has a near constant effect on PP *M*_n_ (1.8–2.7 fold increase) regardless of the remaining substituents. This is an interesting example of additive substituent effects for the catalyst class of interest.

### 3.2. QSAR Modeling

The next step then was the identification of a convenient set of descriptors in order to implement a well-working QSAR model. Whereas this step is nowadays routine in pharmaceutical chemistry [9], with several software packages and libraries of descriptors available commercially, examples in organometallic catalysis are still rare [97], likely because—as we noted above— ‘activity’ in the latter context indicates a whole set of performance properties that descend from different electronic and steric factors [10,11,12,13,84,98] and, moreover, can be difficult to quantify. In the following we define as a ‘clear-box’ QSAR model one which makes use of chemically intuitive descriptors with an evident meaning for the investigated systems (at odds with a ‘black-box’ QSAR model in which the descriptors are chosen tentatively out of very large sets, and selected for the best-working combination based on complex statistical procedures [9]). Out of the few literature clear-box QSAR studies in molecular olefin polymerization catalysis, the majority focused on the prediction of (relative) productivity in ethene or propene homopolymerization [10,12,99]. This property is in fact the nearest equivalent to ‘activity’ for pharma. At the same time however, it may not represent the best choice (at least in the context of interest here), considering the known sensitivity of most catalyst classes to: (a) the activation protocol (which determines the fraction of active metal) [100,101], (b) the invariable presence of adventitious (and ubiquitous) impurities, and (c) a variety of deactivation routes [102]. On the other hand, clear-box QSAR models focusing on microstructural polymer features, e.g., for propene homopolymers or ethene/1-alkene copolymers, can be counted with two hands, possibly due to the lack of adequate experimental data. Cavallo proposed a simple descriptor based on the volume that the ligand occupies around the active metal (so-called ‘buried volume’) in order to predict the stereoselectivity and polymer molar mass capability of prototypical propene polymerization catalysts belonging to different families [42,84,101,103]. In a recent study, we found that this descriptor is indeed effective for accurate and fine predictions of stereoselectivity for a limited set of Spaleck-type zirconocenes, provided that the radius of the sphere centered on the transition metal used in the calculations is properly optimized in order to account for all relevant substituent effects [13]. The possible relevance of remote substituents on catalyst molar mass capability was claimed by Cruz [11]. 

#### 3.2.1. QSAR Descriptors

**Figure 4:**
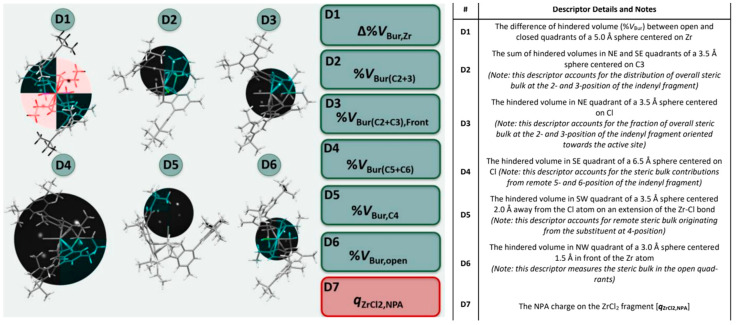
Computational descriptor pool for the *ansa*-zirconocenes of interest. 3D-steric descriptors D1–D6 were calculated using Cavallo’s SambVca 2.0 program. [84] Sphere position and size shown (black sphere), only quadrants with colored backbone are used for descriptor determination, all other quadrants are greyed out. D1–D4 and D6 shown so that the colored quadrant matches the description in the table. D5 has been rotated for clarity. (see SI, Tables S4 and S5 details on coordinate system definitions and construction of spheres and for further details).

Moving from these studies, we implemented a pool of seven descriptors, all related intuitively to simple electronic or steric properties of neutral LZrX_2_ precatalysts that are easy to quantify with DFT methods (Figure 4 and SI) using previously established protocols (see experimental section for details) [61,62,63,64,65,66,67,68,69,70,71,72,73,74,75,76,77,78,79,80,81,82,83]. The six steric descriptors screen different regions of space around the catalyst, that were selected based on well-established olefin insertion and chain transfer transition state structures (SI, Table S4). Descriptors provides the fraction of hindered volume in a quadrant of a sphere centered on a certain atom (descriptors D3–D6), the difference in the fraction of hindered volume between open and closed quadrants (descriptor D1), or the summed fractions of hindered volume of two quadrants (descriptor D2) (see Figure 4, Tables S4 and S5). Altogether, the descriptors screen the distribution of steric hindrance in the catalytic pocket, so that mathematical optimization can fine-tune the impact of each region. It should be noted here that Cavallo’s buried volume model [103] works best for prediction of stereoselectivity when the separation of steric bulk between the quadrants is analyzed, not when the overall buried volume in the sphere is considered. Our approach is an extension to this: different regions of space are analyzed using various spheres, which ultimately also allows to account for different influences from different regions. This would be impossible using a single sphere. The Natural Population Analysis (NPA [85]) charge on the ZrCl_2_ fragment turned out to perform satisfactorily as an electronic descriptor (D7).

#### 3.2.2. QSAR Models

**Figure 5:**
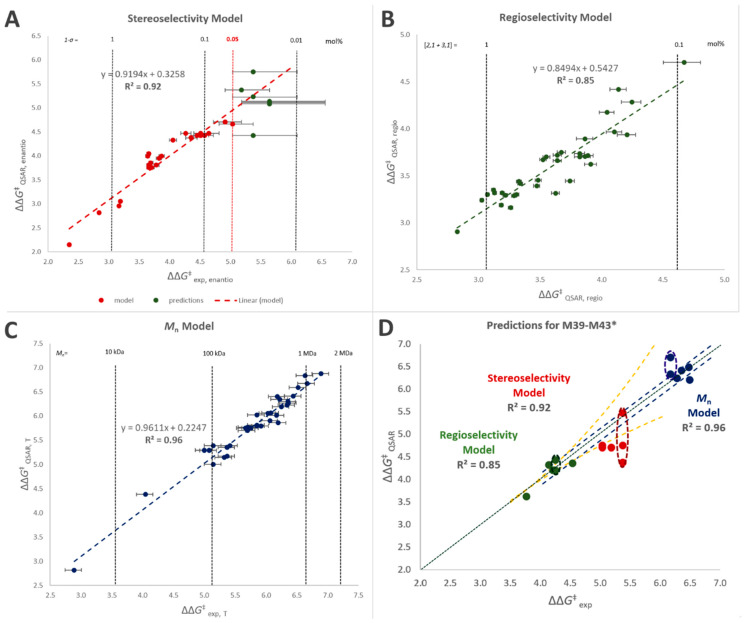
(**A**–**C**) QSAR-predicted vs. observed ΔΔ*G*^‡^ values (in kcal/mol) for the catalyst training set **M1**–**M38** (Figure 1 and Table 1): stereoselectivity (**A**); regioselectivity (**B**); and polymer molar mass capability (**C**). The model for stereoselectivity was built using the available experimental data for (1 − *σ*) ≥ 0.05% (see text). (**D**) QSAR-predicted vs. observed ΔΔ*G*^‡^ values for the catalyst validation set **M39**–**M43** (Figure 6 and Table 8): stereoselectivity (red dots); regioselectivity (green dots); and polymer molar mass capability (blue dots). Green dotted line = perfect agreement. In all cases, error bars (yellow dotted lines: for stereo- and regioselectivity, blue dotted lines for molar mass capability) reflect experimental uncertainty (see Table 1 and Table 8). * For **M43** two conformational isomers of comparable stability were identified by DFT modeling (see SI); the calculated values of all polymer properties of interest are reported for each isomer, inscribed into dashed ellipses.

Linear combinations of said descriptors (Equation (1)) were tested in order to reproduce the performance properties of interest (quantified in terms of ΔΔ*G*^‡^ between the relevant competing events) for the 38 catalysts in the training set of Figure 1. The quantitative mathematical formulations of the best-fit models are given in the SI (Equations (S4)–(S6)), whereas the results are shown in Figure 5A–C and Table 6. An overview of which descriptors contribute to each model and in what sense is provided in Table 7.

Stereoselectivity was satisfactorily reproduced as a function of descriptor D1 alone (Figure 5; *R*^2^ = 0.92), which is not surprising in view of the previous literature, although the calculation protocol had to be customized (SI, Figures S41–S43). This notwithstanding, modeling the experimental data was complicated because, as already noted above, the error bar on (1 − *σ*) explodes as soon as a catalyst approaches perfect stereoselectivity (Figure 5A). To solve this problem the model was trained on experimental values of *σ* ≤ 0.9995, i.e., (1 − *σ*) ≥ 0.05%.

Regioselectivity and polymer molar mass capability, on the other hand, required linear combinations of five descriptors (*R*^2^ = 0.85 and 0.96, respectively). This was also to be expected, considering that, unlike stereoselectivity, both properties: (a) can be influenced via all seven substituent positions on the indenyl fragment, (b) may result from various molecular kinetic paths, and (c) can be influenced by steric and electronic effects. 

The best-working model for regioselectivity does indeed make use of both electronic and steric descriptors, even though in agreement with previous computational studies the latter are dominating [98,104]. The best-working model for polymer molar mass capability, on the other hand, relies solely on steric descriptors. The outcome of key model assessment criteria, i.e., coefficient of determination (*R*^2^), adjusted coefficient of determination (adj.-*R*^2^) and cross validated *R*^2^ via leave-one-out analysis (*q*^2^), can be found in Table 6. Mean Average Deviation from Experiment (MAD) and Root Mean Square Error (RMSE) values are low (0.11–0.20 kcal/mol) for all models. Table 7 shows an overview of the impact of the different descriptors on each model. 

#### 3.2.3. Predictive QSAR Modeling

**Figure 6:**
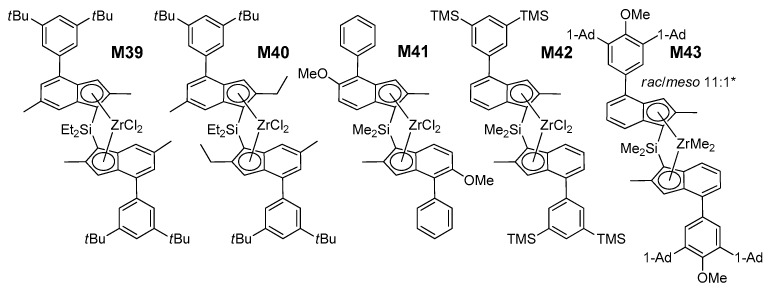
Structures of **M39–M43** (Ad = Adamantyl). * **M43** could not be further purified. The influence of varying the bridge from SiMe_2_ to SiEt_2_ is negligible within the experimental error margins [13].

Once the training stage was satisfactorily finalized, we applied the QSAR models to anticipate the catalytic properties of novel catalysts. About 30 structures were screened in-silico, and classified with respect to the predicted performance and the ease of preparation (SI, Tables S19 and S20). Based on such criteria, five (pre)catalysts (**M39–M43**; Figure 6) were selected, prepared, and tested experimentally under the same conditions of **M1–M38** (see SI for details). The results are summarized in Table 8. 

**Figure 7:**
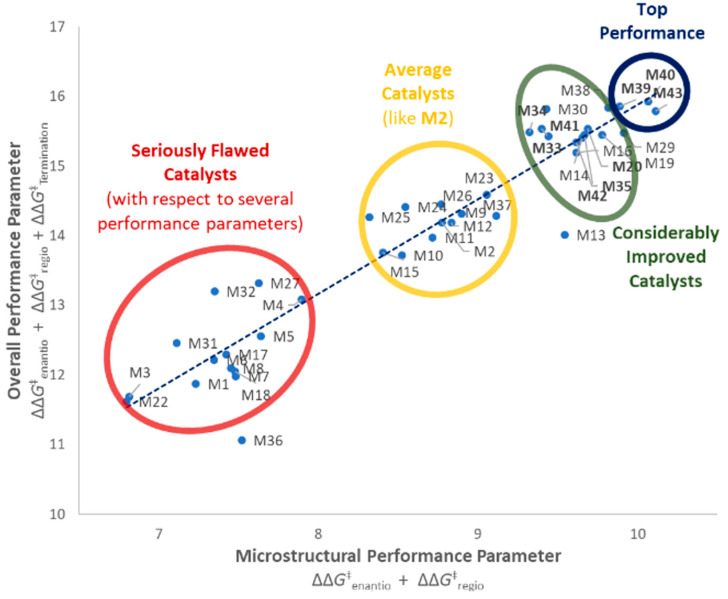
Performance balance of catalysts **M1**–**M43**. Blue trendline showing average trend of microstructural performance (stereo- and regioselectivity) vs. overall performance (including molar mass capability). Catalysts on the left of this line show higher molar mass capabilities than would be expected by this trend, catalysts on the right show lower ones. Three distinct catalyst groupings can be observed (seriously flawed to considerably improved). Nine out of 15 catalysts with considerable performance improvement (in bold) have been first reported in this manuscript, including the most balanced ones **M39**, **M40**, and **M43** (top performers).

All five turned out to perform remarkably well (see also Figure 7), and the agreement between experimental and QSAR-calculated properties is very nice (Figure 5D). **M40** in particular turned out to outperform all catalysts in the training set.

Compared to Spaleck’s archetype **M2**, it yielded i-PP samples with only 1/2 the amount of stereodefects (enantiodefects), 1/3 the amount of regiodefects, a 2-fold larger *M*_n_ value, and a two-fold higher productivity (with MAO activation, see SI, Table S3). Also with respect to the previous most balanced catalyst (**M14**) in Reference [13] a noticeable improvement in *T*_m_ is observed for **M40** (162.4 °C and 163.1 °C) and regioerrors are reduced by 1/3 to 0.11%. To the best of our knowledge, this *T*_m_ is the highest ever measured under the used polymerization conditions for this class of *ansa*-zirconocenes, and is indeed fairly close to that of ZNS i-PP.

## 4. Conclusions

In the mid-1980s, *C*_2_-symmetric *ansa*-bis(1-Indenyl) zirconocene catalysts represented the first compelling demonstration that highly isotactic-selective propene polymerization can be achieved with molecular catalysts, and even though several other classes of Group 4 *ansa*-metallocenes and post-metallocenes can now be used for that purpose they still represent the catalyst class of highest interest for industrial use. On the other hand, a higher cost-to-performance balance compared with classical ZNS has hampered large-scale application until now, and little progress in that respect has been reported in the last two decades.

In this paper, we have shown that state-of-the-art HTE-aided QSAR modeling can trigger new advances in the field. A structure-properties database of unprecedented size and robustness was rapidly assembled, and used to train and validate a clear-box QSAR model with predictive ability. The model utilizes, in particular, a limited number of chemically intuitive 3D geometric descriptors screening various regions of space in and around the catalytic pocket in a modular way that easily enables weighing and balancing the individual descriptor contributions to the overall catalytic performance. 

In our opinion, the proposed HTE/QSAR approach represents a valuable tool for catalyst optimization of rather general validity, of course with proper adaptations and the more so the more steric effects are dominating. In the meantime, application to the class of *ansa*-zirconocenes covered is continuing, and we will report major advances in the near future.

## Figures and Tables

**Table 1:** Results of the characterization of polypropylene (PP) samples prepared at *T*_p_ = 60 °C and *p*(C_3_H_6_) = 6.6 bar in toluene with the 38 *ansa*-zirconocene catalysts of Figure 1 (see text for details).

ID	Substituent Pattern	*Regio*_tot_^#^	1−*σ* ^≠^	*M*_n_ *	PDI **	*T*_m_^‡^
**M1 ^†^**	2-Me	0.29	1.35(3)	100	1.9	145.7
**M2 ^†^**	2-Me-4-Ph	0.32	0.12	320	2.0	160.2
**M3 ^†^**	2-Me-4-furyl	0.91	0.80	130	2.1	143.2
**M4 ^†^**	2-Me-4-thienyl	0.50	0.29	230	2.0	153.2
**M5 ^†^**	2-Me-4-Cl	0.53	0.40	150	2.0	151.7
**M6 ^†^**	2-Me-4-Br	0.82	0.40	140	1.9	148.9
**M7 ^†^**	2-Me-4-Me	0.67	0.40	80	2.1	148.9
**M8 ^†^**	2-Me-4-*i*Pr	0.74	0.38	100	2.1	149.7
**M9 ^†^**	2-Me-4-(4-F-Ph)	0.29	0.11	320	2.1	157.3
**M10 ^†^**	2-Me-4-(4-Me-Ph)	0.40	0.14	230	2.4	157.6
**M11 ^†^**	2-Me-4-(4-*t*Bu-Ph)	0.30	0.14	250	2.2	159.3
**M12 ^†^**	2-Me-4-(4-CF_3_-Ph)	0.32	0.11	290	2.4	156.9
**M13 ^†^**	2-Me-4-mesityl	0.40	0.03	80	2.2	155.3
**M14 ^†^**	2-Me-4-(3,5-*t*Bu-Ph)	0.17	0.06	530	2.2	162.4
**M15 ^†^**	2-Me-4-(3,5-F-Ph)	0.42	0.16	290	2.3	157.2
**M16 ^†^**	2-Me-4-C_6_F_5_	0.18	0.06	410	2.2	160.8
**M17 ^†^**	2-Me-4-Br, SiEt_2_-bridge	0.79	0.37	140	2.2	149.0
**M18 ^†^**	2-Me-4-Me, SiEt_2_-bridge	0.66	0.41	90	2.3	149.3
**M19 ^†^**	2-Me-4-*o*-tolyl	0.42	<0.02	470	1.8	158.2
**M20**	2,5-Me-4-Ph	0.48	<0.02	610	2.1	158.2
**M21**	2,3-Me-4-Ph	N/A	N/A	1.0	2.2	N/A
**M22**	2-Me-2-furyl, SiEt_2_-bridge	0.90	0.83	130	1.3	143.2
**M23**	2,6-Me-4-Ph	0.28	0.09	380	2.2	159.7
**M24**	2-Me-4-Ph-6-*i*Pr	0.54	0.10	630	2.3	156.1
**M25**	2-Me-4-Ph-6-*t*Bu	0.69	0.11	710	2.4	153.4
**M26**	2-Me-4,6-Ph	0.43	0.09	480	2.0	158.2
**M27**	2-Me-4-Ph-6-mesityl	0.98	0.22	490	2.2	148.4
**M28**	2,3-Me-6-tBu	N/A	N/A	3	1.8	N/A
**M29**	2-Me-4-(3,5-*t*Bu-Ph)-7-OMe	0.23	0.03	400	2.2	162.2
**M30**	2-Me-4-Ph-5-OMe-6-*t*Bu	0.71	0.02	1400	2.3	156.0
**M31**	2-Me-4-Br-6-*t*Bu	1.41	0.33	290	2.0	143.0
**M32**	2-Me-4-thienyl-6-*t*Bu	1.05	0.31	620	2.3	148.0
**M33**	2-Me-4-(3,5-tBu-Ph)-6-*t*Bu	0.30(3)	0.05	950	2.3	160.6
**M34**	2-Me-4-*o*-tolyl-6-tBu	0.83	<0.02	990	2.3	154.3
**M35**	2-Me-4-*o*-tolyl-6-Cl	0.36	0.03	510	2.3	159.7
**M36**	2-*i*Pr-4-Ph	0.09	2.80(3)	19	2.3	137.2
**M37**	2-Et-4-Ph	0.21	0.11	220	2.1	160.5
**M38**	2-Me-4-(N-carbazolyl)	0.20	0.04	800	2.3	161.5

Experimental conditions: *T*_p_ = 60 °C, toluene solvent, *p*(C_3_H_6_) = 6.6 bar, TIBA/AB activation (for abbreviations see text). Experimental uncertainty is ±2 on last significant digit for 1−*σ* and *regio*_tot_, unless otherwise indicated in parentheses; ±20% on *M*_n_. For more details see Supplementary Information (SI), Tables S1 and S2. ^†^ Data taken from Reference [13] ^#^ Total fraction of 2,1 and 3,1 monomeric units in % (^13^C NMR). *^≠^* Fraction of stereoirregular monomeric units in % (^13^C NMR), according to the enantiomorphic-site statistical model [29]. * In kDa. ** PDI = Poly-Dispersity Index (*M*_w_/*M*_n_). ^‡^ In °C. N/A: not available (the catalyst produced oligomers under the used conditions).

**Table 2:** Density Functional Theory (DFT)-calculated values of α and β angles (see text) vs. experimentally observed stereoselectivity *σ* for selected catalysts.

Catalyst	α (°) *	β (°)	1−*σ ^≠^*
**M2**	60.5	49.6	0.12
**M14**	60.5	51.1	0.06
**M29**	60.2	52.8	0.03
**M30**	60.9	56.1	0.02
**M20**	60.9	63.5	<0.02
**M19**	60.8	70.8	<0.02

* Bite angle or interplanar ring angle (α): angle formed by the two planes defined by the five carbon atoms of the C_5_ fragments of the indenyl rings [93]. ^≠^ Fraction of stereoirregular monomeric units in % (^13^C NMR), according to the enantiomorphic-site statistical model [29].

**Table 3:** 2-R substituent size vs. catalyst regioselectivity.

Catalyst	2-R Substituent	*Regio*_tot_^≠^
**M2**	Me	0.32
**M37**	Et	0.21
**M36**	iPr	0.09

^≠^ Fraction of stereoirregular monomeric units in % (^13^C NMR), according to the enantiomorphic-site statistical model [29].

**Table 4:** Effect of the 6-position substituents on the charge on the ZrCl_2_ fragment (see text) and PP *M*_n_ (in kDa). For all catalysts 4-R = Ph.

Catalyst	Substituent	*M*_n_	*e* ^−^_ZrCl2, NPA_	*M*_n_ Increase Factor
**M2**	H	320	0.412	-
**M23**	Me	380	0.405	1.2
**M26**	Ph	480	0.412	1.5
**M24**	iPr	630	0.404	2.0
**M25**	tBu	710	0.403	2.2
**M30**	tBu + 5-OMe	1400	0.400	4.4

**Table 5:** Effect of a 6-*t*Bu substituent on PP *M*_n_ (in kDa).

Catalyst Pair	*M*_n_ without 6-*t*Bu	*M*_n_ with 6-*t*Bu	Increase Factor
**M2**/**M25**	320 (**M2**)	710 (**M25**)	2.2
**M4**/**M32**	230 (**M4**)	620 (**M32**)	2.7
**M6**/**M31**	140 (**M6**)	290 (**M31**)	2.1
**M14**/**M33**	530 (**M14**)	950 (**M33**)	1.8
**M19**/**M34**	470 (**M19**)	990 (**M34**)	2.1

**Table 6:** Key QSAR model assessment criteria.

	1−*σ*	*Regio*_tot_	*M*_n_ (kDa)
**Data range (DR)**	2.8–0.05	1.41–0.09	3–1400
**DR in kcal/mol**	3.75	1.85	4.00
***R*^2^/adj. *R*^2^**	0.92/0.92	0.85/0.82	0.96/0.95
**max. *p*-value**	4 × 10^−8^	2 × 10^−3^	1 × 10^−4^
***q*^2^**	0.90	0.77	0.83
**MAD (kcal/mol)**	0.15	0.13	0.11
**MAD (1-*σ*/*regio*_tot_/*M*_n_)**	0.10	0.10	58 kDa
**RMSE (kcal/mol)**	0.18	0.16	0.20

**Table 7:** List of used descriptors and their impact in the QSAR models for stereoselectivity, regioselectivity, and polymer molar mass capability.

#	Regio	Stereo	*M*_n_
**D1**	×	+	+
**D2**	+	×	×
**D3**	×	×	−
**D4**	−	×	+
**D5**	+	×	+
**D6**	−	×	−
**D7**	−	×	×

+ = used in the linear combination with positive weight. − = used in the linear combination with negative weight. × = not used in the model.

**Table 8:** Predicted/observed (in bold) performance properties for catalysts **M39–M43**.

QSAR/Exp.	1−*σ* (%)	*Regio*_tot_ (%)	*M*_n_ (kDa)	*T*_m_ (°C)
**M39**	0.08/0.04	0.17/0.18	760/740	-/162.2
**M40**	0.08/0.05	0.14/0.11	680/620	-/163.1
**M41**	0.08/0.04	0.42/0.35	490/760	-/159.7
**M42**	0.08/0.05	0.14/0.20	520/550	-/161.6
**M43**	0.02–0.12 ^a^/0.03	0.12–0.15 ^a^/0.17	1060–490 ^a^/470	-/162.6

^a^ The two pairs of QSAR-predicted values refer to two different precatalyst conformers identified by DFT, featuring the 1-Ad groups either oriented towards the active pocket or away from it, due to steric repulsion between 4-OMe and 3,5-Ad.

## Supplementary Materials

The following are available online at https://www.mdpi.com/2073-4360/12/5/1005/s1. Details for the synthesis of **M20**-**M28**, **M31**-**M36,** and **M39-M43**, including Figures S1–S38 with ^1^H-NMR (odd numbers) and ^13^C-NMR (even numbers). Additional references relevant for synthesis [58,105,106,107,108,109,110,111,112,113,114,115,116]. Detailed polymerization procedure and results (Table S1: Propene Polymerization Experiments, Table S2: Full Polymer Characterization, Table S3: Performance of selected catalysts when activated with MAO). Figure S39: Experimental Trends - Deleterious Effect of the 3-Position Substituents on Molar Mass Capability. QSAR model details (Table S4: Descriptor list and reasons for inclusion of the descriptor in the descriptor pool, Figure S40: Differences between propene 1,2 and 2,1 insertion TS for **M39**, Table S5: Procedures for Descriptor Determination). QSAR models (Equations (S1)–(S6), Table S6: Experimental data used for QSAR modeling, Figure S41: Rationale for increased scanning sphere size to determine %*V*_Bur,Zr_ on the example of **M2**, Figure S42: Rationale for deletion of atoms or groups in the 5-, 6-, and 7-position and the Si-bridge to determine %*V*_Bur,Zr_, Figure S43: Exclusion of atoms of the 2-position substituents not in line-of-sight of the active center for calculation of %V_Bur,Zr_). QSAR model details, analysis of variance for QSAR models, leave-one-out analysis for QSAR models (Tables S7–S18), QSAR screening and synthesis candidates (Table S19: Predicted performance for selected catalysts, including **M39**–**M43** and Table S20: Estimated synthetic complexity, including **M39**–**M43**). Full Gaussian citation. (PDF) LMCl_2_ structures (XYZ).
