# Supplementary material for: An Integrated High Throughput Experimentation/Predictive QSAR Modeling Approach to ansa-Zirconocene Catalysts for Isotactic Polypropylene
Source: Polymers (Basel). 2020 Apr 27;12(5):1005. doi: 10.3390/polym12051005 (PMC7284373; doi:10.3390/polym12051005)
Supplement: Supplementary file 1 [file polymers-12-01005-s001.zip › SI.pdf]

SUPPORTING INFORMATION

An Integrated High Throughput Experimentation / Predictive QSAR Modeling Approach to *ansa*-Zirconocene Catalysts for i-PP

Christian Ehm,[a,] Antonio Vittoria,[a] Georgy P. Goryunov,[b] Vyatcheslav V. Izmer,[b] Dmitry S. Kononovich,[b]Oleg V. Samsonov,[b] Rocco Di Girolamo,[a] Peter H. M. Budzelaar,[a] Alexander Z. Voskoboynikov,[b] Vincenzo Busico,[a] Dmitry V. Uborsky[b,*] and Roberta Cipullo[a,*]*

[a] Dipartimento di Scienze Chimiche, Università di Napoli Federico II, Via Cintia, 80126 Napoli (Italy); DPI, P.O. Box 902, 5600 AX Eindhoven, the Netherlands.

[b] Department of Chemistry, Lomonosov Moscow State University, 1/3 Leninskie Gory, 119991 Moscow (Russia); DPI, P.O. Box 902, 5600 AX Eindhoven, the Netherlands.

Contents

1. Synthesis of the Pre-catalysts ... 3
General Comments ... 3
M20 ... 3
M21 ... 5
M22 ... 8
M23 ... 10
M24 ... 13
M25 ... 16
M27 ... 20
M28 ... 24
M31 ... 26
M32 ... 28
M33 ... 30
M34 ... 33
M35 ... 36
M36 ... 38
M39 ... 40
M40 ... 42
M41 ... 47
M42 ... 50
M43 ... 52
References ... 55
2. Polymerization Details ... 56
Table S1. Propene Polymerization Experiments. ... 57
Table S1, ctd. Propene Polymerization Experiments. ... 58
Table S2. Full Polymer Characterization. ... 59
Table S2, ctd. Full Polymer Characterization. ... 60
Table S3. Performance of selected catalysts when activated with MAO. ... 61
Experimental Trends -Deleterious Effect of 3-Position Substituents on Molar Mass Capability ... 61
3. QSAR Modeling ... 62
QSAR Modeling – General Comments ... 62
QSAR Modeling – Descriptor Pool ... 63
QSAR Modeling – Determination of Gibbs Free Energy Differences ... 66
QSAR Modeling – $\%V_{Bur,Zr}$ determination ... 69
QSAR Modeling – Equations for Stereo-, Regioselectivity and Molecular Weight Capability Models ... 71
QSAR Modeling – Stereoselectivity Model ... 72
QSAR Modeling – Stereoselectivity Model, Analysis of Variance ... 75
QSAR Modeling – Stereoselectivity Model, Leave-One-Out Analysis ... 76
QSAR Modeling – Molar Mass Capability Model, Single Descriptor Model ... 77
QSAR Modeling – Molar Mass Capability Model, Final Model ... 78
QSAR Modeling – Molar Mass Capability Model, Analysis of Variance ... 80
QSAR Modeling – Molar Mass Capability Model, Leave-One-Out Analysis ... 81
QSAR Modeling – Regioselectivity, Final Model ... 82
QSAR Modeling – Regioselectivity Model, Analysis of Variance ... 84
QSAR Modeling – Regioselectivity Model, Leave-One-Out Analysis ... 85
QSAR Modeling – Screened Catalyst Candidates and Predicted Performance ... 86
4. Full Gaussian Citation ... 88

1. Synthesis of the Pre-catalysts

General Comments

All manipulations with compounds which are sensitive to moisture and air were performed either in an atmosphere of purified argon using a standard Schlenk technique or in a controlled atmosphere glove box (MBraun). Bis(2,5-dimethyl-4-phenyl-1*H*-inden-1-yl)dimethylsilane [1], 4-bromo-2,6-dimethyl-1-methoxyindane [2], 4-bromo-1-methoxy-6-isopropyl-2-methylindane and 2,6-dimethyl-4-(3,5-di-*tert*-butylphenyl)-1*H*-indene [3], 4-bromo-1-methoxy-6-*tert*-butyl-2-methylindan and 5-*tert*-butyl-7-(3,5-di-*tert*-butylphenyl)-2-methyl-1*H*-indene [4], 7-bromo-5-*tert*-butyl-2-methyl-1*H*-indene [5], 2-methyl-4-bromo-6-chloro-1*H*-indene [6];metallocenes M26 [7], M29 [8], M30 [9], M37 [10], M38 [11]; $NiCl_2(PPh_3)IPr$ [12], $[PhN(CH_2)_3NPh]ZrCl_2(THF)_2$[13], (5-bromo-1,3-phenylene)bis(trimethylsilane) [14], 6-methoxy-2-methyl-2,3-dihydro-1H-inden-1-one [15] were synthesized according to the published procedures. Tetrahydrofuran and diethyl ether were distilled from sodium benzophenone ketyl. Hydrocarbon solvents were degassed by bubbling with argon and dried over molecular sieves 4A. Dichloromethane-d_2, chloroform-*d* and benzene-d_6 were freeze-pump-thaw degassed on a vacuum line and dried over molecular sieves 3Å. ^{1}H and ^{13}C{^{1}H} spectra were recorded with Bruker Avance-400 spectrometers for 1–10% solutions in deuterated solvents. Chemical shifts for ^{1}H and ^{13}C were measured relatively to TMS or deuterated solvent residual signals. C, H microanalyses were done using Vario Micro Cube analyzer. HRMS spectra were measured using Orbitrap Elite instrument.

M20

rac-dimethylsilanediyl-bis(η^5-2,5-dimethyl-4-phenyl-inden-1-yl)dimethylzirconium (M20)

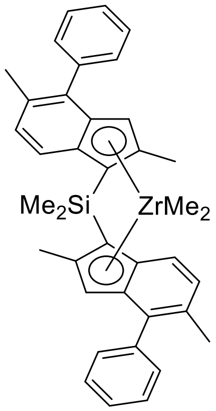

To a cooled to –40 °C solution of 4.80 g (9.66 mmol) of bis(2,5-dimethyl-4-phenyl-1*H*-inden-1-yl)dimethylsilane in 200 ml of ether 7.70 ml (19.3 mmol) of 2.5 M ^{n}BuLi in hexanes was added in one portion. This mixture was stirred overnight at room temperature. The resulting light-yellow solution was cooled to -80 °C, and 3.64 g (9.66 mmol) of $ZrCl_4(THF)_2$ was added. The reaction mixture was stirred for 24 h, then evaporated to dryness. The residue was heated with 250 ml of toluene, and the formed hot suspension was filtered through glass frit (G4). The filtrate was evaporated to dryness giving 2.90 g of a 1:1 mixture of *rac*- and *meso*-complexes. Further on, 1.20 g (1.83 mmol) of this crude mixture was dissolved in 100 ml of toluene, and 2.03 ml (5.5 mmol, 2.7 M) of MeMgBr in diethyl ether was added. The reaction mixture was stirred at 90 °C overnight and then evaporated to dryness. The crude product was extracted with 2 x 50 ml of hot toluene, the combined extract was filtered through a thin pad of Celite 503, then the filtrate was evaporated to dryness yielding 870 mg of a 20:1 mixture of *meso*- and *rac*-dimethylsilanediylbis[η^5-2,5-dimethyl-4-phenyl-inden-1-yl]dimethylzirconium. This product was dissolved in 50 ml of THF, and 10 mg of LiCl was added. The reaction mixture was stirred overnight at 50 °C, then evaporated to dryness. The resulting sticky solids were extracted with 2 x 50 ml of hot methylcyclohexane. The combined extract was filtered through a thin pad of Celite 503, and the filtrate was evaporated to dryness. Thus obtained solid was recrystallized three times from hexane yielding 90 mg (1.5%) of analytically pure racemic $ZrMe_2$-complex as a light-yellow powder. *Rac*-zirconocene. Anal. calc. for $C_{38}H_{40}SiZr$: C, 74.09; H, 6.54. Found: C, 74.28; H, 6.70. ^{1}H NMR ($CDCl_3$): δ 7.47–7.30 (m, 10H), 7.39 (d, *J* = 8.9 Hz, 2H), 6.89 (d, *J* = 8.9 Hz, 2H), 6.44 (br.s, 2H), 2.26 (s, 6H), 2.05

(s, 6H), 1.07 (s, 6H), –1.19 (s, 6H).$^{13}C\{^1H\}$ NMR (C_6D_6): δ 140.1, 136.0, 134.4, 131.62, 131.61, 127.4, 126.0, 124.7, 116.3, 78.5, 35.5, 19.9, 18.2, 2.4.

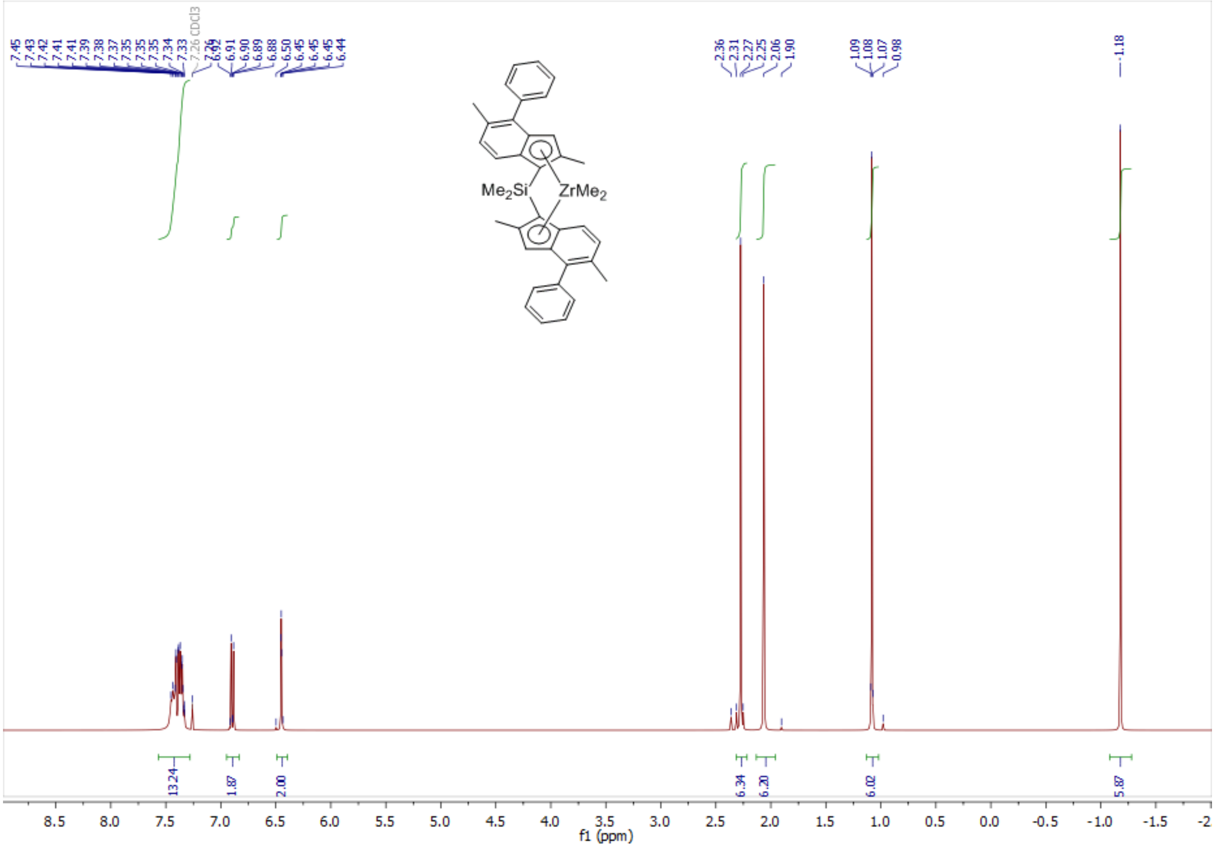

Figure S1. ^{1}H NMR spectrum of **M20** in $CDCl_3$.

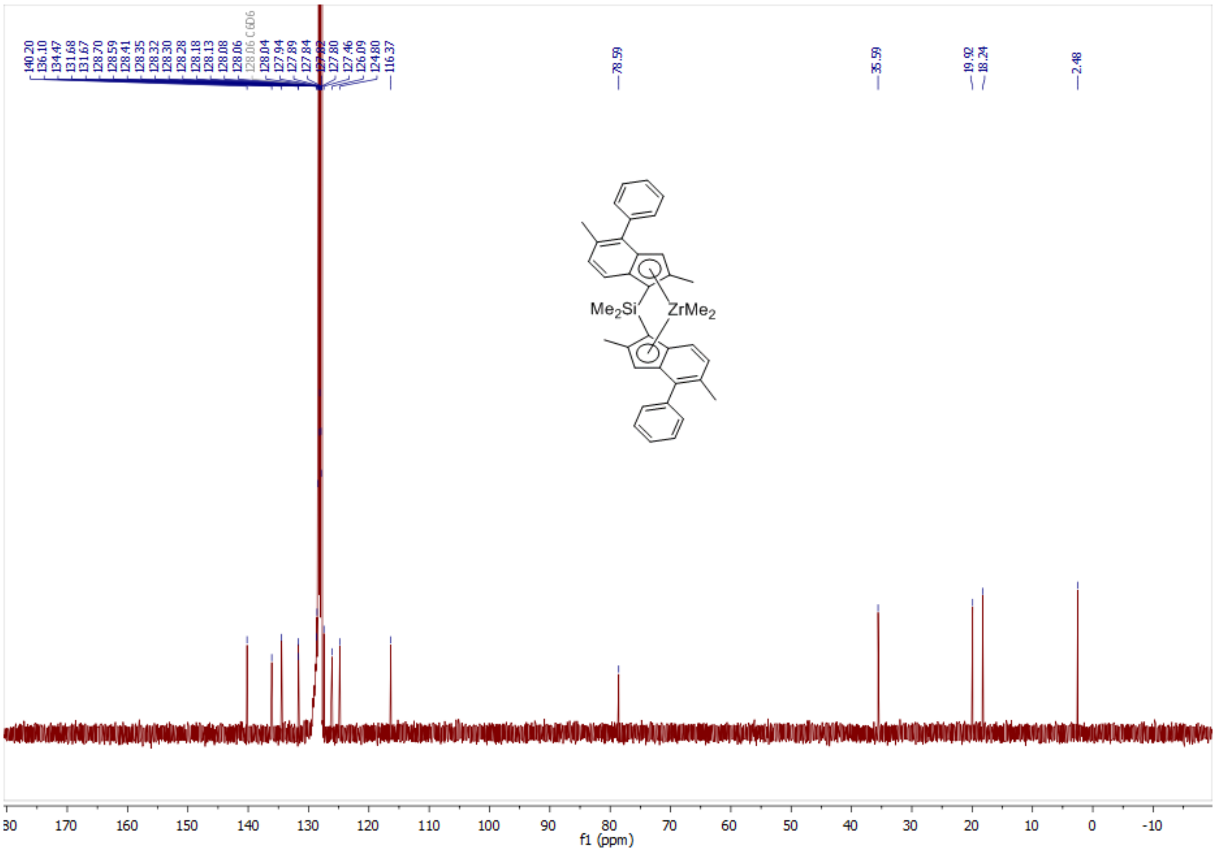

Figure S2.$^{13}C\{^1H\}$ NMR spectrum of **M20** in C_6D_6.

M21

4-bromo-2,3-dimethyl-2,3-dihydro-1H-inden-1-one

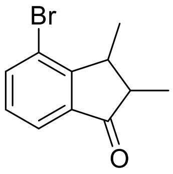

To a solution of sodium ethoxide obtained from 17.0 g (740 mmol) of sodium and 500 ml of anhydrous ethanol 125 g (700 mmol) of diethyl methylmalonate was added. This solution was stirred for 10 min, and then 186 g (700 mmol) of 1-bromo-2-(1-bromoethyl)benzene was added dropwise for 15 min. The resulting mixture was refluxed for 4 h, and then a solution of 163 g (2.90 mol) of KOH in 500 ml of water was added. This mixture was refluxed for 4 h, then ethanol was distilled off under atmospheric pressure. The resulting solution was acidified to pH 1-2 by 12 N HCl, and then crude substituted malonic acid was extracted by 3 x 300 ml of ethyl acetate and 200 ml of ether. The combined organic extract was washed by 3 x 500 ml of water, dried over Na_2SO_4, and then evaporated to dryness. Decarboxylation of the residue at 180-200°C gave the respective carboxylic acid which was then cooled to room temperature and treated with 180 ml (2.4 mol) of thionyl chloride. The resulting mixture was stirred overnight, and then an excess of thionyl chloride was distilled off. Vacuum rectification of the residue gave 146 g of 3-(2-bromophenyl)-2-methylbutanoyl chloride, b.p. 131°C/2 mbar. Further on, a solution of this product in 400 ml of dichloromethane was added to a suspension of 88.0 g (660 mmol) of $AlCl_3$ in 360 ml of dichloromethane at -10°C. This mixture was stirred overnight at room temperature and then poured on 1000 cm^3 of ice. The organic layer was separated, the aqueous layer was extracted by 3 x 200 ml of dichloromethane. The combined organic extract was washed by 3 x 300 ml of water, dried over Na_2SO_4, and then evaporated to dryness. The residue was purified by flash chromatography on silica gel 60 (40-63 µm, eluent: dichloromethane). This procedure

gave 93.0 g (56%) of the product as a yellowish oil. According to ^{1}H NMR, the product was a mixture of two diastereomers, A and B, in ~2:1 molar ratio.^{1}H NMR ($CDCl_3$): δ 7.75 (d, J = 7.6 Hz, 1H in A), 7.72 (d, J = 7.7 Hz, 1H in B), 7.69–7.66 (m, 2H in A and B), 7.25 (m, 1H in A), 7.24 (m, 1H in B), 3.56 (m, 1H in B), 3.04 (qd, J = 7.0 Hz, J = 2.1 Hz, 1H in A), 2.83 (m, 1H in B), 2.36 (qd, J = 7.4 Hz, J = 2.1 Hz, 1H in A), 1.46 (d, J = 7.0 Hz, 3H in A), 1.27 (d, J = 7.4 Hz, 3H in A), 1.26 (d, J = 7.2 Hz, 3H in B), 1.19 (d, J = 7.0 Hz, 3H in B).

4-bromo-2,3-dimethyl-1H-indene

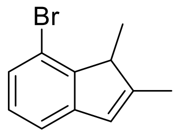

To a suspension of 4.10 g (105 mmol) of $NaBH_4$ in 60 ml of methanol 17.2 g (72 mmol) of 4-bromo-2,3-dimethyl-2,3-dihydro-1*H*-inden-1-one was added at room temperature. The resulting mixture was stirred overnight at room temperature, then poured into 200 ml of water. Crude product was extracted with 3 x 50 ml of ethyl acetate, the combined extract was dried over Na_2SO_4 and then evaporated to dryness. The residue was dissolved in 100 ml of toluene, the obtained solution was added in one portion to a hot solution of 0.1 g of TsOH in 400 ml of toluene. The reaction mixture was refluxed for one minute and then poured into 300 ml of aqueous Na_2CO_3. The organic layer was separated, dried over Na_2SO_4, and evaporated to dryness. The product was isolated by flash chromatography on silica gel 60 (40-63 µm, eluent: hexane). This procedure gave 14.7 g (92%) of the product as a colorless oil. ^{1}HNMR ($CDCl_3$): δ 7.21 (d, J = 7.8 Hz, 1H), 7.15 (d, J = 7.3 Hz, 1H), 7.06 (m, 1H), 6.36 (m, 1H), 3.32 (q, J = 7.4 Hz, 1 H), 2.06 (d, J = 0.9 Hz, 3H), 1.41 (d, J = 7.4 Hz, 3H).

2,3-dimethyl-4-phenyl-1H-indene

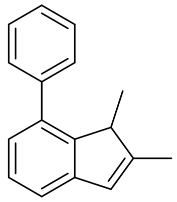

To a solution of 3.90 g (17.5mmol) of 4-bromo-2,3-dimethyl-1H-indene in 100 ml of 1,4-dioxane 3.20 g (26.2mmol) of phenylboronic acid, 14.3 g (43.7 mmol) of caesium carbonate, and 50 ml of water were subsequently added. The mixture obtained was purged with argon for 10 min followed by addition of 605mg (0.523mmol) of $Pd(PPh_3)_4$. This mixture was stirred for 12 h at 90°C, then cooled to room temperature, and diluted with 50 ml of water. The obtained mixture was extracted with dichloromethane (3 x 100 ml). The combined organic extract was dried over Na_2SO_4 and then evaporated to dryness. The residue was purified by flash chromatography on silica gel 60 (40-63 µm, eluent: hexane). Yield 3.60 g (94%) of the product as a colorless oil. ^{1}H NMR ($CDCl_3$): δ 7.39–7.30 (m, 6H), 7.14–7.04 (m, 2H), 3.30 (br.s, 2H), 2.01 (m, 3H), 1.50–1.45 (m, 3H).

bis(2,3-dimethyl-4-phenyl-1H-inden-1-yl)dimethylsilane

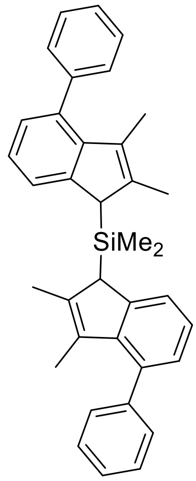

To a solution of 3.50 g (16.0mmol) of 2,3-dimethyl-4-phenyl-1*H*-indene in 100 ml of ether 6.40 ml (16.0mmol) of 2.5 M ^{n}BuLi in hexanes was added in one portion at -40 °C. This mixture was stirred overnight at room temperature, then the resulting light-yellow solution was cooled to -80°C, and 50 mg of *N*-methylimidazole was added. The resulting mixture was stirred for 5 min at -80 °C, then 1.03 g (8.00 mmol) of dichlorodimethylsilane was added in one portion. Further on, this mixture was stirred overnight at ambient temperature, then filtered through a short pad of silica gel 60 (40-63 µm) which was additionally washed by 2 x 30 ml of dichloromethane. The combined filtrate was evaporated under reduced pressure, and the residue was purified by flash chromatography on silica gel 60 (40-63 µm, eluent: hexane:dichloromethane; 10:1, vol.). Yield 2.30 g (58%) of the product as an orange glassy solid. According to 1H NMR spectrum, the product was a mixture of *rac*- and *meso*-isomers in ca. 1:1 molar ratio. Anal. calc. for $C_{36}H_{36}Si$: C, 87.04; H, 7.30. Found: C, 86.63; H, 7.36.^{1}H NMR

($CDCl_3$): δ7.47–7.24 (m, 24H in *rac* and *meso*), 7.14–7.09 (m, 8H in *rac* and *meso*), 3.71 (s, 2H in *rac* or *meso*), 3.68 (s, 2H in *meso* or *rac*), 2.11 (br.s, 6H in *rac* or *meso*), 2.06 (br.s, 12H in *rac* and *meso*), 1.56 (m, 12H in *rac* and *meso*), –0.21 (s, 3H in *meso*), –0.29 (s, 6H in *rac*), –0.33 (s, 3H in *meso*).

***rac*-dimethylsilanediylbis[η^5-2,3-dimethyl-4-phenyl-inden-1-yl]zirconium dichloride (M21)**

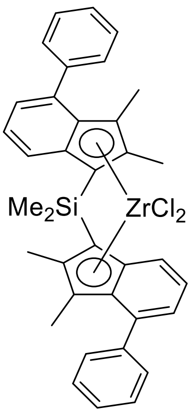

To a cooled to –40 °C solution of 2.30 g (4.63 mmol) of bis(4-phenyl-2,3-dimethyl-1*H*-inden-1-yl)dimethylsilane in 150 ml of ether 3.70 ml (9.26 mmol) of 2.5 M ^{n}BuLi in hexanes was added in one portion. This mixture was stirred overnight at room temperature, then cooled to -80 °C, and 1.75 g (4.63 mmol) of $ZrCl_4(THF)_2$ was added. The reaction mixture was stirred for 24 h, and then evaporated to dryness. The residue was heated with 150 ml of toluene, and the formed hot suspension was filtered through glass frit (G4). The filtrate was evaporated to dryness. This procedure gave 1.57 g of a 2:1 mixture of *rac*- and *meso*-complexes. This crude product was recrystallized for three times from methylcyclohexane/toluene mixture (1:1, vol.) yielding 65 mg (2.1%) of *rac*-complex as an orange powder. *Rac*-zirconocene. Anal. calc. for $C_{36}H_{34}Cl_2SiZr$: C, 65.83; H, 5.22. Found: C, 65.97; H, 5.45. ^{1}H NMR ($CDCl_3$): δ 7.62 (dd, *J* = 8.8 Hz, *J* = 0.9 Hz, 2H),7.62–7.57 (m, 2H), 7.42–7.31 (m, 8H), 7.11 (dd, *J* = 6.8, *J* = 0.9 Hz, 2H), 6.97 (dd, *J* = 8.8, *J* = 6.8 Hz, 2H), 2.02 (s, 6H), 1.69 (s, 6H), 1.36 (s, 6H).^{13}C{^{1}H} NMR (C_6D_6): δ141.1, 139.9, 133.7, 131.5, 129.7, 128.6, 127.7, 127.6, 124.8, 124.6, 79.6, 15.8, 14.4, 3.8.

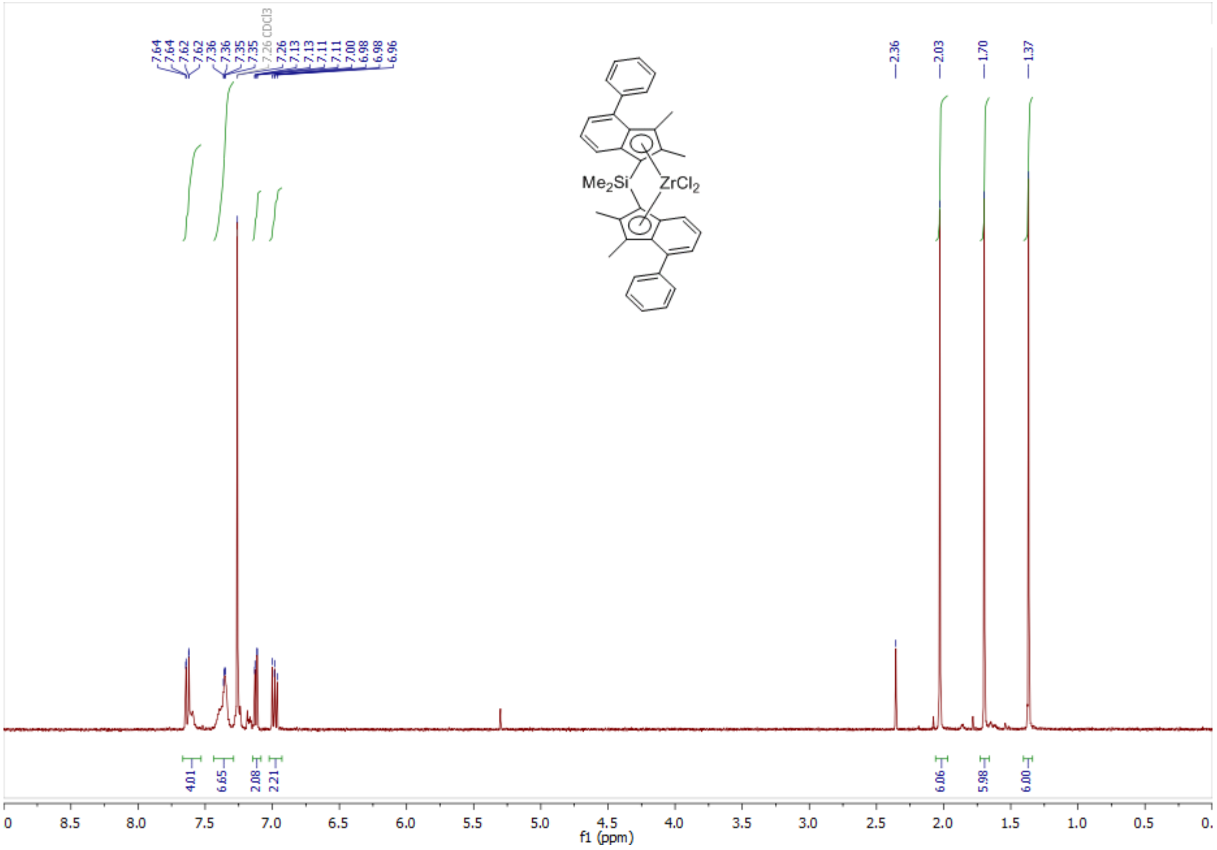

Figure S3. ^{1}H NMR spectrum of **M21** in $CDCl_3$. Traces of toluene are visible at 2.36 and 7.13–7.26 ppm.

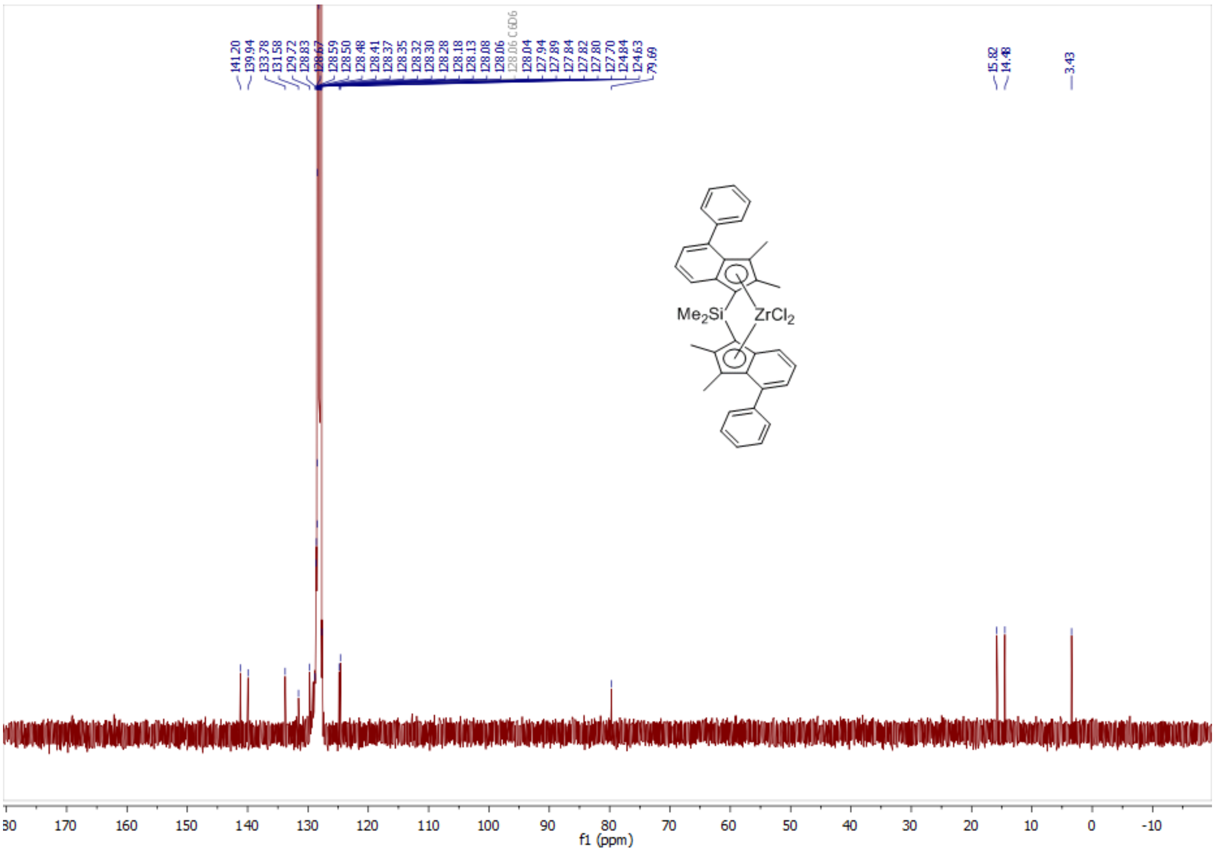

Figure S4. $^{13}C\{^{1}H\}$ NMR spectrum of **M21** in C_6D_6.

M22

***rac*-diethylsilanediylbis[η^5-2-methyl-4-(2-furyl)inden-1-yl]zirconium dichloride (M22)**

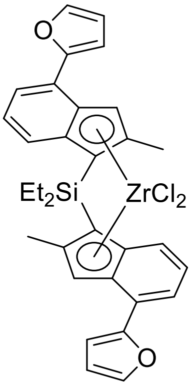

M22 was synthesized according to procedure previously published for M7 [13], but using (2-furyl)zinc chloride instead of MeZnCl, and ether instead of THF. The yield was 20% after crystallization from toluene. Anal. calc. for $C_{32}H_{30}Cl_2O_2SiZr$: C, 60.36; H, 4.75. Found: C, 60.81, H, 4.81. ^{1}H NMR ($CDCl_3$): δ 7.68 (d, J = 7.2 Hz, 2H), 7.57 (d, J = 8.6 Hz, 2H), 7.47 (m, 2H), 7.23 (s, 2H), 7.09 (dd, J = 8.6 Hz, J = 7.2 Hz, 2H), 6.78 (d, J = 3.4 Hz, 2H), 6.45 (dd, J = 3.4 Hz, J = 1.8 Hz, 2H), 2.26 (s, 6H), 1.97–1.78 (m, 4H), 1.48–1.43 (m, 6H). $^{13}C\{^{1}H\}$ NMR (C_6D_6): δ 142.8, 140.2, 139.7, 133.0, 129.1, 129.0, 126.2, 124.5, 120.5, 82.8, 26.2, 16.7, 2.6.

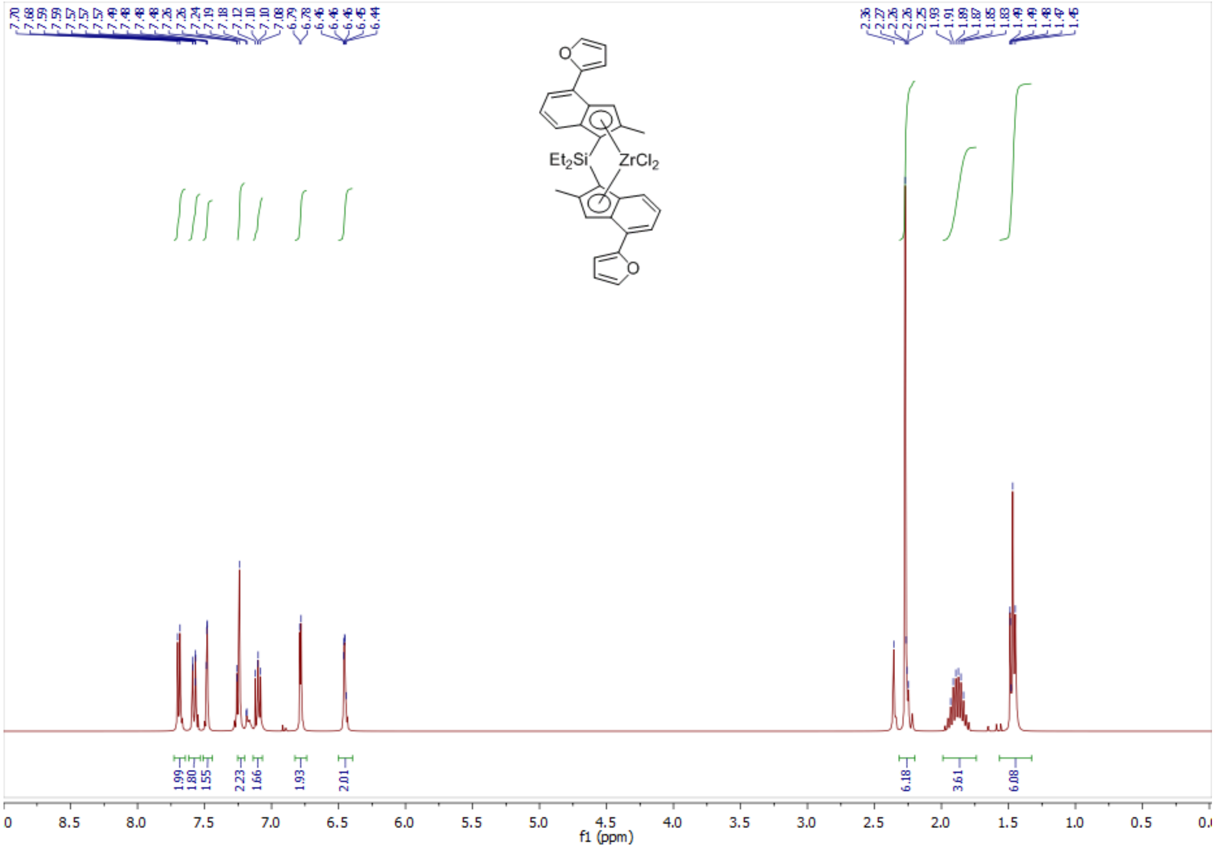

Figure S5. ^{1}H NMR spectrum of **M22** in $CDCl_3$. Traces of toluene are visible at 2.36 and 7.13–7.26 ppm.

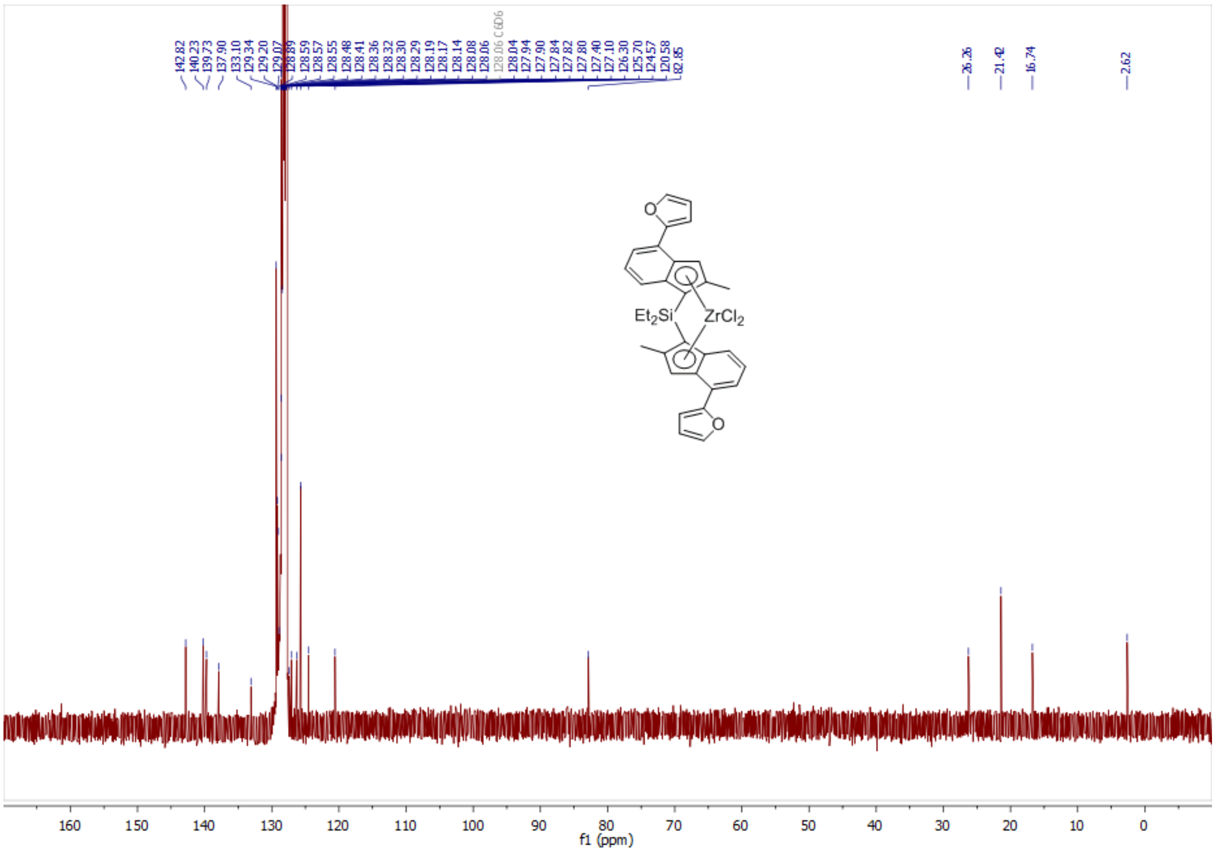

Figure S6. $^{13}C\{^{1}H\}$ NMR spectrum of **M22** in $CDCl_3$.

M23

4-phenyl-2,6-dimethyl-1-methoxyindane

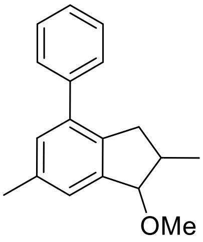

To a mixture of 1.20 g (1.0 mol.%) of $NiCl_2(PPh_3)IPr$ and 40.0 g (157 mmol) of 4-bromo-2,6-dimethyl-1-methoxyindane 220 ml (220 mmol) of 1.0 M phenylmagnesium bromide in THF was added at a such rate to maintain a gentle reflux. The resulting solution was refluxed for 40 min, and then cooled to room temperature. Water (500 ml) and dichloromethane (400 ml) were added to the reaction mixture, followed by 500 ml of 2 M HCl. The organic layer was separated, and the aqueous layer was extracted with 2 x 100 ml of dichloromethane. The combined organic extract was evaporated to dryness to give slightly greenish oil. The product was isolated by flash chromatography on silica gel 60 (40–63 µm; eluent: hexanes-dichloromethane = 5:1, vol., then 1:2, vol.). This procedure gave 38.2 g (97%) of the title product as a colorless thick oil which was a mixture of two diastereomers A and B in 1.5:1 molar ratio. ^{1}H NMR ($CDCl_3$): δ 7.49–7.41 (m, 4H in A and 4H in B), 7.39–7.32 (m, 1H in A and 1H in B), 7.23 (br.s, 1H in A), 7.22 (br.s, 1H in B), 7.18 (br.s, 1H in B), 7.17 (br.s, 1H in A), 4.56 (d, J = 5.7 Hz, 1H in B), 4.44 (d, J = 4.0 Hz, 1H in A), 3.54 (s, 3H in A), 3.50 (s, 3H in B), 3.33–3.23 (m, 1H in A), 2.93 (dd, J = 15.5 Hz, J = 7.1 Hz, 1H in B), 2.80 (dd, J = 15.5 Hz, J = 6.5 Hz, 1H in B), 2.65–2.45 (m, 2H in A and 1H in B), 2.44 (br.s, 3H in A and 3H in B), 1.16 (d, J = 6.9 Hz, 3H in A) 1.10 (d, J = 7.0 Hz, 3H in B). ^{13}C{^{1}H} NMR ($CDCl_3$): δ 143.58, 143.10, 140.90, 140.86, 138.43, 138.34, 138.12, 137.66, 136.52, 136.16, 129.50, 129.15, 128.47, 128.17, 126.82, 124.83, 124.80, 91.34, 86.15, 56.84, 56.68, 40.11, 39.04, 37.94, 37.80, 21.24, 19.16, 13.50.

2,5-dimethyl-7-phenyl-1H-indene

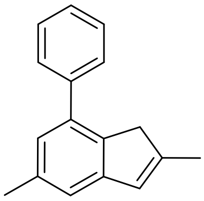

To a solution of 38.2 g (151 mmol) of 4-phenyl-2,6-dimethyl-1-methoxyindane in 350 ml of toluene 400 mg of TsOH was added. The resulting solution was refluxed using Dean-Stark head for 10 min, then cooled to room temperature, and finally washed by 100 ml of 10% K_2CO_3. The organic layer was separated, and the aqueous layer was extracted with 2 x 100 ml of dichloromethane. The combined organic extract was evaporated to dryness, and the resulting greenish solid was heated with 400 ml of n-hexane. This hot mixture was filtered through a short pad of silica gel 60 (40-63 µm), and the filtrate was kept for 5 h at room temperature, then 1 h at 0 °C, and finally 2 h at -30 °C. Precipitated large crystals were filtered off, washed with 200 of cold (0 °C) n-hexane and dried in vacuum to give 27.9 g (84%) of 2,5-dimethyl-7-phenyl-1*H*-indene. ^{1}H NMR ($CDCl_3$): δ 7.55–7.49 (m, 2H), 7.46–7.38 (m, 2H), 7.37–7.29 (m, 1H), 7.07 (s, 1H), 6.96 (s, 1H), 6.48 (m, 1H), 3.33 (s, 2H), 2.41 (s, 3H), 2.12 (s, 3H). ^{13}C{^{1}H} NMR ($CDCl_3$): δ 146.74, 146.59, 141.44, 137.89, 136.99, 136.56, 128.38, 128.31, 127.01, 126.90, 125.02, 119.74, 42.37, 21.42, 16.76.

bis(2,6-dimethyl-4-phenyl-1H-inden-1-yl)(dimethyl)silane

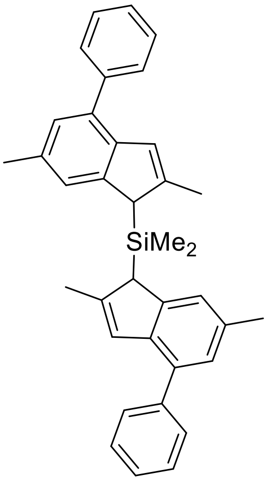

To a solution of 10.5 g (47.7 mmol) of 2,5-dimethyl-7-phenyl-1*H*-indene in 250 ml of ether and 7.5 ml of THF 19.6 ml (47.6 mmol) of 2.43 M *n*BuLi in hexanes was added in one portion at -50 °C. This mixture was stirred for 4 h at room temperature, then the resulting heterogeneous mixture was cooled to -40°C, and 250 mg of CuCN was added. The obtained mixture was stirred for 30 min at -25 °C followed by addition of 3.08 g (23.9 mmol) of dichlorodimethylsilane. Further on, this mixture was stirred overnight at ambient temperature. The resulting mixture was filtered through a short pad of silica gel 60 (40-63 µm) which was additionally washed by 2 x 50 ml of dichloromethane. The combined filtrate was evaporated in vacuum, and thus obtained residue was dried in vacuum. This procedure gave 12.0 g (ca. 100%) of the title product (ca. 80% purity by NMR) as yellowish glassy solid, which was used further without additional purification. According to ^{1}H NMR, the product was a 1.5:1 mixture of *meso*- and *rac*-isomers. HRMS: $[M]^+$ calc. for $C_{36}H_{36}Si$: 496.2586; found: 496.2571. ^{1}H NMR ($CDCl_3$): δ 7.55–7.52 (m, 4H in *rac* and 4H in *meso*), 7.47–7.42 (m, 4H in *rac* and 4H in *meso*), 7.37–7.31 (m, 2H in *rac* and 2H in *meso*), 7.22 (br.s, 2H in *meso*), 7.18 (br.s, 2H in *rac*), 7.11 (br.s, 2H in *meso*), 7.09 (br.s, 2H in *rac*), 6.76 (br.s, 2H in *rac* and 2H in *meso*), 3.76 (s, 2H in *rac*), 3.73 (s, 2H in *meso*), 2.412 (s, 6H in *meso*), 2.407 (s, 6H in *rac*), 2.24 (s, 6H in *rac*), 2.17 (s, 6H in *meso*), –0.16 (s, 3H in *meso*), –0.18 (s, 6H in *rac*), –0.20 (s, 3H in *meso*).$^{13}C\{^1H\}$ NMR ($CDCl_3$): δ 146.56, 146.48, 145.97, 145.89, 141.43, 140.45, 133.74, 133.7, 132.56, 132.45, 128.86, 128.3, 126.64, 126.5, 125.74, 125.7, 123.11, 47.27, 47.23, 21.6, 17.99, 17.92, –5.50, –5.55, –5.8.

***rac*-dimethylsilanediyl-bis(η^5-2,6-dimethyl-4-phenylinden-1-yl)zirconium dichloride (M23)**

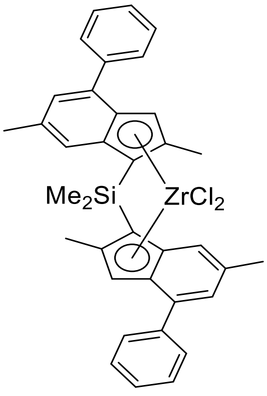

To a solution of 12.0 g (23.8 mmol) of bis(2,6-dimethyl-4-phenyl-1*H*-inden-1-yl)(dimethyl)silane in 250 ml of ether 19.6 ml (47.6 mmol) of 2.43 M ^{n}BuLi in hexanes was added in one portion at -50 °C. This mixture was stirred overnight at room temperature, then it was cooled to -60 °C, and 5.56 g (23.9 mmol) of $ZrCl_4$ was added. The reaction mixture was stirred for 24 h and then evaporated to dryness in vacuum. The residue was taken up in 250 ml of toluene, and thus obtained suspension was filtered while hot through glass frit (G4). On the evidence of NMR spectroscopy, the filtrate consisted of a mixture of *rac*- and *meso*-zirconocenes (~1:1.1 molar ratio). The filtrate was evaporated to ca. 200 ml. The orange crystalline solid precipitated overnight at room temperature was filtered off (G3) and then dried in vacuum. This procedure gave 2.85 g (18.2%) of *meso*-dimethylsilanediyl-bis(*η*^{5}-2,6-dimethyl-4-phenylinden-1-yl)zirconium dichloride, containing ~5% of *rac*-isomer. The mother liquor was evaporated to ca. 90 ml and allowed to stand for 45 min at room temperature. The precipitated yellow solid was filtered off (G3) and dried in vacuum. This procedure gave 1.00 g (6.4%) of *rac*-complex contaminated with 1% of *meso*-isomer. The mother liquor was allowed to stand overnight at room temperature. The precipitated orange solid was filtered off (G3) and then dried in vacuum. This procedure gave 2.30 g (14.7%) of a mixture of *rac*-and *meso*-zirconocenes in ratio ca. 56/44. The mother liquor was evaporated again to ca. 50 ml and allowed to stand overnight at room temperature. The precipitated yellow solid was filtered off (G3) and dried in vacuum. This procedure gave 0.85 g (5.4%) of *rac*-complex contaminated with 5% of *meso*-isomer. Finally, the mother liquor was evaporated to ca. 15 ml, and 50 ml of n-pentane was added. The precipitated orange solid was

filtered off (G3) and dried in vacuum to give 5.70 g (36.4%) of a slightly dirty mixture of *rac*-and *meso*-zirconocenes in ratio ca. 60/40. Thus, the total yield of *rac*- and *meso*-zirconocenes isolated in this synthesis was 12.7 g (81%). *Rac*-complex. Anal. calc. for $C_{36}H_{34}Cl_2SiZr$: C, 65.83; H, 5.22. Found: C, 65.95; H, 5.40. ^{1}H NMR ($CDCl_3$): δ 7.62 (d, J = 7.3 Hz, 2H), 7.41 (dd, J = 7.3 Hz, 2H), 7.32 (t, J = 7.3 Hz, 1H), 7.22 (s, 1H), 6.90 (s, 1H), 2.37 (s, 3H), 2.23 (s, 3H), 1.32 (s, 3H). ^{13}C{^{1}H} NMR ($CDCl_3$): δ 139.50, 138.23, 135.74, 134.80, 130.51, 129.17, 128.56 (two resonances), 128.15, 127.61, 122.76, 121.92, 82.41, 22.14, 18.35, 2.79.

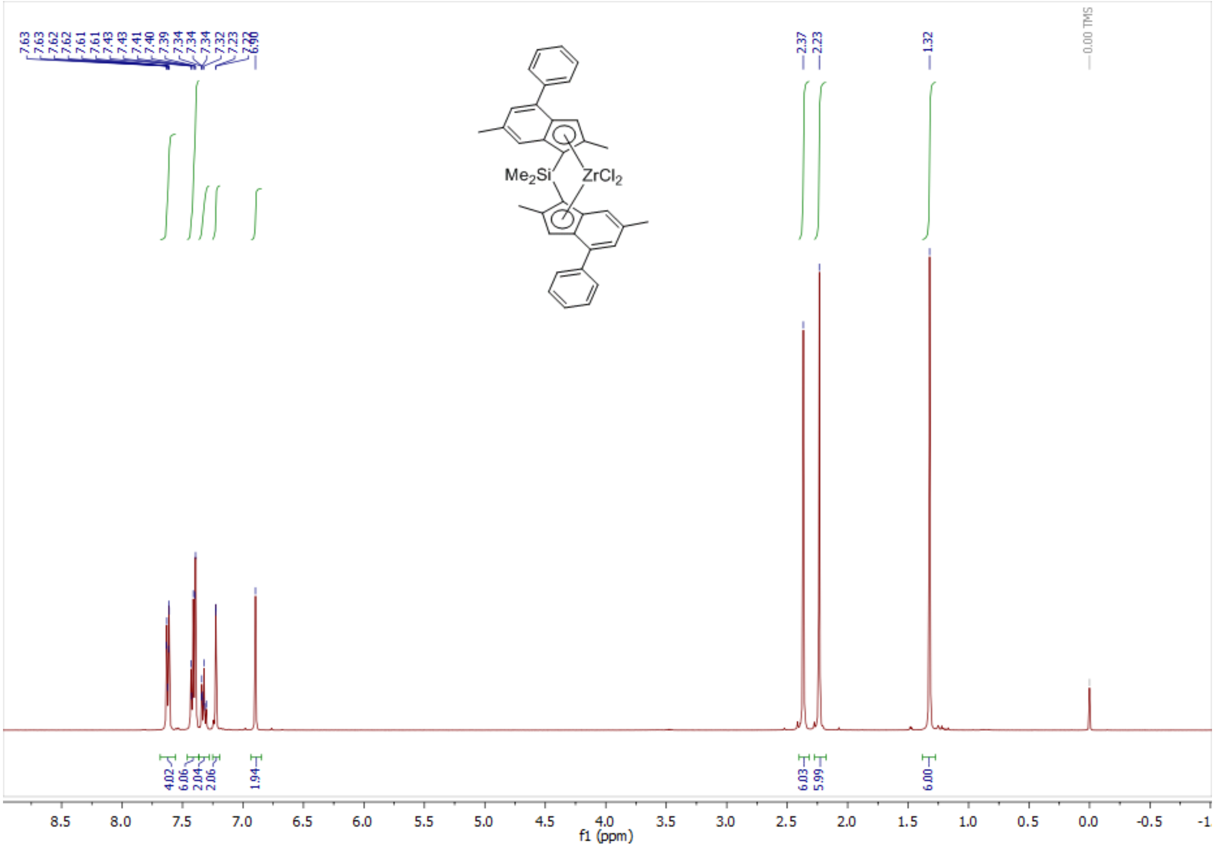

Figure S7. ^{1}H NMR spectrum of **M23** in $CDCl_3$.

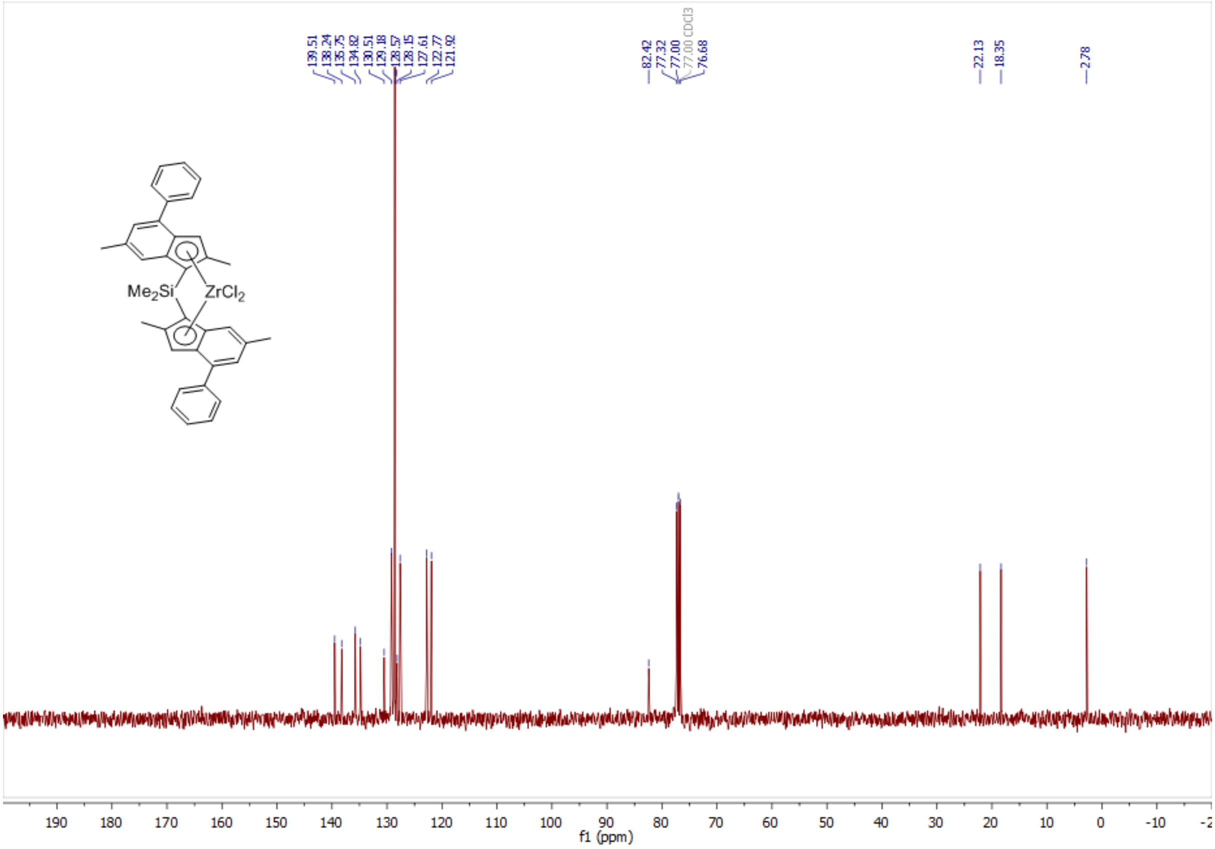

Figure S8. $^{13}C\{^{1}H\}$ NMR spectrum of **M23** in $CDCl_3$.

M24

6-isopropyl-4-phenyl-2-methyl-1-methoxyindane

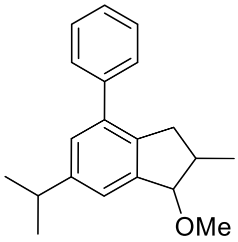

To a mixture of 1.10 g (1.0 mol.%) of $NiCl_2(PPh_3)IPr$ and 38.8 g (137.1 mmol) of 4-bromo-1-methoxy-6-isopropyl-2-methylindane 200 ml (200 mmol) of 1.0 M phenylmagnesium bromide in THF was added at a such rate to maintain a gentle reflux. The resulting solution was refluxed for 1h, then cooled to room temperature. 500 ml of water and 500 ml of dichloromethane were added to the reaction mixture, followed by 500 ml of 1 M HCl. The organic layer was separated, and the aqueous layer was extracted with 250 ml of dichloromethane. The combined organic extract was evaporated to dryness to give a slightly greenish oil. The product was isolated by flash-chromatography on silica gel 60 (40-63 µm; eluent: hexanes-dichloromethane = 1:2, vol.). This procedure gave 37.9 g (99%) of the title product as a colorless thick oil which is a mixture of two diastereomers A and B in 1.2:1 molar ratio. ^{1}H NMR ($CDCl_3$): δ 7.47–3.38 (m, 4H in A and 4H in B), 7.35–7.29 (m, 1H in A and 1H in B), 7.24 (m, 1H in A and 1H in B), 7.18 (m, 1H in A and 1H in B), 4.54 (d, J = 5.7 Hz, 1H in B), d, (= 4.2 Hz, 1H in A), 3.52 (s, 3H in A), 3.47 (s, 3H in B), 3.29–3.19 (m, 1H in A), 2.97 (sept, 1H in A and 1H in B), 2.91 (dd, J = 15.5 Hz, J = 7.1 Hz, 1H in B), 2.77 (dd, J = 15.5 Hz, J = 6.5 Hz, 1H in B), 2.58 (m, 1H in B), 2.53–2.43 (m, 2H in A), 1.29 (d, J = 6.9 Hz, 6H in A and 6H in B), 1.14 (d, J = 6.7 Hz, 3H in A), (d, J = 6.9 Hz, 3H in B). $^{13}C\{^{1}H\}$ NMR ($CDCl_3$): δ 147.79, 147.49, 143.50, 143.07, 141.10 (two resonances), 138.53, 138.36, 138.35, 137.98, 128.55, 128.49, 128.19, 128.16, 127.03, 126.81, 126.79, 126.74, 122.12, 122.07, 91.41, 86.28, 56.88, 56.64, 40.17, 38.98, 37.98, 37.84, 34.03, 34.07, 24.08, 24.30, 24.28, 24.14, 19.22, 13.58.

5-isopropyl-2-methyl-7-phenyl-1H-indene

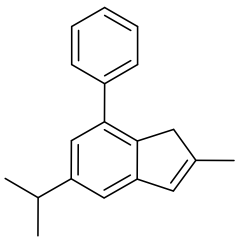

To a solution of 37.9 g (135.2 mmol) of 6-isopropyl-4-phenyl-2-methyl-1-methoxyindanein 350 ml of toluene 250 mg of TsOH was added. The resulting solution was refluxed using Dean-Stark head for 10 min, then, additional 100 mg of TsOH was added, and the reaction mixture was refluxed using Dean-Stark head for another 10 min. Then, it was cooled to room temperature and washed by 100 ml of 10% K_2CO_3. The organic layer was separated, and the aqueous layer was extracted with 2 x 100 ml of dichloromethane. The combined organic extract was evaporated to dryness. The resulting oil was dissolved in 150 ml of n-hexane. This solution was filtered through a short pad of silica gel 60 (40-63 μm), which was washed additionally with 2 x 50 ml of n-hexane. The filtrate was evaporated, and the residue was dried in vacuum to give 30.3 g (90%) of the title product. ^{1}H NMR ($CDCl_3$): δ 7.53 (dm, J = 7.3 Hz, 2H), 7.42 (dd, J = 7.3 Hz, 2H), 7.32 (tm, J = 7.3 Hz, 1H), 7.13 (s, 1H), 7.01 (s, 1H), 6.49 (s, 1H), 3.33 (s, 2H), 2.96 (sept, J = 6.9 Hz, 1H), 2.11 (s, 3H), 1.30 (d, J = 6.9 Hz, 1H). ^{13}C{^{1}H} NMR ($CDCl_3$): δ 147.97, 146.69, 146.50, 141.63, 138.35, 137.05, 128.42, 128.32, 127.16, 126.89, 122.63, 117.04, 42.41, 34.24, 24.33, 16.74.

bis(6-isopropyl-2-methyl-4-phenyl-1H-inden-1-yl)(dimethyl)silane

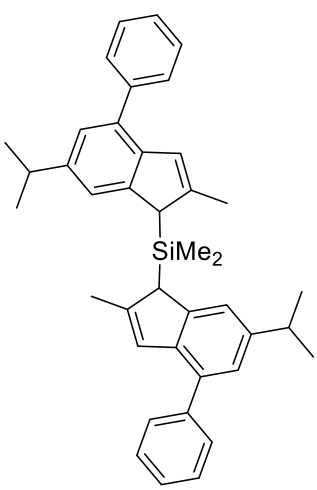

To a solution of 12.9 g (51.9 mmol) of 5-isopropyl-2-methyl-7-phenyl-1*H*-indene in 250 ml of ether and 11 ml of THF 21.4 ml (52.0 mmol) of 2.43 M ^{n}BuLi in hexanes was added in one portion at -50 °C. This mixture was stirred for 5 h at room temperature, then the resulting heterogeneous light-orange solution with a lot of white precipitate was cooled to -60°C, and 3.35 g (26.0 mmol) of dichlorodimethylsilane was added in one portion. Further on, this mixture was stirred overnight at ambient temperature. The resulting mixture was filtered through a short pad of silica gel 60 (40-63 μm) which was additionally washed by 2 x 50 ml of dichloromethane. The combined filtrate was evaporated under reduced pressure. The product was isolated by flash-chromatography on silica gel 60 (40-63 μm; eluent: hexanes-dichloromethane = 10:1, vol., then 3:1, vol.). This procedure gave 12.34 g (86%) of the title product (ca. 90% purity by NMR, ~1.3:1 mixture of *meso*- and *rac*- isomers) as yellowish glass, which was used further without additional purification. HRMS: $[M]^+$ calc. for $C_{40}H_{44}Si$: 552.3212; found: 552.3224. ^{1}H NMR ($CDCl_3$): δ 7.59–7.51 (m, 4H in *meso* and *rac*), 7.50–7.43 (m, 4H in *meso* and *rac*), 7.39–7.33 and 7.26 (m and br.s, respectively, sum 4H in *meso* and *rac*), 7.15 (m, 2H in *meso* and *rac*), 7.13 (2s), 6.78 (s, 2H in *meso*), 6.74 (s, 2H in *rac*), 3.74 (s, 2H in *meso*), 3.71 (s, 2H in *rac*), 2.97 (sept, J = 6.9 Hz, 2H in *meso* and 2H in *rac*), 2.23 (br.s, 6H in *meso*), 2.15 (br.s, 6H in *rac*), 1.30 (d, J = 6.9 Hz, 12H in *meso*), 1.28 (d, J = 6.9 Hz, 12H in *rac*), –0.13 (s, 3H in *meso*), –0.14 (s, 6H in *rac*), –0.20 (s, 3H in *meso*).^{13}C{^{1}H} NMR ($CDCl_3$): δ 147.02, 146.76, 145.92, 145.87, 143.95, 141.70, 141.66, 140.88, 140.79, 133.73, 128.95, 128.36, 126.67, 125.74, 125.67, 124.15, 124.07, 120.45, 47.53, 47.40, 34.21, 24.61, 24.52, 24.38, 24.27, 17.98, 17.92, –5.13, –5.24.

rac-dimethylsilanediylbis[η^5-6-isopropyl-2-methyl-4-phenylinden-1-yl]zirconium dichloride (M24)

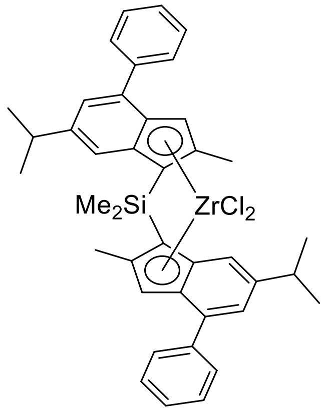

To a cooled to -50 °C solution of 12.3 g (22.3 mmol) of bis(6-isopropyl-2-methyl-4-phenyl-1*H*-inden-1-yl)(dimethyl)silanein 250 ml of ether 18.4 ml (44.7 mmol) of 2.43 M ^{n}BuLi in hexanes was added in one portion. This mixture was stirred overnight at room temperature. The resulting dark-red solution was cooled to -60 °C, and 5.21 g (22.4 mmol) of $ZrCl_4$ was added. The reaction mixture was stirred for 24 h resulting in dark-red solution with little sediment. This mixture was evaporated to dryness, and the residue was heated with 100 ml of toluene. The obtained hot suspension was filtered through glass frit (G4). The filtrate was evaporated to ca. 20 ml, heated to ca. 60 °C, and 25 ml of n-hexane was added. Yellow solid precipitated from this solution overnight at room temperature was filtered off (G3) and then dried in vacuum. This procedure gave 3.30 g (21%) of *rac*-complex. The mother liquor was evaporated to dryness, and the residue was recrystallized from a mixture of 10 ml of toluene and 25 ml of n-hexane to give 2.50 g (16%) of *meso*-complex. *Rac*-complex. Anal. calc. for $C_{40}H_{42}Cl_2SiZr$: C, 67.38; H, 5.94. Found: C, 67.51; H, 6.02. ^{1}H NMR ($CDCl_3$): *δ* 7.63 (dm, *J* = 7.3 Hz, 2H), 7.44 (s, 1H), 7.41 (dd, *J* = 7.3 Hz, 2H), 7.32 (tt, *J* = 7.3 Hz, 1H), 7.28 (d, *J* = 1.0 Hz, 1H), 6.90 (s, 1H), 2.92 (sept, *J* = 6.9 Hz, 1H), 2.26 (s, 3H), 1.33 (s, 3H), 1.25 (d, *J* = 6.9 Hz, 6H). ^{13}C{^{1}H} NMR ($CDCl_3$): *δ* 146.40, 139.73, 138.36, 134.91, 130.94, 128.57 (two resonances), 127.94, 127.59, 127.43, 121.84, 120.03, 82.43, 34.55, 23.49, 23.36, 18.51, 2.79.

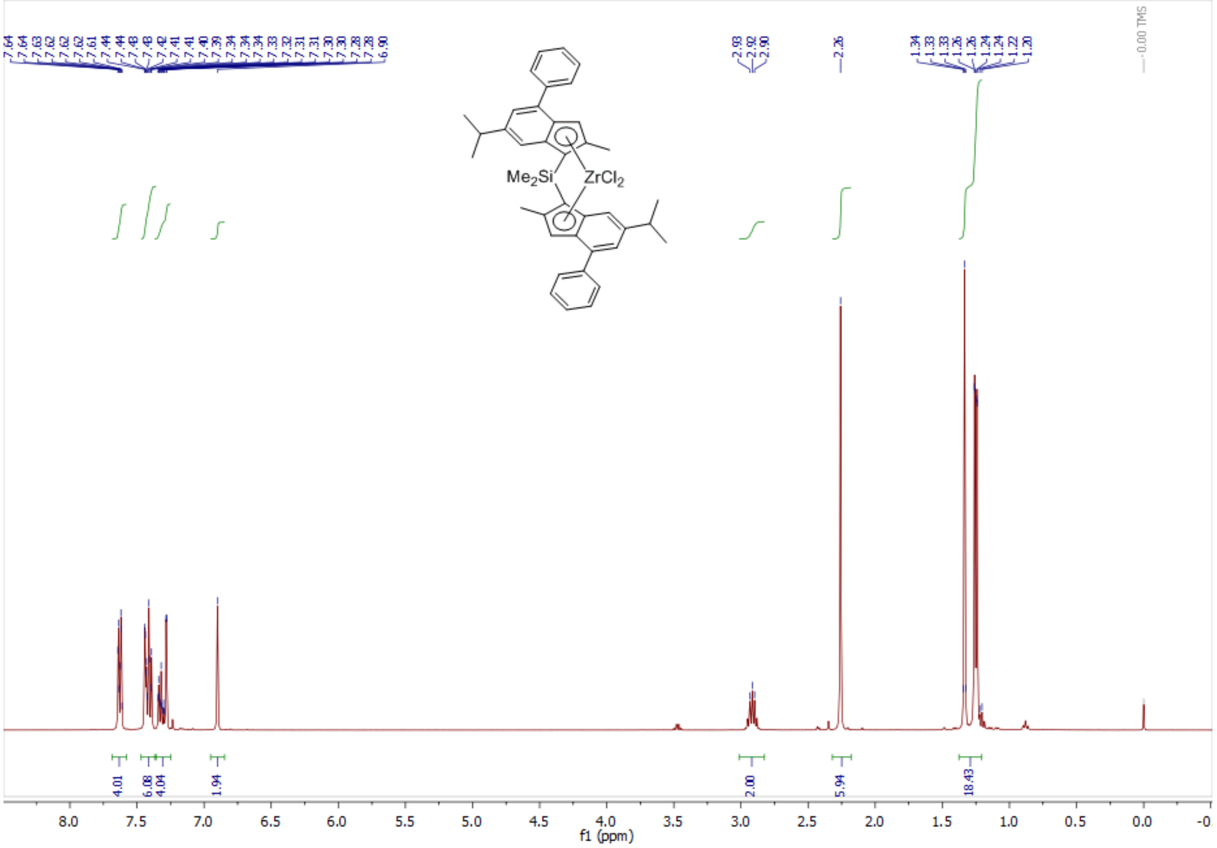

Figure S9.^{1}H NMR spectrum of **M24** in $CDCl_3$.

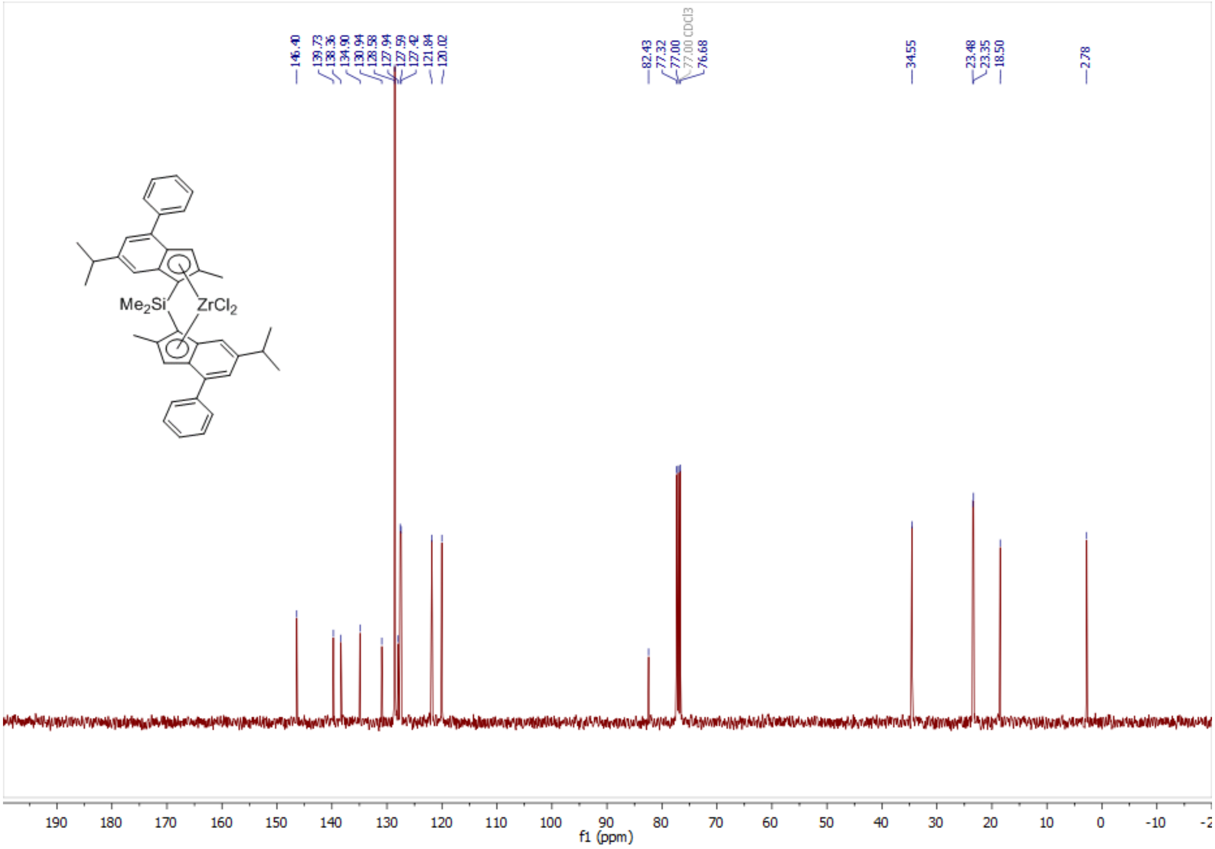

Figure S10. $^{13}C\{^1H\}$ NMR spectrum of **M24** in $CDCl_3$.

M25

6-tert-butyl-4-phenyl-2-methyl-1-methoxyindane

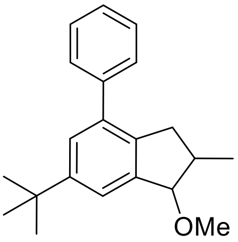

To a mixture of 1.2 g (1.0 mol.%) of $NiCl_2(PPh_3)IPr$ and 45.2 g (152 mmol) of 4-bromo-1-methoxy-6-*tert*-butyl-2-methylindan 220 ml (220 mmol) of 1.0 M phenylmagnesium bromide in THF was added at a such rate to maintain a gentle reflux. The resulting solution was refluxed for 40 min, then cooled to room temperature. 500 ml of water and 500 ml of dichloromethane were added to the reaction mixture followed by 500 ml of 1 M HCl. The organic layer was separated, and the aqueous layer was extracted with 250 ml of dichloromethane. The combined organic extract was evaporated to dryness to give a slightly greenish oil. The product was isolated by flash-chromatography on silica gel 60 (40-63 µm; eluent: hexanes-dichloromethane = 5:1, vol., then 1:2, vol.). This procedure gave 44.0 g (98%) of the title product as a colorless thick oil which was a mixture of two diastereomers A and B in 1.3:1 molar ratio. ^1H NMR ($CDCl_3$): δ 7.46–7.29 (m, 7H in A and B), 4.55 (d, J = 5.7 Hz, 1H in B), 4.44 (d, J = 4.3 Hz, 1H in A), 3.51 (s, 3H in A), 3.47 (s, 3H in B), 3.30–3.19 (1H in A), 2.90 (dd, J = 15.5 Hz, J = 7.1 Hz, 1H in B), 2.76 (dd, J = 15.5 Hz, J = 6.5 Hz, 1H in B), 2.64–2.42 (m, 2H in A and 1H in B), 1.37 (s, 9H in A or B), 1.36 (s, 9H in B or A), 1.14 (d, J = 6.9 Hz, 3H in A), 1.07 (d, J = 6.9 Hz, 3H in B). $^{13}C\{^1H\}$ NMR ($CDCl_3$): δ 150.13, 149.78, 143.22, 142.78, 141.36, 141.32, 138.16, 138.03, 137.65, 128.57, 128.20, 126.80, 126.03, 125.69, 121.05, 121.01, 91.54, 86.40, 56.87, 56.58, 40.13, 38.97, 37.94, 37.77, 34.66, 31.57, 19.30, 13.60.

5-tert-butyl-2-methyl-7-phenyl-1H-indene

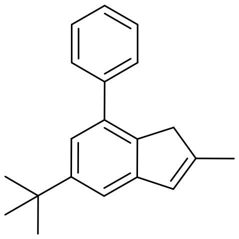

To a solution of 44.0 g (149 mmol) of 6-*tert*-butyl-4-phenyl-2-methyl-1-methoxyindanein 300 ml of toluene 300 mg of TsOH was added. The resulting solution was refluxed using Dean-Stark head for 10 min, then additional 300 mg of TsOH was added, and the formed mixture was refluxed using Dean-Stark head for another 10 min. Then, it was cooled to room temperature and finally washed by 100 ml of 10% K_2CO_3. The organic layer was separated, and the aqueous layer was extracted with 150 ml of dichloromethane. The combined organic extract was evaporated to dryness. The obtained oil was dissolved in 200 ml of n-hexane. This solution was filtered through a short pad of silica gel 60 (40-63 µm), which was washed additionally with 2 x 50 ml of n-hexane. The filtrate was evaporated to dryness, and the residue was dried in vacuum to give 38.0 g (97%) of the title product as yellowish oily liquid. ^{1}H NMR ($CDCl_3$): δ 7.54 (d, J = 7.3 Hz, 2H), 7.43 (dd, J = 7.3 Hz, 2H), 7.36–7.29 (m, 2H), 7.17 (d, J = 1.3 Hz, 1H), 6.51 (s, 1H), 3.33 (s, 2H), 2.11 (s, 3H), 1.37 (s, 9H). ^{13}C{^{1}H} NMR ($CDCl_3$): δ 150.24, 146.48, 146.41, 141.85, 138.01, 136.72, 128.46, 128.34, 127.31, 126.90, 121.50, 116.15, 42.36, 34.74, 31.68, 16.76.

bis(6-tert-butyl-2-methyl-4-phenyl-1H-inden-1-yl)(dimethyl)silane

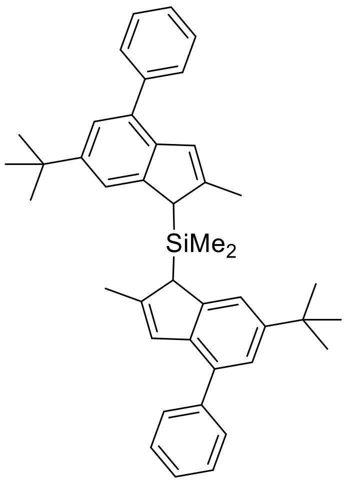

To a solution of 13.8 g (52.7 mmol) of 5-*tert*-butyl-2-methyl-7-phenyl-1*H*-indene in 250 ml of ether and 7.5 ml of THF 21.7 ml (52.7 mmol) of 2.43 M^{n}BuLi in hexanes was added in one portion at -60 °C. This mixture was stirred for 4 h at room temperature, then the obtained suspension was cooled to -40°C, and 250 mg of CuCN was added. This mixture was stirred for 30 min at -25 °C followed by addition of 3.41 g (26.4 mmol) of dichlorodimethylsilane. Further on, this mixture was stirred overnight at ambient temperature. The resulting mixture was filtered through a short pad of silica gel 60 (40-63 µm) which was additionally washed by 2 x 50 ml of dichloromethane. The combined filtrate was evaporated under reduced pressure, and the residue was dried in vacuum at elevated temperature. This procedure gave 15.3 g (~100%) of the title product (ca. 90% purity by NMR, ~2:1 mixture of *rac* and *meso* isomers) as yellowish glassy solid, which was used further without additional purification. HRMS: [M]$^+$ calc. for $C_{42}H_{48}Si$: 580.3525; found: 580.3538. ^{1}H NMR ($CDCl_3$): δ 7.61–7.28 (m, 14H in *rac* and *meso*), 6.77 (br.s, 2H in *meso*), 6.72 (br.s, 2H in *rac*), 3.73 (br.s, 2H in *meso*), 3.72 (br.s, 2H in *rac*), 2.21 (br.s, 3H in *meso*), 2.11 (br.s, 3H in *rac*), 1.37 (s, 9H in *rac*), 1.36 (s, 9H in *meso*), –0.13 (s, 3H in *meso*), –0.14 (s, 6H in *rac*) and –0.19 (s, 3H in *meso*). ^{13}C{^{1}H} NMR ($CDCl_3$): δ 147.25, 146.94, 146.12, 145.62, 145.51, 141.88, 141.83, 140.46, 140.30, 133.31, 133.26, 128.93, 128.33, 126.62, 125.61, 125.54, 122.74, 119.64, 119.48, 47.60, 47.43, 34.65, 31.74, 17.93, 17.87, –5.12, –5.34.

rac-dimethylsilanediylbis(η5-6-tert-butyl-2-methyl-4-phenylinden-1-yl)zirconium dichloride (M25)

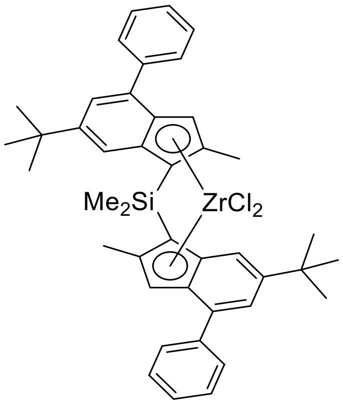

To a cooled to –60 °C solution of 15.3 g (26.4 mmol) of bis(6-*tert*-butyl-2-methyl-4-phenyl-1*H*-inden-1-yl)(dimethyl)silanein 250 ml of ether 21.7 ml (52.7 mmol) of 2.43 M ^{n}BuLi in hexanes was added in one portion. This mixture was stirred overnight at room temperature. The resulting red solution was cooled to -60 °C, and 6.15 g (26.39 mmol) of $ZrCl_4$ was added. Thus obtained mixture was stirred for 24 h giving red suspension. On the evidence of NMR spectroscopy, the precipitate is almost pure *rac*-zirconocene (in a mixture with LiCl), while the solution includes ca. 35 to 65 mixture of *rac*- and *meso*-zirconocenes. The precipitate was filtered off (G4), then poured into 100 ml of hot toluene, and thus obtained hot suspension was filtered through glass frit (G4). The filtrate was evaporated to ca. 60 ml. Orange crystals precipitated overnight at room temperature were filtered off and dried in vacuum. This procedure gave 4.00 g (21%) of *rac*-dimethylsilanediyl-bis(*η*^{5}-6-*tert*-butyl-2-methyl-4-phenylinden-1-yl)zirconium dichloride. The mother liquor was recrystallized from ca. 20 ml of toluene to give 2.80 g (14%) of *rac*-complex as orange crystalline solid. The filtrate originated from filtration of the original reaction mixture was evaporated to ca. 30 ml, and 100 ml of n-hexane was added. Yellow solid precipitated from this mixture at room temperature was filtered off (G3) and dried in vacuum to give 5.20 g (27%) of a ca. 3:7 mixture of *rac*-and *meso*-zirconocenes. Thus, the total yield of *rac*- and *meso*-zirconocenes isolated in this synthesis was 12.0 g (61%). *Rac*-zirconocene. Anal. calc. for $C_{42}H_{46}Cl_2SiZr$: C, 68.07; H, 6.26. Found: C, 68.18; H, 6.34. ^{1}H NMR ($CDCl_3$): *δ* 7.63 (dm, *J* = 7.3 Hz, 2H), 7.58 (s, 1H), 7.47 (d, *J* = 1.1 Hz, 1H), 7.42 (dd, *J* = 7.3 Hz, 2H), 7.33 (tm, *J* = 7.3 Hz, 1H), 6.89 (s, 1H), 2.27 (s, 3H), 1.34 (s, 3H), 1.33 (s, 9H). ^{13}C{^{1}H} NMR ($CDCl_3$): *δ* 148.45, 139.94, 137.95, 134.92, 130.55, 128.58 (two resonances), 127.57, 126.44, 121.61, 118.78, 82.74, 35.10, 30.75, 18.53, 2.71.

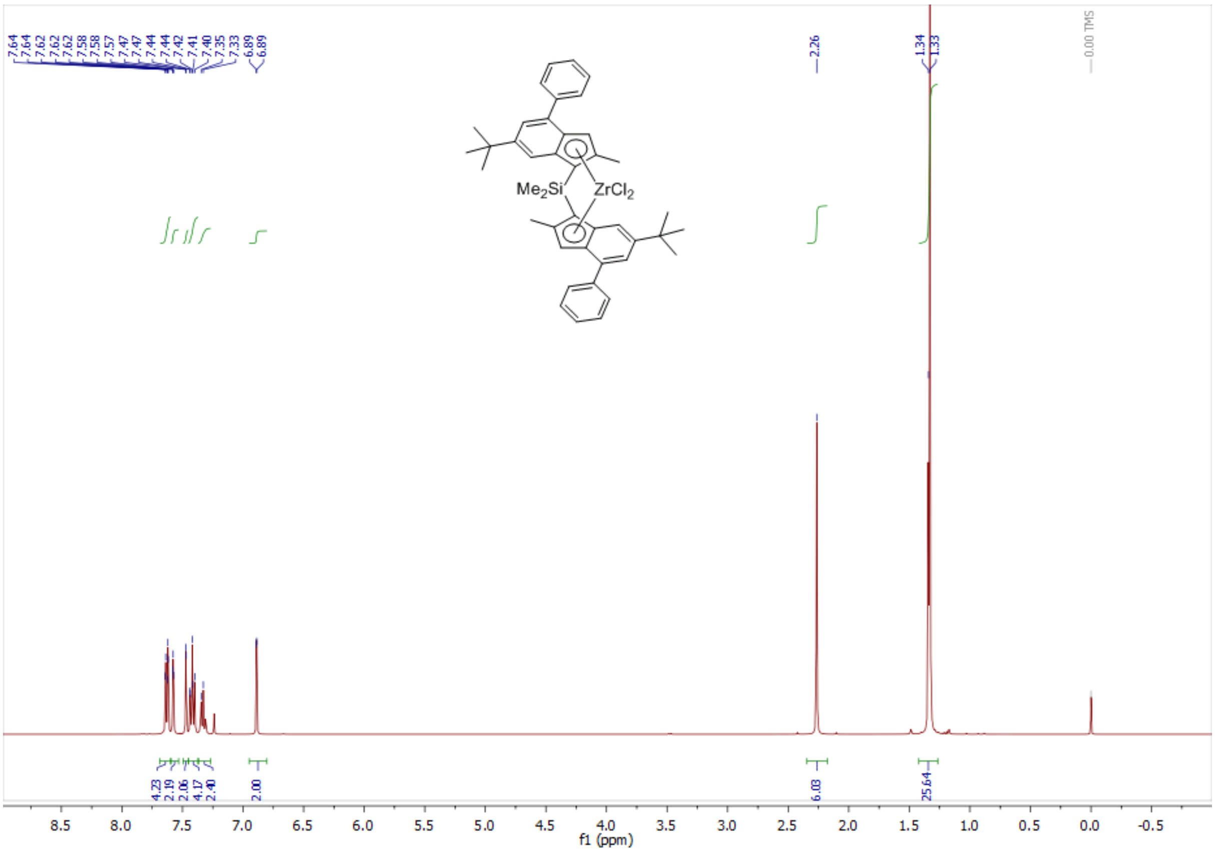

Figure S11.^{1}H NMR spectrum of **M25** in $CDCl_3$.

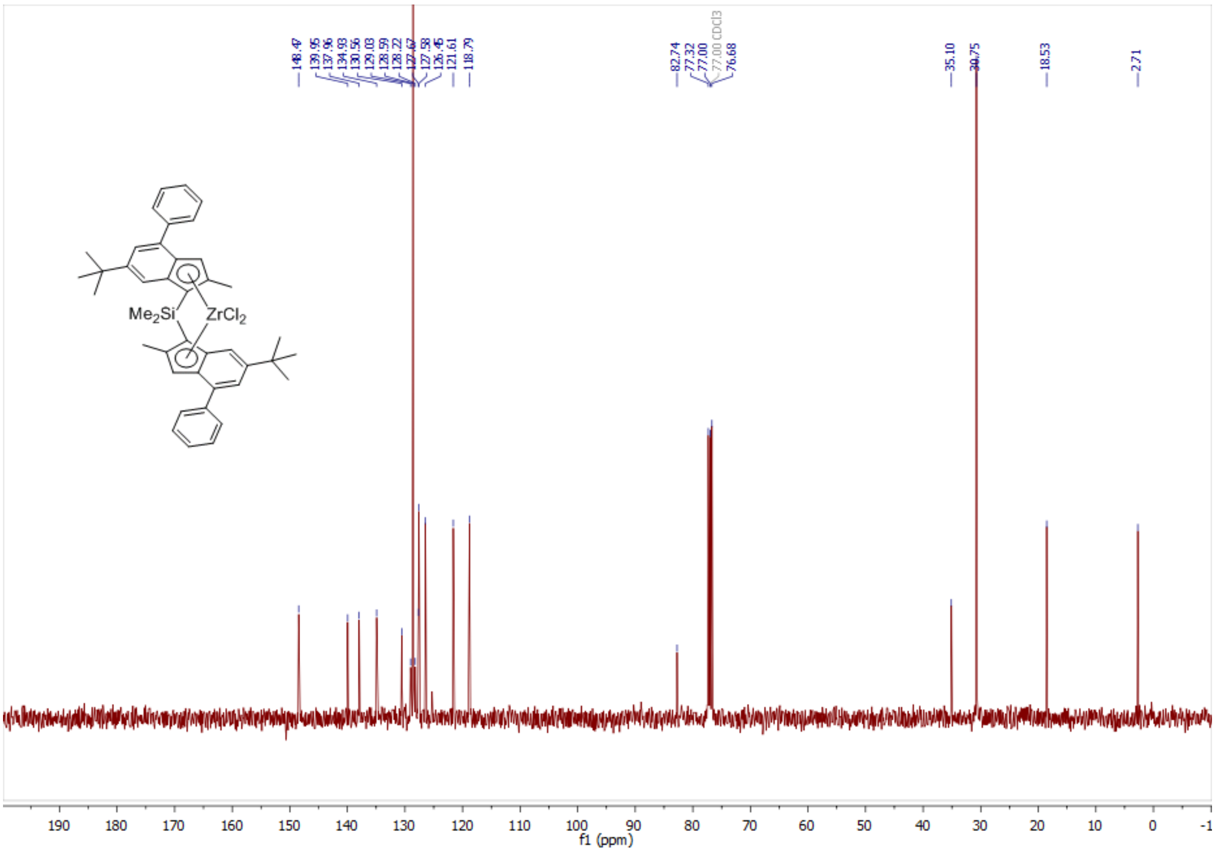

Figure S12.$^{13}C\{^1H\}$ NMR spectrum of **M25** in $CDCl_3$.

M27

6-chloro-1-methoxy-2-methyl-4-phenylindane

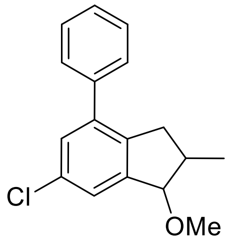

To a mixture of 27.8 g (101 mmol) of 4-bromo-6-chloro-1-methoxy-2-methylindane, 15.4 g (126.3 mmol, 1.25 equiv.) of $PhB(OH)_2$, 28.9 g (273 mmol) of Na_2CO_3, 320 ml of DME and 130 ml of H_2O, 1.20 g (5.35 mmol, 5.3 mol. %) of $Pd(OAc)_2$ and 2.79 g (10.6 mmol, 10.5 mol.%) of triphenylphosphine were added. This mixture was refluxed for 6 h, then cooled to room temperature, evaporated to dryness, and 1.5 liter of cold water was added. The crude product was extracted with 3 x 250 ml of dichloromethane. The combined organic extract was dried over K_2CO_3 and then evaporated to dryness to give black oily liquid. The product was isolated by flash chromatography of the residue on silica gel 60 (40-63 µm; eluent: hexanes-dichloromethane = 3:1, vol., then hexanes-dichloromethane = 1:1, vol.). This procedure gave 23.9 g (87%) of the title product as yellowish oil. According to ^{1}H NMR, the product was a mixture if diastereomers A and B in 1.6:1 molar ratio. ^{1}H NMR ($CDCl_3$):*δ* 7.44–7.26 (m, 7H in A and B), 4.50 (d,*J* = 5.7 Hz, 1H in B), 4.38 (d, *J* = 4.4 Hz, 1H in A), 3.49 (s, 3H in A), 3.44 (s, 3H in B), 3.25–3.14 (m, 1H in A), 2.87 (dd, *J* = 15.8 Hz, *J* = 7.0 Hz, 1H in B), 2.73 (dd, *J* = 15.8 Hz, *J* = 6.4 Hz, 1H in B), 2.61–2.39 (m, 2H in A and 1H in B), 1.11 (d, *J* = 6.8 Hz, 3H in A), 1.04 (d, *J* = 6.9 Hz, 3H in B).^{13}C{^{1}H} NMR ($CDCl_3$): *δ* 145.28, 144.95, 140.04, 139.96, 139.47, 139.43, 139.01, 132.37, 132.04, 128.35, 128.13, 127.45, 124.12, 124.07, 90.82, 85.74, 56.94, 56.88, 40.45, 39.07, 37.79, 37.65, 18.86, 13.32.

6-mesityl-1-methoxy-2-methyl-4-phenylindane

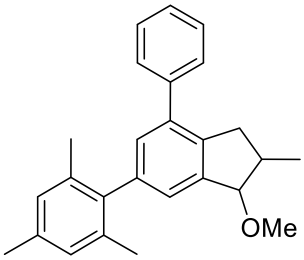

To a mixture of 2.00 g (2.90 mol.%) of $NiCl_2(PPh_3)$IPr and 23.9 g (87.6 mmol) of 6-chloro-1-methoxy-2-methyl-4-phenylindane 150 ml (150 mmol) of 1.0 M 2-mesitylmagnesium bromide in THF was added in one portion. The resulting solution was refluxed for 2 h, then cooled to room temperature, 300 ml of water and 400 ml of dichloromethane were added followed by 200 ml of 1 M HCl. The organic layer was separated, and the aqueous layer was extracted with 150 ml of dichloromethane. The combined organic extract was evaporated to dryness to give red oil. The product was isolated by flash-chromatography on silica gel 60 (40-63 µm; eluent: hexanes-dichloromethane = 3:1, vol., then 1:1, vol.). This procedure gave 27.8 g (89%) of the title productas lightly yellowish thick oil which is a mixture of two diastereomers A and B in 1.5:1 molar ratio. ^{1}H NMR ($CDCl_3$): *δ* 7.49–7.30 (m, 5H in A and 5H in B), 7.15–7.11 (m, 2H in A and 2H in B), 6.95 (br.s, 2H in A and 2H inB), 4.52 (d, *J* = 5.5 Hz, 1H in B), 4.48 (d, *J* = 4.2 Hz, 1H in A), 3.48 (s, 3H in A), 3.42 (s, 3H in B), 3.38–3.30 (m, 1H in A), 3.04–2.85 (m, 2H in B), 2.65–2.48 (m, 2H in A and 1H in B), 2.33 (br.s, 3H in A and 3H in B), 2.08 (s) and 2.07 (s) and 2.06 (s) sum 6H in A and B, 1.17 (d, *J* = 6.7 Hz, 3H in A and 3H in B).^{13}C{^{1}H} NMR ($CDCl_3$): *δ* 143.62, 143.13, 140.74, 139.86, 139.63, 139.24, 138.95, 138.81, 138.47, 138.38, 136.45, 136.30, 136.20, 135.96, 135.90, 129.55, 129.40, 128.56, 128.39, 128.23, 128.06, 128.00, 126.91, 125.24, 125.13, 91.29, 86.11, 56.53, 56.46, 40.44, 39.52, 38.29, 38.20, 20.99, 20.95, 20.90, 19.02, 13.65.

5-mesityl-2-methyl-7-phenyl-1H-indene

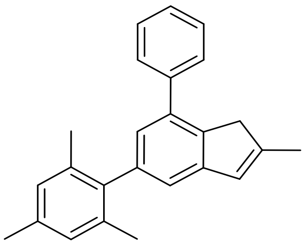

To a solution of 27.8 g (78.0 mmol) of 6-mesityl-1-methoxy-2-methyl-4-phenylindanein 250 ml of toluene 250 mg of TsOH was added. The resulting solution was refluxed using Dean-Stark head for 10 min, then additional 100 mg of TsOH was added, and the reaction mixture was refluxed using Dean-Stark head for another 10 min. Further on, it was cooled to room temperature and washed by 100 ml of 10% K_2CO_3. The organic layer was separated, and the aqueous layer was extracted with 150 ml of dichloromethane. The combined organic extract was evaporated under reduced pressure, and the product was isolated by flash-chromatography on silica gel 60 (40-63 µm; eluent: hexanes, then hexanes-dichloromethane = 10:1, vol.). This procedure gave 23.5 g (93%) of the title product as a slightly yellowish thick oil. ^{1}H NMR ($CDCl_3$): δ 7.56 (d, *J* = 7.3 Hz, 2H), 7.42 (dd, *J* = 7.3 Hz, 2H), 7.32 (t, *J* = 7.3 Hz, 1H), 7.02 (s, 1H), 6.94 (s, 3H), 6.54 (m, 1H), 3.45 (s, 2H), 2.32 (s, 3H), 2.15 (s, 3H), 2.08 (s, 6H). ^{13}C{^{1}H} NMR ($CDCl_3$): δ 146.65, 146.57, 141.28, 139.90, 139.45, 138.82, 137.10, 136.28, 136.11, 128.41, 128.34, 127.97, 127.27, 126.96, 125.26, 119.93, 42.67, 21.00, 20.89, 16.72.

bis(6-mesityl-2-methyl-4-phenyl-1H-inden-1-yl)(dimethyl)silane

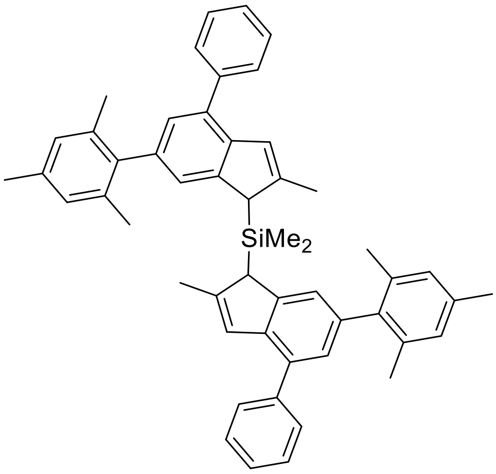

To a solution of 22.7 g (70.0 mmol) of 5-mesityl-2-methyl-7-phenyl-1*H*-indene in 300 ml of ether 28.8 ml (70.0 mmol) of 2.43 M ^{n}BuLi in hexanes was added in one portion at -40 °C. This mixture was stirred for 3.5 h at room temperature, then the resulting bright red solution was cooled to -40°C (a lot of yellow precipitate fell out that does not dissolve when the temperature reached -20 °C), and 280 mg of CuCN was added. The resulting mixture was stirred for 30 min at -25 °C, then 4.52 g (35.0 mmol) of dichlorodimethylsilane was added in one portion. Further on, this mixture was stirred overnight at ambient temperature, then filtered through a short pad of silica gel 60 (40-63 µm) which was additionally washed by 2 x 50 ml of dichloromethane. The combined filtrate was evaporated under reduced pressure, and the residue was dried in vacuum at elevated temperature. This procedure gave 24.6 g (ca. 100%) of the title product (ca. 85% purity by NMR, mixture of *meso* and *rac* isomers isomers in 1.5:1 molar ratio) as yellowish glass which was used further without additional purification. HRMS: [M]$^+$ calc. for $C_{52}H_{52}Si$: 704.3838; found: 704.3856.^{1}H NMR ($CDCl_3$): δ 7.60–7.52 (m, 4H in *rac* and *meso*), 7.47–7.29 (m, 8H in *rac* and *meso*), 7.13 (br.s, 2H in *meso*), 7.08 (d, *J* = 1.1 Hz, 2H in *rac*), 7.06 (d, *J* = 1.1 Hz, 2H in *meso*), 6.97–6.91 (m, 2H in *meso* and 4H in *rac*), 6.85 (br.s, 2H in *rac* and *meso*), 3.90 (s, 2H in *rac*), 3.85 (s, 2H in *meso*), 2.32 (s) and 2.22 (br.s) and 2.13 (br.s) and 2.10 (s) and 2.07 (s) and 2.06 (br.s) sum 24H in *rac* and *meso*, –0.15 (s, 3H in *meso*), –0.21 (s, 3H in *rac*), –0.23 (s, 6H in *meso*).

rac-dimethylsilanediylbis[η[5]-6-mesityl-2-methyl-4-phenylinden-1-yl]zirconium dichloride (M27)

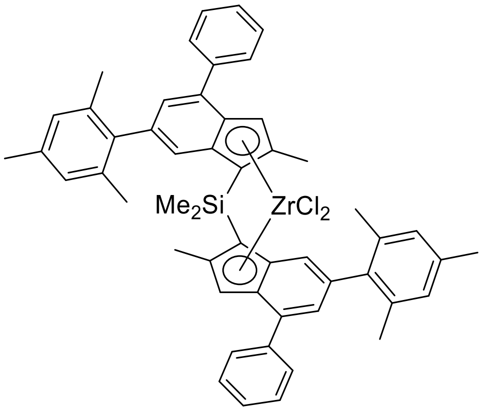

To a cooled to –60 °C solution of 14.8 g (20.99 mmol) of bis(6-mesityl-2-methyl-4-phenyl-1*H*-inden-1-yl)(dimethyl)silanein 170 ml of ether 17.3 ml (42.0 mmol) of 2.43 M ^{n}BuLi in hexanes was added in one portion. This mixture was stirred overnight at room temperature. The resulting red solution was cooled to -60 °C, and 4.9 g (21.03 mmol) of $ZrCl_4$ was added. The reaction mixture was stirred for 24 h, then evaporated to dryness, the residue was heated with 200 ml of toluene, and thus obtained hot suspension was filtered through glass frit (G4). The filtrate was evaporated to ca. 70 ml. Yellow solid precipitated overnight at room temperature was filtered off and dried in vacuum to give 5.20 g (27%) of *rac*-complex (2:1 solvate with toluene). The mother liquor was evaporated to ca. 50 ml. Yellow solid precipitated overnight at room temperature was filtered off and then dried in vacuum to give 3.60 g (19%) of *rac*-complex (2:1 solvate with toluene). Finally, the mother liquor was evaporated to ca. 20 ml, and 40 ml of n-hexane was added. Yellow solid precipitated from this solution at ambient temperature was filtered off (G3) and dried in vacuum to give 1.15 g (6%) of *rac*-complex (2:1 solvate with toluene). Thus, the total yield of *rac*-complex (as 2:1 solvate with toluene) isolated in this synthesis was 9.95 g (52%). Anal. calc. for $C_{52}H_{50}Cl_2SiZr{\cdot}0.5PhMe$: C, 73.15; H, 5.97. Found: C, 73.24; H, 6.08. ^{1}H NMR ($CDCl_3$): δ 7.72–7.65 (m, 2H), 7.46–7.38 (m, 3H), 7.37–7.30 (m, 1H), 7.28–7.22 (m, PhMe), 7.20 (d, *J* = 1.0 Hz, 1H), 7.19–7.12 (m, PhMe), 7.06 (s, 1H), 6.96 (s, 2H), 2.35 (s, PhMe), 2.34 (s, 3H), 2.24 (s, 3H), 2.12 (s, 3H), 2.05 (s, 3H), 1.23 (s, 3H). ^{13}C{^{1}H} NMR ($CDCl_3$, resonances of the cocrystallized toluene were omitted): δ 139.53, 139.27, 138.81, 138.52, 136.96, 136.87, 136.13, 135.22, 131.24, 129.02, 128.75, 128.67, 128.64, 128.32, 127.98, 127.80, 123.82, 122.23, 84.24, 21.19, 21.04 (two resonances), 18.89, 2.51.

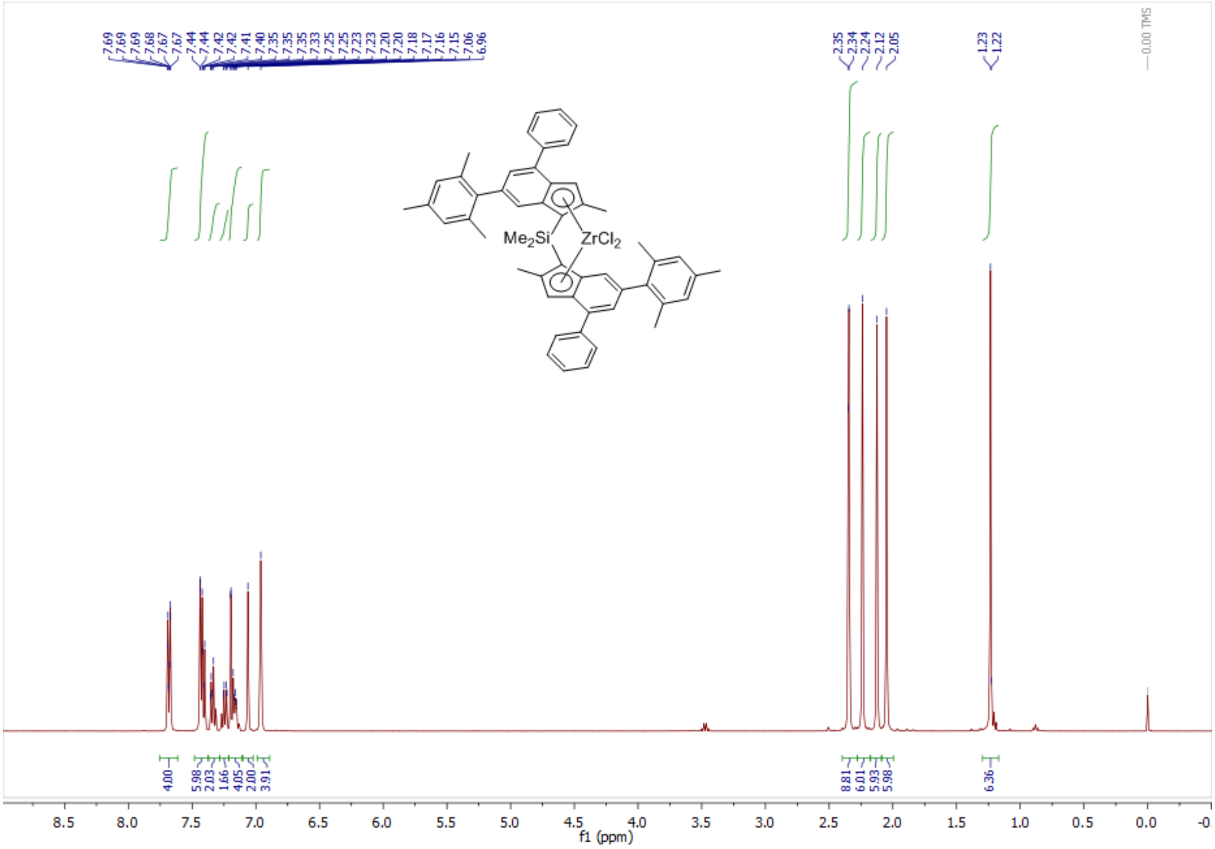

Figure S13. ^{1}H NMR spectrum of **M27** in $CDCl_3$.

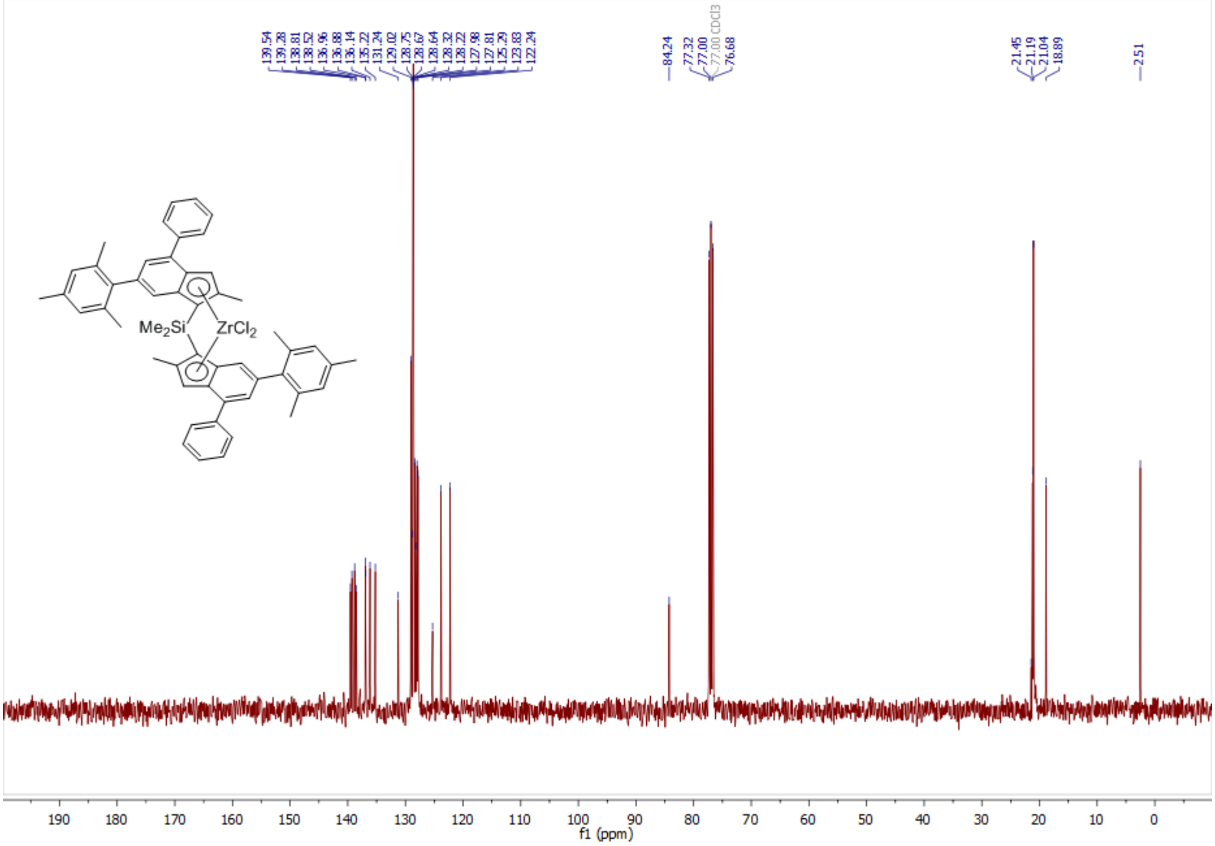

Figure S14. $^{13}C\{^{1}H\}$ NMR spectrum of **M27** in $CDCl_3$.

M28

2,7-dimethyl-5-tert-butyl-indan-1-one

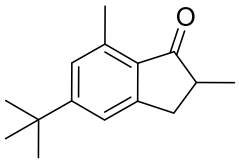

To a solution of diisopropylamine (6.92 ml, 49.4 mmol) in THF (200 ml) *n*BuLi (19.8 ml 2.5M in hexanes, 49.4 mmol) was added at –80°C. The reaction mixture was stirred for 10 min at room temperature, and a solution of 7-methyl-5-*tert*-butyl-indan-1-one (10.0 g, 49.4 mmol) in THF (200 ml) was added dropwise for 30 min. To the resulting solution HMPA (42.6 ml, 247 mmol) and iodomethane (15.4 ml, 247 mmol) were subsequently added. The reaction mixture was stirred overnight at room temperature. Water (700 ml) and dichloromethane (300 ml) were then added to the reaction mixture. The organic layer was separated, and the aqueous layer was extracted with 400 ml of dichloromethane. The combined organic phase was evaporated to dryness. The product was isolated by flash-chromatography on silica gel 60 (40-63 µm; eluent: hexanes-ethyl acetate; 15:1, vol.). This procedure gave 8.10 g (76%) of the title product as a colorless oil. ^{1}H NMR ($CDCl_3$): δ 7.24 (br.s, 1H), 7.12 (br.s, 1H), 3.31 (dd, J = 17.9, J = 8.8 Hz, 1H), 2.69–2.59 (m, 2H), 2.62 (s, 3H), 1.33 (s, 9H), 1.29 (d, J = 7.2 Hz, 3H).

6-tert-butyl-2,3,4-trimethyl-1H-indene

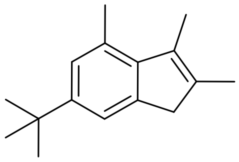

To a solution of 2,7-dimethyl-5-*tert*-butyl-indan-1-one (8.10 g, 37.4 mmol) in diethyl ether (200 ml) methylmagnesium bromide (25.8 ml of 2.9M in ether, 74.9 mmol) was added dropwise at room temperature. The resulting mixture was stirred overnight at room temperature, then it was accurately poured into 400 ml of saturated aqueous NH_4Cl solution. The organic layer was separated, and the aqueous layer was extracted with 100 ml of diethyl ether. The combined organic phase was evaporated to dryness. To the resulting oil 200 ml of toluene and 500 mg of *p*-toluenesulfonic acid were added. Further on, this mixture was stirred for 30 min at 60 °C, then filtered through a short pad of silica gel 60 (40-63 µm) which was additionally washed by 2x20 ml of toluene. The combined filtrate was evaporated to dryness, and the residue was purified by flash chromatography on silica gel 60 (40-63 µm, eluent: hexanes). Yield 7.27 g (91%) of the product as a colorless solid. ^{1}H NMR ($CDCl_3$): δ 7.25 (br.s, 1H), 7.01 (br.s, 1H), 3.21 (br.s, 2H), 2.59 (s, 3H), 2.21 (m, 3H), 2.02 (br.s, 3H), 1.33 (s, 9H).

bis(6-tert-butyl-2,3,4-trimethyl-1H-inden-1-yl)dimethylsilane

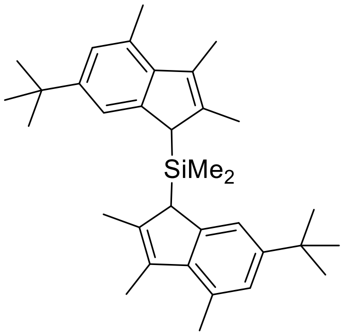

To a solution of 7.27 g (33.9 mmol) of 6-*tert*-butyl-2,3,4-trimethyl-1*H*-indene in 130 ml of ether 13.6 ml (33.9 mmol) of 2.5 M^{n}BuLi in hexanes was added in one portion at -40 °C. This mixture was stirred overnight at room temperature, then the resulting light-orange solution was cooled to -80°C, and 50 mg of N-methylimidazole was added. The resulting mixture was stirred for 5 min at -80 °C, then 2.19 g (17.0 mmol) of dichlorodimethylsilane was added in one portion. Further on, this mixture was stirred overnight at ambient temperature, then filtered through a short pad of silica gel 60 (40-63 µm) which was additionally washed by 2 x 30 ml of dichloromethane. The combined filtrate was evaporated under reduced pressure, and the residue was purified by flash chromatography on silica gel 60 (40-63 µm, eluent: hexane:dichloromethane; 10:1, vol.). Yield 4.75 g (58%) of the product as a yellowish foam. According to ^{1}H NMR, the product was a mixture of *rac*- and *meso*-isomers in ca. 1:6 molar ratio. Anal. calc. for $C_{34}H_{48}Si$: C, 84.23; H, 9.98. Found: C, 84.15; H, 10.14.^{1}H NMR ($CDCl_3$): δ 7.39 (m, 2H in *rac*), 7.20 (m, 2H in *meso*), 6.99 (m, 2H in *rac*), 6.98 (m,

2H, *meso*), 3.46 (br.s, 2H, in *rac* and *meso*), 2.63 (s, 3H in *rac*), 2.61 (s, 3H in *meso*), 2.27 (m, 3H in *meso*), 2.20 (m, 3H in *rac*), 2.07 (br.s, 3H in *meso*), 1.88 (br.s, 3H in *rac*), 1.31 (s, 18H in *rac*), 1.30 (s, 18H in *meso*), –0.24 (s, 3H in *meso* and 6H in *rac*), –0.45 (s, 3H in *meso*).

***rac*-dimethylsilanediylbis[η^5-2,3,4-trimethyl-6-tert-butyl-inden-1-yl]zirconium dichloride (M28)**

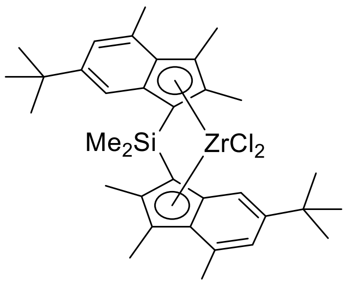

To a cooled to –40 °C solution of 2.00 g (4.12 mmol) of bis(2,3,4-trimethyl-6-*tert*-butyl-1*H*-inden-1-yl)dimethylsilane in 150 ml of ether 3.30 ml (8.25 mmol) of 2.5 M ^{n}BuLi in hexanes was added in one portion. This mixture was stirred overnight at room temperature. The resulting light-yellow solution was cooled to -80 °C, and 960 mg (4.12 mmol) of $ZrCl_4$ was added. The reaction mixture was stirred for 24 h, and then evaporated to dryness. The residue was heated with 150 ml of toluene, and the formed hot suspension was filtered through glass frit (G4). The filtrate was evaporated to dryness giving 2.10 g of a 1:1 mixture of *rac*- and *meso*-complexes. This crude product was recrystallized from methylcyclohexane/hexane mixture (1:1, vol.) yielding 770 mg (29%) of *rac*-complex as an yellow powder. *Rac*-zirconocene. Anal. calc. for $C_{34}H_{46}Cl_2SiZr$: C, 63.32; H, 7.19. Found: C, 63.42; H, 7.28. ^{1}H NMR ($CDCl_3$): δ 7.32 (br.s, 2H), 7.04 (br.s, 2H), 2.51 (s, 6H), 2.34 (s, 6H), 1.95 (s, 6H), 1.29 (s, 6H), 1.24 (s, 18H).^{13}C{^{1}H} NMR (CD_2Cl_2): δ 148.8, 136.9, 134.0, 131.9, 129.4, 128.8, 128.3, 119.0, 80.5, 36.1, 31.9, 23.1, 17.2, 15.7, 5.1.

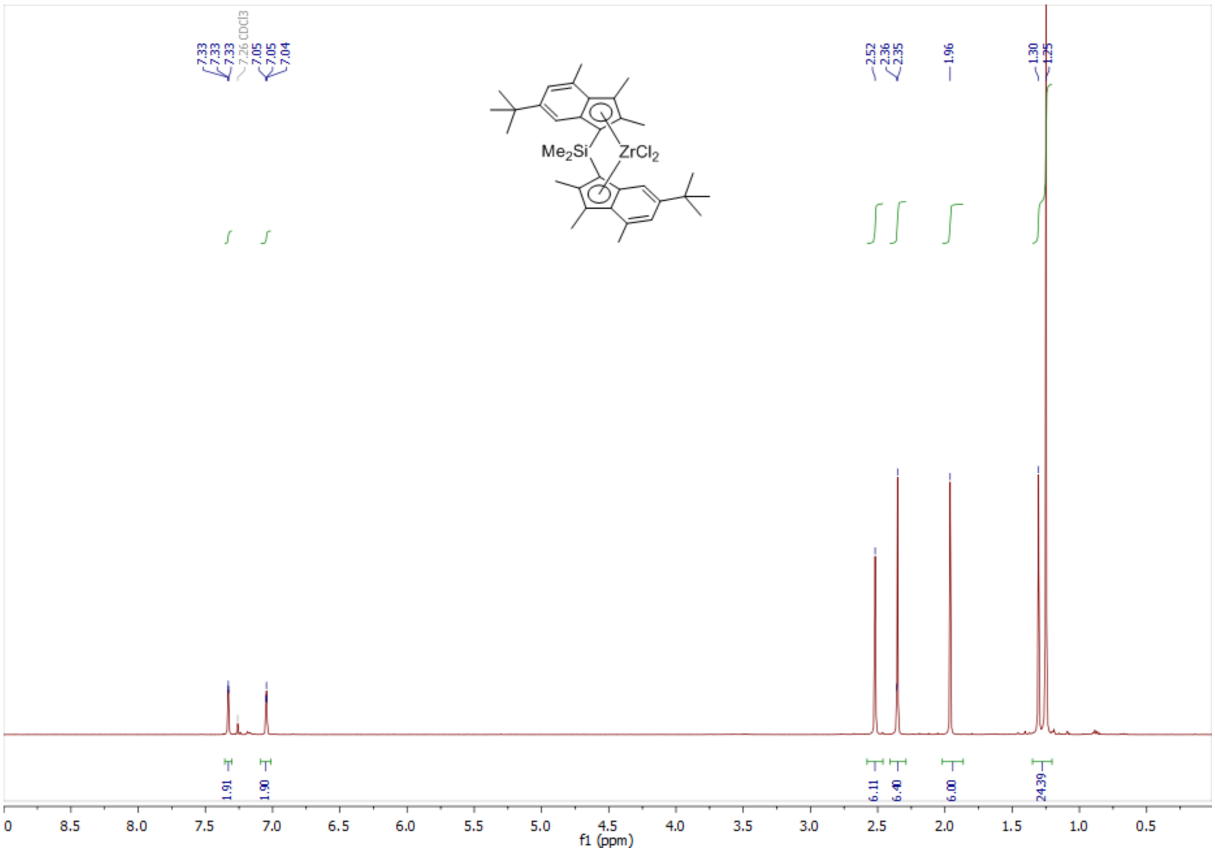

Figure S15.^{1}H NMR spectrum of **M28** in $CDCl_3$.

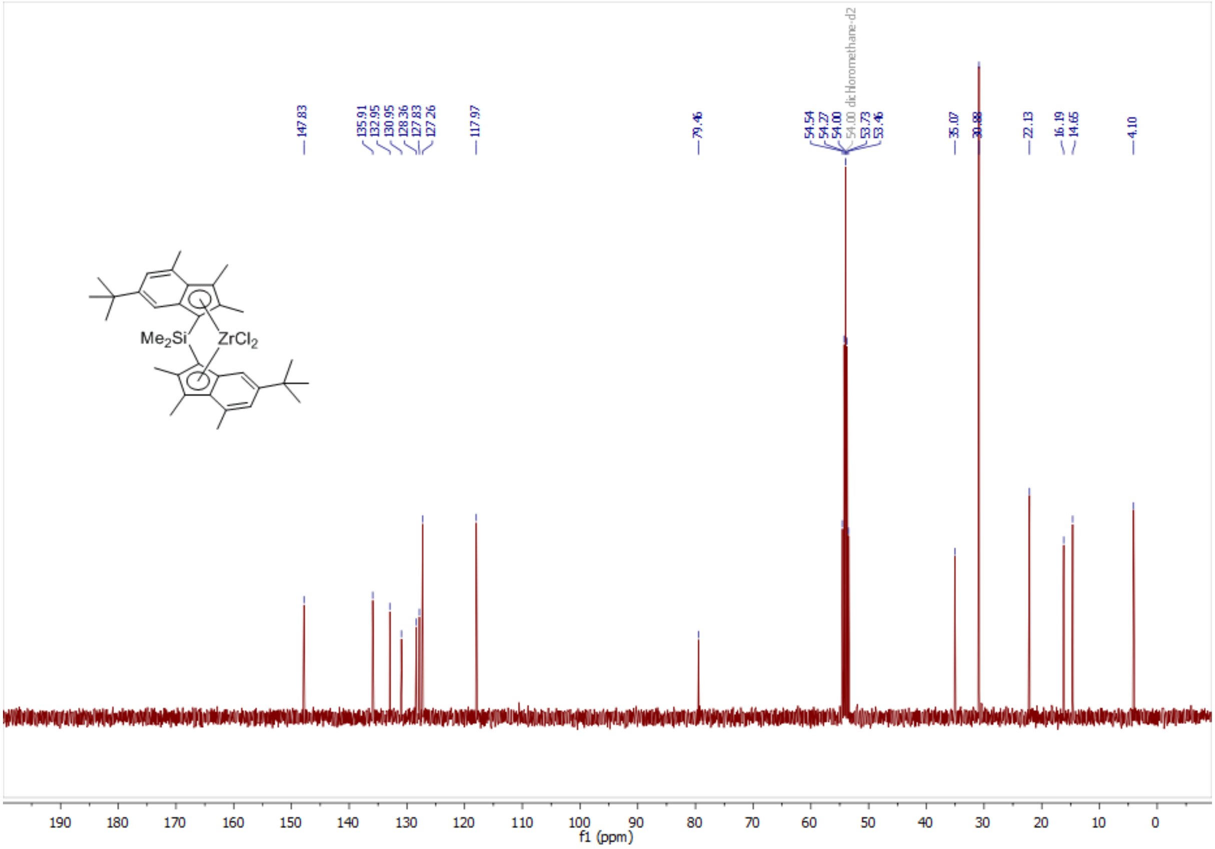

Figure S16.$^{13}C\{^1H\}$ NMR spectrum of **M28** in CD_2Cl_2.

M31

bis(6-tert-butyl-2-methyl-4-bromo-1H-inden-1-yl)dimethylsilane

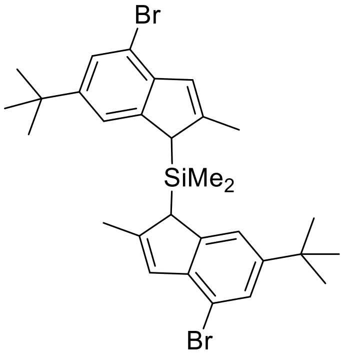

To a solution of 5.00 g (18.8 mmol) of 7-bromo-5-*tert*-butyl-2-methyl-1*H*-indene in 130 ml of ether 7.55 ml (18.8 mmol) of 2.5 M ^{n}BuLi in hexanes was added in one portion at -40 °C. This mixture was stirred overnight at room temperature, then cooled to -80°C, and 30 mg of N-methylimidazole was added. The resulting mixture was stirred for 5 min at -80 °C, and then 1.21 g (9.40 mmol) of dichlorodimethylsilane was added in one portion. Further on, this mixture was stirred overnight at ambient temperature, then filtered through a short pad of silica gel 60 (40-63 µm) which was additionally washed by 2 x 30 ml of dichloromethane. The combined filtrate was evaporated under reduced pressure, and the residue was purified by flash chromatography on silica gel 60 (40-63 µm, eluent: hexane:dichloromethane; 10:1, vol.). Yield 4.85 g (88%) of the product as yellowish foam. According to ^1H NMR, the product was a mixture of *rac*- and *meso*-isomers in ca. 1:2.5 molar ratio. Anal. calc. for $C_{30}H_{38}Br_2Si$: C, 61.43; H, 6.53. Found: C, 61.80; H, 6.71.^1H NMR ($CDCl_3$): δ 7.40 (m, 2H in *rac*), 7.37 (d, *J* = 1.3 Hz, 2H in *rac*), 7.36 (d, *J* = 1.3 Hz, 2H in *meso*), 7.26 (m, 2H in *meso*), 6.65 (br.s, 2H in *meso*), 6.61 (br.s, 2H in *rac*), 3.68 (br.s, 2H in *rac*), 3.64 (br.s, 2H in *meso*), 2.18 (d, *J* = 0.9 Hz, 3H in *meso*), 2.14 (d, *J* = 0.9 Hz, 3H in *rac*), 1.29 (br.s, 18H in *rac* and *meso*), –0.17 (s, 3H in *meso*), –0.24 (s, 6H in *rac*), –0.30 (s, 3H in *meso*).

rac-dimethylsilanediylbis(η[5]-2-methyl-4-bromo-6-tert-butyl-inden-1-yl)dimethylzirconium (M31)

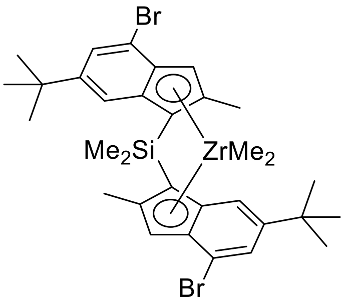

To a cooled to –40 °C solution of 4.85 g (8.27 mmol) of bis(2-methyl-4-bromo-6-*tert*-butyl-1*H*-inden-1-yl)dimethylsilane in 150 ml of ether 6.61 ml (16.5 mmol) of 2.5 M ^{n}BuLi in hexanes was added in one portion. This mixture was stirred overnight at room temperature, then cooled to -80 °C, and 4.39 g (8.27 mmol) of $[PhN(CH_2)_3NPh]ZrCl_2(THF)_2$ was added. The reaction mixture was stirred for 24 h, and then evaporated to dryness. The residue was heated with 150 ml of toluene, and the formed hot suspension was filtered through glass frit (G4). The filtrate was evaporated to dryness, the residue was dissolved in 140 ml of diethyl ether/dichloromethane (1:1, vol.). This solution was cooled to -110C, and 9.58 ml (14.8 mmol, 1.54 M) of HCl in diethyl ether was carefully added. The resulting yellow suspension was stirred overnight at room temperature and then evaporated to dryness. The residue was recrystallized from toluene yielding 970 mg (18%) of a 10:1 mixture of *rac*- and *meso*-zirconocene dichlorides. This solid was dissolved in a 40 ml of toluene, and 1.00 ml (2.7 mmol, 2.7 M) of MeMgBr in diethyl ether was added. The reaction mixture was stirred at 90 °C overnight and then evaporated to dryness. The residue was extracted with 2 x 20 ml of hot toluene, the combined extract was filtered through a thin pad of Celite 503, and the filtrate was evaporated to dryness. The crude product was recrystallized from hexane yielding 150 mg of pure dimethylated *rac*-complex as a light-yellow powder. Anal. calc. for $C_{32}H_{42}Br_2SiZr$: C, 54.46; H, 6.00. Found: C, 55.12; H, 6.11. ^{1}H NMR ($CDCl_3$): δ 7.52 (d, J = 1.3 Hz, 2H), 7.38 (m, 2H), 6.77 (s, 2H), 2.06 (s, 6H), 1.25 (s, 18H), 1.08 (s, 6H), –1.16 (s, 6H).^{13}C{^{1}H} NMR ($CDCl_3$): δ 150.9, 136.5, 133.1, 131.1, 129.4, 122.9, 120.9, 120.5, 86.6, 36.6, 31.9, 20.0, 3.7.

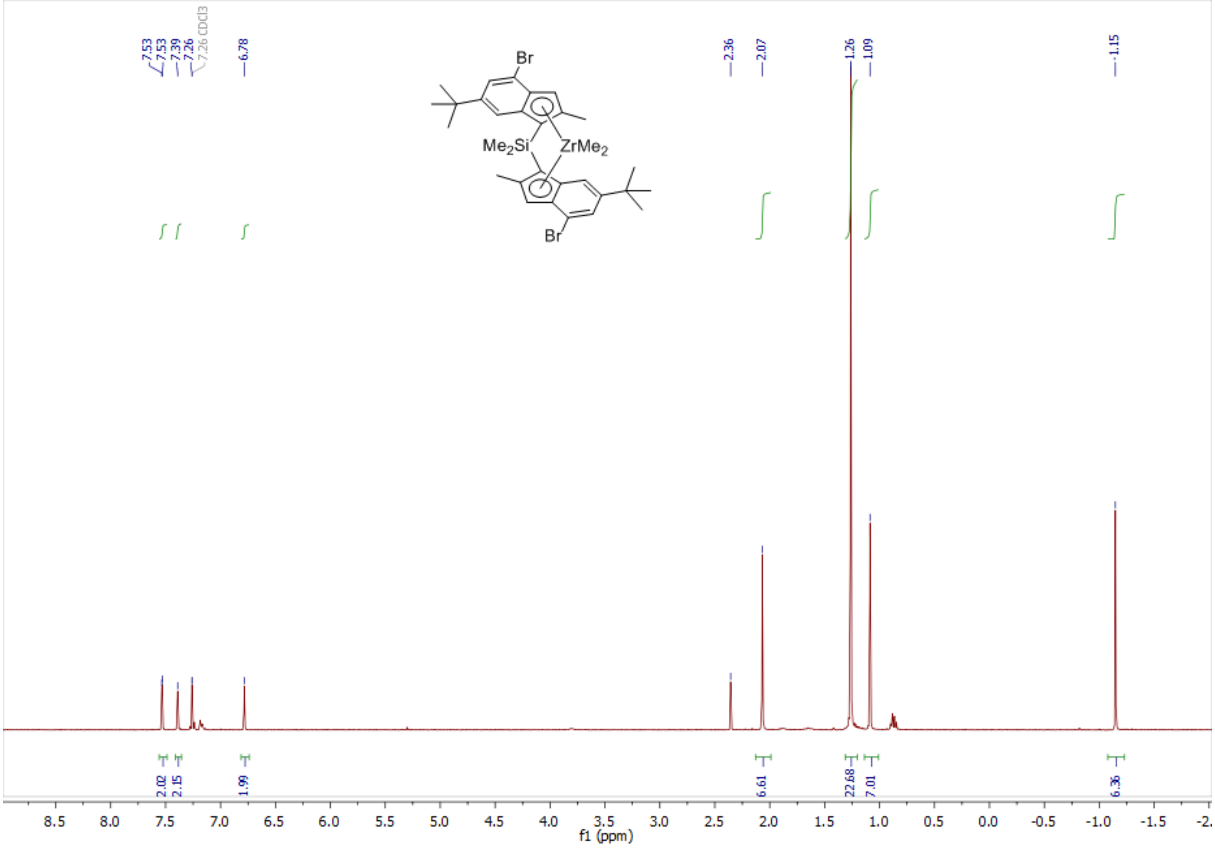

Figure S17.^{1}H NMR spectrum of **M31** in $CDCl_3$.Traces of toluene are visible at 2.36 and 7.13–7.26 ppm.

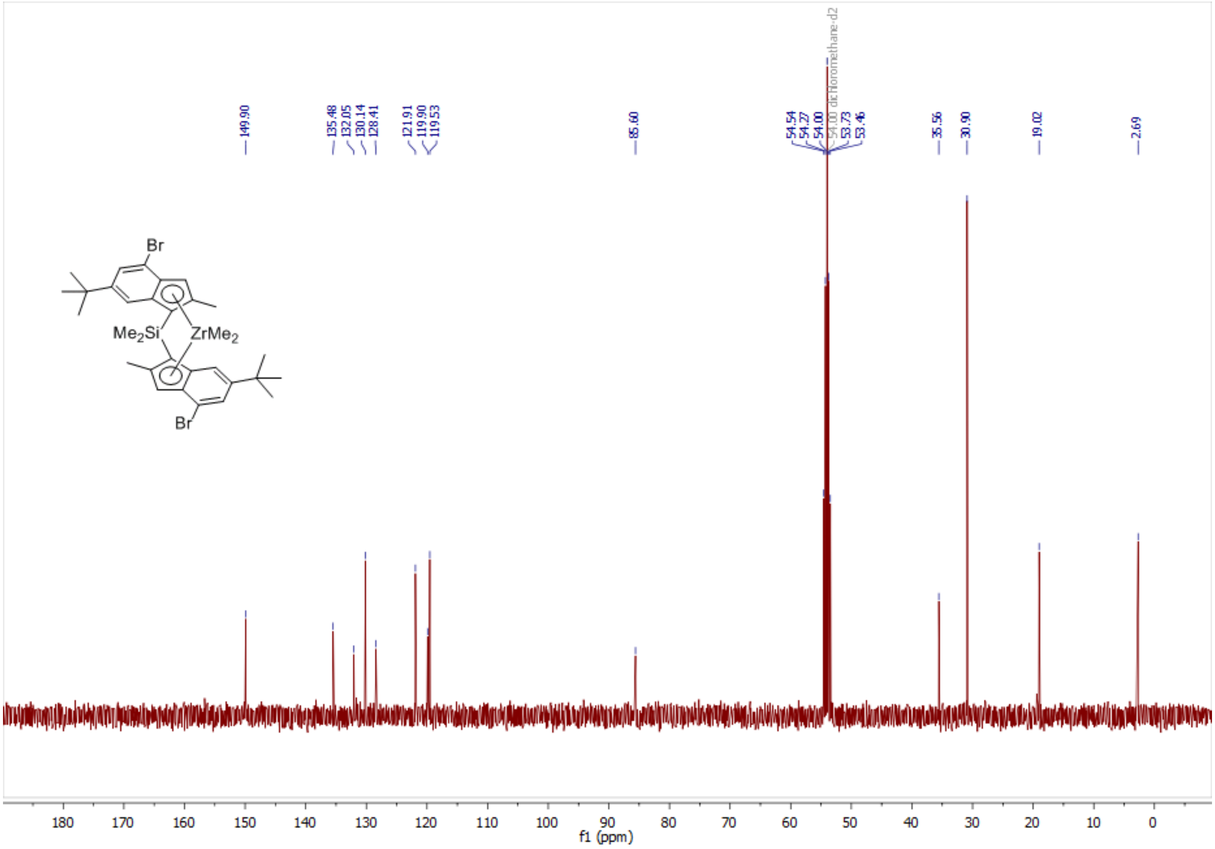

Figure S18.$^{13}C\{^{1}H\}$ NMR spectrum of **M31** in CD_2Cl_2.

M32

5/6-tert-butyl-2-methyl-7/4-(2-thienyl)-1H-indene

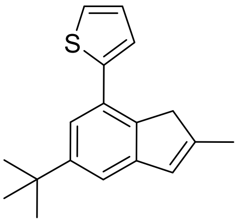

To a mixture of 4.00 g (2.1 mol.%) of $NiCl_2(PPh_3)$IPr and 65.0 g (245.1 mmol) of 7-bromo-5-*tert*-butyl-2-methyl-1*H*-indene a solution of 2-thienylmagnesium bromide in THF obtained from 71.4 g (438 mmol) of 2-bromothiophene, 11.7 g (482 mmol) of magnesium turnings, and 300 ml of THF was added in one portion. The resulting solution was refluxed for 40 min, then cooled to room temperature, 1200 ml of water and 1000 ml of ether were added followed by 500 ml of 1 M HCl. The organic layer was separated, and the aqueous layer was extracted with 500 ml of ether. The combined organic extract was evaporated to dryness to give a red oil. The product was isolated by flash-chromatography on silica gel 60 (40-63 µm; eluent: hexanes-dichloromethane = 10:1, vol.) followed by distillation in vacuo, b.p. 188 °C/7 mm Hg. This procedure gave 12.7 g (19%) of a ca. 65:35 mixture of 5-*tert*-butyl-2-methyl-7-(2-thienyl)-1*H*-indene and 6-*tert*-butyl-2-methyl-4-(2-thienyl)-1*H*-indene as yellowish thick oil. ^{1}H NMR ($CDCl_3$): δ 7.40 (d, J = 1.7 Hz), 7.38 (s), 7.55 (dd, J = 3.7 Hz, J = 1.1 Hz), 7.32–7.28 (2dd), 7.27 (d, J = 1.7 Hz), 7.22 (dd, J = 3.6 Hz, J = 1.1 Hz, sum 4H), 7.13–7.08 (2dd, 1H), 6.85 and 6.50 (2m, sum 1H), 3.46 and 3.33 (2m, sum 2H), 2.16 and 2.15 (2m, sum 3H), 1.37 and 1.36 (2s, sum 9H). $^{13}C\{^{1}H\}$ NMR ($CDCl_3$): δ 150.27, 147.04, 146.89, 146.43, 146.32, 144.26, 144.02, 143.93, 140.71, 136.87, 129.08, 127.39, 127.32, 127.23, 126.05, 124.95, 124.51, 124.44, 124.38, 123.58, 120.30, 120.08, 116.57, 43.41, 42.98, 34.68, 34.56, 31.58, 16.95, 16.77.

rac-dimethylsilanediylbis[η^5-6-tert-butyl-2-methyl-4-(2-thienyl)-1H-inden-1-yl]zirconium dichloride (M32)

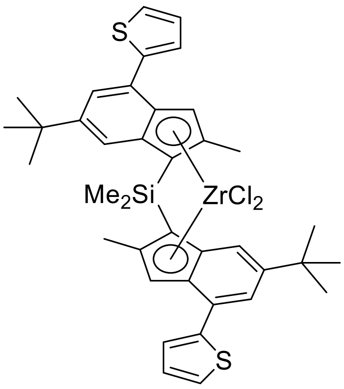

To a solution of 11.9 g (44.3 mmol) of 5-*tert*-butyl-2-methyl-7-(2-thienyl)-1*H*-indene in 200 ml of ether 18.2 ml (44.2 mmol) of 2.43 M ^{n}BuLi in hexanes was added in one portion at –60 °C. This mixture was stirred overnight at room temperature, then the obtained suspension was cooled to -40°C, and 2.86 g (22.16 mmol) of dichlorodimethylsilane was added in one portion. Further on, this mixture was stirred overnight at ambient temperature, then cooled to -40 °C, and 18.2 ml (44.2 mmol) of 2.43 M ^{n}BuLi in hexanes was added in one portion. This mixture was stirred for 5.5 h at room temperature, then cooled to -60 °C, and 5.16 g (22.1 mmol) of $ZrCl_4$was added. The obtained mixture was stirred overnight at room temperature, then evaporated to dryness, and the residue was heated with 200 ml of toluene, and thus obtained hot suspension was filtered through glass frit (G4). The obtained filtrate was evaporated to dryness, and the residue was crystallized from a mixture of toluene and n-hexane giving 5.70 g (34%) of pure *rac*-material. Anal. calc. for $C_{38}H_{42}Cl_2S_2SiZr$: C, 60.60; H, 5.62. Found: C, 61.03; H, 5.70. ^{1}H NMR ($CDCl_3$): δ 7.58–7.54 (m, 2H), 7.40 (dd, *J* = 3.6 Hz, *J* = 1.0 Hz, 1H), 7.28 (dd, *J* = 5.1 Hz, *J* = 1.0 Hz, 1H), 7.09–7.05 (m, 2H), 2.26 (s, 3H), 1.34 (s, 3H), 1.33 (s, 9H). ^{13}C{^{1}H} NMR ($CDCl_3$): δ 148.31, 141.87, 134.96, 131.29, 129.46, 127.98, 127.92, 126.09, 126.01, 125.03, 121.76, 118.97, 83.40, 35.01, 30.74, 18.58, 2.66.

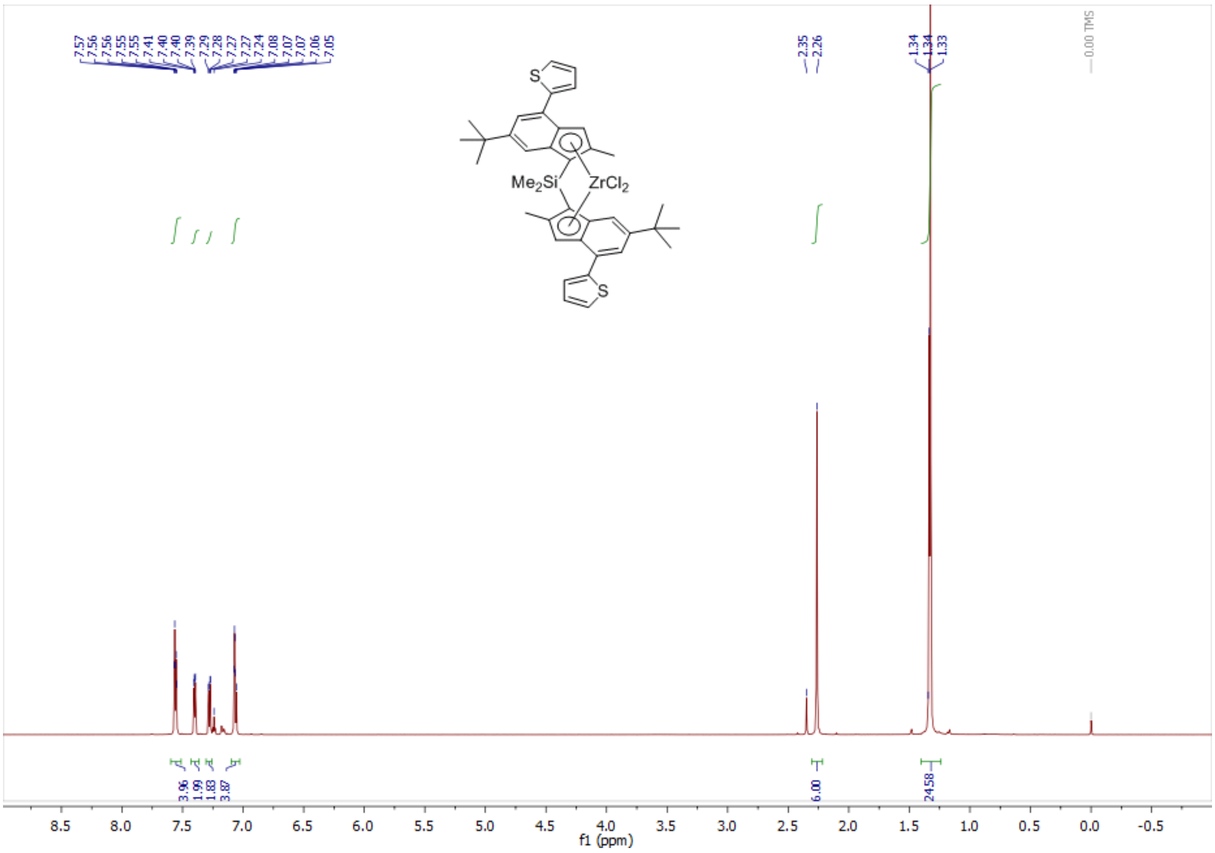

Figure S19. ^{1}H NMR spectrum of **M32** in $CDCl_3$. Traces of toluene are visible at 2.36 and 7.13–7.26 ppm.

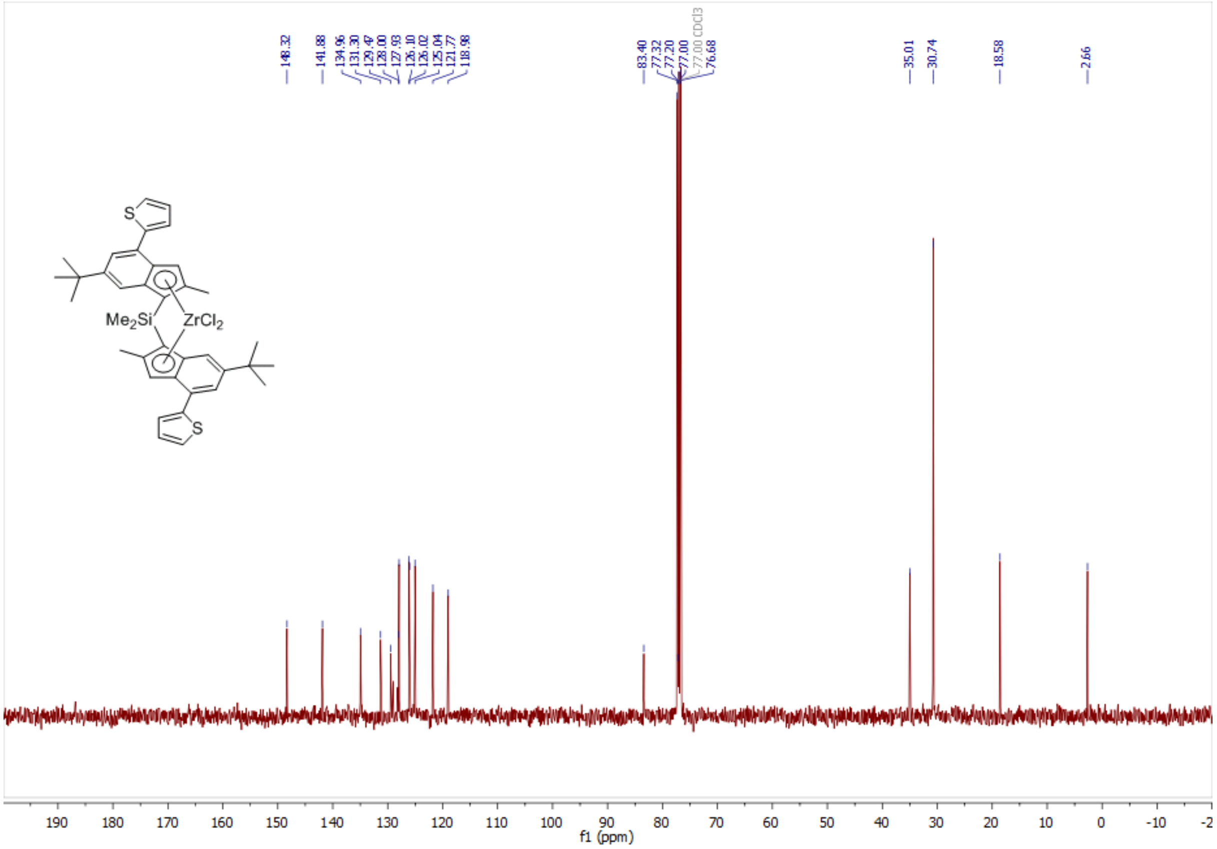

Figure S20. $^{13}C\{^{1}H\}$ NMR spectrum of **M32** in $CDCl_3$.

M33

bis[6-tert-butyl-4-(3,5-di-tert-butylphenyl)-2-methyl-1H-inden-1-yl](dimethyl)silane

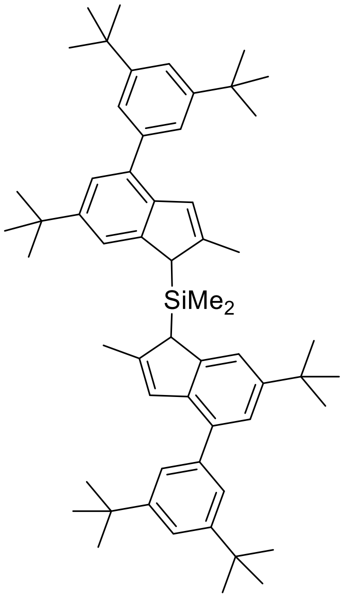

To a solution of 18.7 g (50.0 mmol) of 5-*tert*-butyl-7-(3,5-di-*tert*-butylphenyl)-2-methyl-1*H*-indene in 250 ml of ether 20.6 ml (50.0 mmol) of 2.43 M ^{n}BuLi in hexanes was added in one portion at -50 °C. This mixture was stirred for 6 h at room temperature, then the resulting light-orange solution was cooled to -50°C, and 200 mg of CuCN was added. This mixture was stirred for 30 min at -25 °C, and 3.23 g (25.0 mmol) of dichlorodimethylsilane was added in one portion. Further on, this mixture was stirred overnight at ambient temperature, then filtered through a short pad of silica gel 60 (40–63 µm) which was additionally washed by 2 x 50 ml of dichloromethane. The combined filtrate was evaporated under reduced pressure, and the residue was dried in vacuum at elevated temperature. This procedure gave 20.1 g (99%) of the title product (ca. 85% purity by NMR, a mixture of *meso*- and *rac*-isomers in ~7:3 molar ratio) as yellowish glass, which was used further without additional purification. HRMS: $[M]^+$ calc. for $C_{58}H_{80}Si$: 804.6029; found: 804.6045. ^{1}H NMR ($CDCl_3$): δ 7.65 (s, 2H in *rac*), 7.45–7.31 (m, 10H in *meso*, and 8H in *rac*), 6.78 (br.s, 2H in *rac* and *meso*), 3.82 (s, 2H in *rac*), 3.77 (s, 2H in *meso*), 2.25 (br.s, 3H in *meso*), 2.24 (br.s, 3H in *rac*), 1.41–1.38 (set of singlets, 54H in *rac* and *meso*), –0.15 (s, 3H in *meso*), –0.18 (s, 3H in *meso*), –0.22 (s, 6H in *rac*). $^{13}C\{^{1}H\}$ NMR ($CDCl_3$), the major *meso*-isomer: δ

150.5, 146.9, 146.0, 145.5, 141.0, 140.5, 134.4, 125.8, 123.4, 122.9, 120.6, 119.5, 47.4, 35.0, 31.6 (two resonances), 18.1, –5.26, –5.29.

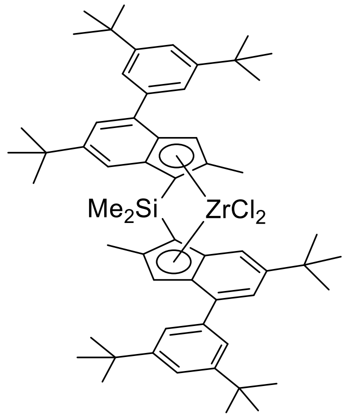

***rac*-dimethylsilanediyl-bis[η^5-6-tert-butyl-4-(3,5-di-tert-butylphenyl)-2-methylinden-1-yl]zirconium dichloride (M33)**

To a cooled to –60 °C solution of 20.0 g (24.8 mmol) of bis[6-*tert*-butyl-4-(3,5-di-*tert*-butylphenyl)-2-methyl-1*H*-inden-1-yl](dimethyl)silanein 250 ml of ether 20.5 ml (49.8 mmol) of 2.43 M ^{n}BuLi in hexanes was added in one portion. This mixture was stirred overnight at room temperature. The resulting light-orange slightly turbid solution was cooled to -60 °C, and 5.79 g (24.9 mmol) of $ZrCl_4$ was added. The reaction mixture was stirred for 24 h, then evaporated to dryness, the residue was heated with 250 ml of toluene, and thus obtained hot suspension was filtered through glass frit (G4). The filtrate was evaporated to ca. 70 ml. Yellow solid precipitated overnight at room temperature was filtered off (G3) and dried in vacuum. This procedure gave 4.00 g (17%) of a ca. 1:2 mixture of *rac*-and *meso*-zirconocenes. The mother liquor was evaporated to ca. 50 ml. Yellow solid precipitated at room temperature was filtered off (G3) and dried in vacuum to give 4.30 g (18%) of pure *rac*-complex. Further on, the mother liquor was evaporated to ca. 40 ml. Yellow solid precipitated overnight at room temperature was filtered off (G3) and dried in vacuum. This procedure gave 3.80 g (16%) of a ca. 85/15 mixture of *rac*-and *meso*-zirconocenes. Finally, the mother liquor was evaporated to the state of viscous oil, and the residue was triturated with 30 ml of n-hexane. The precipitate was filtered off and dried in vacuum. This procedure gave 1.40 g (6%) of *rac*-complex contaminated with a few percent of an unknown impurity. Thus, the total yield of *rac*- and *meso*-zirconocenes isolated in this synthesis was 13.5 g (56%). *Rac*-zirconocene. Anal. calc. for $C_{58}H_{78}Cl_2SiZr$: C, 72.15; H, 8.14. Found: C, 72.32; H, 8.22. ^{1}H NMR ($CDCl_3$): δ 7.59 (s, 1H), 7.62 (d, J = 1.7 Hz, 2H), 7.50 (d, J = 1.3 Hz, 1H), 7.40 (t, J = 1.7 Hz, 1H), 6.87 (s, 1H), 2.25 (s, 3H), 1.35 (2s, 12H), 1.32 (s, 18H). ^{13}C{^{1}H} NMR ($CDCl_3$): δ 150.89, 148.41, 139.01, 134.76, 130.57, 128.03, 126.05, 123.25, 122.36, 121.46, 118.58, 82.55, 35.09, 35.04, 31.53, 30.90, 18.55, 2.79.

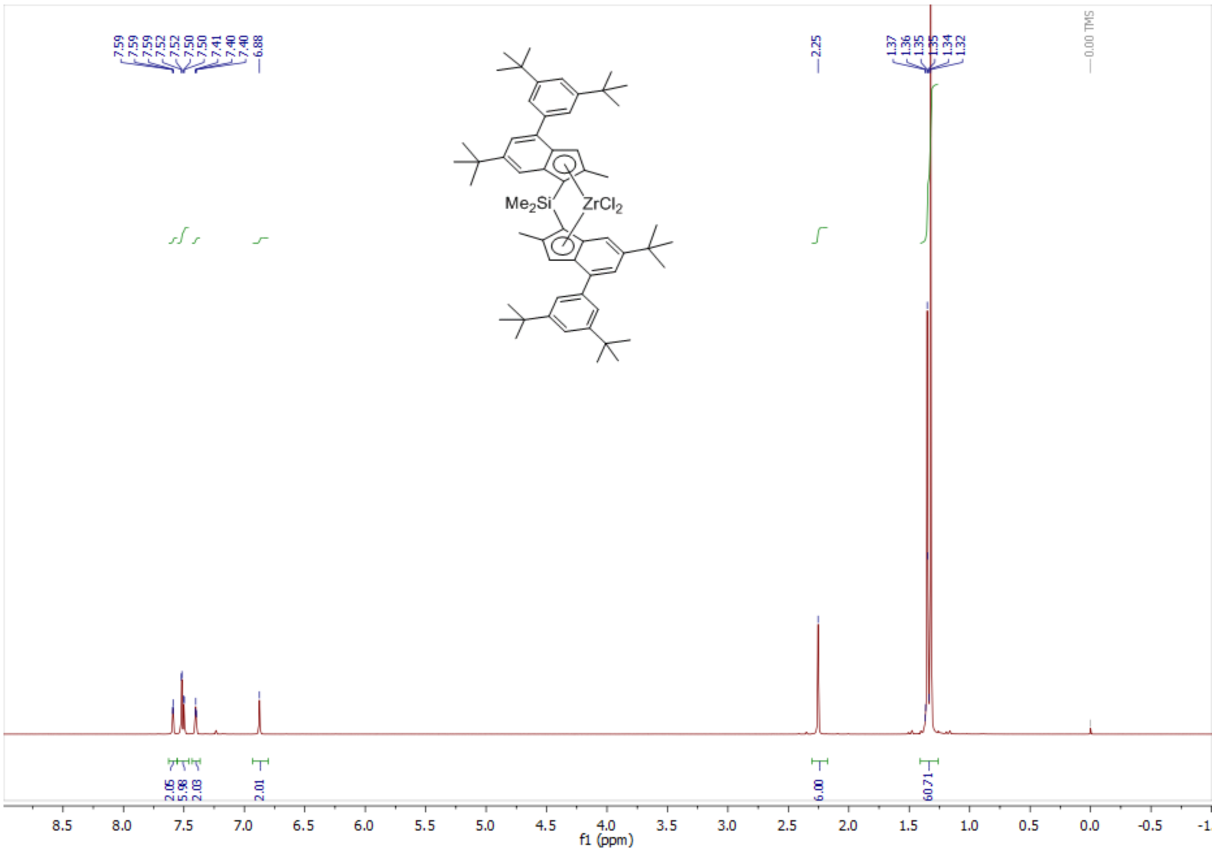

Figure S21.^{1}H NMR spectrum of **M33** in $CDCl_3$.

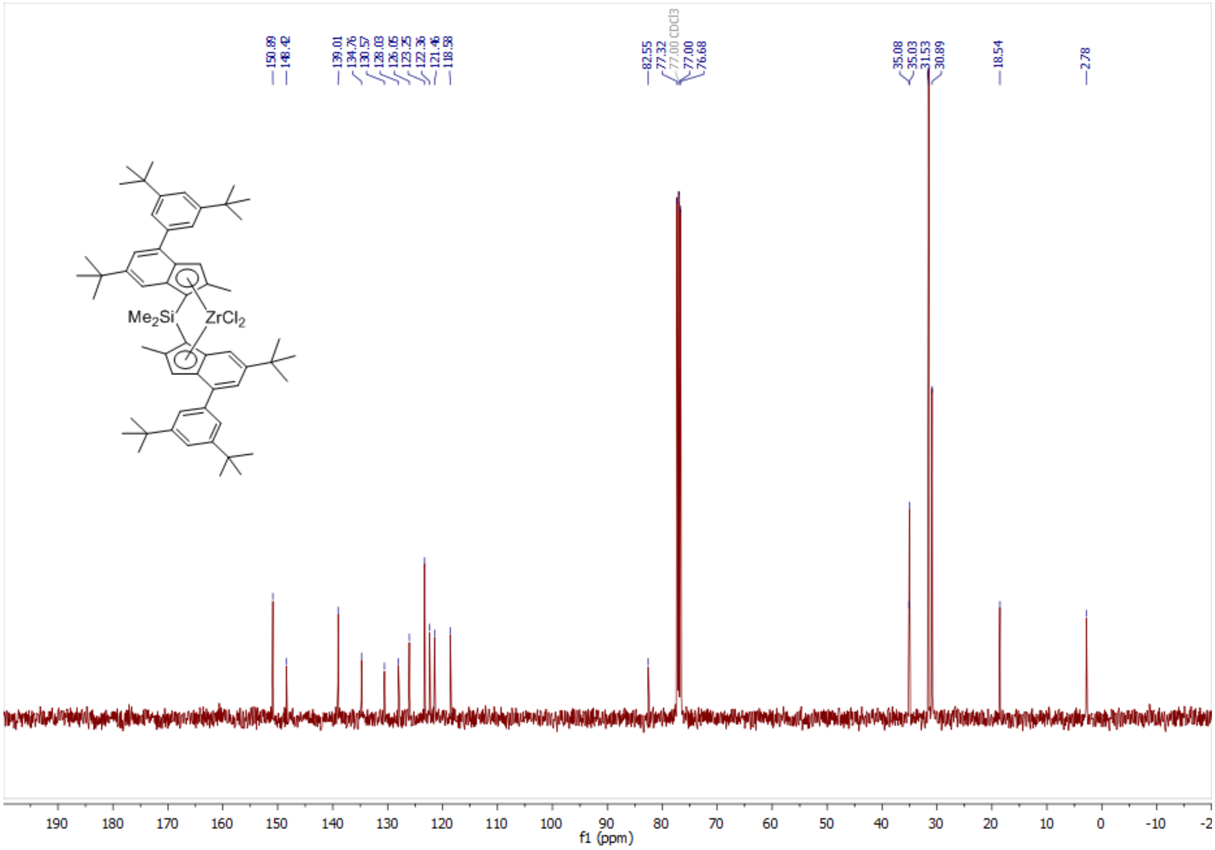

Figure S22.$^{13}C\{^1H\}$ NMR spectrum of **M33** in $CDCl_3$.

M34

6-tert-butyl-4-(2-methylphenyl)-2-methyl-1-methoxyindane

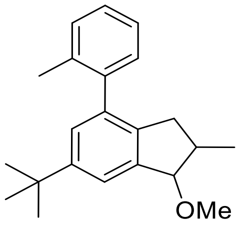

To a mixture of 1.80 g (2.0 mol. %) of $NiCl_2(PPh_3)$IPr and 34.8 g (117 mmol) of 4-bromo-1-methoxy-6-*tert*-butyl-2-methylindan 150 ml (150 mmol) of 1.0 M *o*-tolylmagnesium bromide in THF was added at a such rate to maintain a gentle reflux. The resulting solution was refluxed for 30 min, then cooled to room temperature followed by addition of 300 ml of water and 400 ml of dichloromethane, then 200 ml of 1 M HCl. The organic layer was separated, and the aqueous layer was extracted with 150 ml of dichloromethane. The combined organic extract was evaporated to dryness to give a slightly greenish oil. The product was isolated by flash-chromatography on silica gel 60 (40-63 µm; eluent: hexanes-dichloromethane = 3:1, vol., then 1:1, vol.). This procedure gave 33.1 g (92%) of the title product as a lightly yellowish oily liquid which was a mixture of diastereomers A and b in ~1:1 molar ratio. ^{1}H NMR ($CDCl_3$): δ 7.38 (m, sum 2H in A and B), 7.28–7.12 (m, 10H in A and B), 4.59 (m) and 4.45 (d, J = 4.2 Hz) sum 6H in A and B, 3.53 (br.s) and 3.48 (s) sum 6H in A and B, 3.00–2.32 (m, sum 3H in A and B), 2.12 (s) and 2.11 (s) = sum 6H in A and B, 1.341 (s) and 1.1336 (s) sum 18 H in A and B, 1.14 (br.d, J = 6.7 Hz) and 1.03 (br.d) sum 6H in A and B.

5-tert-butyl-2-methyl-7-(2-methylphenyl)-1H-indene

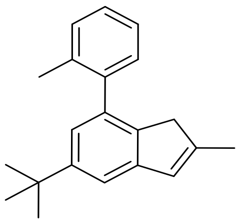

To a solution of 33.1 g (107.3 mmol) of 6-*tert*-butyl-4-(2-methylphenyl)-2-methyl-1-methoxyindanein 300 ml of toluene 250 mg of TsOH was added. The resulting solution was refluxed using Dean-Stark head for 10 min, then additional 200 mg of TsOH was added, and the reaction mixture was refluxed using Dean-Stark head for another 5 min. Then, it was cooled to room temperature, and finally washed by 100 ml of 10% K_2CO_3. The organic layer was separated, and the aqueous layer was extracted with 150 ml of dichloromethane. The combined organic extract was filtered through a short pad of silica gel 60 (40-63 µm) which was additionally washed by 2 x 50 ml of dichloromethane. The combined filtrate was evaporated under reduced pressure, and the residue was distilled in vacuum to give 24.9 g (84%) of the title product, b.p. 160 °C/5 mm Hg, as a slightly yellowish oil. ^{1}H NMR ($CDCl_3$): δ 7.39–7.26 (m, 5H), 7.06 (d, J = 1.7 Hz, 1H), 6.59 (m, 1H), 3.11 (s, 2H), 2.23 (s, 3H), 2.16 (s, 3H), 1.42 (s, 9H). $^{13}C\{^1H\}$ NMR ($CDCl_3$): δ 149.54, 146.38, 145.80, 141.35, 138.74, 136.56, 135.71, 130.05, 129.23, 127.34, 127.10, 125.44, 122.07, 115.69, 41.85, 34.69, 31.68, 20.01, 16.75.

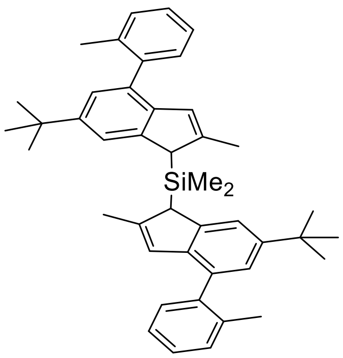

bis[6-tert-butyl-2-methyl-4-(2-methylphenyl)-1H-inden-1-yl](dimethyl)-silane

To a solution of 18.4 g (66.7 mmol) of 5-*tert*-butyl-2-methyl-7-(2-methylphenyl)-1*H*-indenein 250 ml of ether 27.5 ml (66.8 mmol) of 2.43 M ^{n}BuLi in hexanes was added in one portion at -78 °C. This mixture was stirred overnight at room temperature, then the resulting suspension was cooled to -40°C, and 250 mg of CuCN was added. This mixture was stirred for 30 min at -25 °C, then 4.30 g (33.3 mmol) of dichlorodimethylsilane was added in one portion. Thus obtained mixture was stirred overnight at ambient temperature, then filtered through a short pad of silica gel 60 (40-63 µm) which was additionally washed by 2 x 50 ml of dichloromethane. The combined filtrate was evaporated under reduced pressure, and the residue was dried in vacuum at elevated temperature. This procedure gave 20.3 g (ca. 100%) of the title product (ca. 95% purity by NMR, ~2:1 mixture of

diastereomers A and B) as a yellowish glassy solid, which was used further without additional purification. HRMS: $[M]^+$ calc. for $C_{44}H_{52}Si$: 608.3838; found: 608.3822. ^1H NMR ($CDCl_3$): δ7.64 (br.s) and 7.44 (br.s) sum 2H in A and B, 7.28 (8H in A and B), 7.15 (s, 2H in A and 2H in B), 6.31 (br.s, 2H in A and 2H in B), 3.83 (br.s) and 3.75 (br.s) sum 4H in A and B, 2.21 (br.s) and 2.17 (br.s) sum 12H in A and B, 1.36 (s) and 1.35 (s) sum 18H in A and B, –0.21 (br.s) and –0.27 (br.s) sum 6H in A and B.$^{13}C\{^1H\}$ NMR ($CDCl_3$): δ 146.9, 145.9, 145.5, 144.8, 141.4, 141.1, 141.0, 136.1, 133.1, 130.3, 129.9, 127.0, 125.8, 125.6, 123.4, 119.13, 119.08, 47.6, 47.5 (broad), 34.7, 31.8, 20.4. 18.0, 5.7 (broad).

***rac*-dimethylsilanediylbis[η^5-6-tert-butyl-4-(2-methylphenyl)-2-methylinden-1-yl]zirconium dichloride (M34)**

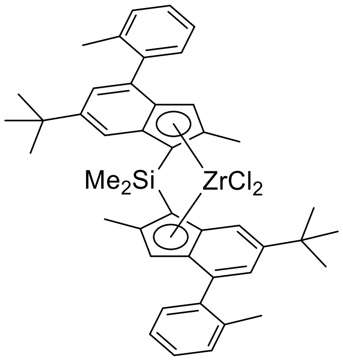

To a cooled to -60 °C solution of 20.3 g (33.4 mmol) of bis[6-*tert*-butyl-4-(2-methylphenyl)-2-methyl-1*H*-inden-1-yl](dimethyl)silanein 250 ml of ether 27.5 ml (66.8 mmol) of 2.43 M ^{n}BuLi in hexanes was added in one portion. This mixture was stirred overnight at room temperature. The resulting light-red solution was cooled to -78 °C, and 7.77 g (33.34 mmol) of $ZrCl_4$ was added. This mixture was stirred for 24 h, then evaporated to dryness, the residue was heated with 200 ml of toluene. Thus obtained hot suspension was filtered through glass frit (G4). The filtrate was evaporated to 50 ml, and 50 ml of n-hexane was added. Yellow solid precipitated overnight at room temperature was filtered off and then dried in vacuum to give 2.20 g (9%) of *meso*-complex. The mother liquor was evaporated to ca. 40 ml and heated to dissolve the precipitated solid. Yellow solid precipitated overnight at room temperature was filtered off and dried in vacuum. This procedure gave a ca. 5:1 mixture of *rac*- and *meso*-zirconocenes which was dissolved in 30 ml of hot toluene. Yellow crystals precipitated from this solution overnight at room temperature were filtered off and dried in vacuum to give 2.70 g (11%) of *rac*-complex. Further on, yellow crystals precipitated from the mother liquor for one week at room temperature were collected and dried in vacuum to give 5.00 g (20%) of a ca. 1:2 mixture of *rac*-and *meso*-zirconocenes. All remaining mother liquors were combined, evaporated to ca. 10 ml, and triturated with 40 ml of n-hexane. Yellow precipitate formed was filtered off (G3) and dried in vacuum to give 6.10 g (24%) of a ca. 3:2 mixture of *rac*-and *meso*-zirconocenes. Thus, the total yield of *rac*- and *meso*-zirconocenes isolated in this synthesis was 16.0 g (62%). *Rac*-zirconocene. Anal. calc. for $C_{44}H_{50}Cl_2SiZr$: C, 68.71; H, 6.55. Found: C, 69.23; H, 6.63. ^1H NMR ($CDCl_3$): δ 7.60 (br.s, 2H), 7.55 (s, 2H), 7.34 (d, J = 0.9 Hz, 2H), 7.29–7.22 (m, 6H), 6.51 (br.s, 2H), 2.26 (s, 6H), 2.13 (br.s, 6H), 1.33 (s, 6H), 1.29 (s, 18H). $^{13}C\{^1H\}$ NMR ($CDCl_3$): δ 147.99, 139.13, 136.44, 135.42, 134.56, 132.15, 130.04, 129.71, 127.90, 127.43, 126.81, 125.95, 121.66, 118.51, 82.28, 35.06, 30.66, 20.18, 18.54, 2.65.

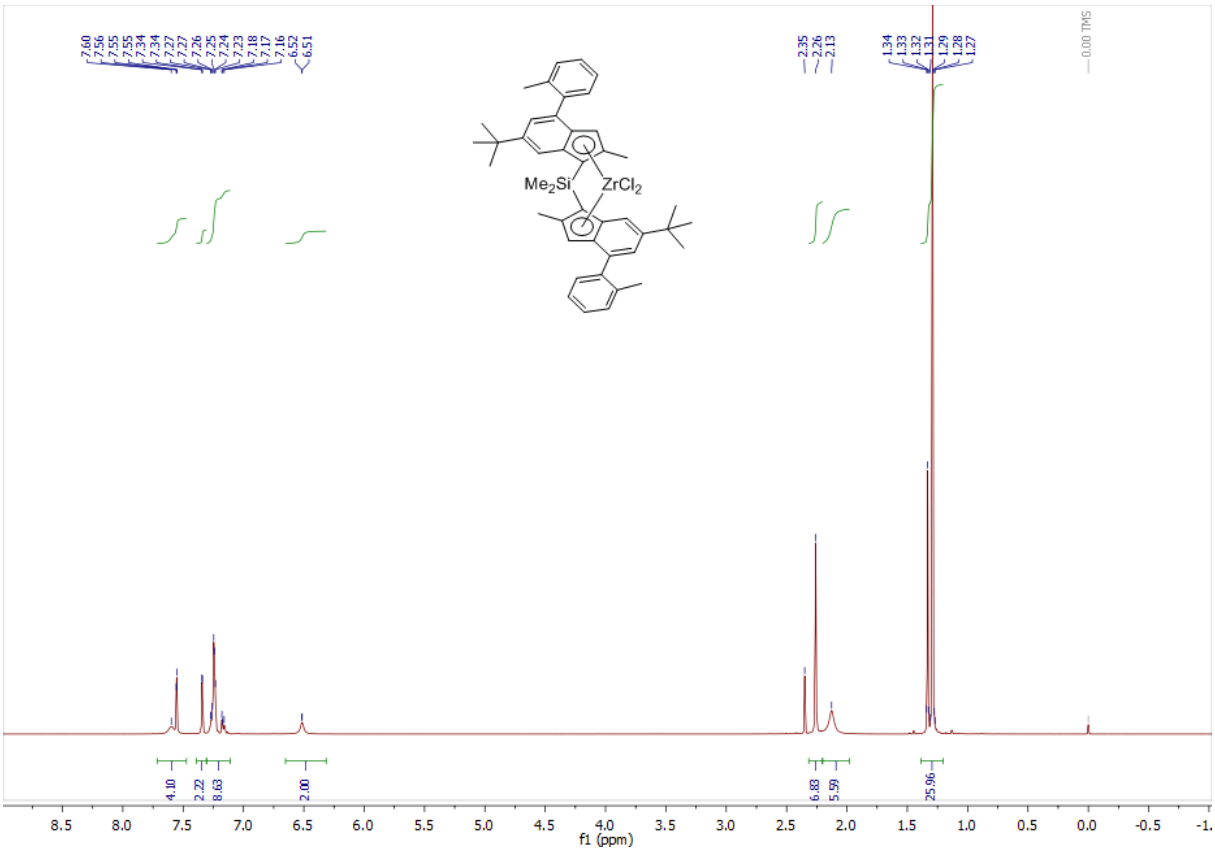

Figure S23. ^{1}H NMR spectrum of **M34** in $CDCl_3$. Traces of toluene are visible at 2.36 and 7.13–7.26 ppm.

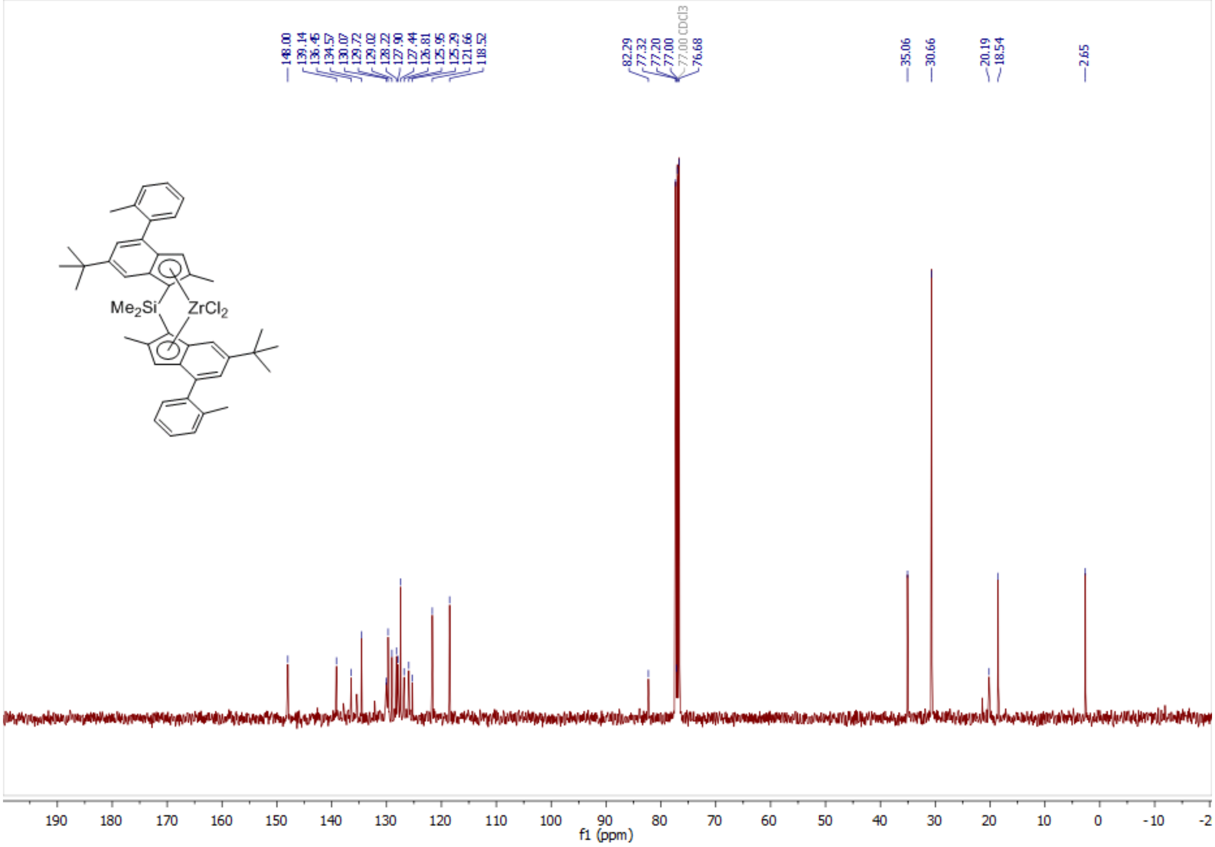

Figure S24. $^{13}C\{^{1}H\}$ NMR spectrum of **M34** in $CDCl_3$.

M35

6-chloro-4-(o-tolyl)-2-methyl-1H-indene

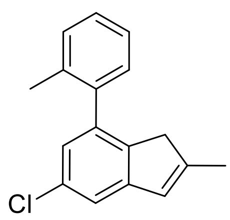

To a solution of zinc chloride (7.76 g, 56.9 mmol) in THF (200 ml) *o*-tolylmagnesium bromide (45.5 ml, 1.0M in THF, 45.5 mmol) and Pd[P(^{t}Bu$_3$)$_2$] (770 mg, 1.5 mmol) were added. To the resulting solution 2-methyl-4-bromo-6-chloro-1*H*-indene (9.24 g, 37.9 mmol) was added. The mixture was refluxed for 6 h, then cooled to room temperature. Water (200 ml) and dichloromethane (300 ml) were added to the reaction mixture followed by 1 M HCl (100 ml). The organic layer was separated, and the aqueous layer was extracted with 250 ml of dichloromethane. The combined organic phase was evaporated to dryness to give a slightly yellowish oil. The product was isolated by flash-chromatography on silica gel 60 (40-63 µm; eluent: hexanes). This procedure gave 9.0 g (93%) of the title product as a colorless solid. ^{1}H NMR (CDCl$_3$): *δ* 7.31 (m, 2H), 7.29–7.26 (m, 1H), 7.25 (d, *J* = 1.9 Hz, 1H), 7.22–7.19 (m, 1 H), 6.99 (d, *J* = 1.9 Hz, 1 H), 6.51 (m, 1H), 3.05 (br.s, 2 H), 2.18 (s, 3 H), 2.13 (br.s, 3 H).

bis[6-chloro-2-methyl-4-(2-methylphenyl)-1H-inden-1-yl]dimethylsilane

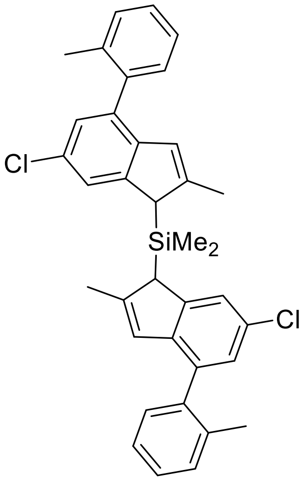

To a solution of 9.00 g (35.3 mmol) of 6-chloro-2-methyl-4-(2-methylphenyl)-1*H*-indene in 300 ml of ether 14.1 ml (35.3 mmol) of 2.5 M ^{n}BuLi in hexanes was added in one portion at -40 °C. This mixture was stirred overnight at room temperature, then cooled to -80°C, and 50 mg of N-methylimidazole was added. The resulting mixture was stirred for 5 min at -80 °C, then 2.28 g (17.7 mmol) of dichlorodimethylsilane was added in one portion. Further on, this mixture was stirred overnight at ambient temperature. The resulting mixture was filtered through a short pad of silica gel 60 (40-63 µm) which was additionally washed by 2 x 30 ml of dichloromethane. The combined filtrate was evaporated under reduced pressure, and the residue was purified by flash chromatography on silica gel 60 (40-63 µm, eluent: hexane:dichloromethane; 10:1, vol.). Yield 3.00 g (30%) of the product as yellowish foam. According to ^{1}H NMR, the product was a mixture of two diastereomers A and B in ~2:1 molar ratio.^{1}H NMR (CDCl$_3$): *δ* 7.37–7.16 (m, 10H in A and 10 H in B), 7.12 (br.s, 2H in A and 2H in B), 6.29 (br.s, 2H in A and 2H in B), 3.79 (br.s, 2H in A), 3.74 (br.s, 2H in B), 2.23 and 2.17 and 2.15 (3 br.s, sum 12H in A and B), –0.19 and –0.22 (2 br.s, sum 6H in A and B).

***rac*-dimethylsilanediylbis[η^5-2-methyl-6-chloro-4-(2-methylphenyl)-inden-1-yl]zirconium dichloride (M35)**

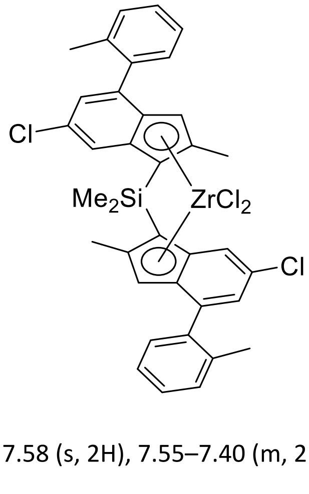

To a cooled to -40 °C solution of 3.00 g (5.30 mmol) of bis(2-methyl-6-chloro-4-(2-methylphenyl)-1*H*-inden-1-yl)dimethylsilane in 250 ml of ether 4.24 ml (10.6 mmol) of 2.5 M ^{n}BuLi in hexanes was added in one portion. This mixture was stirred overnight at room temperature, then cooled to -80 °C, and 2.00 g (5.30 mmol) of ZrCl$_4$(THF)$_2$ was added. The reaction mixture was stirred for 24 h, then evaporated to dryness. The residue was heated with 250 ml of toluene, and the formed hot suspension was filtered through glass frit (G4). The filtrate was evaporated to dryness. The resulting solid was recrystallized from toluene yielding 460 mg (12%) of *rac*-complex as a yellow powder. Anal. calc. for $C_{36}H_{32}Cl_4SiZr$: C, 59.58; H, 4.44. Found: C, 59.78; H, 4.57. ^{1}H NMR (CDCl$_3$): *δ* 7.58 (s, 2H), 7.55–7.40 (m, 2H), 7.33–7.13 (m, 8H), 6.71–6.41 (m, 2H), 2.26 (s, 6H), 2.19–1.96 (m, 6H), 1.31 (s,

6H). $^{13}C\{^{1}H\}$ NMR (C_6D_6): δ 139.7, 137.9, 135.9, 135.4, 132.3, 132.2, 130.4, 129.8, 129.1, 128.5, 127.1, 126.5, 122.8, 122.6, 83.8, 19.9, 18.3, 1.7.

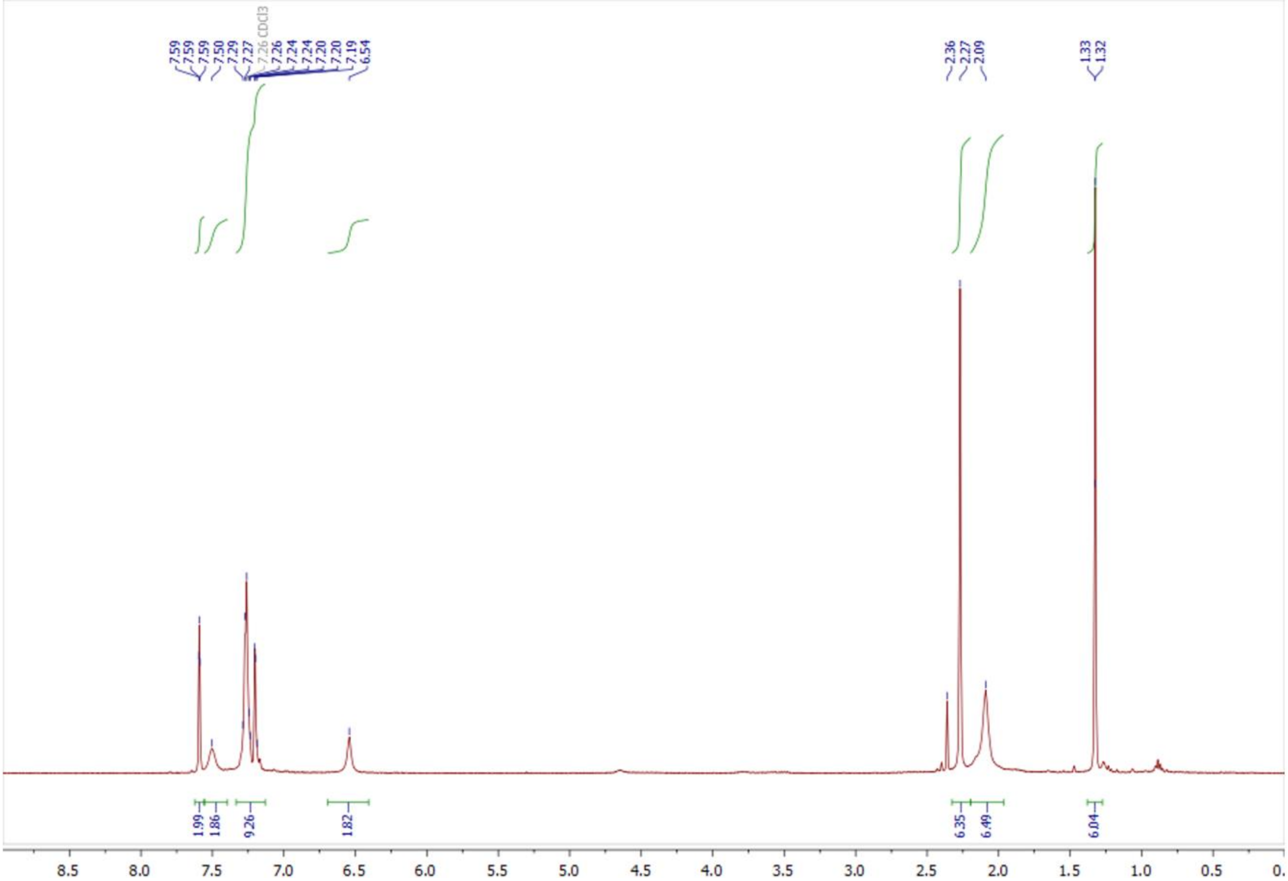

Figure S25. ^{1}H NMR spectrum of **M35** in $CDCl_3$. Traces of toluene are visible at 2.36 and 7.13–7.26 ppm.

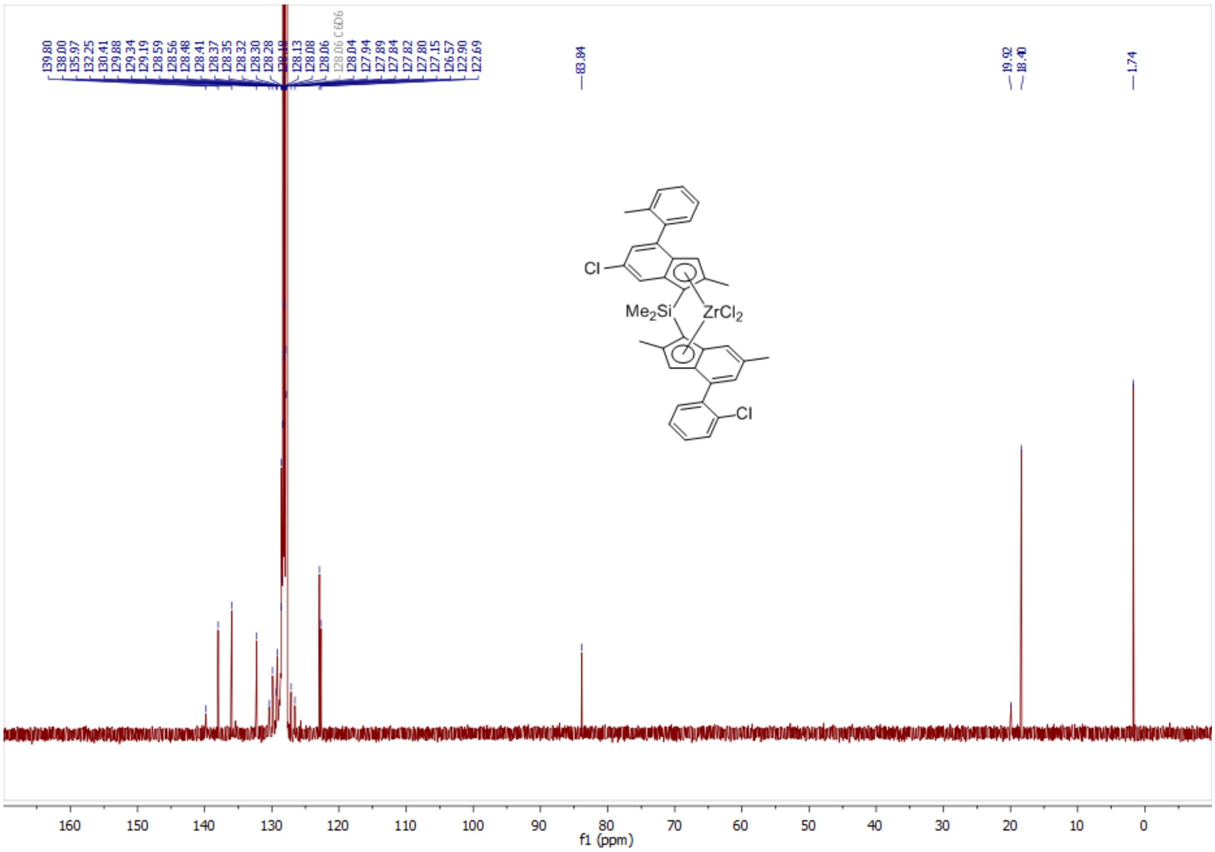

Figure S26. $^{13}C\{^1H\}$ NMR spectrum of **M35** in $CDCl_3$.

M36

bis(2-isopropyl-4-phenyl-1H-inden-1-yl)dimethylsilane

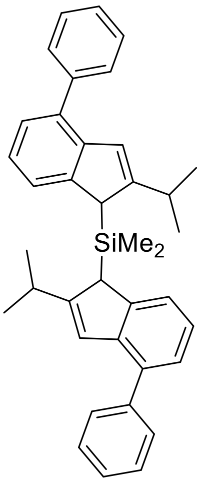

To a solution of 7.40 g (31.5 mmol) of 2-isopropyl-4-phenyl-1*H*-indene in 130 ml of ether 12.6 ml (31.5 mmol) of 2.5 M ^{n}BuLi in hexanes was added in one portion at -40 °C. This mixture was stirred overnight at room temperature, then the resulting solution was cooled to -80°C, and 50 mg of N-methylimidazole was added. The resulting mixture was stirred for 5 min at -80 °C, then 2.03 g (15.6 mmol) of dichlorodimethylsilane was added in one portion. Further on, this mixture was stirred overnight at ambient temperature. The resulting mixture was filtered through a short pad of silica gel 60 (40-63 μm) which was additionally washed by 2 x 30 ml of dichloromethane. The combined filtrate was evaporated under reduced pressure, and the residue was purified by flash chromatography on silica gel 60 (40-63 μm, eluent: hexane:dichloromethane; 10:1, vol.). Yield 7.70 g (94%) of the product as white foam. According to ^{1}H NMR, the product was a mixture of *rac* and *meso* isomers in 1.7:1 molar ratio. Anal. calc. for: $C_{38}H_{40}Si$: C, 86.97; H, 7.68. Found: C, 86.90; H, 7.74. ^{1}H NMR ($CDCl_3$): δ 7.65–7.61 (m, 2H in *rac* and 2H in *meso*), 7.59–7.41 (4H in *rac* and 4H in *meso*), 7.34 (t, *J* = 7.6 Hz, 2H in *rac* and 2H in *meso*), 7.28 (d, *J* = 7.6 Hz, 2H in *rac*), 7.24 (t, *J* = 7.5 Hz, 2H in *meso*), 6.87 (br.s, 2H in *meso*), 6.82 (br.s, 2H in *rac*), 3.98 (br.s, 2H in *rac*), 3.94 (br.s, 2H in *meso*), 2.77 (m, 2H in *meso*), 2.66 (m, 2H in *rac*), 1.34 (d, *J* = 6.7 Hz, 3H in *meso*), 1.24 (d, *J* = 6.6 Hz, 3H in *rac*), 1.19 (d, *J* = 6.9 Hz, 3H in *meso*), 1.11 (d, *J* = 6.9 Hz, 3H in *rac*), –0.03 (s, 6H in *rac*), –0.05 (s, 3H in *meso*), –0.13 (s, 3H in *meso*).

rac-dimethylsilanediylbis[η⁵-2-isopropyl-4-phenyl-inden-1-yl]zirconium dichloride (M36)

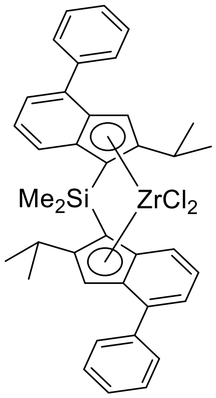

To a solution of 7.70 g (14.7 mmol) of bis(2-isopropyl-4-phenyl-1H-inden-1-yl)dimethylsilane in 250 ml of Et_2O, cooled to -40°C, 11.7 ml (29.3 mmol) of 2.5 M *n*BuLi in hexanes was added in one portion. This mixture was stirred overnight at room temperature. The resulting light-yellow solution was cooled to -80 °C and 5.54 g (14.7 mmol) of $ZrCl_4(THF)_2$ was added. The reaction mixture was stirred for 24 h resulting in orange solution with light-orange precipitate. The resulting light-orange slightly turbid solution was evaporated to dryness, the residue was heated with 250 ml of toluene and this mixture was filtered while hot through glass frit (G4). The filtrate was evaporated to dryness The resulting solid was recrystallized from toluene yielding 185 mg (1.8%) of a rac-dimethylsilanediylbis[η⁵-2-isopropyl-4-phenyl-inden-1-yl]zirconium dichloride as orange crystals. Anal. calc. for $C_{38}H_{38}Cl_2SiZr$: C, 66.64; H, 5.59. Found: C, 66.72; H, 5.53. ^{1}H NMR (C_6D_6): δ 7.91 (m, 4H), 7.41 (d, *J* = 8.7 Hz, 2H), 7.31 (d, *J* = 7.0 Hz, 2H), 7.22 (m, 4H), 7.08 (m, 2H), 6.87 (dd, *J* = 8.7 Hz, *J* = 7.0 Hz, 2H), 3.08 (m, 2H), 1.11 (d, *J* = 6.6 Hz, 6H), 0.95 (d, *J* = 6.8 Hz, 6H), 0.93 (s, 6H). ^{13}C{^{1}H} NMR (C_6D_6): δ 149.6, 140.3, 140.2, 132.3, 129.2, 129.1, 128.12, 128.06, 126.8, 126.4, 123.9, 115.6, 82.3, 30.6, 29.2, 19.8, 3.6.

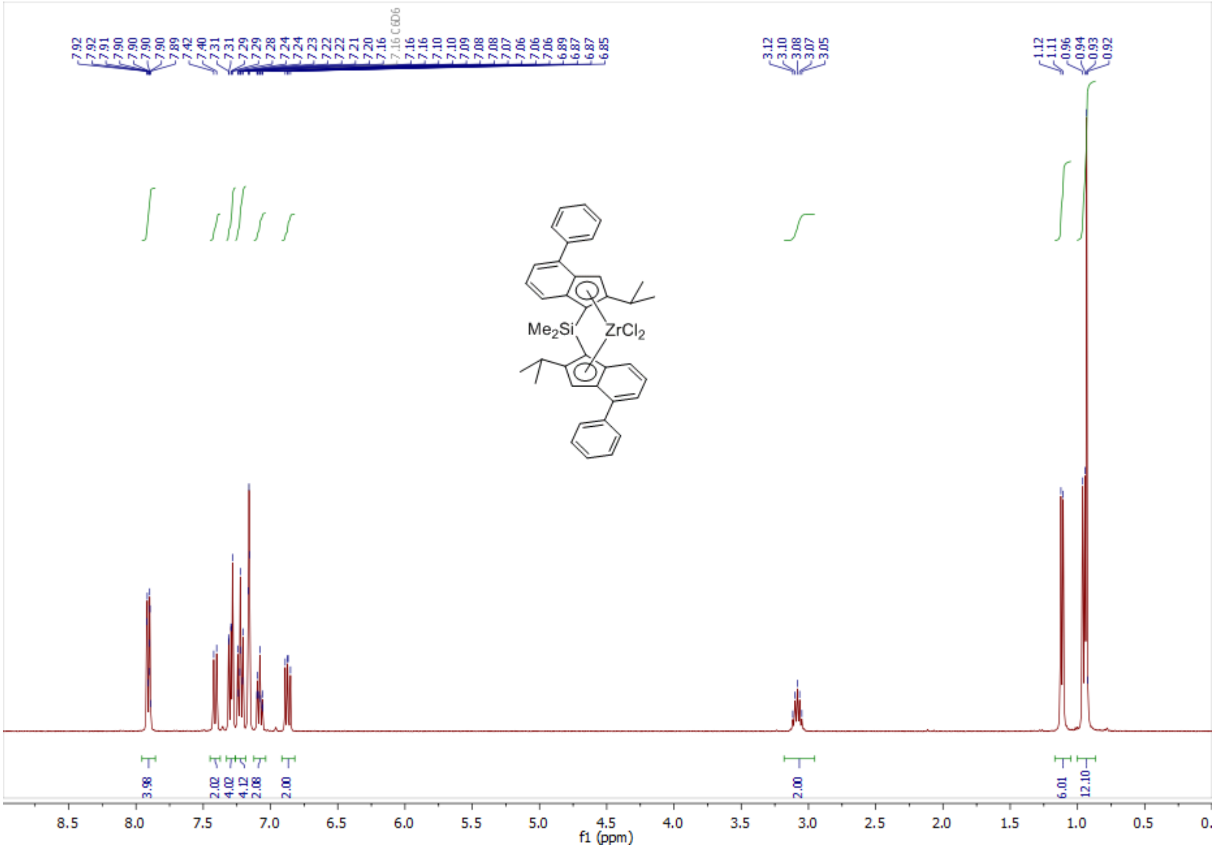

Figure S27. ^{1}H NMR spectrum of **M36** in C_6D_6.

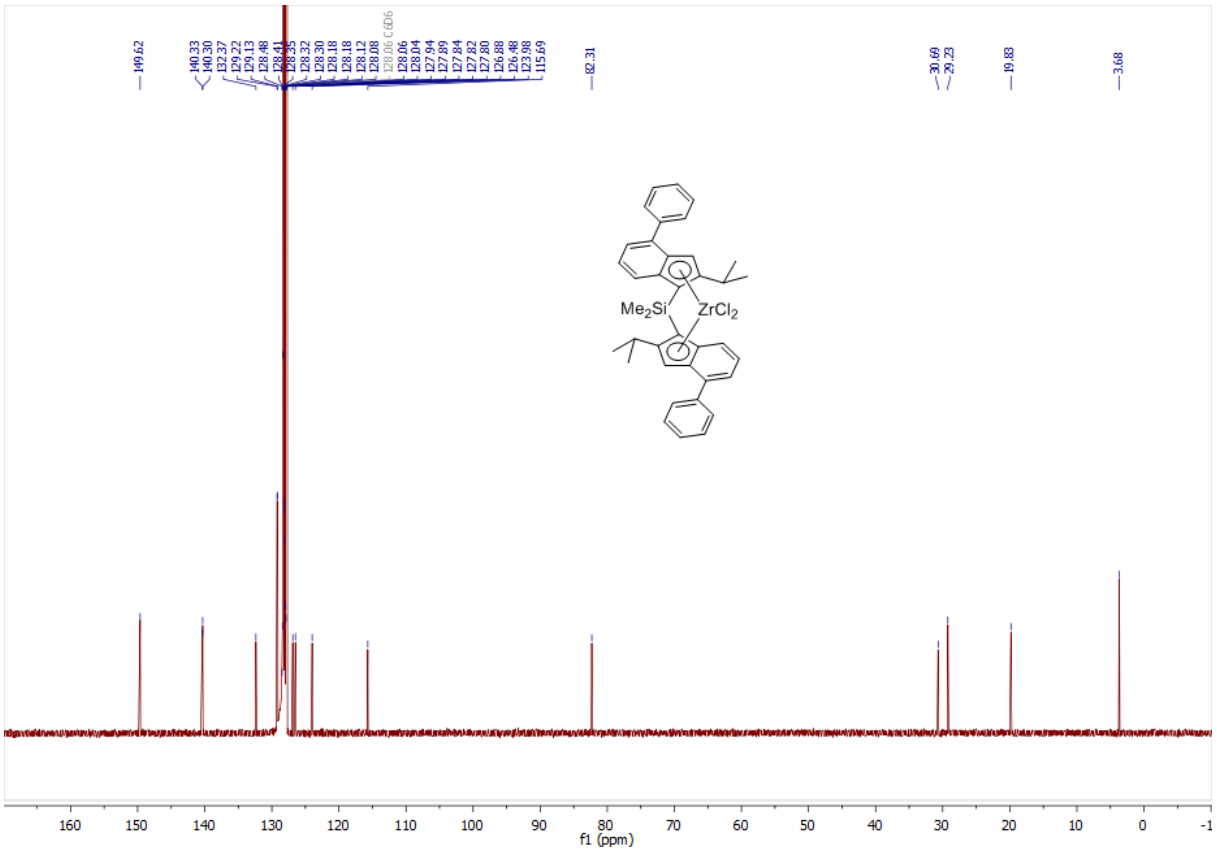

Figure S28.$^{13}C\{^1H\}$ NMR spectrum of **M36** in C_6D_6.

M39

bis(2,6-dimethyl-4-(3,5-di-tert-butylphenyl)-1H-inden-1-yl)diethylsilane

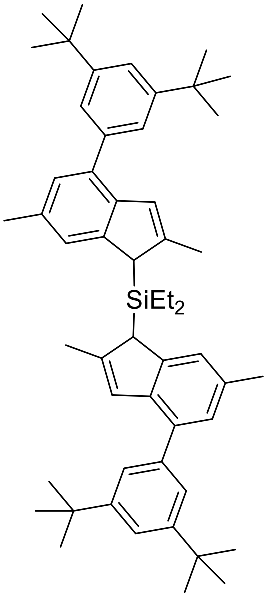

To a solution of 8.70 g (26.2 mmol) of 2,6-dimethyl-4-(3,5-di-*tert*-butylphenyl)-1*H*-indene in 200 ml of ether 10.5 ml (26.2 mmol) of 2.5 M ^{n}BuLi in hexanes was added in one portion at -40 °C. This mixture was stirred overnight at room temperature, then the resulting light-orange solution was cooled to -40°C, and 1.17 g (13.1 mmol) of CuCN was added. The resulting mixture was stirred for 30 min at -25 °C, then 2.06 g (13.1 mmol) of dichlorodiethylsilane was added in one portion. Further on, this mixture was stirred overnight at ambient temperature. The resulting mixture was filtered through a short pad of silica gel 60 (40-63 µm) which was additionally washed by 2 x 30 ml of dichloromethane. The combined filtrate was evaporated under reduced pressure, and the residue was purified by flash chromatography on silica gel 60 (40-63 µm, eluent: hexane:dichloromethane, 10:1 vol). Yield 4.64 g (47%) of the product as a white foam. According to ^{1}H NMR, the product was a mixture of *rac*- and *meso*-isomers in ca. 1:1 molar ratio. Anal. calc. for $C_{54}H_{72}Si$: C, 86.57; H, 9.69. Found: C, 86.51; H, 9.80. ^{1}H NMR ($CDCl_3$): δ 7.45–7.35 (m, 6H), 7.21 (s, 1H), 7.12 (s, 1H), 7.15 (s, 1H), 7.02 (s, 1H), 6.76 (s, 1H), 6.80 (s, 1H), 3.65 (s, 1H), 3.60 (s, 1H), 2.44 (s, 3H), 2.46 (s, 3H), 2.26 (s, 3H), 2.15 (s, 3H), 1.41 and 1.40 (s and s, sum 36H), 0.89–0.61 (m, 10H).

rac-diethylsilanediylbis[η⁵-2,6-dimethyl-4-(3,5-di-tert-butylphenyl)-inden-1-yl]zirconium dichloride (M39)

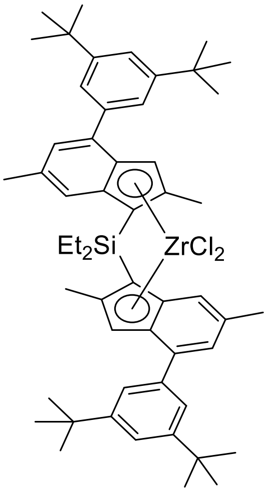

To a cooled to –40 °C solution of 4.64 g (6.19 mmol) of bis(4-(3,5-di-*tert*-butylphenyl)-2,6-dimethyl-1*H*-inden-1-yl)diethylsilane in 150 ml of ether 4.95 ml (12.4 mmol) of 2.5 M ^{n}BuLi in hexanes was added in one portion. This mixture was stirred overnight at room temperature. The resulting light-yellow solution was cooled to -80 °C, and 2.33 g (6.19 mmol) of $ZrCl_4(THF)_2$ was added. The reaction mixture was stirred for 24 h and then evaporated to dryness. The residue was heated with 100 ml of toluene, and the formed hot suspension was filtered through glass frit (G4). The filtrate was evaporated to dryness giving 3.23 g of a ca. 1:1 mixture of *rac*- and *meso*-complexes. This crude product was recrystallized twice from 100 ml of methylcyclohexane/hexane mixture (1:1 vol.) yielding 310 mg (5.5%) of *rac*-complex as a yellow powder. Anal. calc. for $C_{54}H_{70}Cl_2SiZr$: C, 71.32; H, 7.76. Found: C, 71.15; H, 7.92. ^{1}H NMR ($CDCl_3$): *δ* 7.50 (d, *J* = 1.7 Hz, 4H), 7.41–7.34 (m, 4H), 7.23 (br.s, 2H), 6.91 (s, 2H), 2.38 (s, 6H), 2.23 (s, 6H), 1.98–1.77 (m, 4H), 1.46 (t, *J* = 7.8 Hz, 6H), 1.31 (s, 36H).^{13}C{^{1}H} NMR (CD_2Cl_2): *δ* 152.8, 140.5, 140.1, 137.6, 137.0, 131.8, 130.5, 129.7, 124.8, 124.5, 124.4, 123.0, 36.5, 32.8, 23.4, 7.9, 7.1.

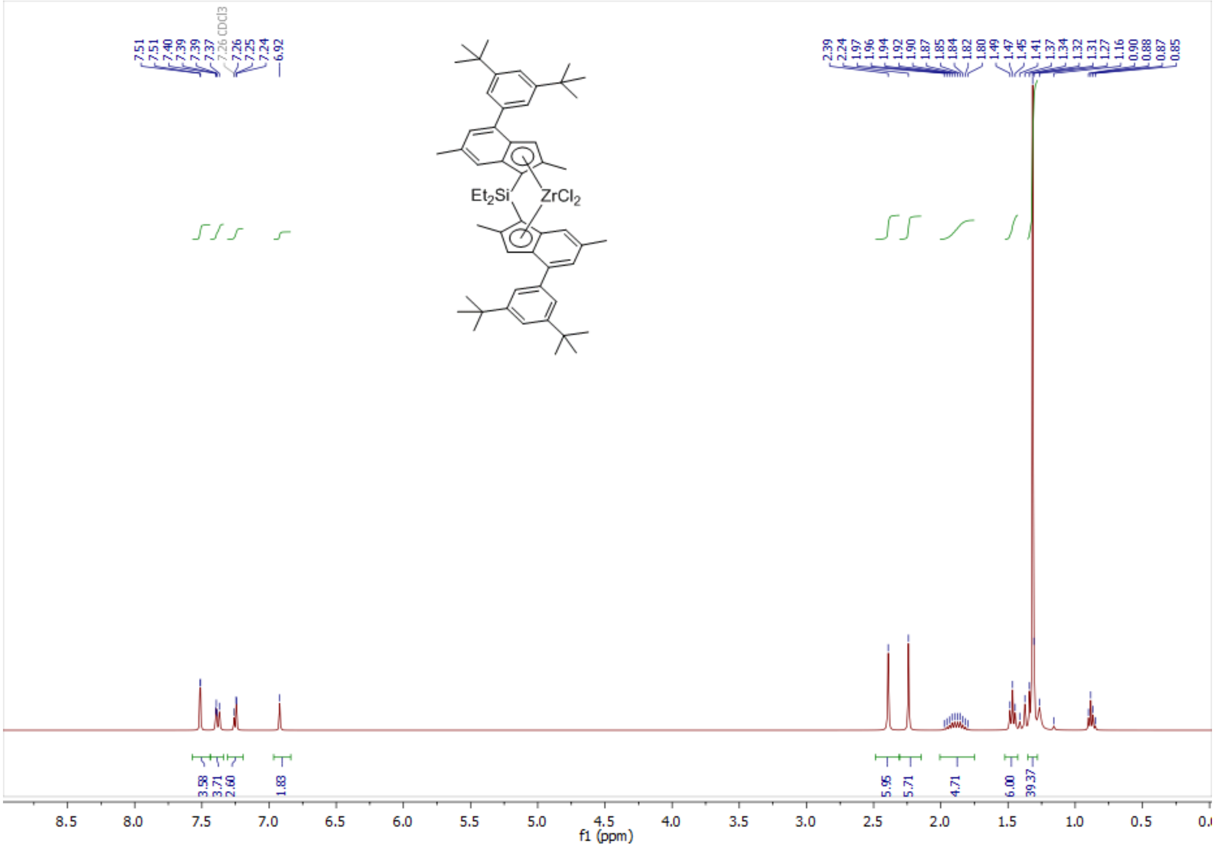

Figure S29. ^{1}H NMR spectrum of **M39** in $CDCl_3$.

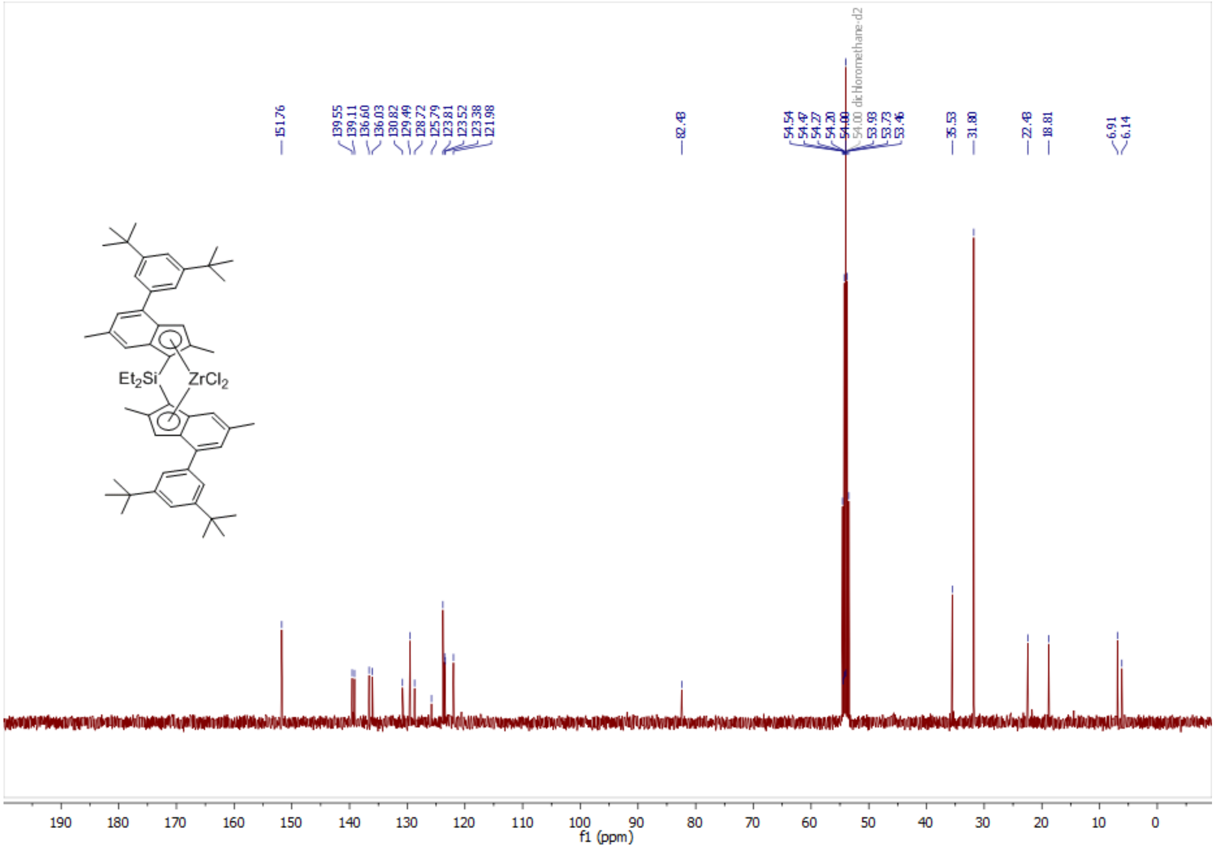

Figure S30. ${}^{13}C\{{}^{1}H\}$ NMR spectrum of **M39** in $CDCl_3$.

M40

2-bromo-4-methylbenzaldehyde

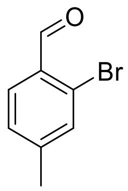

To a solution of 2-bromo-1-iodo-4-methylbenzene (50.0 g, 168 mmol) in THF (500 ml) cooled to –60 °C *i*PrMgCl·LiCl (195 ml of 0.95 M in THF, 185 mmol) was added for 1 h. Then *N*,*N*-dimethylformamide (18.5 g, 253 mmol) was added at the same temperature. After 30 min of stirring at room temperature the resulting mixture was poured into water (1000 ml) and extracted with diethyl ether (3 × 100 ml). The combined organic extracts were dried over Na_2SO_4 and the solvents were removed in vacuum. Distillation of the residue in vacuum afforded 26.4 g (79%) of the product as a colorless oil, b.p. 110–130 °C/1 mbar. ${}^{1}H$ NMR (600 MHz, $CDCl_3$): δ 10.18 (s, 1H), 7.69 (d, *J*=7.9 Hz, 1H), 7.35 (s, 1H), 7.12 (d, *J*=7.9 Hz, 1H), 2.30 (s, 3H). ${}^{13}C\{{}^{1}H\}$ NMR (151 MHz, $CDCl_3$): δ 191.0, 146.6, 133.9, 130.9, 129.4, 128.6, 126.8, 21.2.

2-bromo-1-(chloromethyl)-4-methylbenzene

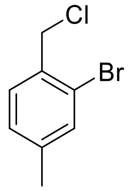

To a solution of 2-bromo-4-methylbenzaldehyde (25.5 g, 128 mmol) in THF (200 ml) cooled to 5 °C $NaBH_4$ (7.28 g, 192 mmol) was added. Then, methanol (100 ml) was added dropwise to this mixture under vigorous stirring at 5 °C for ca. 3 h. The solvents were then removed in vacuum, and the residue was dissolved in a mixture of dichloromethane (200 ml) and water (100 ml). The mixture was acidified with 4M HCl to pH 5. The organic phase was separated, and the aqueous layer was additionally extracted with dichloromethane (2 × 100 ml). The combined organic phase was dried over Na_2SO_4 and evaporated to dryness in vacuum to give a colorless liquid which was diluted with dichlomethane (200 ml). Then thionyl chloride (18.3 g, 154

mmol) was added dropwise for 30 min. After 30 min of stirring at room temperature, the resulting mixture was poured into water (500 ml) of and extracted with dichloromethane (3 × 100 ml). The extract was washed with 10% aqueous K_2CO_3, dried over Na_2SO_4 and then passed through a short pad of silica gel 60 (40–63 µm). The silica gel layer was additionally washed with dichloromethane (100 ml). The combined organic filtrate was evaporated in vacuum. Distillation of the residue in vacuum afforded 24.0 g (89%) of the product as a colorless oil, b.p. 68–72 °C/1 mbar. ^{1}H NMR (400 MHz, $CDCl_3$): δ7.42 (d, J=0.9 Hz, 1H), 7.34 (d, J=7.8 Hz, 1 H), 7.11 (dd, J=7.8 Hz, J=0.9 Hz, 1H), 4.68 (s, 2 H), 2.33 (s, 3 H). ^{13}C{^{1}H} NMR (101 MHz, $CDCl_3$): δ 140.4, 133.6, 133.5, 130.6, 128.6, 123.9, 46.0, 20.7.

2-(2-bromo-4-methylbenzyl)butanoyl chloride

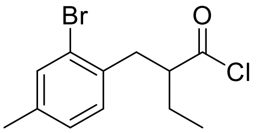

To suspension of sodium hydride (4.75 g of 60% dispersion in mineral oil, 119 mmol), in THF (500 ml) cooled to 0 °C, diethyl ethylmalonate (24.4 g, 129 mmol) was added. After 30 min of stirring at r.t., 2-bromo-1-(chloromethyl)-4-methylbenzene (23.7 g, 108 mmol) was added for 1 h. The resulting mixture was stirred overnight at 60 °C and the solvents were removed in vacuum. To the residue 500 ml of water was added and the mixture was extracted with dichloromethane (3 × 150 ml). The combined organic extracts were stripped of solvent in vacuum and the residue was dissolved in ethanol (200 ml, 95%). Then aqueous potassium hydroxide (18.2 g, 324 mmol in 60 ml of water) was added carefully. The resulting mixture was refluxed for 6 h, then diluted with water (300 ml), and most of the ethanol was distilled off. Water (200 ml) was added followed by acidification with 4M HCl to pH 1. The crude product was extracted with dichloromethane (3 × 150 ml), the combined organic extract was dried over Na_2SO_4 and the solvents were removed in vacuum. The residue was heated to 200 °C and kept at this temperature until evolution of gas ceased. To the residue thionyl chloride (38.6 g, 324 mmol) was added. The mixture was stirred overnight at r.t., then the excess of thionyl chloride was distilled off. Distillation of the reside in vacuum afforded 27.8 g (89%) of the product as a greenish oil, b.p. 105–118 °C/1 mbar. ^{1}H NMR (400 MHz, $CDCl_3$): δ7.38 (d, J=0.9 Hz, 1H), 7.11 (d, J=7.8 Hz, 1H), 7.04 (dd, J=7.8 Hz, J=0.9 Hz, 1H), 3.23–3.15 (m, 1H), 3.15–3.08 (m, 1 H), 2.94 (dd, J=13.7 Hz, J=6.1 Hz, 1H), 2.30 (s, 3H), 1.90–1.75 (m, 1H), 1.80–1.65 (m, 1H), 1.02 (t, J=7.4 Hz, 3H). ^{13}C{^{1}H} NMR (101 MHz, $CDCl_3$) δ176.1, 138.4, 133.5, 133.1, 130.7, 128.0, 123.9, 58.1, 36.9, 24.6, 20.2, 10.7.

4-bromo-2-ethyl-6-methylindan-1-one

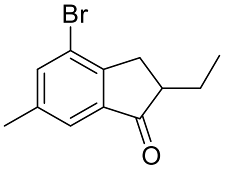

To a suspension of $AlCl_3$ (16.0 g, 120 mmol) in dichloromethane (100 ml) cooled to 0 °C a solution of 2-(2-bromo-4-methylbenzyl)butanoyl chloride (27.8 g, 96.0 mmol) in dichloromethane (50 ml) was added dropwise for 2 h. After stirring overnight the obtained mixture was poured into 200 g of crushed ice and acidified with 5 ml of concentrated HCl. The organic phase was separated and the water phase was extracted with dichloromethane (3 x 50 ml). The combined organic phases were passed through a short layer of silica gel. Solvents were removed from the filtrate in vacuum. Distillation of the residue in vacuum afforded 21.3 g (88%) of the product as a yellowish oil, b.p. 105–115 °C/1 mbar. ^{1}H NMR (400 MHz, $CDCl_3$): δ 7.54 (s, 1H), 7.44 (s, 1H), 3.17 (dd, J=17.3 Hz, J=7.7 Hz, 1H), 2.68–2.56 (m, 2H), 2.35 (s, 3H), 1,99–1.88 (m, 1H), 1.57–1.45 (m, 1H), 0.98 (t, J=7.4 Hz, 3H). ^{13}C{^{1}H} NMR (101 MHz, $CDCl_3$): δ 208.0, 150.6, 139.5, 138.8, 138.1, 122.8, 121.6, 49.0, 32.9, 24.3, 20.7, 11.5.

4-(3,5-di-tert-butylphenyl)-2-ethyl-6-methylindan-1-one

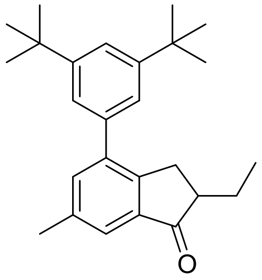

To a solution of 4-bromo-2-ethyl-6-methylindan-1-one (12.5 g, 49.4 mmol) in 1,4-dioxane (200 ml) 2-(3,5-di-*tert*-butylphenyl)-4,4,5,5-tetramethyl-1,3,2-dioxaborolane (23.4 g, 74.1 mmol), cesium carbonate (48.3 g, 148 mmol), and water (100 ml) were subsequently added. The obtained mixture was purged with argon for 10 min followed by addition of $Pd(PPh_3)_4$ (2.85 g, 2.47 mmol). The mixture was stirred for 12 h at 90 °C, then cooled to room temperature, and diluted with 50 ml of water. The mixture was extracted with dichloromethane (3 × 100 ml). The combined organic extract was dried over Na_2SO_4 and the solvent was removed in vacuum. The residue was purified by flash chromatography on silica gel 60 (40–63 µm, eluent: hexane-ethyl acetate = 20:1, vol). Yield 14.4 g (80%) of the product as a white powder. ^{1}H NMR (400 MHz, $CDCl_3$): δ 7.60 (br.s, 1H), 7.51 (t, J=1.7 Hz, 1H), 7.48 (d, J=1.0 Hz, 1H), 7.34 (d, J=1.8 Hz, 2H), 3.37 (dd, J=17.2 Hz, J=7.8 Hz, 1 H), 2.84 (dd, J=17.2 Hz, J=3.7 Hz, 1 H), 2.66 (m, J=8.4 Hz, J=4.2 Hz, 1 H), 2.50 (s, 3 H), 2.07–1.95 (m, 1H), 1.63–1.50 (m, 1H), 1.43 (s, 18H), 1.02 (t, J=7.4 Hz, 3 H). ^{13}C{^{1}H} NMR (101 MHz, $CDCl_3$): δ 209.1, 150.9, 148.7, 141.0, 138.5, 137.7, 137.6, 136.0, 122.8, 122.5, 121.4, 49.2, 34.9, 32.0, 31.5, 24.4, 21.1, 11.6.

7-(3,5-di-tert-butylphenyl)-2-ethyl-5-methyl-1H-indene

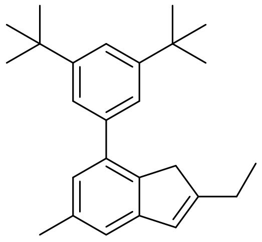

To a solution 4-(3,5-di-tert-butylphenyl)-2-ethyl-6-methylindan-1-one (14.4 g, 39.6 mmol) in THF (100 ml) cooled to 5 °C, $NaBH_4$ (2.25 g, 59.4 mmol) was added. Further on, methanol (50 ml) was added dropwise to this mixture under vigorous stirring at 5 °C for ca. 3 h. Then, the solvents were removed in vacuum, and to the residue dichloromethane (200 ml) and water (100 ml) were added. The mixture was acidified to pH 5 with 4M HCl. The organic layer was separated, the aqueous layer was additionally extracted with dichloromethane (2 x 50 ml). The combined organic phase was dried over Na_2SO_4 and evaporated to dryness in vacuum. The residue was dissolved in warm (40–50 °C) toluene (100 ml), and TsOH (0.05 g) was added. The mixture was refluxed with Dean-Stark head for 10 min, and then cooled to room temperature using external ice-water bath. The resulting solution was passed through a short layer of silica gel 60 (40–63 um), the silica gel layer was additionally washed with 100 ml of dichloromethane. The combined organic filtrate was evaporated to dryness and the residue was additionally purified by flash chromatography on silica gel 60 (40–63 µm; eluent: hexane). Yield 13.0 g (95%) of the product as a white solid. ^{1}H NMR (400 MHz, $CDCl_3$): δ 7.56 (t, J=1.8 Hz, 1H), 7.54 (d, J=1.8 Hz, 2H), 7.24 (s, 1H), 7.15 (s, 1H), 6.64 (br.s, 1H), 3.51 (s, 2H), 2.61 (q, J=7.4 Hz, 2H), 2.58 (s, 3H), 1.52 (s, 18H), 1.33 (t, J=7.5 Hz, 3H). ^{13}C{^{1}H} NMR (101 MHz, $CDCl_3$): δ152.9, 150.5, 146.5, 140.6, 138.3, 137.7, 136.5, 125.18, 125.16, 122.8, 120.8, 119.7, 40.7, 34.9, 31.6, 24.3, 21.5, 13.3.

bis(4-(3,5-di-tert-butylphenyl)-2-ethyl-6-methyl-1H-inden-1-yl)diethylsilane

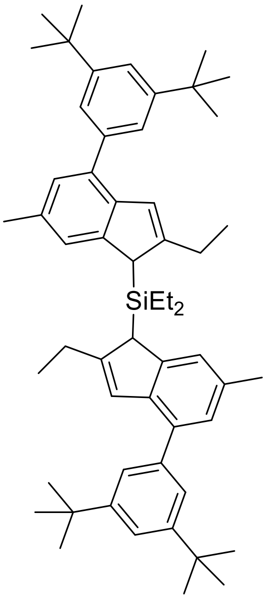

To a solution of 7-(3,5-di-*tert*-butylphenyl)-2-ethyl-5-methyl-1*H*-indene (12.9 g, 37.2 mmol) in ether (150 ml) *n*BuLi (14.9 ml of 2.5 M in hexanes, 37.2 mmol) was added in one portion at –40 °C. This mixture was stirred overnight at r.t. and THF (20 ml) was added. The mixture was cooled to –40°C, and CuCN (1.66 g, 18.6 mmol) was added. The mixture was stirred for 30 min at –25 °C, then dichlorodiethylsilane (2.92 g, 18.6 mmol) was added in one portion, and the mixture was stirred overnight at ambient temperature. The mixture was filtered through a short pad of silica gel 60 (40–63 µm) which was additionally washed with dichloromethane (2 × 30 ml). The combined filtrate was evaporated in vacuum, and the residue was purified by flash chromatography on silica gel 60 (40–63 µm, eluent: hexane-dichloromethane, 10:1, vol).Yield 5.80 g (40%) of the product as a white foam-like solid. HRMS: $[M]^+$ calc for $C_{56}H_{76}Si$: 776.5716; found: 776.5731.^{1}H NMR (400 MHz, $CDCl_3$): *δ* 7.53–7.44 (m, 12H), 7.25 (s, 2H), 7.21 (s, 2H), 7.19 (s, 2H), 7.01 (s, 2H), 6.90 (s, 2H), 6.86 (s, 2H), 3.69 (s, 2H), 3.62 (s, 6H), 2.78–2.66 (m, 4H), 2.53 (s, 6H), 2.50 (s, 6H), 2.48–2.35 (m, 4H), 1.472 (s, 36H), 1.466 (s, 36H), 0.92–0.70 (m, 10H). $^{13}C\{^1H\}$ NMR (101 MHz, $CDCl_3$): *δ*153.7, 153.5, 150.87, 150.83, 150.47, 150.45, 146.3, 140.51, 140.48, 140.32, 140.27, 134.8, 132.4, 126.4, 126.3, 123.6, 123.5, 123.31, 123.28, 120.5, 44.2, 44.1, 34.4, 31.6, 25.0, 24.8, 21.7, 21.6, 13.57, 13.55, 7.42, 7.35, 4.1, 3.8.

***rac*-[1,1'-(diethylsilanediyl)bis(η^5-4-(3,5-di-tert-butylphenyl)-2-ethyl-6-methyl-1H-inden-1-yl)]zirconium dichloride (M40)**

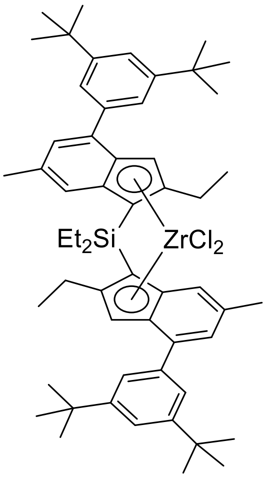

To a solution of bis(4-(3,5-di-*tert*-butylphenyl)-2-ethyl-6-methyl-1*H*-inden-1-yl)diethylsilane (5.70 g, 7.33 mmol) in ether (250 ml) cooled to –40 °C *n*BuLi (5.87 ml of 2.5 M in hexanes, 14.7 mmol) was added in one portion. The mixture was stirred overnight at room temperature, then cooled to –80 °C, and $ZrCl_4(THF)_2$ (2.77 g, 7.33 mmol) was added. The mixture was stirred for 24 h and then the solvents were removed in vacuum. The residue was taken up in toluene (100 ml), and the formed hot suspension was filtered through a glass frit (G4). The filtrate was evaporated in vacuum to dryness. Recrystallization of the solid residue from 70 ml of methylcyclohexane/hexane mixture (2:1 vol.) afforded 0.50 g (7%) of the *rac*-complex as a yellow powder. Anal. calc. for $C_{56}H_{74}Cl_2SiZr$: C, 71.75; H, 7.96. Found: C, 71.84; H, 8.02. ^{1}H NMR (400 MHz, $CDCl_3$): *δ* 7.51 (d, *J*=1.8 Hz, 4H), 7.38 (t, *J*=1.8 Hz, 2H), 7.33 (br.s, 2H), 7.23 (br.s, 2H), 6.96 (s, 2H), 2.78–2.67 (m, 2H), 2.54–2.42 (m, 2H), 2.38 (s, 6H), 1.96–1.86 (m, 2H), 1.96–1.86 (m, 2H), 1.85–1.75 (m, 2H), 1.47 (t, *J*=7.8 Hz, 6H), 1.31 (s, 36H), 1.05 (t, *J*=7.5 Hz, 6H). $^{13}C\{^1H\}$ NMR (101MHz, $CDCl_3$): *δ* 150.9, 142.4, 139.5, 138.6, 135.9, 130.7, 128.73, 128.68, 123.4, 122.6, 121.3, 120.7, 80.4, 35.1, 31.5, 25.8, 22.2, 16.7, 6.7, 6.0.

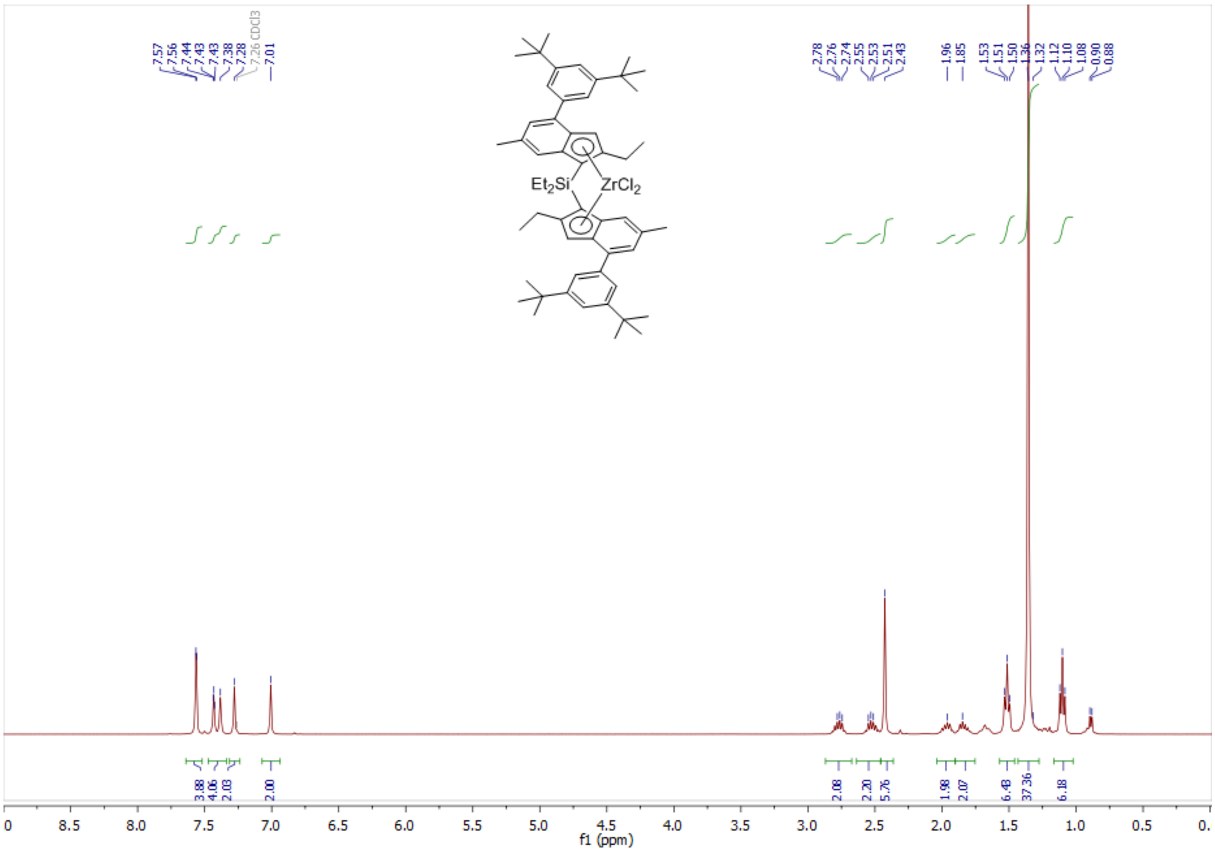

Figure S31. ^{1}H NMR spectrum of **M40** in $CDCl_3$. Traces of hexane are visible at 0.88–0.90 ppm.

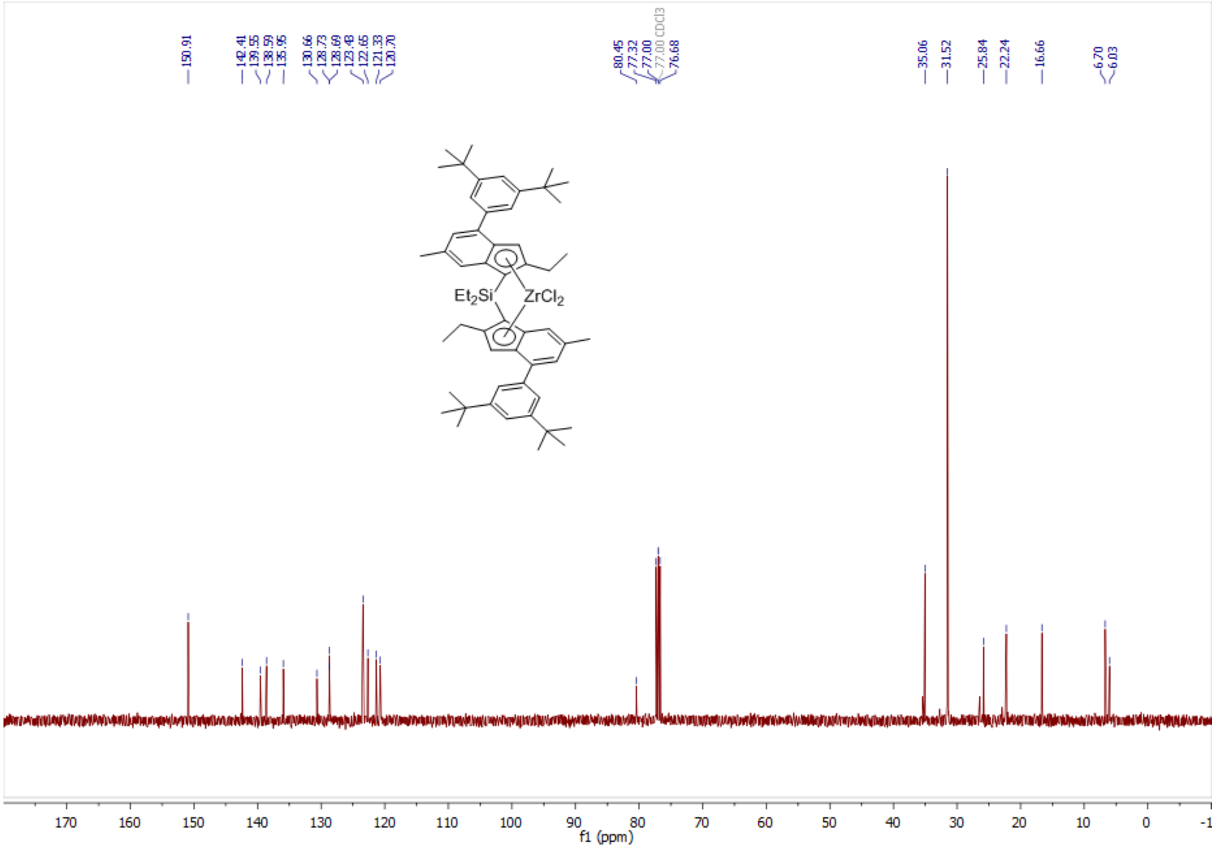

Figure S32. $^{13}C\{^{1}H\}$ NMR spectrum of **M40** in $CDCl_3$.

M41

7-Bromo-6-methoxy-2-methyl-2,3-dihydro-1*H*-inden-1-one

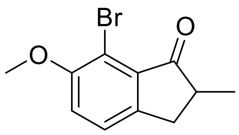

To a solution of 33.6 g (191 mmol) of 6-methoxy-2-methyl-2,3-dihydro-1*H*-inden-1-one in 1000 ml of DMF a solution of 33.9 g (191 mmol) of *N*-bromosuccinimide in 200 ml of DMF was added dropwise for 12 h, and the obtained mixture was stirred overnight. Then the resulting solution was poured into 2 L of water and extracted with dichloromethane (3 x 200 ml). The combined organic extracts were washed with saturated aqueous solution of Na_2SO_3, dried over Na_2SO_4 and evaporated. The residue was washed on a glass frit (G3) with hexane (3 x 40 ml) and dried in vacuo. Yield: 20.9 g (43%) of a the product as a white powder. ^{1}H NMR (400 MHz, $CDCl_3$): δ7.33 (d, *J* = 8.3 Hz, 1H), 7.14 (d, *J* = 8.3 Hz, 1H), 3.92 (s, 3H), 3.27 (dd, *J* = 16.6 Hz, *J* = 8.0 Hz, 1H), 2.79–2.68 (m, 1H), 2.59 (dd, *J* = 16.6 Hz, *J* = 4.3 Hz, 1H), 1.30 (d, *J* = 7.3 Hz, 3H). ^{13}C{^{1}H} NMR (101 MHz, $CDCl_3$): δ 206.7, 155.6, 147.4, 134.6, 125.7, 118.2, 108.4, 57.0, 43.5, 33.1, 16.3.

6-Methoxy-2-methyl-7-phenyl-2,3-dihydro-1*H*-inden-1-one

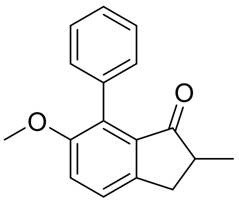

To a solution of 10.0 g (39.2 mmol) of 7-bromo-6-methoxy-2-methyl-2,3-dihydro-1*H*-inden-1-one in 100 ml of 1,4-dioxane 7.17 g (58.8 mmol) of phenylboronic acid, 38.3 g (118 mmol) of cesium carbonate and 50 ml of water were subsequently added. The obtained mixture was purged with argon for 10 min followed by addition of 2.26 g (1.97 mmol) of $Pd(PPh_3)_4$. This mixture was stirred for 12 h at 90 °C, then cooled to room temperature and diluted with 200 ml of water. The resulting mixture was extracted with dichloromethane (3 x 100 ml). The combined organic extracts were dried over Na_2SO_4 and then evaporated to dryness. The residue was purified by flash chromatography on silica gel 60 (40-63 µm, eluent: hexane-ethyl acetate = 10:1, vol). Yield: 8.74 g (88%) of the product as a white solid. ^{1}H NMR (400 MHz, $CDCl_3$): δ7.50–7.38 (m, 4H), 7.38–7.32 (m, 2H), 7.27 (d, *J* = 8.4 Hz, 1H), 3.79 (s, 3H), 3.43–3.31 (m, 1H), 2.77–2.65 (m, 2H), 1.28 (d, *J* = 7.2 Hz, 3H). ^{13}C{^{1}H} NMR (101 MHz, $CDCl_3$): δ 208.0, 156.0, 145.6, 134.1, 133.6, 129.8, 128.9, 127.4, 127.3, 126.0, 117.9, 56.5, 43.2, 33.5, 16.0.

5-Methoxy-2-methyl-4-phenyl-1*H*-indene

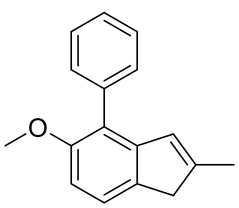

To a solution of 8.74 g (34.6 mmol) of 6-methoxy-2-methyl-7-phenyl-2,3-dihydro-1*H*-inden-1-one in 50 ml of THF cooled to 5 °C 1.96 g (52.0 mmol) of $NaBH_4$ was added portion wise. Further on, 25 ml of methanol was added dropwise to this mixture at 5 °C for 1 h. Then, the solvents were removed in vacuum, and to the residue dichloromethane (200 ml) and water (100 ml) were added. The mixture was acidified to pH 5 with 4M HCl. The organic layer was separated, the aqueous layer was additionally extracted with dichloromethane (2 x 50 ml). The combined organic phase was dried over Na_2SO_4 and evaporated. The residue was dissolved in 100 ml of toluene and to the resulting solution TosOH (0.04 g) was added. The mixture was refluxed with Dean-Stark head for 10 min, and then cooled to room temperature using external ice-water bath. The resulting solution was passed through a short layer of silica gel 60 (40–63 µm), the silica gel layer was additionally washed with 100 ml of dichloromethane. The combined organic filtrate was evaporated and the residue was additionally purified by flash chromatography on silica gel 60 (40–63 µm; eluent: hexane-ethyl acetate = 5:1). Yield: 7.79 g (95%) of a white crystalline solid. ^{1}H NMR (400 MHz, $CDCl_3$): δ 7.53 - 7.44 (m,

4H), 7.44 - 7.36 (m, 1 H), 7.33 (d, J = 8.1 Hz, 1 H), 6.79 (d, J = 8.1 Hz, 1 H), 6.41 (br.s., 1 H), 3.81 (s, 3 H), 3.37 (s, 2 H), 2.15 (s, 3 H). $^{13}C\{^1H\}$ NMR (101 MHz, $CDCl_3$): δ155.4, 147.6, 146.1, 136.8, 135.6, 130.4, 127.8, 126.6, 126.4, 123.0, 122.5, 106.5, 56.0, 42.3, 16.9.

Bis(5-methoxy-2-methyl-4-phenyl-1*H*-inden-1-yl)dimethylsilane

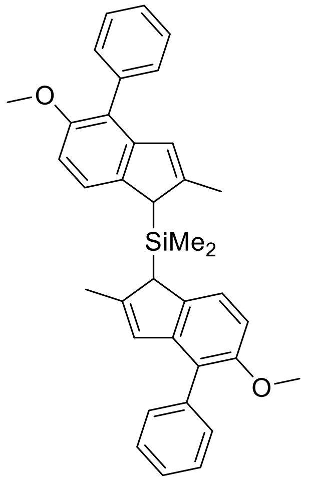

To a solution of 7.45 g (31.5 mmol) of 5-methoxy-2-methyl-4-phenyl-1*H*-indene in 200 ml of diethyl ether 12.6 ml (31.5 mmol) of 2.5 M ^{n}BuLi in hexanes was added in one portion at –40 °C. This mixture was stirred overnight at room temperature, then the resulting solution was cooled to –78 °C, and 50 mg of *N*-methylimidazole was added. The resulting mixture was stirred for 5 min at –78 °C, then 2.03 g (15.6 mmol) of dichlorodimethylsilane was added in one portion. Further on, this mixture was stirred overnight at ambient temperature. The resulting mixture was filtered through a short pad of silica gel 60 (40–63 µm) which was additionally washed by 2 x 30 ml of dichloromethane. The combined filtrate was evaporated, and the residue was purified by flash chromatography on silica gel 60 (40–63 µm, eluent: hexane-dichloromethane = 1:1, vol.). Yield: 5.69 g (68%) of the product as a yellowish glassy solid. ^1H NMR (400 MHz, $CDCl_3$): δ7.50–7.42 (m, 18H), 7.40–7.34 (m, 4H), 7.32 (d, J = 8.3 Hz, 1H), 6.80 (d, J = 8.2 Hz, 2H), 6.79 (d, J = 8.3 Hz, 2H), 6.52 (br.s, 4H), 3.80 (s, 6H), 3.79 (s, 6H), 3.73 (br.s, 4H), 2.22 (s, 6H), 2.15 (s,6H), –0.18 (s,3H),–0.19 (s, 6H), -0.19 (s, 3H).$^{13}C\{^1H\}$ NMR (101 MHz, $CDCl_3$): δ154.5, 154.5, 148.8, 148.6, 145.6, 145.5, 137.4, 137.3, 137.0, 130.5, 127.8, 126.6, 126.2, 126.1, 122.8, 122.7, 122.4, 122.4, 106.4, 106.3, 56.1, 56.0, 46.7, 46.6, 18.0, 18.0, –5.9, –6.0.

***rac*-Dimethylsilanediyl-bis(η^5-5-methoxy-2-methyl-4-phenyl-inden-1-yl)zirconium dichloride (M41)**

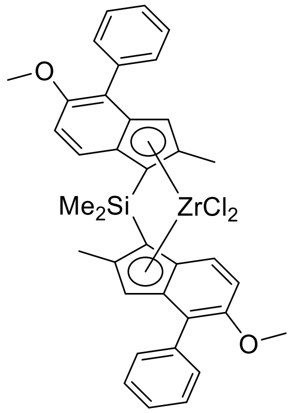

To a cooled to –40 °C solution of 3.97 g (7.51 mmol) of bis(5-methoxy-2-methyl-4-phenyl-1*H*-inden-1-yl)dimethylsilane in 250 ml of diethyl ether 6.0 ml (15.0 mmol) of 2.5 M ^{n}BuLi in hexanes was added in one portion. This mixture was stirred overnight at room temperature. The resulting solution was cooled to –78 °C and 2.83 g (7.51mmol) of $ZrCl_4(THF)_2$ was added. The reaction mixture was stirred for 24 h and then evaporated. The residue was taken up in 100 ml of hot toluene, and the formed mixture was filtered through a short pad of Celite 503. The filtrate was evaporated to dryness, giving a ca. 1:1 mixture of *rac*- and *meso*-complexes. This crude product was recrystallized from toluene yielding 0.22 g (4%) of *rac*-complex as orange crystalline solid. Anal. calc. for $C_{36}H_{34}Cl_2O_2SiZr$: C, 62.77; H, 4.98. Found: C, 63.16; H, 5.05. ^1H NMR (400 MHz, $CDCl_3$): δ 7.62 (d, J = 9.4 Hz, 2H), 7.58 (m,4H), 7.46–7.42 (m, 4H), 7.36–7.30 (m, 2H), 6.99 (d, J = 9.4 Hz, 2H), 6.62 (s, 2H), 3.77 (s, 6H), 2.21 (s, 6H), 1.30 (s, 6H).$^{13}C\{^1H\}$ NMR (101 MHz, $CDCl_3$): δ 156.6, 136.2, 135.6, 135.5, 129.9, 128.1, 127.2, 125.8, 123.4, 122.1, 120.7, 117.2, 81.9, 58.7, 18.5, 2.5.

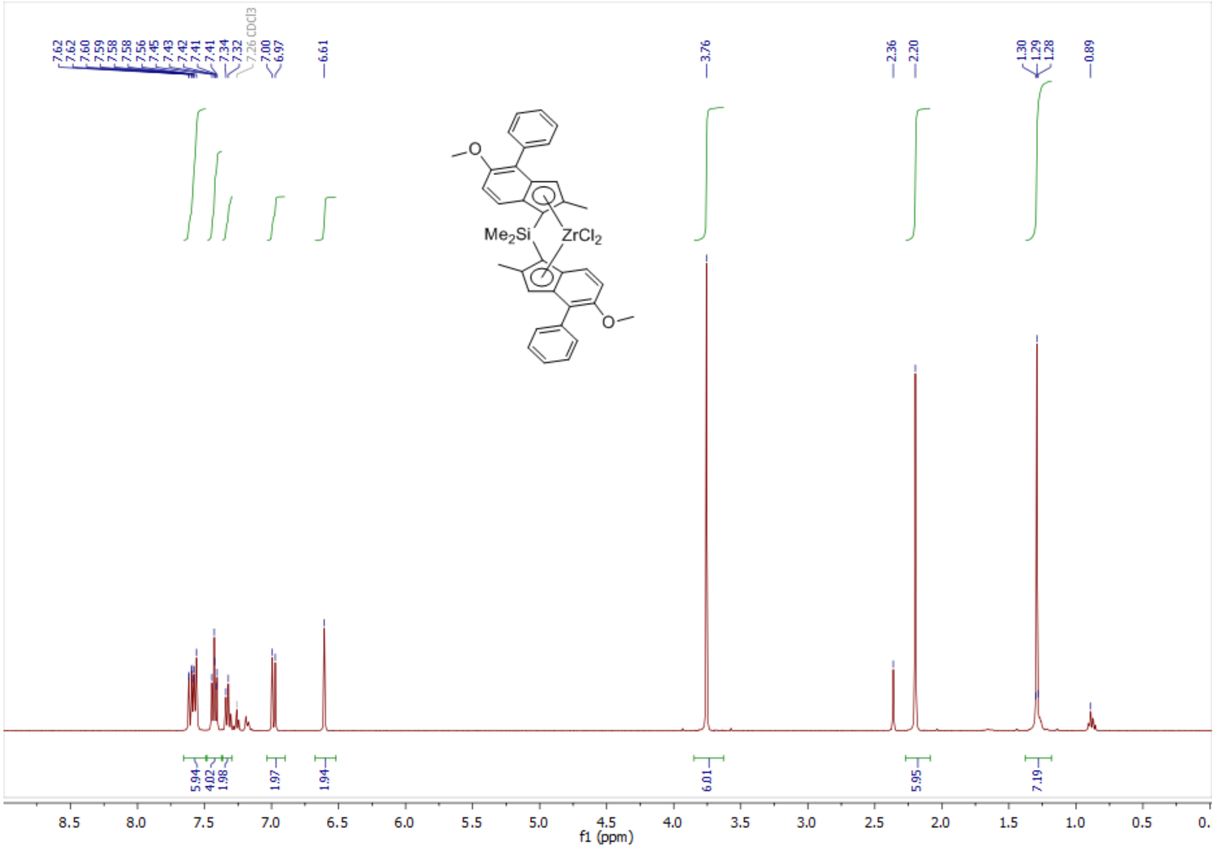

Figure S33.^{1}H NMR spectrum of **M41** in CDCl₃. Traces of toluene and hexane are visible at 2.36 and 0.89 ppm, respectively.

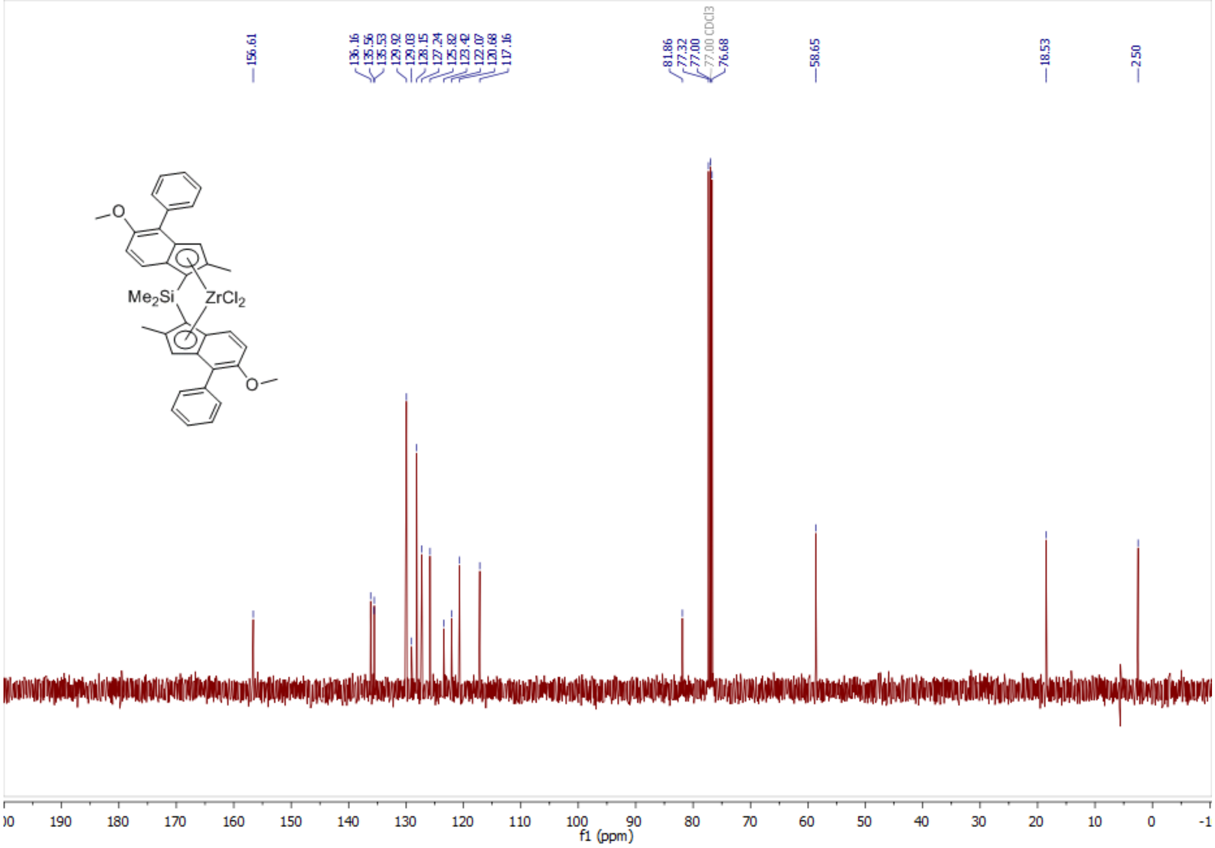

Figure S34.^{13}C{^{1}H} NMR spectrum of **M41** in CDCl₃.

M42

Bis(4-(3,5-bis(trimethylsilyl)phenyl)-2-methyl-1*H*-inden-1-yl)dimethylsilane

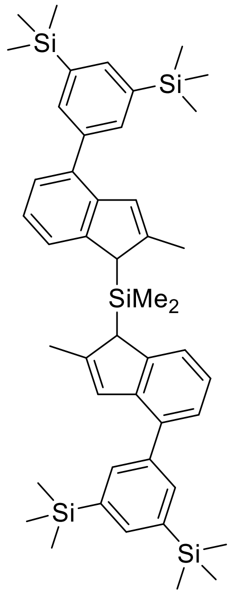

To a solution of (3,5-bis(trimethylsilyl)phenyl)lithium in 500 ml of THF, prepared from 9.80 g (32.5 mmol) of (5-bromo-1,3-phenylene)bis(trimethylsilane) and 36.1 ml (65.0mmol) of 1.8 M ^{t}BuLi in pentane at –78 °C, 4.78 g (35.1 mmol) of $ZnCl_2$ was added in one portion. The obtained mixture was allowed to warm to room temperature and then stirred overnight. To the resulting organozinc compound 6.17 g (13.0 mmol) of bis(4-bromo-2-methyl-1*H*-inden-1-yl)dimethylsilane, 0.48 g (0.52 mmol) of $Pd_2(dba)_3$ and 0.42 g (20.8 mmol) of P^tBu_3 was subsequently added. The reaction mixture was stirred overnight at 60 °C, then cooled to room temperature, poured into 1000 ml of water and extracted with 3 x 200 ml of diethyl ether. The combined extracts were passed through a short pad of silica gel 60 (40–63 µm) and then evaporated. The product was isolated by flash-chromatography on silica gel 60 (40–63 µm; eluent: hexanes-dichloromethane = 20:1, vol.). Yield: 4.79 g (47%) of an yellowish glassy solid.^{1}H NMR (400MHz, $CDCl_3$): δ 7.83–7.76 (m, 12H), 7.62 (d, J = 7.6 Hz, 2H), 7.49 (d, J = 7.5 Hz, 2H), 7.40 (m, 4H), 7.33–7.25 (m, 4H), 6.92 (s, 2H), 6.90 (s, 2H), 3.95 (s, 2H), 3.93 (s, 2H), 2.36 (s, 6H), 2.35 (s, 6H), 0.43 (2s, sum 72H), –0.07 (s, 3H), –0.10 (s, 3H),–0.13 (s, 6H).^{13}C{^{1}H} NMR (101 MHz, $CDCl_3$): δ 147.8, 147.7, 145.5, 145.4, 143.1, 143.1, 139.6, 139.6, 139.4, 136.5, 134.7, 134.7, 134.5, 126.0, 126.0, 125.7, 125.7, 123.2, 123.1, 122.2, 122.1, 47.6, 47.6, 18.2, 18.1, –1.0, –5.7, –5.8, –6.2.

***rac*-Dimethylsilanediyl-bis[η^5-4-(3,5-bis(trimethylsilyl)phenyl)-2-methylinden-1-yl]zirconium dichloride (M42)**

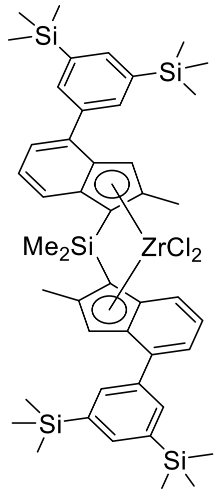

To a cooled to –40 °C solution of 4.61 g (6.09 mmol) of bis(4-(3,5-bis(trimethylsilyl)phenyl)-2-methyl-1*H*-inden-1-yl)dimethylsilane in 250 ml of ether 4.87 ml (12.2 mmol) of 2.5 M ^{n}BuLi in hexanes was added in one portion. This mixture was stirred overnight at room temperature. The resulting solution was cooled to –78 °C and 2.30 g (6.09 mmol) of $ZrCl_4(THF)_2$ was added. The reaction mixture was stirred for 24 h and then evaporated to dryness. The residue was taken up in 100 ml of hot toluene, and the formed mixture was filtered through a short pad of Celite 503. The filtrate was evaporated to dryness, giving a ca. 1:1 mixture of *rac*- and *meso*-complexes. This crude product was crystallized from methylcyclohexane/hexane mixture (1:1, vol.) at –30 °C yielding 0.17 g (3%) of *rac*-complex as a yellow powder. Anal. calc. for $C_{46}H_{62}Cl_2Si_5Zr$: C, 60.22; H, 6.81. Found: C, 60.54; H, 6.91. ^{1}H NMR (400 MHz, $CDCl_3$): δ 7.84 (s, 4 H), 7.74–7.66 (m, 4 H), 7.46 (d, J = 6.9 Hz, 2 H), 7.17 (m, 2H), 6.97 (s, 2 H), 2.30 (s, 6 H), 1.37 (s, 6 H), 0.29 (s, 36 H). ^{13}C{^{1}H} NMR (101 MHz , $CDCl_3$): δ 139.9, 139.3, 137.9, 137.6, 135.7, 134.3, 132.5, 127.6, 126.3, 125.9, 124.2, 122.5, 83.7, 18.6, 2.6, –1.0.

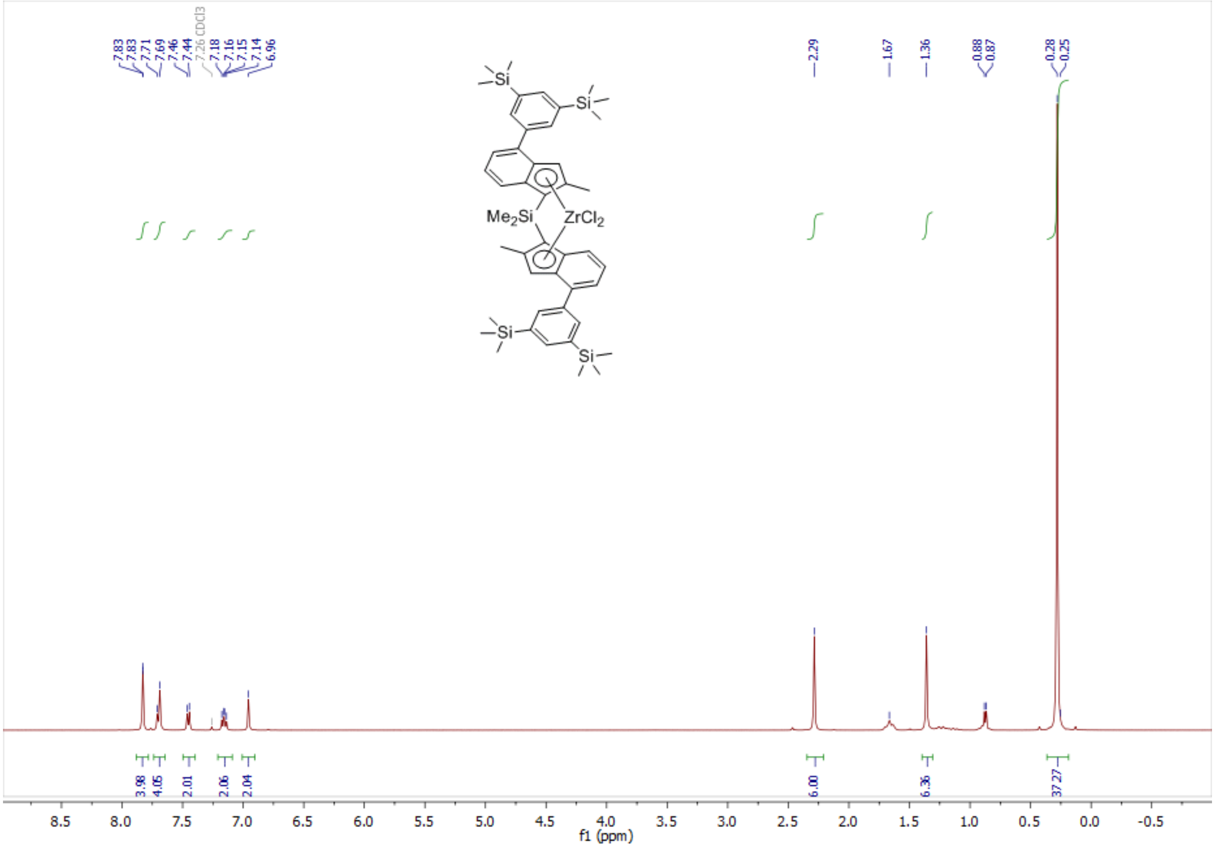

Figure S35. ^{1}H NMR spectrum of **M42** in $CDCl_3$. Traces of methylcyclohexane are visible at 0.88 ppm.

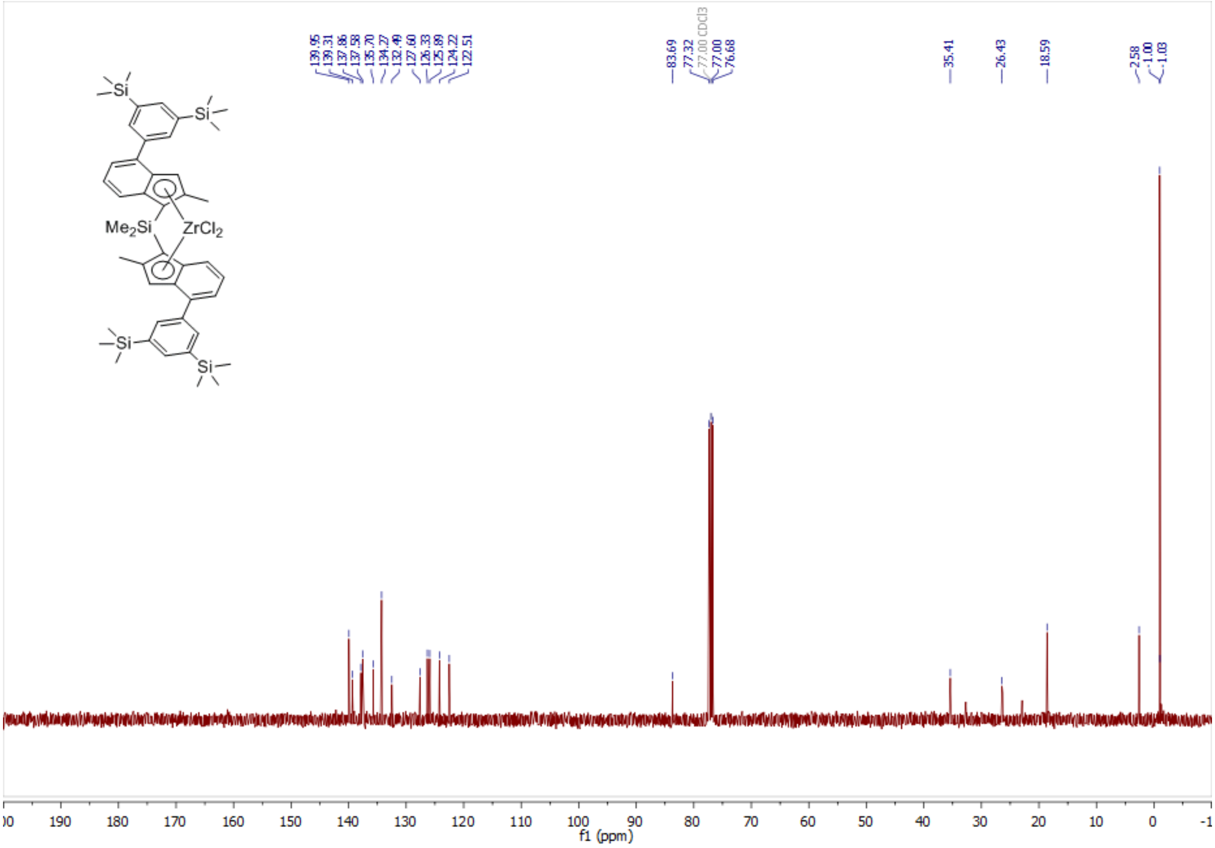

Figure S36. $^{13}C\{^{1}H\}$ NMR spectrum of **M42** in $CDCl_3$.

M43

2,6-Di(adamantan-1-yl)-4-bromophenol

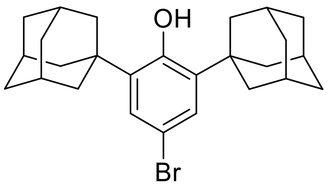

To a mixture of 17.3 g (100 mmol) of 4-bromophenol and 30.4 g (200 mmol) of adamantan-1-ol in 350 ml of dichlorobenzene 19.2 g (200 mmol) of methanesulfonic acid was added dropwise for 30 min at room temperature. Then the obtained solution was stirred for 5 days at 60°C. The obtained mixture was poured into 1 L of 10% aqueous NH_3 and then extracted with dichloromethane (3 x 200 ml). The combined organic extracts were washed with 300 ml of water, dried over Na_2SO_4, passed through a short pad of silica gel and evaporated. The remaining dichlorobenzene was removed under vacuum (1 mbar). The residue was dried using a Kugelrohr apparatus, 150 °C/0.5 mbar. The remaining solid was purified by flash chromatography on silica gel 60 (40-63 µm, eluent: hexane). Yield: 32.9 g (75%) of a white powder. ^{1}H NMR (600MHz, $CDCl_3$): δ 7.21 (s, 2H), 5.34 (s, 1H), 2.13–2.07 (br.s, 18 H), 1.84–1.74 (m, 12H). ^{13}C{^{1}H} NMR (151MHz, $CDCl_3$): δ 153.6, 138.1, 127.8, 113.3, 41.0, 36.9, 36.9, 28.9.

1,1'-(5-Bromo-2-methoxy-1,3-phenylene)bis(adamantane)

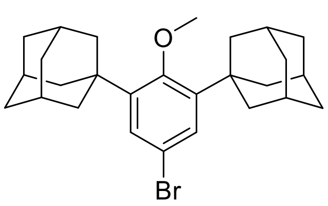

To a suspension of 3.03 g of NaH (60% in mineral oil, 1.82 g, 75.8mmol) in 200 ml of dry THF a solution of 22.3 g (50.5 mmol) of 2,6-di(adamantan-1-yl)-4-bromophenol in 100 ml of dry THF was added dropwise at 0 °C for 2 h. After complete addition, the reaction mixture was allowed to warm to room temperature and stirred for 30 min. Then 7.01 g (55.6 mmol) of Me_2SO_4 was added dropwise and the obtained mixture was stirred at 65 °C for 12 h. Further on, the excess of NaH was quenched with50 ml of water at 0 °C. Then the resulting mixture was diluted with additional 2000 ml of water and extracted with hexane (3 x 100 ml). The combined organic extracts were washed 100 ml of 30% aqueous NH_3, water (2 x 100 ml), passed through a short pad of silica gel and evaporated. The residue was purified by flash chromatography on silica gel 60 (40-63 µm, eluent: hexane). Yield: 20.8 g (90%) of yellowish powder. ^{1}H NMR (600MHz, $CDCl_3$): δ 7.33 (s, 2H), 3.67 (s, 3H), 2.10 (br.s, 18H), 1.78 (br.s., 12H). ^{13}C{^{1}H} NMR (151 MHz, $CDCl_3$): δ 159.7, 146.0, 129.2, 116.8, 65.9, 42.5, 38.7, 36.8, 29.2.

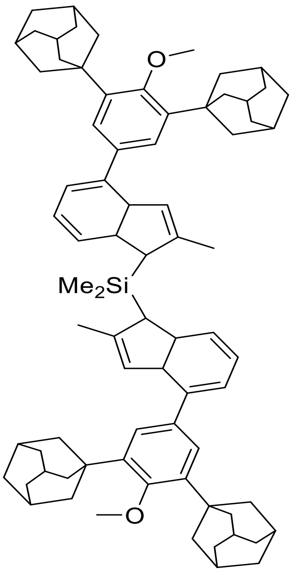

Bis(4-(3,5-di(adamantan-1-yl)-4-methoxyphenyl)-2-methyl-1*H*-inden-1-yl)dimethylsilane

To a solution of (3,5-di(adamantan-1-yl)-4-methoxyphenyl)lithium in 500 ml of THF, prepared from 13.0 g (28.6mmol) of 1,1'-(5-bromo-2-methoxy-1,3-phenylene)bis(adamantane) and 31.8 ml (57.2mmol) of 1.8 M ^{t}BuLi in pentane at –78 °C, 4.43 g (32.5 mmol) of $ZnCl_2$ was added in one portion. The resulting mixture was allowed to warm to room temperature and then stirred overnight. To the resulting organozinc compound 6.17 g (13.0 mmol) of bis(4-bromo-2-methyl-1*H*-inden-1-yl)dimethylsilane, 0.24 g (0.26mmol) of $Pd_2(dba)_3$ and 0.21 g (10.4mmol) of P^tBu_3 was subsequently added. The reaction mixture was stirred overnight at 60 °C, then cooled to room temperature, poured into 1000 ml of water and then extracted with 3 x 200 ml of diethyl ether. The combined extracts were passed through a short pad of silica gel 60 (40–63 µm) and then evaporated. The product was isolated by flash chromatography on silica gel 60 (40–63

μm; eluent: hexanes-dichloromethane = 5:1, vol.). Yield: 6.00 g (43%) of the product as a yellowish glassy solid. ^{1}H NMR (400 MHz, $CDCl_3$): *δ* 7.54 (d, *J* = 7.6 Hz, 2H), 7.47 (s, 4H), 7.46 (s, 4H), 7.41 (d, *J* = 7.3 Hz, 1H), 7.35–7.31 (m, 4H), 7.25–7.18 (m, 4H), 6.88 (s, 2H), 6.87 (s, 2H), 3.90 (s, 2H), 3.86 (s,2H), 3.81 (s, 6H), 3.81 (s, 6H), 2.34 (s, 6H), 2.32 (s, 6H), 2.30–2.20 (br.s, 48H), 2.10–2.06 (br.s, 24H), 1.92–1.74 (m, 48H), –0.10 (s, 3H),–0.16 (s, 3H),–0.17 (s, 6H). ^{13}C{^{1}H} NMR (101 MHz, $CDCl_3$): *δ* 159.4, 147.4, 147.2, 145.4, 145.4, 143.3, 142.8, 142.8, 135.5, 135.5, 134.9, 134.9, 126.8, 126.8, 126.3, 126.2, 125.6, 125.5, 123.0, 122.9, 121.7, 121.7, 65.7, 47.5, 42.7, 38.6, 37.0, 29.3, 18.2, 18.2, –5.5, –5.7, –6.0.

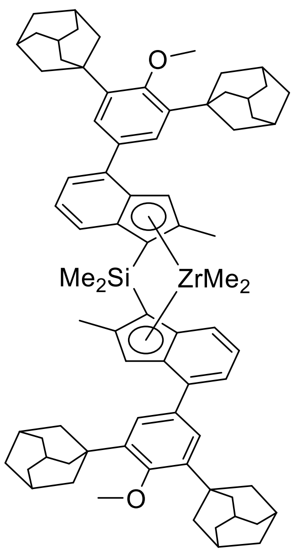

***rac*-Dimethylsilanediyl-bis[η^5-4-(4-methoxy-3,5-diadamantylphenyl)-2-methylinden-1-yl]dimethylzirconium (M43)**

To a solution of bis(4-(3,5-di(adamantan-1-yl)-4-methoxyphenyl)-2-methyl-1H-inden-1-yl)dimethylsilane (2.50 g, 2.35 mmol) in ether (150 ml) cooled to –40 °C *n*BuLi (1.90 ml of 2.5 M in hexanes, 4.69 mmol) was added in one portion. The mixture was stirred overnight at room temperature, then evaporated to near dryness. The resulting foam was cooled to –100 °C and 100 ml of precooled dichloromethane was added via cannula. To the resulting solution 547 mg (2.35 mmol) of $ZrCl_4$ was added in one portion. The resulting suspension was stirred for 12 h and then the solvents were removed in vacuum. The residue was taken up in toluene (100 ml), and the formed suspension was filtered through a glass frit (G4). The filtrate was evaporated in vacuum to dryness. To the residue 50 ml of toluene and MeMgBr (2.43 ml, 7.05 mmol, 2.9M in ether) were subsequently added. The resulting suspension was stirred for 12 h at 60 °C and then the solvents were removed in vacuum. The residue was taken up in hexane (100 ml), and the formed suspension was filtered through a glass frit (G4). The filtrate was evaporated in vacuum to dryness. Recrystallization of the solid residue from 5 ml of hexane at -30 °C afforded 117mg (4%) of the complex as a light-yellow powder (*rac*/*meso* = 11/1). Anal. calc. for $C_{78}H_{96}O_2SiZr$: C, 79.06; H, 8.17. Found: C, 79.35; H, 8.34. ^{1}H NMR (400 MHz, C_6D_6): δ 7.86 (s, 4H), 7.46–7.51 (m, 4H), 7.25 (s, 2H), 6.93–6.97 (m, 2H), 3.51 (s, 6H), 2.28–2.42 (m, 24H), 2.03–2.15 (m, 12H), 1.97 (s, 6H), 1.70–1.86 (m, 24H), 0.79 (s, 6H), –0.41 (s, 6H). ^{13}C{^{1}H} NMR (101MHz, C_6D_6): *δ* 160.9, 144.6, 140.1, 136.0, 135.1, 129.8, 129.3, 127.5, 125.0, 124.8, 124.6, 116.6, 79.6, 65.9, 43.6, 39.5, 37.6, 36.6, 30.2, 18.5, 2.9.

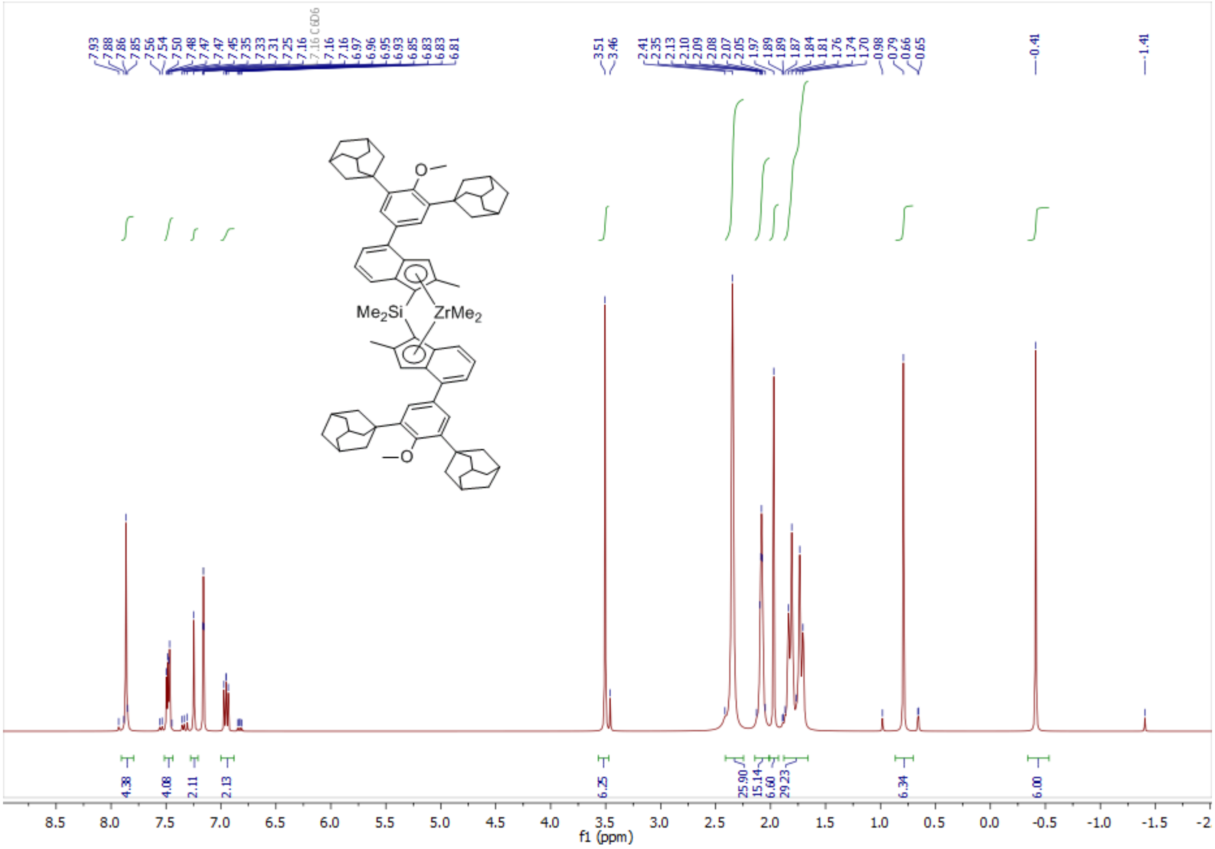

Figure S37.^{1}H NMR spectrum of **M43** (*rac*/*meso* = 11/1) in C_6D_6.

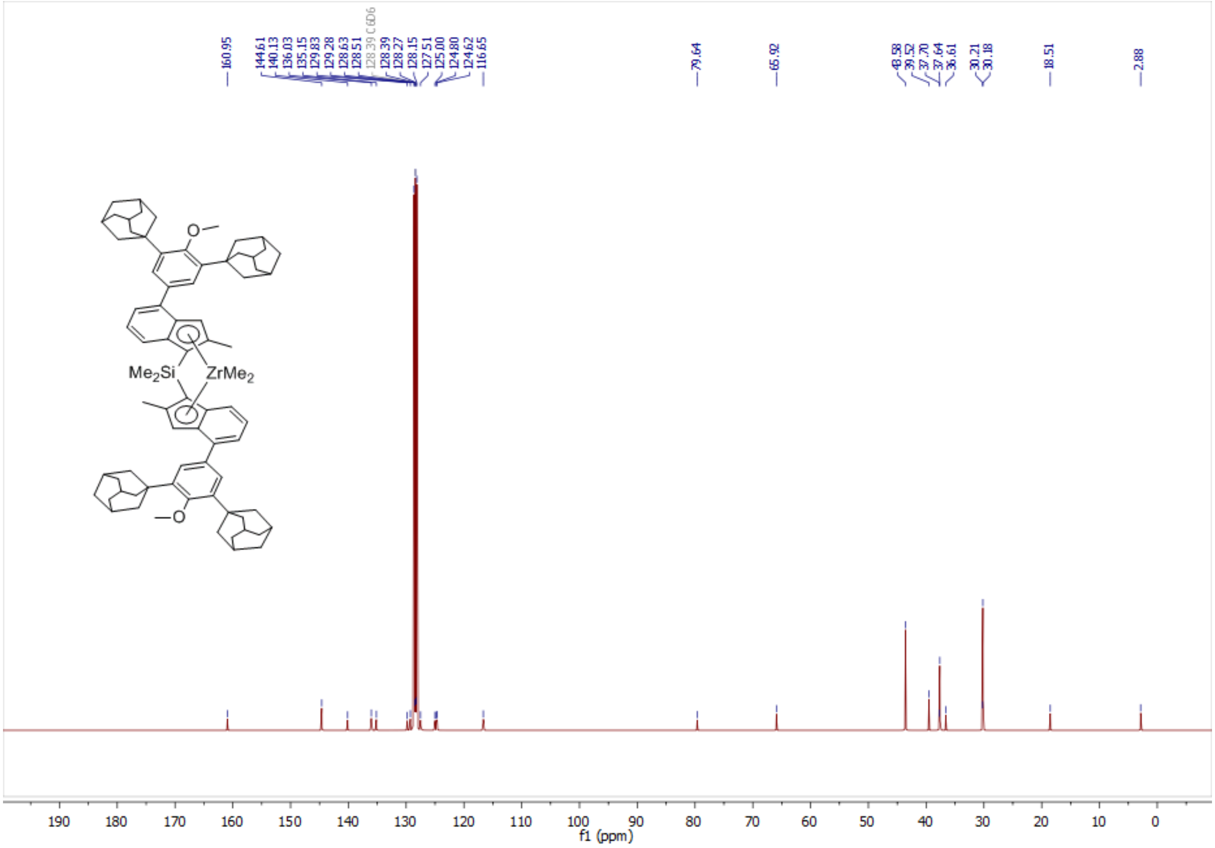

Figure S38.^{13}C{^{1}H} NMR spectrum of **M43** (*rac*/*meso* = 11/1) in C_6D_6.

References

[1] Nikulin, M. V.; Tsarev, A. A.; Lygin, A. V.; Ryabov, A. N.; Beletskaya, I. P.; Voskoboinikov, A. Z., Palladiumcatalyzed arylationof bis(4-bromo-2-methylinden-1-yl)dimethylsilane and related compounds, *Russ. Chem. Bull*. **2008**, *57*, 2298–2306.

[2] Resconi, L.; Castro, P.; Virkkunen, V.; Izmer, V. V.; Kononovich, D. S.; Kulyabin, P. S.; Voskoboynikov, A. Z. *Int. Pat. Appl. WO 2014096164A1.*

[3] Resconi, L.; Virkkunen, V.; Noureddine, A.; Castro, P.; Izmer, V.; Kononovich, D.; Voskoboynikov, A. *Int. Pat. Appl. WO 2014096282A1.*

[4] Resconi, L.; Castro, P.; Virkkunen, V.; Izmer, V. V.; Kononovich, D. S.; Kulyabin, P. S.; Voskoboynikov, A. Z. *Int. Pat. Appl. WO2014096166A1.*

[5] Voskoboynikov, A. Z., Asachenko, A. F., Kononovich, D. S., Nikulin, M. V., Tsarev, A. A., Maaranen, J., Vanne, T., Kauhanen, J., Mansner, E., Kokko, E., Saarinen, L. *Int. Pat. Appl. WO2009027075A1.*

[6] Voskoboynikov, A. Z.; Ryabov, A. N.; Nikulin, M. V.; Lygin, A. V.; Coker, C. L.; Canich, J. A. M. *Int. Pat. Appl. WO 2007070041A1.*

[7] Izmer, V. V.; Lebedev, A. Y.; Nikulin, M. V.; Ryabov, A. N.; Asachenko, A. F.; Lygin, A. V.; Sorokin, D. A.; Voskoboynikov, A. Z., Palladium-Catalyzed Pathways to Aryl-Substituted Indenes: Efficient Synthesis of Ligands and the Respective ansa-Zirconocenes. *Organometallics***2006**, *25*, 1217-1229.

[8] Schöbel, A.; Herdtweck, E.; Parkinson,M.; Rieger, B., Ultra-Rigid Metallocenes for Highly Iso- and Regiospecific Polymerization of Propene: The Search for the Perfect Polypropylene Helix. *Chem. Eur. J.* **2012**, *18*, 4174–4178.

[9] Resconi, L.; Focante, F.; Balboni, D.; Nifant'ev, I. E.; Ivchenko, P. V.; Bagrov V.; *Int. Pat. Appl. WO2007116034A1.*

[10] Fujita, T.; Fukuoka, O.; Imuda, J.; Kawai, K.; Kiso, Y.; Nitahara, M.; Saito, J.; Tashiro, K.; Ueda, T.; Yoshida, M. *Japan. Pat. Appl. JPH07286005A*, 1995.

[11] Canich, J. A. M.; Jiang, P.; Casty, G. L.; Kiss, G.; Voskoboynikov, A. Z. U. S. Pat. US 7812104B2, 2010.

[12] Matsubara, K.; Ueno, K.; Shibata, Y.; Synthesis and Structures of Nickel Halide Complexes Bearing Mono- and Bis-coordinated *N*-Heterocyclic Carbene Ligands, Catalyzing Grignard Cross-Coupling Reactions, *Organometallics*, **2000**, *25*, 3422–3427.

[13] Zhang, X.; Zhu, Q.; Guzei, I. A.; Jordan, R. F.; General Synthesis of Racemic Me_2Si-Bridged Bis(indenyl) Zirconocene Complexes, *J. Am. Chem. Soc.***2000**, *122*, 8093–8094.

[14] Sevov, C.S.; Hartwig, J.F., Iridium-Catalyzed Oxidative Olefination of Furans with Unactivated Alkenes, *J. Am. Chem. Soc.* **2014**, *136*, 10625–10631.

[15] Sam, J.; Plamp, J.N., Hypotensive Basic Ethers in the Indan Series, *J. Am. Chem. Soc.* **1960**, *82*, 5205–5209.

2. Polymerization Details

Prior to the execution of a polymerization library, the PPR modules undergo 'bake-and-purge' cycles overnight (8 h at 90-140°C with intermittent dry N_2 flow), to remove any contaminants and left-overs from previous experiments. After cooling to glove-box temperature, the module stir tops are taken off, and the 48 cells are fitted with disposable 10 mL glass inserts (pre-weighed in a Mettler-Toledo Bohdan Balance Automator) and polyether ether ketone (PEEK) stir paddles. The stir tops are then set back in place, and the cells are loaded with the appropriate amounts of toluene solvent and triisobutylaluminum (TIBA, 10 μmol) scavenger, thermostated at 60 °C, and brought to 95 psi of pressure with propene. At this point, the catalyst injection sequence is started; aliquots of (a) a toluene 'chaser', (b) a toluene solution of catalyst (variable amount, see Table S1), (c) a toluene solution of the proper activator ($HNMe_2Ph^+[B(C_6F_5)_4]^-$ or $[Ph_3C^+]$ $[B(C_6F_5)_4]^-$, the first one was used in 2-fold excess with respect to catalyst, the second one was used in 5-10-fold excess), (d) a toluene 'buffer', all separated by nitrogen gaps, are uploaded into the slurry needle and subsequently injected into the cell of destination, thus starting the reaction. This is left to proceed under stirring (800 rpm) at constant temperature and pressure with continuous feed of propene on demand until the desired monomer consumption has been reached (for reaction time, see Table S1), and quenched by over-pressurizing the cell with 50 psi (3.4 bar) of dry air (preferred over other possible catalyst quenchers because in case of cell or quench line leakage oxygen is promptly detected by the dedicated glove-box sensor). Once all cells have been quenched, the modules are cooled down and vented, the stir-tops are removed, and the glass inserts containing the reaction phases are taken out and transferred to a centrifugal evaporator (Genevac EZ-2 Plus or Martin Christ RVC 2-33 CDplus), where all volatiles are distilled out and the polymers are thoroughly dried overnight.

Reaction yields are double-checked against on-line monomer conversion measurements by robotically weighing the dry polymers while still in the reaction vials, subtracting the pre-recorded tare. Polymer aliquots are then sent to the characterizations.

GPC curves were recorded with a Freeslate Rapid GPC setup, equipped with a set of two mixed-bed Agilent

PLgel 10 μm columns and a Polymer Char IR4 detector. Calibration was performed with the universal method, using 10 monodisperse polystyrene samples (M_n between 1.3 and 3700 kDa).

^{13}C NMR spectra were recorded with a 100 MHz Bruker Avance III 400 spectrometer equipped with a 5 mm high-temperature cryoprobe. Polymer samples (~25 mg) were dissolved at 120 °C in

tetrachloroethane-1,2-d_2 (0.5 mL) with 0.40 mg mL^{-1} BHT (2,6-di-*tert*-butyl-4-methylphenol) as stabilizer. DSC curves were obtained with a differential scanning calorimeter (DSC-822 by Mettler Toledo) in a flowing N_2 atmosphere at a scanning rate of 10 °C/min from 0 to 200 °C. Polymer melting points (T_m) were collected from the second heating run.

Table S1. Propene Polymerization Experiments.

Activity (R_{pAV}) given in kg $mmol^{-1}$ h^{-1}. For experimental data for **M1-M19**: *Macromolecules* **2018**, *51*, 8073-8083. Updated data provided for **M3** in the table. Except for dimethyl precursors (**M20**, **M31** and **M43**) activity values for AB are given for completeness and are not indicative of activity trends; the reaction rate was intentionally slowed down, see main paper for more details.

Catalyst	Activator	n_{cat} (nmol)	*t* (s)	Yield (mg)	R_{pAV}
**M20**	AB	3.75	1200	28	60
		5	496	67	
**M21**	AB	20	2456	59	5
		30	1397	57	
**M22**	AB	25	1462	156	14
		25	1742	142	
**M23**	AB	15	894	34	15
		15	716	66	
**M24**	AB	20	2578	143	8
		20	3143	91	
**M25**	AB	20	3601	125	9
		20	2867	186	
**M26**	AB	20	1089	107	11
		20	2302	62	
**M27**	AB	20	4583	63	2
		30	6491	66	
**M28**	AB	30	3602	47	2
		20	3601	56	
**M29**	AB	40	3324	75	3
		40	3281	136	
**M30**	AB	40	1847	264	12
		40	1270	145	
	TTB	2	724	118	292
**M31**	AB	5	2075	21	25
		6	730	53	
**M32**	AB	20	1446	74	7
		20	1367	37	

Table S1, ctd. Propene Polymerization Experiments.

Catalyst	Activator	n_{cat} (nmol)	*t* (s)	Yield (mg)	R_{pAV}
**M33**	AB	30	2047	62	3
		35	3026	40	
	TTB	3	500	78	187
**M34**	AB	20	7014	26	7
		25	1174	103	
**M35**	AB	30	1237	63	5
		40	2308	83	
**M36**	AB	40	3824	83	2
		80	1580	50	
**M37**	AB	20	497	119	38
		15	896	122	
**M38**	AB	25	1844	22	2
		40	2229	40	
**M39**	AB	20	3714	61	5
		20	2707	114	
**M40**	AB	8	1520	41	1
		8	1544	41	
**M41**	AB	30	2186	69	3
		30	1832	37	
**M42**	AB	30	1671	77	5
		30	1364	55	
**M43**	AB	7.5	318	107	133
		2.5	974	70	
**M3**	AB	40	2009	65	5
		40	1428	119	

Table S2. Full Polymer Characterization.

Catalyst	Activator	NMR								T_m[b]	GPC		
		mmrrmm[a]	σ	σ_{Av}	*[mmmm]*[d]	*[2,1]*[a]	*[3,1]*[a]	*regio*$_{tot}$[a]	*regio*$_{tot,AV}$[a]		M_n[c]	M_w[c]	PDI
**M20**	AB	<0.01	>0.9999	**>0.9999**	>99.95	0.44	0.04	0.48	**0.48**	158.2	**573**	1289	2.2
		<0.01	>0.9999		>99.95	0.45	0.03	0.48			**642**	1386	2.2
**M21**	TTB	oligomers; maybe very slightly isotactic									**0.8**	1	1.3
											**0.8**	1	1.3
**M22**	AB	0.80	0.9916	**0.9917**	95.87	0.89	n.d.	0.89	**0.90**	143.2	**120**	247	2.1
		0.78	0.9918		95.97	0.90	n.d.	0.90			**132**	283	2.1
**M23**	AB	0.08	0.9992	**0.9991**	99.60	0.27	n.d.	0.27	**0.28**	159.7	**393**	854	2.2
		0.11	0.9989		99.45	0.26	0.02	0.28			**364**	841	2.3
**M24**	AB	0.10	0.9990	**0.9990**	99.50	0.52	0.03	0.55	**0.54**	156.1	**539**	1317	2.4
		0.10	0.9990		99.50	0.49	0.03	0.52			**724**	1582	2.2
**M25**	AB	0.11	0.9989	**0.9989**	99.45	0.64	0.05	0.69	**0.69**	153.4	**692**	1661	2.4
		0.11	0.9989		99.45	0.65	0.04	0.69			**729**	1760	2.4
**M26**	AB	0.09	0.9991	**0.9991**	99.55	0.40	0.03	0.43	**0.43**	158.2	**402**	869	2.2
		0.10	0.9990		99.50	0.40	0.03	0.43			**552**	1080	2.0
**M27**	AB	0.22	0.9978	**0.9978**	98.90	0.81	0.15	0.96	**0.98**	148.4	**535**	1060	2.0
		0.22	0.9978		98.90	0.84	0.16	1.00			**444**	1079	2.4
**M28**	AB	too short; sPP CE									**3.3**	6.1	1.8
											**3.3**	5.9	1.8
**M29**	AB	0.03	0.9997	**0.9997**	99.85	0.23	n.d.	0.23	**0.23**	162.2	**374**	898	2.4
		0.03	0.9997		99.85	0.23	n.d.	0.23			**417**	891	2.1
**M30**	AB	<0.02	>0.9998	**0.9997**	>99.90	0.66	0.05	0.71	**0.71**	156.0	**1392**	3028	2.2
		<0.04	>0.9996		>99.90	0.67	0.03	0.70			**1478**	3436	2.3
	TTB										**1405**	3461	2.5

[a] in mol%, [b] in °C, [c] in kDa, [d] calculated from σ.

Table S2, ctd. Full Polymer Characterization.

Catalyst	Activator	NMR								T_m[b]	GPC		
		mmrrmm[a]	*σ*	$σ_{Av}$	*[mmmm]*[d]	*[2,1]*[a]	*[3,1]*[a]	*regio*$_{tot}$[a]	*regio*$_{tot,AV}$[a]		M_n[c]	M_w[c]	PDI
**M31**	AB	0.32	0.9967	**0.9967**	98.36	1.41	n.d.	1.41	**1.41**	143.0	**284**	549	1.9
		0.32	0.9967		98.36	1.41	n.d.	1.41			**301**	602	2.0
**M32**	AB	0.31	0.9968	**0.9969**	98.41	0.94	0.11	1.05	**1.06**	148.0	**669**	1485	2.2
		0.31	0.9968		98.41	0.94	0.11	1.05			**580**	1327	2.3
**M33**	AB	0.05	0.9995	**0.9995**	99.75	0.33	n.d.	0.33	**0.30**	160.6	**1074**	2387	2.2
		0.06	0.9994		99.70	0.29	n.d.	0.29			**1028**	2459	2.4
	TTB	0.04	0.9996		99.80	0.29	n.d.	0.29			**749**	1795	2.4
**M34**	AB	<0.02	>0.9998	**>0.9998**	>99.90	0.64	0.18	0.82	**0.83**	154.3	**991**	2405	2.4
		<0.02	>0.9998		>99.90	0.64	0.20	0.84			**994**	2108	2.1
**M35**	AB	0.04	0.9996	**0.9996**	99.80	0.29	0.07	0.36	**0.36**	159.7	**499**	1136	2.3
		0.03	0.9997		99.85	0.30	0.05	0.35			**527**	1251	2.4
**M36**	AB	2.20	0.974	**0.9715**	87.66	n.d.	0.09	0.09	**0.09**	137.2	**18**	34	1.9
		2.40	0.969		85.43	n.d.	0.08	0.08			**20**	33	1.7
**M37**	AB	0.11	0.9989	**0.9989**	99.45	0.19	0.02	0.21	**0.20**	160.5	**214**	437	2.0
		0.12	0.9988		99.40	0.18	0.02	0.20			**222**	479	2.2
**M38**	AB	0.04	0.9996	**0.9996**	99.80	0.20	n.d.	0.20	**0.20**	161.5	**721**	1737	2.4
		0.04	0.9996		99.80	0.20	n.d.	0.20			**886**	1927	2.2
**M39**	AB	0.03	0.9997	**0.9996**	99.85	0.18	n.d.	0.18	**0.18**	161.7	**608**	1323	2.2
		0.05	0.9995		99.75	0.18	n.d.	0.18			**869**	1999	2.3
**M40**	AB	0.05	0.9995	**0.9995**	99.75	0.11	n.d.	0.11	**0.11**	163.1	**627**	1481	2.4
		0.05	0.9995		99.75	0.11	n.d.	0.11			**617**	1485	2.4
**M41**	AB	0.04	0.9996	**0.9996**	99.80	0.34	0.02	0.36	**0.36**	159.7	**781**	1702	2.2
		0.04	0.9996		99.80	0.33	0.02	0.35			**746**	1575	2.1
**M42**	AB	0.05	0.9995	**0.9995**	99.75	0.19	n.d.	0.19	**0.20**	161.6	**589**	1172	2.0
		0.06	0.9994		99.70	0.20	n.d.	0.20			**514**	1086	2.1
**M43**	AB	0.03	0.9997	**0.9997**	99.85	0.17	n.d.	0.17	**0.17**	162.6	**511**	1099	2.2
		0.03	0.9997		99.85	0.17	n.d.	0.17			**428**	947	2.2
**M3**	AB	0.77	0.992	**0.9920**	96.06	0.91	n.d.	0.91	**0.91**	143.3	**134**	314	2.3
		0.77	0.992		96.06	0.91	n.d.	0.91			**137**	293	2.1

Table S3. Performance of selected catalysts when activated with MAO.

Catalyst	*MAO ratio*	n_{cat} *(nmol)*	*t (s)*	*Yield (mg)*	R_p[a]
**M2**	15,000	2	400	70	**315**
**M14**	15,000	2	970	46	**85**
**M29**	10,000	3	1110	49	**53**
**M30**	7,500	4	2085	113	**49**
**M39**	15,000	2	764	64	**151**
**M40**	15,000	2	387	121	**566**
**M41**	500	30	3600	21	**1**[b]
**M42**	15,000	2	980	41	**65**
**M43**	15,000	2	1410	35	**47**

[a] in kg $mmol^{-1}$ h^{-1}. [b] **M41** shows a very long activation delay.

Experimental Trends -Deleterious Effect of 3-Position Substituents on Molar Mass Capability

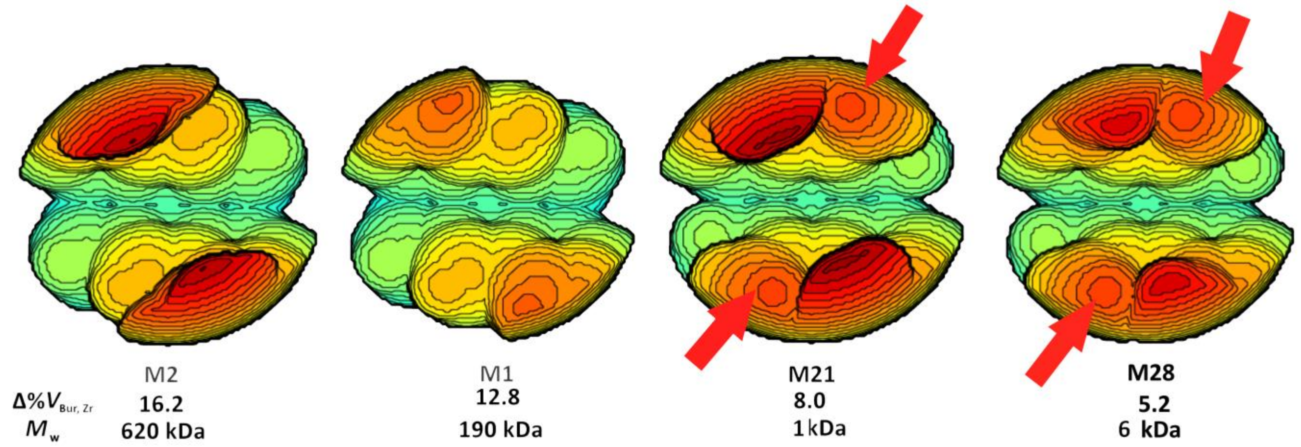

Figure S39. Steric heat maps of catalysts **M1**, **M2**, **M21** and **M28**. The deleterious effect of 3-position substitution – i.e. substituents in the open quadrant – on separation of steric bulk between the quadrants – and thus molecular weight capability – is clearly visible (indicated by red arrows). Heatmaps generated with SambVca 2.0, sphere size 5.0 Å.

3. QSAR Modeling

QSAR Modeling – General Comments

All metallocenes were optimized in ***C*₂-symmetry**.

Experimental temperature: **60°C**.

The **stereoselectivity and regioselectivity model does not include** catalysts **M21** and **M28** for which no stereoselectivity/regioselectivity could be determined experimentally.

M13 is neither included in the molecular weight model nor in the regioselectivity model. This catalyst is the only one that brings steric bulk in the equatorial plane of the catalysts (right in front of the central metal). While a descriptor screening this bulk could have been developed, the lack of other examples would have precluded testing its validity. For a more extensive reasoning about the effects of steric bulk in the equatorial plane of the catalyst see *Macromolecules* **2018**, *51*, 8073-8083.

The **molecular weight model does not include** catalyst **M21**, as the experimentally determined M_W (1 kDa) is below the lower limit of the calibration range of the GPC measurements (3 kDa).

Catalysts with σ > 0.9995 (1-σ < 0.05) are not included in the stereoselectivity model. See main paper for details.

QSAR Modeling – Descriptor Pool

Table S4. Descriptor list and reasons for inclusion of the descriptor in the descriptor pool.

#	Descriptor	Reason for inclusion in descriptor package
D1	**%$V_{Bur,Zr}$**<br>The difference of hindered volume between open and closed quadrants of a 5.0 Å sphere centered on Zr analyzes the separation of steric bulk in the active pocket.	**Simple, yet powerful, descriptor based on possible orientations of the inserting monomer in 1,2 orientation; analyzes the space around the metal where the $Me_{propene}$ groups can be located.**<br><br>Established concept for stereoselectivity (see Dalton Trans. 2009, (41), 8885-8890 and Macromolecules 2018, 51, 8073-8083) and molecular weight capability (see ACS Catal. 2015, 6815-6822). For the connection of stereoselectivity and regioselectivity/molecular weight capability see Macromolecules 2018, 51, 8073-8083.
D2	**%$V_{Bur(C2+3)}$**<br>The sum of hindered volumes in NE and SE quadrants of a 3.5 Å sphere centered on C3 (*Note: this descriptor accounts for the distribution of overall steric bulk at the 2- and 3-position of the indenyl fragment*)	**2-position substituents are spatially close to $Me_{propene}$ in 2,1-insertion TS and to the propene α-carbon in both chain transfer to monomer TS and 1,2 insertion TS. The close proximity of $Me_{propene}$ to 2-R substituent can lead to distortions even if the bulk of 2-R is oriented towards the back of the catalyst.**<br><br>2-position substituents have dramatic effects on molecular regioselectivity and molecular weight capability. However, bulkier substituents have opposite effects on both performance indicators.
D3	**%$V_{Bur(C2+C3),Front}$**<br>The hindered volume in NE quadrant of a 3.5 Å sphere centered on Cl (*Note: this descriptor accounts for the fraction of overall steric bulk at the 2- and 3-position of the indenyl fragment oriented towards the active site*)	
D4	**%$V_{Bur(C5+C6)}$**<br>The hindered volume in SE quadrant of a 6.5 Å sphere centered on Cl (*Note: this descriptor accounts for the steric bulk contributions from remote 5- and 6-position of the indenyl fragment*)	**The interference of $Me_{propene}$ with the indenyl backbone and/or the 2-R substituent distorts the 2,1 insertion geometry with the chain going up and the inserting monomer going down. This increases $H_{monomer}$-$H_{6\text{-}R}$ contact distances. This region of space comes into closer contact with the bulkier chain transfer to monomer TS and with substituents in 2-position.**<br><br>Bulky substituents in 6-position have an evident and opposing experimental effects on regioselectivity and molecular weight capability
D5	**%$V_{Bur,C4}$**<br>The hindered volume in SW quadrant of a 3.5 Å sphere centered 2.0 Å away from the Cl atom on an extension of the Zr-Cl bond (*Note: this descriptor accounts for remote steric bulk originating from the substituent at 4-position*)	**Substituents in this region of space do not interfere with the preferred insertion pathway. However, the bulkier chain transfer to monomer TS or the spatial change of the polymeryl chain in the 2,1 insertion TS (see Figure S40 for an illustration) lead to close contacts.**<br><br>Both regioselectivity and molecular weight capability are positively influenced.
D6	**%$V_{Bur,open}$**<br>The hindered volume in NW quadrant of a 3.0 Å sphere centered 1.5 Å in front of the Zr atom (Note: this descriptor measures the steric bulk in the open quadrants)	**Steric bulk in the open quadrants reduces the quadrant difference. This affects the preferred insertion pathway. Steric bulk obstructing this region has more dramatic effects than the quadrant difference alone would estimate.**<br><br>Molecular weight in particular is dramatically reduced upon 3-position substitution.
D7	**q_{ZrCl2}.**<br>The NPA charge on the $ZrCl_2$ fragment	The electrophilicity of the metal could affect regio and/or molecular weight capability.

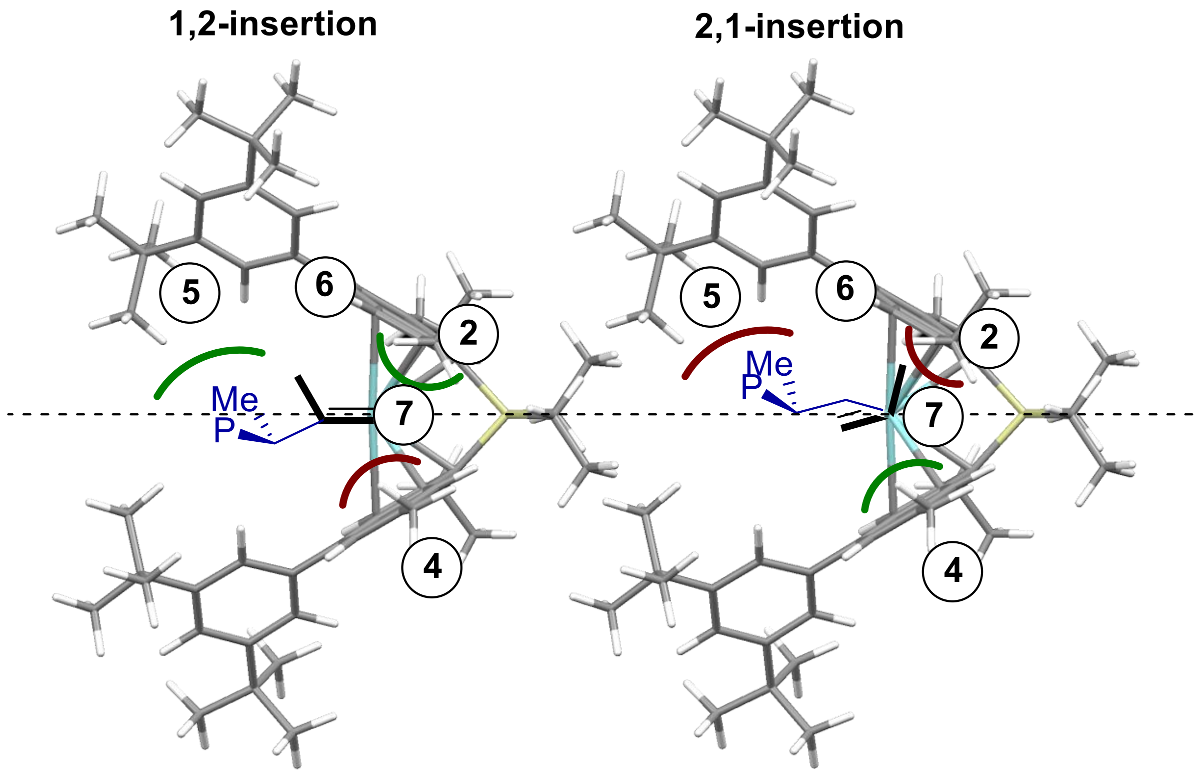

Figure S40. Differences between propene 1,2 and 2,1 insertion TS for **M39**. Dichloride geometry (ligand environment) overlaid with line drawing depiction of the TS geometry. Numbers reflect approximate positions of the scanning spheres used by the descriptors and are numbered as in **Figure 5** and **Table 8** (main paper). The distortion of the TS for 2,1 insertion (olefin down, chain up) with respect to 1,2 insertion (olefin and chain in one plane) brings the chain closer to remote steric bulk on 4-R substituents, but moves the C-H propene bonds further away from steric bulk in 5- and 6-position. Therefore, steric bulk in certain regions affects one of the two TS more (red curved lines) and one less (green curved lines).

Table S5. Procedures for Descriptor Determination.

#	Descriptor	SambVca (D1-D6) or Gaussian (D7) procedure	
**D1**	$\Delta\%V_{Bur,Zr}$	Coordinate System	center of the Sphere: Zr, z-axis: Si-Zr, xz-plane: Cl
		Deleted Atoms	$ZrCl_2$ + SiR_2 bridge + all atoms in 5-, 6-, and 7-position + additional atoms on the 2-position substituent (for details see Table S7 and Figures S41-S43)
		Sphere Size and Position	5.0 Å, Center on Zr
**D2**	$\%V_{Bur(C2+C3)}$	Coordinate System	center of the Sphere: C3, z-axis: middle of the two opposite C-atoms, xz-plane: C2
		Deleted Atoms	$ZrCl_2$, SiR_2 bridge
		Sphere Size and Position	3.5 Å, Center on C3
**D3**	$\%V_{Bur(C2+C3),Front}$	Coordinate System	center of the Sphere: Cl, z-axis: Zr-Cl, xz-plane: Si
		Deleted Atoms	$ZrCl_2$
		Sphere Size and Position	3.5 Å, Center on Cl
**D4**	$\%V_{Bur(C5+C6)}$	Coordinate System	center of the Sphere: Cl, z-axis: Zr-Cl, xz-plane: Si
		Deleted Atoms	$ZrCl_2$, SiR_2 bridge
		Sphere Size and Position	6.5 Å, Center on Cl
**D5**	$\%V_{Bur,C4}$	Coordinate System	center of the Sphere: Cl, z-axis: Zr-Cl, xz-plane: Si
		Deleted Atoms	$ZrCl_2$
		Sphere Size and Position	3.5 Å, Center +2 Å away from Cl
**D6**	$\%V_{Bur,open}$	Coordinate System	center of the Sphere: Zr, z-axis: Si-Zr, xz-plane: Cl
		Deleted Atoms	$ZrCl_2$
		Sphere Size and Position	3.0 Å, Center +1.5 Å away from Cl
**D7**	q_{ZrCl2}	Sum of the NPA charges (NBO 3.0) on Zr and Cl atoms*	

* Charge Model 5 and Hirshfeld charges were also tested but provide a worse correlation.

QSAR Modeling – Determination of Gibbs Free Energy Differences

Equations

Stereoselectivity:

$\Delta\Delta G^{\ddagger}_{enantio,\ exp}$ = RT ln (σ/(1-σ)) (Eq. S1)

Regioselectivity:

$\Delta\Delta G^{\ddagger}_{regiotot,\ exp}$ = RT ln ((*regio*$_{tot}$/100)/1-(*regio*$_{tot}$/100) (Eq. S2)

Molecular Weight Capability:

$\Delta\Delta G^{\ddagger}_{T,\ exp}$ = RT ln (M_n *1000/$MW_{propene}$) (Eq. S3)

Table S6. Experimental data used for QSAR modeling. Catalysts ordered by increasing molecular weight capability.

	***regio*$_{tot}$ (mol%)**	***σ***	***M$_n$* *(kDa)***	**$\Delta\Delta G^{\ddagger}_{regiotot, exp}$ (kcal/mol)**	**$\Delta\Delta G^{\ddagger}_{enantio, exp}$ (kcal/mol)**	**$\Delta\Delta G^{\ddagger}_{T, exp}$ (kcal/mol)**
Training Set						
**M21**	N/A	N/A	1	N/A	N/A	2.10
**M28**	N/A	N/A	3	N/A	N/A	2.89
**M36**	0.09	0.972	19	4.67	2.35	4.05
**M13**	0.40	0.9997	80	3.67	5.37	5.00
**M7**	0.67	0.9960	80	3.33	3.65	5.00
**M18**	0.66	0.9959	90	3.34	3.64	5.08
**M1**	0.29	0.9865	100	3.89	2.84	5.15
**M8**	0.74	0.9962	100	3.26	3.69	5.15
**M22**	0.90	0.9917	130	3.13	3.17	5.32
**M6**	0.82	0.9960	140	3.19	3.65	5.37
**M3**	0.91	0.992	140	3.12	3.19	5.37
**M17**	0.79	0.9963	140	3.22	3.70	5.37
**M5**	0.53	0.9960	150	3.49	3.65	5.41
**M37**	0.21	0.9989	220	4.11	4.51	5.67
**M4**	0.50	0.9971	230	3.53	3.87	5.70
**M10**	0.40	0.9986	230	3.67	4.35	5.70
**M11**	0.30	0.9986	250	3.87	4.35	5.75
**M15**	0.42	0.9984	290	3.64	4.26	5.85
**M12**	0.32	0.9989	290	3.82	4.51	5.85
**M31**	1.41	0.9967	290	2.83	3.78	5.85
**M9**	0.29	0.9989	320	3.89	4.51	5.92
**M2**	0.32	0.9988	320	3.82	4.45	5.92
**M23**	0.28	0.9991	380	3.91	4.64	6.03
**M29**	0.23	0.9997	400	4.04	5.37	6.06
**M16**	0.18	0.9994	410	4.21	4.91	6.08
**M19**	0.42	0.9999	470	3.64	6.10	6.17
**M26**	0.43	0.9991	480	3.63	4.64	6.18
**M27**	0.98	0.9978	490	3.07	4.05	6.20
**M35**	0.36	0.9997	510	3.75	5.37	6.22
**M14**	0.17	0.9994	530	4.25	4.91	6.25
**M20**	0.48	0.9999	610	3.55	6.10	6.34
**M32**	1.05	0.9969	620	3.03	3.82	6.35
**M24**	0.54	0.999	630	3.47	4.57	6.36
**M25**	0.69	0.9989	710	3.31	4.51	6.44
**M38**	0.20	0.9996	800	4.14	5.18	6.52
**M34**	0.83	0.9998	990	3.19	5.64	6.66
**M33**	0.30	0.9995	950	3.87	5.03	6.64
**M30**	0.71	0.9997	1400	3.29	5.37	6.89

Table S6 (ctd.). Experimental data used for QSAR modeling. Catalysts ordered by increasing molecular weight capability.

	regio$_{tot}$ **(mol%)**	$\boldsymbol{\sigma}$	M_n ***(kDa)***	**$\Delta\Delta G^{‡}_{regiotot, exp}$ (kcal/mol)**	**$\Delta\Delta G^{‡}_{enantio, exp}$ (kcal/mol)**	**$\Delta\Delta G^{‡}_{T, exp}$ (kcal/mol)**
Optimized Catalysts						
**M39**	0.18	0.9996	740	4.21	5.18	6.47
**M40**	0.11	0.9995	620	4.54	5.03	6.35
**M41**	0.35	0.9996	760	3.76	5.18	6.49
**M42**	0.20	0.9995	550	4.14	5.03	6.27
**M43**	0.17	0.9997	470	4.35	5.37	6.17

QSAR Modeling – %V$_{Bur,Zr}$ determination

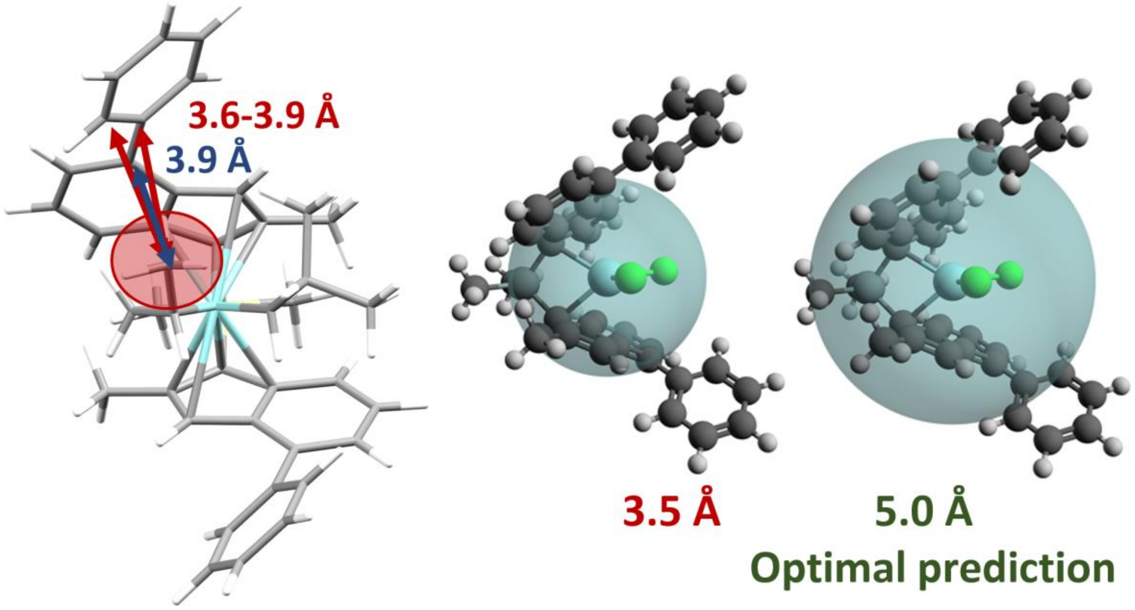

Figure S41. Rationale for increased scanning sphere size to determine %$V_{Bur,Zr}$ on the example of **M2**. Steric bulk further away from the active pocket, i.e. 4-Ph *ipso*- and *ortho*-carbons are as close or closer to the $Me_{propene}$ group in the TS leading to stereoerrors than the carbon in 4-posiiton of the indenyl fragment. A larger sphere is needed to account for these influences. See *Macromolecules* **2018**, *51*, 8073-8083 for further details.

Catalyst	6-position substituent	*σ*
**M2**	H	0.9988
**M23**	Me	0.9991
**M24**	iPr	0.9990
**M25**	tBu	0.9989
**M26**	Ph	0.9991

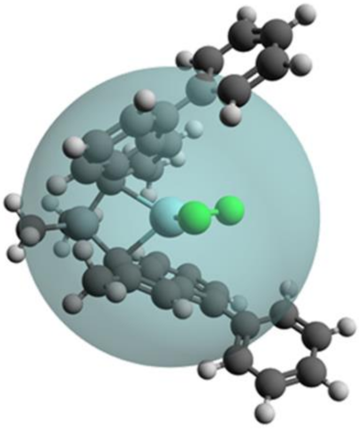

Figure S42. Rationale for deletion of atoms or groups in 5-, 6-, 7-position and the Si-bridge to determine %$V_{Bur,Zr}$. Due to the increased scanning sphere size necessary to screen steric influence originating from the 4-position, other positions which are inconsequential for stereoselectivity (see examples for various 6-position substituents) are also screened. To correct for this bias, these atoms or groups were deleted.

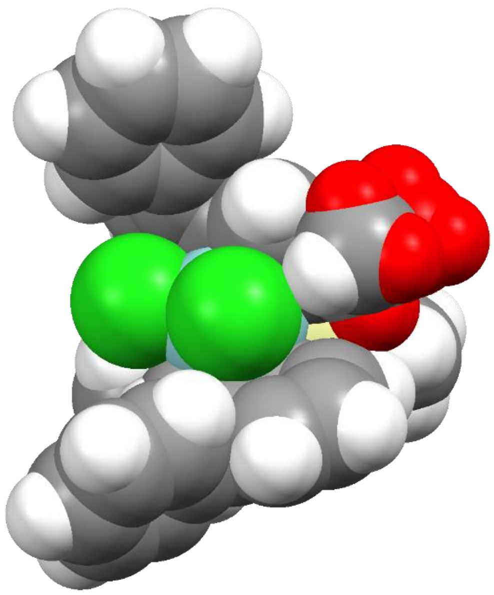

Figure S43. Exclusion of atoms of 2-position substituents not in line-of-sight of the active center for calculation of $\%V_{Bur,Zr}$. Space filling model of **M36**. Additionally deleted atoms are marked in red. The deleted atoms point to the back of the catalyst and other atoms in front of them already block the space. This approach avoids ‘double counting’ of steric bulk. Picture generated with Mercury 4.2

QSAR Modeling – Equations for Stereo-, Regioselectivity and Molecular Weight Capability Models

Stereoselectivity Model

$\Delta\Delta G^{\ddagger}_{\text{enantio, exp}} =$ (Eq. S4)

$0.474\ \Delta\%V_{\text{Bur, Zr}} - 3.247$

Molecular Weight Model

$\Delta\Delta G^{\ddagger}_{\text{T, exp}} =$ (Eq. S5)

$0.127\ \Delta\%V_{\text{Bur, Zr}} + 0.023\ \%V_{\text{Bur, C4}} + 0.039\ \%V_{\text{Bur, C5-6}} - 0.220\ \%V_{\text{Bur, open}} - 0.099\ \%V_{\text{Bur, C2-3-Front}} + 13.971$

Regioselectivity Model

$\Delta\Delta G^{\ddagger}_{\text{regiotot, exp}} =$ (Eq. S6)

$-14.267\ e_{\text{-ZrCl}_2\text{, NPA}} + 0.043\ \%V_{\text{Bur, C2-3-All}} + 0.031\ \%V_{\text{Bur, C4}} - 0.031\ \%V_{\text{Bur, C5-6}} - 0.529\ \%V_{\text{Bur, open}} + 23.619$

QSAR Modeling – Stereoselectivity Model

Table S7. Stereoselectivity (σ and $\Delta\Delta G^{\ddagger}_{\text{enantio, exp}}$), $\%V_{\text{Bur, Zr}}$, and predicted $\Delta\Delta G^{\ddagger}_{\text{enantio, QSAR}}$.

Catalyst	deleted atoms*	σ_{exp}	$\Delta\Delta G^{\ddagger}_{\text{enantio, exp}}$ (kcal/mol)	$\Delta\%V_{\text{Bur, Zr}}$	$\Delta\Delta G^{\ddagger}_{\text{enantio, QSAR}}$ (kcal/mol)
**M36**	2-iPr: far H, far Me, 2 far H on close Me	0.9720	2.35	11.40	2.15
**M7**	2-Me: far H	0.9960	3.65	15.40	4.05
**M18**	2-Me: far H	0.9959	3.64	15.30	4.00
**M1**	2-Me: far H	0.9865	2.84	12.80	2.82
**M8**	2-Me: far H	0.9962	3.69	15.00	3.86
**M22**	2-Me: far H	0.9917	3.17	13.10	2.96
**M6**	2-Me: far H	0.9960	3.65	14.80	3.76
**M3**	2-Me: far H	0.9920	3.19	13.30	3.05
**M17**	2-Me: far H	0.9963	3.70	14.80	3.76
**M5**	2-Me: far H	0.9960	3.65	14.90	3.81
**M37**	2-Et: far Me	0.9989	4.51	16.20	4.43
**M4**	2-Me: far H	0.9971	3.87	15.30	4.00
**M10**	2-Me: far H	0.9986	4.35	16.10	4.38
**M11**	2-Me: far H	0.9986	4.35	16.10	4.38
**M15**	2-Me: far H	0.9984	4.26	16.30	4.47
**M12**	2-Me: far H	0.9989	4.51	16.30	4.47
**M31**	2-Me: far H	0.9967	3.78	14.90	3.81
**M9**	2-Me: far H	0.9989	4.51	16.30	4.47
**M2**	2-Me: far H	0.9988	4.45	16.20	4.43
**M23**	2-Me: far H	0.9991	4.64	16.30	4.47
**M16**	2-Me: far H	0.9994	4.91	16.80	4.71
**M26**	2-Me: far H	0.9991	4.64	16.30	4.47
**M27**	2-Me: far H	0.9978	4.05	16.00	4.33
**M14**	2-Me: far H	0.9994	4.91	16.80	4.71
**M32**	2-Me: far H	0.9969	3.82	15.20	3.95
**M24**	2-Me: far H	0.9990	4.57	16.20	4.43
**M25**	2-Me: far H	0.9989	4.51	16.20	4.43
**M33**	2-Me: far H	0.9995	5.03	16.70	4.66
		**Not Included in the model (σ > 0.9995)**			
**M30**	2-Me: far H	0.9998	5.37	17.60	5.09
**M13**	2-Me: far H	0.9997	5.37	19.00	5.75
**M29**	2-Me: far H	0.9997	5.37	16.20	4.43
**M19**	2-Me: far H	>0.9998	>5.64	17.60	5.09
**M35**	2-Me: far H	0.9997	5.37	17.90	5.23
**M20**	2-Me: far H	>0.9998	>5.64	17.65	5.11
**M34**	2-Me: far H	>0.9998	>5.64	17.70	5.14
**M38**	2-Me: far H	0.9996	5.18	18.20	5.37

*5-, 6-, 7-position substituents and the bridge were generally deleted for $\Delta\%V_{\text{Bur, Zr}}$ determination.

Table S8. Stereoselectivity Model Deviations.

Catalyst	$\|\Delta\|_{exp\text{-}pred}$ (kcal/mol)	$\|\Delta\|^2_{exp\text{-}pred}$	σ_{QSAR}	$\|\Delta\|_{exp\text{-}pred}$	$\|\Delta\|^2_{exp\text{-}pred}$
**M36**	0.20	0.04	0.96126	0.01074	0.00012
**M7**	0.39	0.16	0.99778	0.00178	0.00000
**M18**	0.36	0.13	0.99762	0.00172	0.00000
**M1**	0.03	0.00	0.98577	0.00073	0.00000
**M8**	0.17	0.03	0.99705	0.00085	0.00000
**M22**	0.21	0.04	0.98852	0.00318	0.00001
**M6**	0.11	0.01	0.99660	0.00060	0.00000
**M3**	0.14	0.02	0.99005	0.00195	0.00000
**M17**	0.06	0.00	0.99660	0.00030	0.00000
**M5**	0.16	0.02	0.99683	0.00083	0.00000
**M37**	0.08	0.01	0.99875	0.00015	0.00000
**M4**	0.13	0.02	0.99762	0.00052	0.00000
**M10**	0.03	0.00	0.99866	0.00006	0.00000
**M11**	0.03	0.00	0.99866	0.00006	0.00000
**M15**	0.21	0.04	0.99884	0.00044	0.00000
**M12**	0.04	0.00	0.99884	0.00006	0.00000
**M31**	0.03	0.00	0.99683	0.00013	0.00000
**M9**	0.04	0.00	0.99884	0.00006	0.00000
**M2**	0.03	0.00	0.99875	0.00005	0.00000
**M23**	0.17	0.03	0.9988	0.0003	0.00000
**M16**	0.20	0.04	0.9992	0.0002	0.00000
**M26**	0.17	0.03	0.9988	0.0003	0.00000
**M27**	0.28	0.08	0.9986	0.0008	0.00000
**M14**	0.20	0.04	0.9992	0.0002	0.00000
**M32**	0.13	0.02	0.9974	0.0005	0.00000
**M24**	0.15	0.02	0.9988	0.0002	0.00000
**M25**	0.08	0.01	0.9988	0.0001	0.00000
**M33**	0.37	0.14	0.9991	0.0004	0.00000
	**Not Included in the model (σ > 0.9995)**				
**M30**	0.55	0.30	0.9995	0.0003	0.00000
**M13**	0.38	0.15	0.9998	0.0001	0.00000
**M29**	0.94	0.89	0.9988	0.0009	0.00000
**M19**	0.55	0.30	0.9995	0.0003	0.00000
**M35**	0.14	0.02	0.9996	0.0001	0.00000
**M20**	0.53	0.28	0.9996	0.0002	0.00000
**M34**	0.50	0.25	0.9996	0.0002	0.00000
**M38**	0.19	0.04	0.9997	0.0001	0.00000

Table S9. Mean average deviation (MAD), mean squared error (MSE) and root mean squared error (RMSE) for $\Delta\Delta G^{‡}_{\text{enantio QSAR}}$ and σ_{QSAR}.

$\Delta\Delta G^{‡}_{\text{enantio, QSAR}}$		σ_{QSAR}	
MAD	0.15 kcal/mol	MAD	0.0010
MSE	0.03 kcal^2/mol^2	MSE	0.0000
RMSE	0.18 kcal/mol	RMSE	0.0022

QSAR Modeling – Stereoselectivity Model, Analysis of Variance

SUMMARY OUTPUT

Regression Statistics	
Multiple R	0.9588573
R Square	0.91940733
Adjusted R Square	0.91630761
Standard Error	0.18943287
Observations	28

ANOVA

	df	*SS*	*MS*	*F*	*Significance F*
Regression	1	10.64379365	10.64379	296.61	9.7503E-16
Residual	26	0.933005107	0.035885		
Total	27	11.57679875			

	Coefficients	*Standard Error*	*t Stat*	*P-value*	*Lower 95%*	*Upper 95%*
Intercept	-3.2466414	0.424780994	-7.64309	4.12E-08	-4.1197912	-2.373492
X Variable 1	0.47357732	0.027497804	17.22237	9.75E-16	0.41705477	0.5300999

QSAR Modeling – Stereoselectivity Model, Leave-One-Out Analysis

MEAN = mean experimental value; MAD = mean average deviation; MSE = mean squared error; RMSE = root mean squared error; q^2 = cross-validated R^2

Table S10. LOOCV analysis stereoselectivity model.

	Catalyst	Yintercept	slopeA	predictedEXP	actualEXP	R^2	adj. R^2	|Δ|	Δ^2	(actualEXP-MEAN)^2
1	M36	-3.65944	0.499654	2.04	2.35	0.90	0.89	0.31	0.10	2.87
2	M7	-3.22867	0.47335	4.06	3.65	0.93	0.93	0.41	0.17	0.15
3	M18	-3.21789	0.47256	4.01	3.64	0.93	0.93	0.37	0.14	0.16
4	M1	-3.26831	0.474903	2.81	2.84	0.91	0.90	0.03	0.00	1.45
5	M8	-3.21406	0.47185	3.86	3.69	0.92	0.92	0.17	0.03	0.12
6	M22	-3.43628	0.485307	2.92	3.17	0.92	0.92	0.25	0.06	0.76
7	M6	-3.21565	0.471822	3.77	3.65	0.92	0.92	0.12	0.01	0.15
8	M3	-3.3552	0.48025	3.03	3.19	0.92	0.91	0.16	0.02	0.73
9	M17	-3.22756	0.472475	3.77	3.7	0.92	0.92	0.07	0.00	0.12
10	M5	-3.20986	0.47156	3.82	3.65	0.92	0.92	0.17	0.03	0.15
11	M37	-3.22215	0.471765	4.42	4.51	0.92	0.92	0.09	0.01	0.22
12	M4	-3.2336	0.473026	4.00	3.87	0.92	0.92	0.13	0.02	0.03
13	M10	-3.24799	0.473718	4.38	4.35	0.92	0.92	0.03	0.00	0.09
14	M11	-3.24799	0.473718	4.38	4.35	0.92	0.92	0.03	0.00	0.09
15	M15	-3.30033	0.477571	4.48	4.26	0.92	0.92	0.22	0.05	0.05
16	M12	-3.23208	0.472525	4.47	4.51	0.92	0.91	0.04	0.00	0.22
17	M31	-3.23636	0.472968	3.81	3.78	0.92	0.92	0.03	0.00	0.07
18	M9	-3.23208	0.472525	4.47	4.51	0.92	0.91	0.04	0.00	0.22
19	M2	-3.23642	0.472838	4.42	4.45	0.92	0.92	0.03	0.00	0.17
20	M23	-3.19659	0.469901	4.46	4.64	0.92	0.92	0.18	0.03	0.36
27	M16	-3.15067	0.466823	4.69	4.91	0.92	0.91	0.22	0.05	0.75
21	M26	-3.19659	0.469901	4.46	4.64	0.92	0.92	0.18	0.03	0.36
22	M27	-3.28962	0.477036	4.34	4.05	0.93	0.92	0.29	0.09	0.00
26	M14	-3.15067	0.466823	4.69	4.91	0.92	0.91	0.22	0.05	0.75
23	M32	-3.22898	0.472733	3.96	3.82	0.92	0.92	0.14	0.02	0.05
24	M24	-3.20788	0.470691	4.42	4.57	0.92	0.92	0.15	0.02	0.28
25	M25	-3.22215	0.471765	4.42	4.51	0.92	0.92	0.09	0.01	0.22
28	M33	-3.08828	0.462355	4.63	5.03	0.93	0.92	0.40	0.16	0.97
					**MEAN**			**MAD**	**MSE**	
					**4.04**			**0.16**	**0.04**	
					**MAX**					
					**5.03**					
					**MIN**					
					**2.35**					

q^2=Σ(actualEXP-MEAN)-Σ(Δ^2)/Σ(actualEXP-MEAN)

q^2 **0.90**

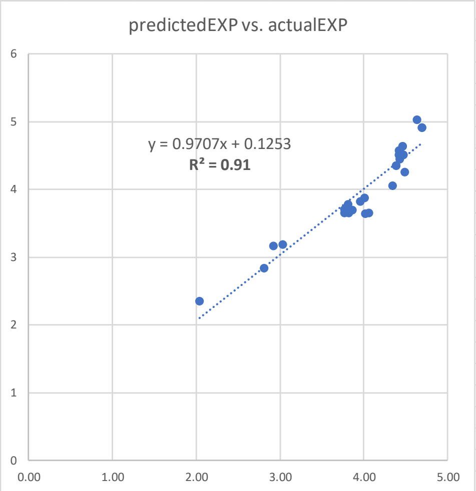

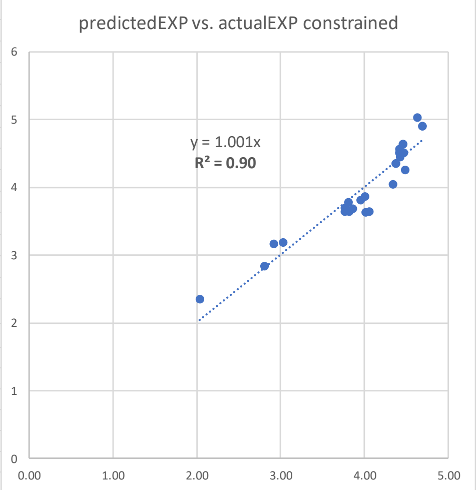

QSAR Modeling – Molar Mass Capability Model, Single Descriptor Model

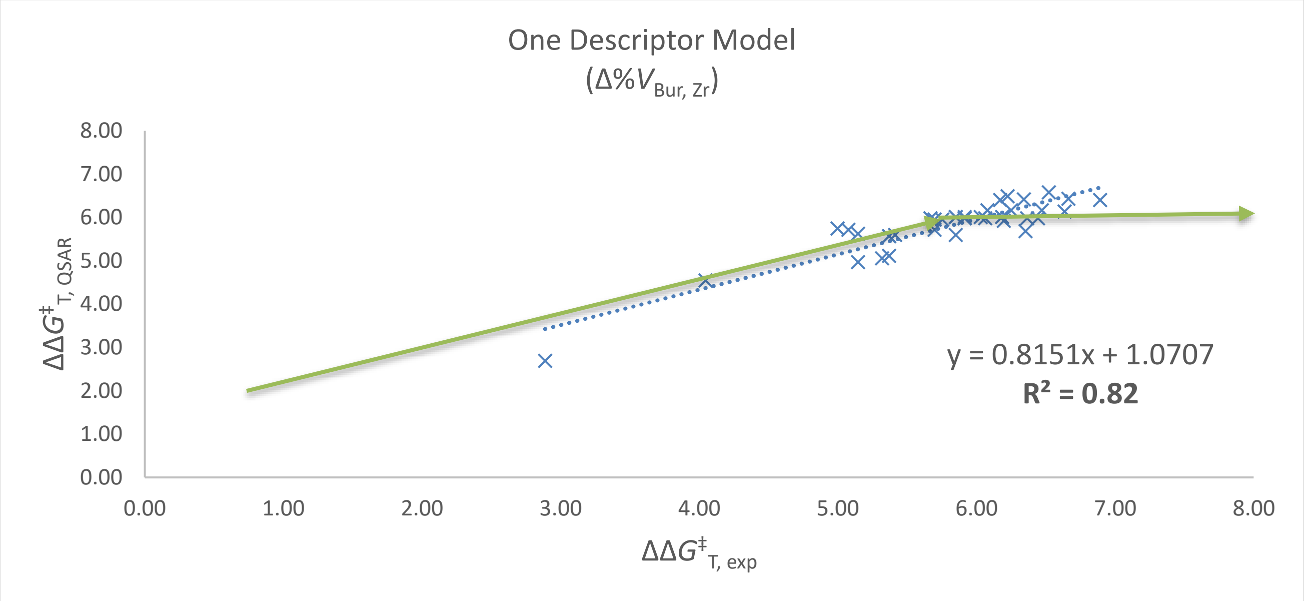

Figure S44. Correlation of predicted ($\Delta\Delta G^{‡}_{T, QSAR}$) and experimentally observed stereoselectivity ($\Delta\Delta G^{‡}_{T, exp}$) using a single descriptor model based on $\Delta\%V_{Bur, Zr}$ (5.0 Å scanning sphere) showing that predicted molecular weight reaches a maximum before experimental molecular weight does. Green arrows added as guide for the eye.

Regression Statistics					
Multiple R	0.902822				
R Square	0.815087				
Adjusted R Square	0.809804				
Standard Error	0.329586				
Observations	37				
ANOVA					
	df	*SS*	*MS*	*F*	*Significance F*
Regression	1	16.75883	16.75883	154.2787	2.1902E-14
Residual	35	3.801946	0.108627		
Total	36	20.56078			
	Coefficients	*andard Err*	*t Stat*	*P-value*	*l*
Intercept	1.133794	0.378769	2.993361	0.005035	
X Variable 1	0.299179	0.024087	12.4209	2.19E-14	

QSAR Modeling – Molar Mass Capability Model, Final Model

Table S11. Molar mass capability (M_n and $\Delta\Delta G^{\ddagger}_{T,\ exp}$), values for all 5 descriptors, and predicted $\Delta\Delta G^{\ddagger}_{T,\ QSAR}$.

	M_n (kDa)	$\Delta\%V_{Bur,\ Zr}$	$\%V_{Bur,\ C4}$	$\%V_{Bur,\ C5\text{-}6}$	$\%V_{Bur,\ open}$	$\%V_{Bur,\ C2\text{-}3\text{-}Front}$	$\Delta\Delta G^{\ddagger}_{T,\ exp}$ (kcal/mol)	$\Delta\Delta G^{\ddagger}_{T,\ QSAR}$ (kcal/mol)
**M28**	3	5.2	7.8	56.9	40.1	54.7	2.89	2.82
**M36**	19	11.4	19.0	40.7	34.3	55.9	4.05	4.38
**M7**	80	15.4	7.5	39.2	35.5	45.9	5.00	5.30
**M18**	90	15.3	7.5	39.1	35.5	45.8	5.08	5.29
**M1**	100	12.8	4.0	38.8	34.6	46.5	5.15	5.01
**M8**	100	15.0	16.0	41.7	36.2	45.9	5.15	5.39
**M22**	130	13.1	9.5	40.1	34.9	46.6	5.32	5.15
**M6**	140	14.8	7.5	39.6	34.7	46.5	5.37	5.35
**M3**	140	13.3	9.5	40.1	34.9	46.6	5.37	5.17
**M17**	140	14.8	7.5	39.6	34.7	46.5	5.37	5.35
**M5**	150	14.9	6.9	39.5	34.6	46.3	5.41	5.39
**M37**	220	16.2	18.5	40.3	34.8	46.9	5.67	5.75
**M4**	230	15.3	18.0	40.4	34.7	46.4	5.70	5.70
**M10**	230	16.1	17.9	40.2	34.7	46.6	5.70	5.77
**M11**	250	16.1	18.1	40.1	34.8	46.6	5.75	5.75
**M15**	290	16.3	19.1	40.1	34.8	46.5	5.85	5.81
**M12**	290	16.3	18.7	40.1	34.8	46.6	5.85	5.79
**M31**	290	14.9	7.6	57.4	34.7	47.0	5.85	6.02
**M9**	320	16.3	18.4	40.1	34.8	46.5	5.92	5.79
**M2**	320	16.2	18.7	40.1	34.8	46.4	5.92	5.80
**M23**	380	16.3	18.5	46.8	34.8	46.5	6.03	6.06
**M29**	400	16.2	31.9	38.7	35.4	46.5	6.06	5.90
**M16**	410	16.8	26.4	41.5	34.7	47.0	6.08	6.07
**M19**	470	17.6	20.2	41.7	34.9	46.8	6.17	6.01
**M26**	480	16.3	18.5	55.0	34.7	46.5	6.18	6.40
**M27**	490	16.0	18.2	58.4	34.7	52.9	6.20	5.86
**M35**	510	17.9	20.4	48.6	34.8	46.8	6.22	6.35
**M14**	530	16.8	33.1	40.2	34.8	46.6	6.25	6.19
**M20**	610	17.65	19.8	46.4	34.9	46.5	6.34	6.22
**M32**	620	15.2	18.3	58.3	34.7	47.4	6.35	6.30
**M24**	630	16.2	18.6	54.7	34.8	47.4	6.36	6.27
**M25**	710	16.2	18.7	58.0	34.8	47.3	6.44	6.41
**M38**	800	18.2	38.2	42.1	34.4	47.2	6.52	6.59
**M34**	990	17.7	20.5	59.8	35.0	47.3	6.66	6.67
**M33**	950	16.7	33.8	58.1	34.8	47.2	6.64	6.84
**M30**	1400	17.6	22.3	63.6	34.8	47.5	6.89	6.87

Table S12. Molar Mass Capability Model Deviations.

	$\|\Delta\|_{exp\text{-}pred}$ (kcal/mol)	$\|\Delta\|^2_{exp\text{-}pred}$	$M_{n,\ QSAR}$ kDa	$\|\Delta\|_{exp\text{-}pred}$	$\|\Delta\|^2_{exp\text{-}pred}$
**M28**	0.07	0.005	3	0	0
**M36**	0.33	0.112	31	12	155
**M7**	0.30	0.088	125	45	2031
**M18**	0.21	0.045	124	34	1143
**M1**	0.14	0.020	81	19	371
**M8**	0.24	0.058	143	43	1886
**M22**	0.17	0.030	100	30	906
**M6**	0.02	0.000	136	4	16
**M3**	0.20	0.039	104	36	1310
**M17**	0.02	0.000	136	4	16
**M5**	0.03	0.001	144	6	39
**M37**	0.08	0.007	248	28	801
**M4**	0.00	0.000	230	0	0
**M10**	0.07	0.005	256	26	696
**M11**	0.00	0.000	248	2	3
**M15**	0.04	0.002	271	19	352
**M12**	0.06	0.004	263	27	703
**M31**	0.17	0.028	372	82	6792
**M9**	0.12	0.015	265	55	3061
**M2**	0.12	0.014	266	54	2879
**M23**	0.03	0.001	396	16	250
**M29**	0.16	0.025	314	86	7424
**M16**	0.01	0.000	402	8	69
**M19**	0.16	0.026	368	102	10380
**M26**	0.22	0.048	667	187	34845
**M27**	0.34	0.117	292	198	39303
**M35**	0.12	0.015	612	102	10442
**M14**	0.06	0.004	483	47	2236
**M20**	0.12	0.015	507	103	10569
**M32**	0.05	0.003	570	50	2523
**M24**	0.09	0.009	545	85	7256
**M25**	0.03	0.001	675	35	1191
**M38**	0.07	0.004	882	82	6770
**M34**	0.01	0.000	1000	10	96
**M33**	0.20	0.041	1287	337	113801
**M30**	0.02	0.000	1358	42	1761

Table S13. Mean average deviation (MAD), mean squared error (MSE) and root mean squared error (RMSE) for $\Delta\Delta G^{\ddagger}_{T\ QSAR}$ and $M_{n,\ QSAR}$.

$\Delta\Delta G^{\ddagger}_{T,\ QSAR}$		$M_{n,\ QSAR}$	
MAD	0.11 kcal/mol	MAD	56 kDa
MSE	0.02 kcal²/mol²	MSE	8179 kDa²
RMSE	0.15 kcal/mol	RMSE	90 kDa

QSAR Modeling – Molar Mass Capability Model, Analysis of Variance

SUMMARY OUTPUT								
Regression Statistics								
Multiple R	0.98034							
R Square	0.961066							
Adjusted R	0.954577							
Standard E	0.161448							
Observatic	36							
ANOVA								
	df	*SS*	*MS*	*F*	*Significance F*			
Regressior	5	19.30234201	3.860468403	148.1065586	3.33615E-20			
Residual	30	0.781964372	0.026065479					
Total	35	20.08430638						
	Coefficients	*Standard Error*	*t Stat*	*P-value*	*Lower 95%*	*Upper 95%*	*ower 95.0%*	*Ipper 95.0%*
Intercept	13.97101	2.373299691	5.88674517	1.91429E-06	9.124085902	18.81794	9.124086	18.81794
X Variable	0.127257	0.028983763	4.390623626	0.000129274	0.068064055	0.18645	0.068064	0.18645
X Variable	0.02314	0.004834894	4.786113139	4.25936E-05	0.013266178	0.033015	0.013266	0.033015
X Variable	0.039415	0.003938418	10.0077167	4.49378E-11	0.031371252	0.047458	0.031371	0.047458
X Variable	-0.21952	0.046156553	-4.756010549	4.63634E-05	-0.313785309	-0.12526	-0.31379	-0.12526
X Variable	-0.09937	0.019726592	-5.037355638	2.09645E-05	-0.139656935	-0.05908	-0.13966	-0.05908

QSAR Modeling – Molar Mass Capability Model, Leave-One-Out Analysis

MEAN = mean experimental value; MAD = mean average deviation; MSE = mean squared error; RMSE = root mean squared error; q^2 = cross-validated R^2

Table S14. LOOCV analysis molar mass capability model.

	Catalyst	Yintercept	slopeA	slopeB	slopeC	slopeD	slopeE	predictedEXP	actualEXP	R^2	adj. R^2	|Δ|	Δ^2	$(\text{actualEXP-MEAN})^2$
**1**	M28	18.30555	0.144665	0.021307	0.03796	-0.34122	-0.10514	1.95	2.89	0.94	0.93	0.938	0.879	8.314
**2**	M36	15.5847	0.103846	0.025356	0.032864	-0.34385	-0.02765	5.25	4.05	0.98	0.97	1.202	1.444	2.974
**3**	M7	12.90711	0.141316	0.020277	0.037971	-0.19639	-0.09594	5.35	5.00	0.97	0.96	0.350	0.123	0.597
**4**	M18	13.30312	0.136064	0.021248	0.038456	-0.20424	-0.09767	5.32	5.08	0.96	0.96	0.248	0.061	0.483
**5**	M1	13.0943	0.136763	0.023194	0.039201	-0.20462	-0.09491	4.97	5.15	0.96	0.95	0.180	0.032	0.391
**6**	M8	13.29046	0.131849	0.022913	0.038695	-0.19776	-0.10165	5.42	5.15	0.96	0.96	0.278	0.077	0.391
**7**	M22	13.08084	0.138198	0.022601	0.039163	-0.20609	-0.09376	5.11	5.32	0.96	0.96	0.205	0.042	0.204
**8**	M6	13.95714	0.127238	0.023234	0.039448	-0.21922	-0.09937	5.35	5.37	0.96	0.95	0.020	0.000	0.162
**9**	M3	13.0898	0.137935	0.02271	0.039216	-0.20624	-0.09386	5.14	5.37	0.96	0.96	0.228	0.052	0.162
**10**	M17	13.95714	0.127238	0.023234	0.039448	-0.21922	-0.09937	5.35	5.37	0.96	0.95	0.020	0.000	0.162
**11**	M5	13.94287	0.127261	0.023289	0.03946	-0.21892	-0.09934	5.38	5.41	0.96	0.95	0.030	0.001	0.127
**12**	M37	13.89691	0.12847	0.023058	0.039137	-0.21835	-0.09872	5.75	5.67	0.96	0.95	0.085	0.007	0.011
**13**	M4	13.97427	0.127219	0.023145	0.039413	-0.21957	-0.09939	5.70	5.70	0.96	0.95	0.002	0.000	0.005
**14**	M10	13.96832	0.127645	0.023118	0.039219	-0.21947	-0.09923	5.77	5.70	0.96	0.95	0.076	0.006	0.005
**15**	M11	13.97233	0.127229	0.023142	0.039424	-0.21955	-0.09938	5.75	5.75	0.96	0.95	0.003	0.000	0.000
**16**	M15	13.99365	0.126838	0.023143	0.039547	-0.22005	-0.09948	5.81	5.85	0.96	0.95	0.045	0.002	0.006
**17**	M12	14.01776	0.126452	0.02318	0.039618	-0.22043	-0.09967	5.79	5.85	0.96	0.95	0.065	0.004	0.006
**18**	M31	14.48356	0.123141	0.022356	0.041074	-0.2297	-0.10251	6.06	5.85	0.96	0.96	0.206	0.043	0.006
**19**	M9	14.05715	0.125709	0.023231	0.039808	-0.2213	-0.09985	5.79	5.92	0.96	0.96	0.131	0.017	0.021
**20**	M2	14.00041	0.126507	0.023124	0.03975	-0.22054	-0.09938	5.79	5.92	0.96	0.96	0.126	0.016	0.021
**21**	M23	13.98111	0.127245	0.023146	0.039443	-0.2196	-0.09953	6.06	6.03	0.96	0.95	0.029	0.001	0.067
**22**	M29	13.93867	0.13069	0.021167	0.040113	-0.22417	-0.09643	5.86	6.06	0.96	0.96	0.200	0.040	0.086
**23**	M16	13.97181	0.127253	0.023085	0.039448	-0.21955	-0.09938	6.07	6.08	0.96	0.95	0.013	0.000	0.095
**24**	M19	14.67147	0.117612	0.023971	0.040296	-0.23097	-0.1038	5.99	6.17	0.96	0.96	0.183	0.034	0.159
**25**	M26	14.43642	0.122772	0.023496	0.040891	-0.22692	-0.10367	6.43	6.18	0.96	0.96	0.242	0.058	0.171
**26**	M27	16.88305	0.086726	0.0287	0.039456	-0.232	-0.14083	5.60	6.20	0.97	0.97	0.601	0.361	0.182
**27**	M35	13.55082	0.133472	0.02255	0.039283	-0.21302	-0.09691	6.36	6.22	0.96	0.95	0.136	0.019	0.206
**28**	M14	13.86853	0.129095	0.022363	0.039579	-0.21875	-0.09829	6.18	6.25	0.96	0.95	0.074	0.006	0.229
**29**	M20	14.36554	0.121591	0.023666	0.039645	-0.22628	-0.10134	6.21	6.34	0.96	0.95	0.133	0.018	0.327
**30**	M32	13.71718	0.130234	0.022872	0.038834	-0.21505	-0.09766	6.29	6.35	0.96	0.95	0.066	0.004	0.339
**31**	M24	13.9047	0.127654	0.023149	0.03894	-0.21791	-0.0989	6.26	6.36	0.96	0.95	0.102	0.010	0.352
**32**	M25	13.92543	0.127673	0.023119	0.039174	-0.21863	-0.09899	6.41	6.44	0.96	0.95	0.036	0.001	0.452
**33**	M38	13.99609	0.126683	0.024015	0.039172	-0.22003	-0.09937	6.61	6.52	0.96	0.95	0.086	0.007	0.565
**34**	M34	13.9495	0.127571	0.023106	0.03946	-0.2192	-0.09929	6.67	6.66	0.96	0.95	0.009	0.000	0.797
**35**	M33	14.99944	0.112376	0.026652	0.04163	-0.23428	-0.10859	6.92	6.64	0.96	0.96	0.281	0.079	0.749
**36**	M30	13.98542	0.126945	0.023165	0.039234	-0.21959	-0.09937	6.87	6.89	0.96	0.95	0.023	0.001	1.259
									**MEAN**			**MAD**	**MSE**	
									**5.77**			**0.18**	**0.10**	
									**MAX**					
									**6.89**					
									**MIN**					
									**2.89**					

$q^2 = \Sigma(\text{actualEXP-MEAN}) - \Sigma(\Delta^2)/\Sigma(\text{actualEXP-MEAN})$

q^2	**0.83**

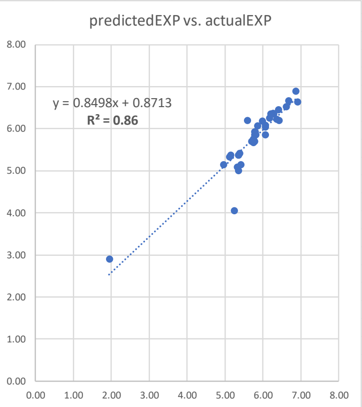

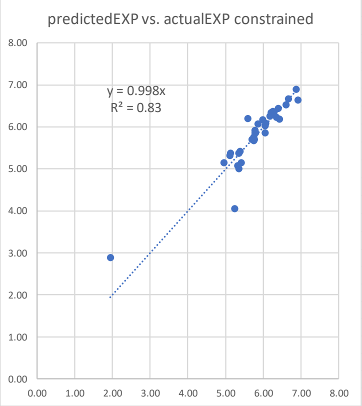

QSAR Modeling – Regioselectivity, Final Model

Table S15. Regioselectivity (*regio*$_{tot}$ and $\Delta\Delta G^{\ddagger}_{regiotot, exp}$), values for all 5 descriptors, and predicted $\Delta\Delta G^{\ddagger}_{regiotot, QSAR}$.

	regio$_{tot}$	$e^{-}_{ZrCl2, NPA}$	**%*V***$_{Bur, C2\text{-}3\text{-}All}$	**%*V***$_{Bur, C4}$	**%*V***$_{Bur, C5\text{-}6}$	**%*V***$_{Bur, open}$	$\Delta\Delta G^{\ddagger}_{regiotot, exp}$	$\Delta\Delta G^{\ddagger}_{regiotot, QSAR}$
**M36**	0.09	0.43049	141.0	19.0	40.7	34.3	4.67	4.70
**M7**	0.67	0.38406	118.1	7.5	39.2	35.5	3.33	3.44
**M18**	0.66	0.38582	118.1	7.5	39.1	35.5	3.34	3.42
**M1**	0.29	0.39032	117.8	4.0	38.8	34.6	3.89	3.71
**M8**	0.74	0.39090	118.1	16.0	41.7	36.2	3.26	3.16
**M22**	0.90	0.41691	118.0	9.5	40.1	34.9	3.13	3.32
**M6**	0.82	0.42025	117.7	7.5	39.6	34.7	3.19	3.32
**M3**	0.91	0.41500	118.1	9.5	40.1	34.9	3.12	3.35
**M17**	0.79	0.42209	117.8	7.5	39.6	34.7	3.22	3.29
**M5**	0.53	0.41368	117.8	6.9	39.5	34.6	3.49	3.45
**M37**	0.21	0.42189	127.1	18.5	40.3	34.8	4.11	3.96
**M4**	0.50	0.41789	118.1	18.0	40.4	34.7	3.53	3.67
**M10**	0.40	0.4117	117.8	17.9	40.2	34.7	3.67	3.75
**M11**	0.30	0.41192	117.8	18.1	40.1	34.8	3.87	3.70
**M15**	0.42	0.41390	118.1	19.1	40.1	34.8	3.64	3.72
**M12**	0.32	0.41332	117.8	18.7	40.1	34.8	3.82	3.70
**M31**	1.41	0.41091	117.7	7.6	57.4	34.7	2.83	2.90
**M9**	0.29	0.41178	117.8	18.4	40.1	34.8	3.89	3.71
**M2**	0.32	0.41191	118.1	18.7	40.1	34.8	3.82	3.73
**M23**	0.28	0.40469	118.0	18.5	46.8	34.8	3.91	3.62
**M29**	0.23	0.39728	120.2	31.9	38.7	35.4	4.04	4.17
**M16**	0.18	0.41509	118.0	26.4	41.5	34.7	4.21	3.94
**M19**	0.42	0.41418	118.4	20.2	41.7	34.9	3.64	3.66
**M26**	0.43	0.41180	117.9	18.5	55.0	34.7	3.63	3.32
**M27**	0.98	0.40457	117.8	18.2	58.4	34.7	3.07	3.30
**M35**	0.36	0.41800	118.2	20.4	48.6	34.8	3.75	3.44
**M14**	0.17	0.40819	119.2	33.1	40.2	34.8	4.25	4.28
**M20**	0.48	0.39857	117.8	19.8	46.4	34.9	3.55	3.70
**M32**	1.05	0.40988	118.0	18.3	58.3	34.7	3.03	3.24
**M24**	0.54	0.40385	118.0	18.6	54.7	34.8	3.47	3.39
**M25**	0.69	0.40338	118.0	18.7	58.0	34.8	3.31	3.30
**M38**	0.20	0.41882	118.6	38.2	42.1	34.4	4.14	4.42
**M34**	0.83	0.40482	118.3	20.5	59.8	35.0	3.19	3.19
**M33**	0.30	0.39839	119.2	33.8	58.1	34.8	3.87	3.89
**M30**	0.71	0.39993	117.9	22.3	63.6	34.8	3.29	3.29

Table S16. Regioselectivity Model Deviations.

	$\|\Delta\|_{exp\text{-}pred}$ (kcal/mol)	$\|\Delta\|^2_{exp\text{-}pred}$	*regio*$_{tot,\ QSAR}$ (mol%)	$\|\Delta\|_{exp\text{-}pred}$	$\|\Delta\|^2_{exp\text{-}pred}$
**M36**	0.03	0.0011	0.082133	0.007867	0.00
**M7**	0.11	0.0119	0.555021	0.114979	0.01
**M18**	0.08	0.0059	0.573802	0.086198	0.01
**M1**	0.17	0.0305	0.365528	0.075528	0.01
**M8**	0.10	0.0105	0.84522	0.10522	0.01
**M22**	0.19	0.0348	0.666005	0.233995	0.05
**M6**	0.12	0.0149	0.667992	0.152008	0.02
**M3**	0.23	0.0508	0.635034	0.274966	0.08
**M17**	0.07	0.0056	0.690542	0.099458	0.01
**M5**	0.04	0.0013	0.544618	0.014618	0.00
**M37**	0.14	0.0198	0.250798	0.040798	0.00
**M4**	0.15	0.0212	0.390627	0.109373	0.01
**M10**	0.07	0.0056	0.346935	0.053065	0.00
**M11**	0.16	0.0269	0.372281	0.072281	0.01
**M15**	0.08	0.0059	0.363442	0.056558	0.00
**M12**	0.12	0.0149	0.37294	0.05294	0.00
**M31**	0.08	0.0056	1.244498	0.165502	0.03
**M9**	0.18	0.0308	0.365925	0.075925	0.01
**M2**	0.09	0.0080	0.35484	0.03484	0.00
**M23**	0.29	0.0855	0.421465	0.141465	0.02
**M29**	0.13	0.0164	0.18312	0.04688	0.00
**M16**	0.27	0.0740	0.261751	0.081751	0.01
**M19**	0.02	0.0003	0.397246	0.022754	0.00
**M26**	0.31	0.0968	0.668532	0.238532	0.06
**M27**	0.23	0.0511	0.684113	0.295887	0.09
**M35**	0.30	0.0910	0.550868	0.190868	0.04
**M14**	0.04	0.0014	0.155005	0.014995	0.00
**M20**	0.15	0.0216	0.374086	0.105914	0.01
**M32**	0.21	0.0447	0.750147	0.299853	0.09
**M24**	0.08	0.0066	0.594957	0.054957	0.00
**M25**	0.01	0.0001	0.683466	0.006534	0.00
**M38**	0.28	0.0788	0.126304	0.073696	0.01
**M34**	0.00	0.0000	0.810113	0.019887	0.00
**M33**	0.03	0.0007	0.279221	0.020779	0.00
**M30**	0.00	0.0000	0.698842	0.011158	0.00

Table S17. Mean average deviation (MAD), mean squared error (MSE) and root mean squared error (RMSE) for $\Delta\Delta G^{\ddagger}_{\text{regiotot, QSAR}}$ and *regio*$_{\text{tot}}$.

$\Delta\Delta G^{\ddagger}_{\text{regiotot, QSAR}}$		***regio***$_{\text{tot, QSAR}}$	
MAD	0.13 kcal/mol	MAD	0.10 mol%
MSE	0.02 kcal2/mol	MSE	0.02 mol%2
RMSE	0.16 kcal/mol	RMSE	0.13 mol%

QSAR Modeling – Regioselectivity Model, Analysis of Variance

SUMMARY OUTPUT								
Regression Statistics								
Multiple R	0.921653							
R Square	0.849445							
Adjusted F	0.823487							
Standard E	0.173701							
Observatic	35							
ANOVA								
	df	*SS*	*MS*	*F*	*Significance F*			
Regressior	5	4.936750878	0.987350176	32.72401827	4.46554E-11			
Residual	29	0.874988972	0.030172034					
Total	34	5.81173985						
	Coefficients	*Standard Error*	*t Stat*	*P-value*	*Lower 95%*	*Upper 95%*	*ower 95.0%*	*Ipper 95.0%*
Intercept	23.61944	5.839914409	4.044484535	0.000354496	11.67547746	35.56341	11.67548	35.56341
X Variable	-14.2674	4.227817528	-3.374654199	0.002115558	-22.9142799	-5.62056	-22.9143	-5.62056
X Variable	0.042727	0.007873359	5.426801508	7.75044E-06	0.02662433	0.05883	0.026624	0.05883
X Variable	0.031339	0.003820138	8.203598781	4.79959E-09	0.023525818	0.039152	0.023526	0.039152
X Variable	-0.0308	0.004153022	-7.417043301	3.57773E-08	-0.039297028	-0.02231	-0.0393	-0.02231
X Variable	-0.52888	0.127285206	-4.155060371	0.000262187	-0.789205189	-0.26855	-0.78921	-0.26855

QSAR Modeling – Regioselectivity Model, Leave-One-Out Analysis

Table S18. LOOCV analysis molar mass capability model.

	Catalyst	Yintercept	slopeA	slopeB	slopeC	slopeD	slopeE	predictedEXP	actualEXP	R^2	adj. R^2	|Δ|	$Δ^2$	$(actualEXP\text{-}MEAN)^2$
**1**	M36	23.06339	-14.54	0.052376	0.030871	-0.03068	-0.54239	4.92	4.67	0.81	0.78	0.253	0.064	1.135
**2**	M7	23.95987	-15.3617	0.043569	0.030826	-0.03132	-0.52763	3.48	3.33	0.85	0.82	0.149	0.022	0.076
**3**	M18	23.74047	-14.9115	0.043251	0.030984	-0.03113	-0.5259	3.44	3.34	0.85	0.82	0.102	0.010	0.070
**4**	M1	16.3964	-9.02291	0.041522	0.033268	-0.02808	-0.38385	3.53	3.89	0.86	0.83	0.361	0.131	0.081
**5**	M8	28.29275	-15.5758	0.04194	0.031281	-0.03142	-0.6443	3.02	3.26	0.85	0.82	0.239	0.057	0.117
**6**	M22	22.64587	-13.2335	0.041853	0.030578	-0.03087	-0.50944	3.34	3.13	0.85	0.82	0.210	0.044	0.224
**7**	M6	23.36186	-13.6274	0.041928	0.030682	-0.03094	-0.52564	3.34	3.19	0.85	0.82	0.142	0.020	0.169
**8**	M3	22.7663	-13.3298	0.041877	0.030449	-0.03097	-0.51161	3.37	3.12	0.85	0.83	0.248	0.062	0.231
**9**	M17	23.35164	-13.7716	0.042197	0.030921	-0.03085	-0.52485	3.31	3.22	0.85	0.82	0.089	0.008	0.149
**10**	M5	23.42434	-14.2422	0.042871	0.031531	-0.03069	-0.52435	3.45	3.49	0.85	0.82	0.041	0.002	0.014
**11**	M37	24.74384	-14.9179	0.040653	0.031401	-0.03083	-0.54656	3.94	4.11	0.85	0.82	0.170	0.029	0.250
**12**	M4	23.55112	-13.8227	0.041929	0.031399	-0.03113	-0.52889	3.68	3.53	0.85	0.83	0.156	0.024	0.006
**13**	M10	23.90957	-14.3292	0.042411	0.031385	-0.03108	-0.535	3.75	3.67	0.85	0.82	0.080	0.006	0.005
**14**	M11	23.42257	-14.3625	0.043425	0.031219	-0.03031	-0.52523	3.69	3.87	0.85	0.83	0.173	0.030	0.069
**15**	M15	23.60661	-14.1371	0.042396	0.031427	-0.03101	-0.52862	3.72	3.64	0.85	0.82	0.081	0.007	0.001
**16**	M12	23.58723	-14.4433	0.043308	0.031218	-0.03046	-0.52837	3.69	3.82	0.85	0.82	0.129	0.017	0.048
**17**	M31	23.46778	-14.0362	0.042624	0.030761	-0.03012	-0.5274	2.92	2.83	0.83	0.80	0.092	0.009	0.602
**18**	M9	23.39141	-14.352	0.043472	0.031183	-0.03026	-0.52466	3.70	3.89	0.85	0.83	0.185	0.034	0.081
**19**	M2	23.51487	-14.3087	0.043054	0.031249	-0.03053	-0.52689	3.73	3.82	0.85	0.82	0.094	0.009	0.048
**20**	M23	22.53027	-13.5109	0.043001	0.031233	-0.03065	-0.50782	3.61	3.91	0.86	0.84	0.305	0.093	0.095
**21**	M29	23.0142	-14.6455	0.043368	0.032685	-0.03153	-0.50887	4.22	4.04	0.85	0.82	0.173	0.030	0.193
**22**	M16	23.2702	-14.6189	0.044296	0.03001	-0.02992	-0.52081	3.91	4.21	0.85	0.83	0.300	0.090	0.364
**23**	M19	23.55739	-14.2072	0.042662	0.031366	-0.03082	-0.52755	3.66	3.64	0.85	0.82	0.019	0.000	0.001
**24**	M26	24.04918	-15.0023	0.043505	0.031602	-0.0325	-0.53345	3.29	3.63	0.87	0.84	0.339	0.115	0.000
**25**	M27	24.19917	-14.6264	0.042715	0.031067	-0.02939	-0.54275	3.33	3.07	0.85	0.83	0.255	0.065	0.282
**26**	M35	25.4641	-16.1405	0.044147	0.031127	-0.03179	-0.56356	3.41	3.75	0.87	0.84	0.332	0.110	0.020
**27**	M14	23.77925	-14.3795	0.042657	0.031693	-0.03103	-0.53176	4.29	4.25	0.84	0.81	0.045	0.002	0.411
**28**	M20	24.45728	-15.0525	0.042774	0.031536	-0.03104	-0.54353	3.71	3.55	0.85	0.83	0.159	0.025	0.003
**29**	M32	23.34959	-13.8715	0.042447	0.031055	-0.02924	-0.52651	3.27	3.03	0.85	0.82	0.239	0.057	0.333
**30**	M24	23.48572	-14.1432	0.042713	0.031378	-0.03114	-0.5261	3.39	3.47	0.85	0.82	0.088	0.008	0.017
**31**	M25	23.61194	-14.2569	0.042721	0.031346	-0.03086	-0.5287	3.30	3.31	0.85	0.82	0.010	0.000	0.087
**32**	M38	25.99365	-14.5487	0.040218	0.03531	-0.03277	-0.58427	4.54	4.14	0.86	0.84	0.403	0.162	0.284
**33**	M34	23.60343	-14.2603	0.04273	0.031338	-0.03078	-0.52854	3.19	3.19	0.84	0.82	0.003	0.000	0.176
**34**	M33	23.75601	-14.4302	0.042831	0.031562	-0.0307	-0.53147	3.90	3.87	0.85	0.82	0.034	0.001	0.069
**35**	M30	23.61266	-14.2558	0.042718	0.031336	-0.03085	-0.52873	3.28	3.29	0.85	0.82	0.006	0.000	0.099
									**MEAN**			**MAD**	**MSE**	
									**3.60**			**0.16**	**0.04**	
									**MAX**					
									**4.67**					
									**MIN**					
									**2.83**					
								**q^2=Σ(actualEXP-MEAN)-Σ($Δ^2$)/Σ(actualEXP-MEAN)**						
									**q^2**	**0.77**				

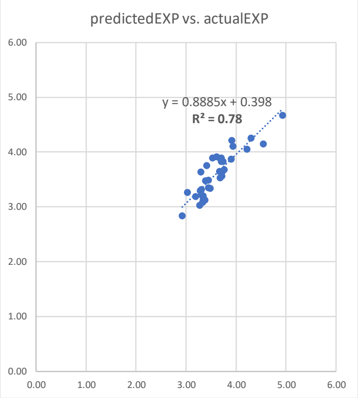

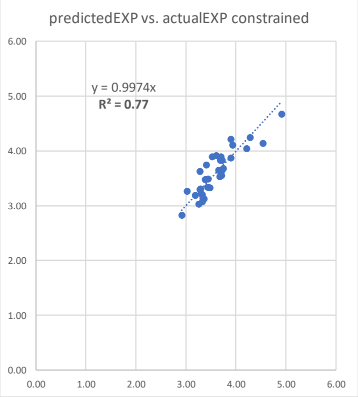

QSAR Modeling – Screened Catalyst Candidates and Predicted Performance

Table S19. Predicted performance for selected catalysts, including **M39**-**M43**.

Cat	Δ%$V_{Bur,Zr}$	%$V_{Bur,C2\text{-}3}$	%$V_{Bur,C2\text{-}3\text{-}Front}$	%$V_{Bur,C5\text{-}6}$	%$V_{Bur,C4}$	%$V_{Bur,open}$	$e^-_{ZrCl2,\ NPA}$	$\Delta\Delta G^{\ddagger}_{enantio}$	$\Delta\Delta G^{\ddagger}_{regiotot}$	$\Delta\Delta G^{\ddagger}_{T}$	*σ*	*regio*$_{tot}$	M_n
2-cyPr-4-(3,5-tBu)Ph-6-Me	10.9	140.2	57.2	40.1	34.8	34.5	0.41105	1.79	5.04	4.76	0.9330	0.05	56
2-Et-4-(3,5-tBu)Ph-6-nPent	16.5	127.8	46.9	47.0	36.5	35.0	0.41033	4.68	4.16	6.61	0.9992	0.19	910
2-iPr-4-(3,5-tBu)Ph-6-Me	11.0	141.9	55.8	51.7	36.3	34.4	0.41605	1.84	5.12	4.99	0.9380	0.04	79
2-Me-4-(5-Me)thienyl	17.5	119.1	46.6	47.6	23.4	34.6	0.42381	5.20	3.72	6.15	0.9996	0.37	452
2-Me-4-C_5F_6-6-Ad	16.5	118.0	47.8	41.4	26.7	34.9	0.40424	4.68	3.26	6.72	0.9992	0.73	1071
2-Me-4-C_5F_6-5-iPr	17.8	117.8	47.3	61.9	29.1	34.8	0.40627	5.36	3.52	6.77	0.9997	0.49	1159
2-Me-4-(3,5-CF_3)Ph	16.3	118.1	46.4	55.8	26.8	34.7	0.41463	4.58	3.89	6.03	0.9990	0.28	377
2-Me-4-(2-Me-5-tBu)Ph	18.7	118.3	46.8	40.3	42.2	35.2	0.40628	5.82	4.19	6.61	0.9998	0.18	915
2-Me-4-(3,5-tBu)Ph-6-TriPhSi	15.9	118.7	52.6	42.2	33.9	34.9	0.39705	4.37	3.52	6.47	0.9986	0.49	740
2-Me-4-(3,5-TMS)Ph-6-tBu	16.5	119.2	47.1	65.5	39.4	34.8	0.39927	4.68	3.95	6.96	0.9992	0.26	1539
2-Me-4-C_5F_6-6-tBu	16.8	118.2	47.5	58.2	26.5	34.7	0.40343	4.84	3.45	6.73	0.9993	0.54	1092
2-Me-4-C_5F_6-6-TMS	16.9	118.1	47.2	59.5	26.5	34.7	0.41181	4.89	3.29	6.82	0.9994	0.69	1243
2-Me-4-C_5F_6-6-TriPhSi	17.1	118.2	50.5	60.6	26.5	34.8	0.40494	4.99	3.12	6.74	0.9995	0.90	1108
2-Me-4-(2-tBu)Ph	16.9	118.1	47.0	66.9	17.3	38.4	0.41145	4.89	1.54	5.18	0.9994	9.71	105
2-Me-4-(4-pyridyl)	16.0	117.8	46.6	44.6	18.1	34.7	0.41491	4.43	3.61	5.76	0.9988	0.43	252
2-Me-4-(4-pyridyl)/$AlMe_3$-adduct	16.2	118.0	46.5	40.1	18.4	34.6	0.41651	4.53	3.65	5.85	0.9989	0.40	289
2-Me-4-(4-pyridyl)-6-TMS/$AlMe_3$-adduct	16.1	118.0	46.8	40.8	18.5	34.7	0.41440	4.48	3.05	6.48	0.9988	1.00	746
2-Me-4-Ph-5-iPr	17.4	117.7	46.6	58.3	19.5	35.5	0.41440	5.15	2.75	6.40	0.9996	1.57	664
2-Me-4-Ph-5-tBu	14.8	117.7	46.5	55.5	24.5	39.6	0.39069	3.81	1.04	5.33	0.9968	20.75	132
2-Me-4-Ph-6-CF_3	16.3	118.0	47.3	56.4	18.3	34.6	0.42791	4.58	3.11	6.22	0.9990	0.91	505
2-Me-4-Ph-6-CN/$AlMe_3$-adduct	16.0	117.9	46.9	51.9	18.2	34.3	0.44166	4.43	3.14	6.17	0.9988	0.87	471
2-Me-4-(5-pyrimidyl)	16.3	117.9	46.4	49.1	18.9	34.8	0.41055	4.58	3.64	5.80	0.9990	0.41	269
2-nPent-4-(3,5-tBu)Ph-6-Me	16.7	147.5	47.3	39.8	39.5	35.4	0.42403	4.79	4.82	6.40	0.9993	0.07	667
2-Me-4-(3,5-Ad)Ph	16.3	118.6	46.7	47.3	35.2	34.9	0.40860	4.58	4.16	6.13	0.9990	0.19	444
2-Et-4-(2-Me)Ph	17.6	127.5	47.1	40.0	20.3	34.9	0.42349	5.25	3.80	5.99	0.9996	0.32	357
2-iPr-4-(3,5-tBu)Ph-6-tBu	11.1	141.8	56.5	41.9	37.2	34.3	0.41715	1.89	4.85	5.41	0.9427	0.07	148
2-Me-4-(3,5-tBu)Ph-6-Me-7-NMe_2	15.4	119.5	46.6	58.5	36.2	36.5	0.41543	4.12	2.90	6.25	0.9980	1.26	529
2-Me-4-Ph-5-Et	17.5	117.7	46.6	53.9	27.9	35.9	0.39291	5.20	3.32	6.25	0.9996	0.66	527
2-Me-4-Ph-5-Cl-6tBu	17.7	117.7	47.2	48.6	19.6	35.1	0.40337	5.30	2.94	6.78	0.9997	1.18	1172
2-Et-4-(3,5-tBu)Ph-6-Me (**M39**)	16.8	119.6	46.3	63.3	33.5	34.8	0.40132	4.84	4.21	6.49	0.9993	0.14	758
2-Et-4-(3,5-tBu)Ph-6-Me (**M40**)	16.8	128.6	46.8	40.1	33.7	35.0	0.40985	4.84	4.36	6.41	0.9993	0.17	675
2-Me-4-Ph-5-OMe (**M41**)	16.9	117.9	46.7	47.5	19.3	34.6	0.41167	4.76	3.63	6.20	0.9992	0.42	494
2-Me-4-(3,5-TMS)Ph (**M42**)	16.9	119.2	46.6	39.9	37.1	35.0	0.40703	4.76	4.33	6.24	0.9992	0.14	520
2-Me-4-[3,5-(1-Ad)-4-OMe]Ph_A (**M43**)	18.4	119.3	46.6	39.8	51.5	35.1	0.40473	5.47	4.77	6.74	0.9997	0.07	1104
2-Me-4-[3,5-(1-Ad)-4-OMe]Ph_B (**M43**)	16.1	118.3	46.5	41.0	35.5	34.8	0.40642	4.38	4.32	6.20	0.9987	0.15	489

Abbreviations: cyPr cyclopropyl, nPent neopentyl, 1-Ad 1-adamantly, TriPhSi triphenylsilyl, TMS trimethylsilyl

Table S20. Estimated synthetic complexity, including **M39**-**M43**.

Cat	Synthetic Complexity
2-cyPr-4-(3,5-tBu)Ph-6-Me	1
2-Et-4-(3,5-tBu)Ph-6-nPent	1
2-iPr-4-(3,5-tBu)Ph-6-Me	1
2-Me-4-(5-Me)thienyl	1
2-Me-4-C_5F_6-6-Ad	5
2-Me-4-C_5F_6-5-iPr	2
2-Me-4-(3,5-CF_3)Ph	1
2-Me-4-(2-Me-5-tBu)Ph	1
2-Me-4-(3,5-tBu)Ph-6-TriPhSi	4
2-Me-4-(3,5-TMS)Ph-6-tBu	1
2-Me-4-C_5F_6-6-tBu	1
2-Me-4-C_5F_6-6-TMS	5
2-Me-4-C_5F_6-6-TriPhSi	5
2-Me-4-(2-tBu)Ph	2
2-Me-4-(4-pyridyl)	4
2-Me-4-(4-pyridyl)/$AlMe_3$-adduct	4
2-Me-4-(4-pyridyl)-6-TMS/$AlMe_3$-adduct	4
2-Me-4-Ph-5-iPr	2
2-Me-4-Ph-5-tBu	5
2-Me-4-Ph-6-CF_3	4
2-Me-4-Ph-6-CN/$AlMe_3$-adduct	5
2-Me-4-(5-pyrimidyl)	4
2-nPent-4-(3,5-tBu)Ph-6-Me	1
2-Me-4-(3,5-Ad)Ph	Derivative Possible (**M43**)
2-Et-4-(2-Me)Ph	1
2-iPr-4-(3,5-tBu)Ph-6-tBu	1
2-Me-4-(3,5-tBu)Ph-7-NMe_2	3
2-Me-4-Ph-5-Et	4
2-Me-4-Ph-5-Cl-6tBu	4
2-Me-4-(3,5-tBu)Ph-6-Me (**M39**)	1
2-Et-4-(3,5-tBu)Ph-6-Me (**M40**)	1
2-Me-4-Ph-5-OMe (**M41**)	1
2-Me-4-(3,5-TMS)Ph (**M42**)	1
2-Me-4-[3,5-(1-Ad)-4-OMe]Ph (**M43**)	4

Ranking: possible – 1 – 2 – 3 –4 – 5 – very challenging/impossible

This ranking reflects personal opinions, based on local or commercial availability of precursor compounds, perceived ease of synthesis – overall and for individual steps – and overall synthetic effort.

4. Full Gaussian Citation

Gaussian 16, Revision A.03, Frisch, M. J.; Trucks, G. W.; Schlegel, H. B.; Scuseria, G. E.; Robb, M. A.; Cheeseman, J. R.; Scalmani, G.; Barone, V.; Petersson, G. A.; Nakatsuji, H.; Li, X.; Caricato, M.; Marenich, A. V.; Bloino, J.; Janesko, B. G.; Gomperts, R.; Mennucci, B.; Hratchian, H. P.; Ortiz, J. V.; Izmaylov, A. F.; Sonnenberg, J. L.; Williams-Young, D.; Ding, F.; Lipparini, F.; Egidi, F.; Goings, J.; Peng, B.; Petrone, A.; Henderson, T.; Ranasinghe, D.; Zakrzewski, V. G.; Gao, J.; Rega, N.; Zheng, G.; Liang, W.; Hada, M.; Ehara, M.; Toyota, K.; Fukuda, R.; Hasegawa, J.; Ishida, M.; Nakajima, T.; Honda, Y.; Kitao, O.; Nakai, H.; Vreven, T.; Throssell, K.; Montgomery, J. A., Jr.; Peralta, J. E.; Ogliaro, F.; Bearpark, M. J.; Heyd, J. J.; Brothers, E. N.; Kudin, K. N.; Staroverov, V. N.; Keith, T. A.; Kobayashi, R.; Normand, J.; Raghavachari, K.; Rendell, A. P.; Burant, J. C.; Iyengar, S. S.; Tomasi, J.; Cossi, M.; Millam, J. M.; Klene, M.; Adamo, C.; Cammi, R.; Ochterski, J. W.; Martin, R. L.; Morokuma, K.; Farkas, O.; Foresman, J. B.; Fox, D. J. Gaussian, Inc., Wallingford CT, 2016.
